# Rapid community-driven development of a SARS-CoV-2 tissue simulator

**DOI:** 10.1101/2020.04.02.019075

**Published:** 2020-04-05

**Authors:** Yafei Wang, Gary An, Andrew Becker, Chase Cockrell, Nicholson Collier, Morgan Craig, Courtney L. Davis, James Faeder, Ashlee N. Ford Versypt, Juliano F. Gianlupi, James A. Glazier, Randy Heiland, Thomas Hillen, Mohammad Aminul Islam, Adrianne Jenner, Bing Liu, Penelope A Morel, Aarthi Narayanan, Jonathan Ozik, Padmini Rangamani, Jason Edward Shoemaker, Amber M. Smith, Paul Macklin

## Abstract

The 2019 novel coronavirus, SARS-CoV-2, is an emerging pathogen of critical significance to international public health. Knowledge of the interplay between molecular-scale virus-receptor interactions, single-cell viral replication, intracellular-scale viral transport, and emergent tissue-scale viral propagation is limited. Moreover, little is known about immune system-virus-tissue interactions and how these can result in low-level (asymptomatic) infections in some cases and acute respiratory distress syndrome (ARDS) in others, particularly with respect to presentation in different age groups or pre-existing inflammatory risk factors like diabetes. Given the nonlinear interactions within and among each of these processes, multiscale simulation models can shed light on the emergent dynamics that lead to divergent outcomes, identify actionable “choke points” for pharmacologic interactions, screen potential therapies, and identify potential biomarkers that differentiate patient outcomes. Given the complexity of the problem and the acute need for an actionable model to guide therapy discovery and optimization, we introduce and iteratively refine a prototype of a multiscale model of SARS-CoV-2 dynamics in lung tissue. The first prototype model was built and shared internationally as open source code and an online interactive model in under 12 hours, and community domain expertise is driving rapid refinements with a two-to-four week release cycle. In a sustained community effort, this consortium is integrating data and expertise across virology, immunology, mathematical biology, quantitative systems physiology, cloud and high performance computing, and other domains to accelerate our response to this critical threat to international health.

## Introduction

The ongoing pandemic caused by the novel severe acute respiratory syndrome coronavirus 2 (SARS-CoV-2) has illuminated the global public health threat posed by highly pathogenic coronaviruses that emerge from zoonotic sources. With the majority of the world's population immunologically naïve and no available antivirals or vaccines, over 860,000 infections and 42,000 deaths amassed by the end of March 2020[1]. Coronavirus disease 2019 (COVID-19)—caused by SARS-CoV-2 infection—is characterized by a range of respiratory symptoms, including fever and cough[2,3], that can progress to acute respiratory distress syndrome (ARDS) in some patients[4,5]. Age and comorbidities seem to be the main risk factors for development of severe disease[6-8]. However, the dynamics of virus replication, interaction with host immune responses, and spread within the respiratory tract are still being established. Because a vaccine may not be available for 9-18 months, there is a critical need to further understand the infection in order to quickly identify pharmacologic interventions and optimal therapeutic designs that work to lessen virus replication and disease severity. However, this requires an international community effort that integrates expertise across a variety of domains and a platform that can be iteratively updated as new information and data arises.

To aid this effort, we have assembled an international, multi-disciplinary coalition to rapidly develop an open-source, multi-scale tissue simulator that can be used to investigate mechanisms of intracellular viral replication, infection of epithelial cells, host immune response, and tissue damage. The aim of this project is to concentrate community modeling efforts to create a comprehensive multiscale simulation framework that can subsequently be calibrated, validated, and used to rapidly explore and optimize therapeutic interventions for COVID-19. Once the prototype has been completed (after several design iterations), this coalition will transition to maintain and support the simulation framework and aggregate calibrated/validated parameter values.

To address the acute need for rapid access to an actionable model, we are using a community-driven coalition and best open science practices to build and iteratively refine the model:

(1) **Open source and GitHub:** All simulation source code is shared as open source on GitHub, with well-defined, versioned and documented releases, and Zenodo-generated DOIs and archives.
(2) **Interactive cloud-hosted models:** Every prototype version is rapidly transformed into a cloud-hosted, interactive model to permit faster scientific communication across communities, particularly with virologists, immunologists, pharmacologists, and others who have essential insights but ordinarily would not directly run the simulation models.
(3) **Social media and virtual feedback:** We enlist community participation (feedback, modeling contributions, software contributions, and data contributions) through social media, virtual seminars, web-based forms, a dedicated Slack workspace, and weekly team meetings. We are particularly encouraging feedback and data contributions by domain experts in virology, epidemiology, and mathematical biology (with a focus
(4) **Frequent preprint updates:** Each model iteration is accompanied by a cloud-hosted, interactive app (see #2) and an updated preprint on *bioRxiv*.
(5) **Integration of feedback:** All community feedback is evaluated to plan the next set of model refinements and recorded in an updated *bioRxiv* preprint.

Our first test of this workflow saw a first proof-of-concept software release (Steps 1-2) in 12 hours, and the first integration of community feedback and preprint dissemination was complete within a week. We have begun integrating community feedback, and it is our intention to continue rapid iteration, with a new candidate model release every 14-21 days.

## Goals and guiding principles

This project is community-driven, including the following contributions:

1) **Community priorities:** The community helps define the driving research questions, definition of the project scope, and selection of critical biological components to be modeled.
2) **Consensus hypotheses:** The community drives a shared, clearly-written consensus specification of the underlying biological hypotheses.

3) **Mathematical modeling:** The community helps develop, review and refine the mathematical interpretation of the biological hypotheses.
4) **Computational implementation:** The computational implementation is shared as open source, with community definition of specifications, unit tests, coding, and code review.
5) **Community feedback:** Community feedback on the model realism, hypotheses, mathematics, computational implementation, and techniques is encouraged throughout the development process.
6) **Community parameter and data:** Community contributions of parameter estimates and data contributions are aggregated to assist in model development and constraint.

### Project scope

While by definition the project scope can be refined by the community, the initial project scope is to:

1) Develop the general computational framework sufficiently to address many of the community-driven research questions.
2) Deliver a working simulation framework for use by others to perform calibration and validation. That is, the prototyping aims of this project are complete once the model is capable of demonstrating essential biological behaviors qualitatively.
3) To provide a software framework whose underlying hypotheses, mathematics, and computational implementation have been rigorously assessed by appropriate domain experts.

In particular, while this project will work to constrain, estimate, and calibrate parameters to the greatest extent possible, it is *not* within scope to delay software release until full calibration and validation. Those tasks are within scope of fully funded teams with dedicated experiments.

This project aims to deliver software that one can reasonably expect to calibrate and validate, thus freeing funded investigations from expensive early software development while providing a broad community consensus on key biological hypotheses. By rapidly prototyping this software, we aim to accelerate many funded research efforts.

### Essential model components

As part of defining the project scope, we have identified the following critical model components:

1) Virus dissemination in epithelial tissue
2) Virus binding, endocytosis, replication, and exocytosis
3) Infected cell responses, including changes to metabolism, secreted signals, and death
4) Inflammatory response
5) Ramp up the immune response (particularly in lymph nodes)
6) Immune cell infiltration
7) Immune cell predation of infected and other cells.
8) Tissue damage by death of infected cells that can be regarded as an early surrogate marker for ARDS

### Guiding principles

The coalition aims to model not merely the disease endpoints, but the disease *dynamics*: this will allow scientists to investigate mechanistic "what if" questions on potential interventions: *What if* we could inhibit endocytosis? *What if* we could introduce a cytokine early or late in the disease course? *What if* the infected cell apoptosis could be accelerated or delayed?

To accomplish this, we use a modular design: an overall tissue-scale model integrates an array targeted *submodels* that simulate critical processes (e.g., receptor binding and trafficking; virus replication). Each submodel is clearly specified to enable interoperability, and to make it feasible to for subteams to simultaneously develop and test the model components in parallel. Throughout development, we use open source methodologies that enhance communication, transparency, and reproducibility. See **Box 1.**

**Guiding principles: motivation**

- The model should investigate the dynamics of infection and treatment, and not merely endpoints.
- The model should help the community ask "what if" questions to guide experiments and interventions[9,10].

**Guiding principles: approach**

- We will gather community consensus and pool efforts into a "standardized" model that captures key SARS-CoV-2 dynamics. We will supply this model to the community for parallel studies by multiple labs.
- The model framework can be built with relatively sparse data, relying upon domain expertise and observations to choose its general form and assess its qualitative behavior.
- The model will be modular. Each submodel will have well-defined inputs and outputs, allowing parallel development and replacement of submodels with improved versions without change to the overall integrated model or other submodels.
- As part of the model formulation, documentation, and dissemination, we will craft clearly delineated "conceptual model and hypotheses" to encourage development of independent models with independent methodologies and software frameworks.
- The submodels should be independently executable and verifiable, allowing parallel development.
- The overall model framework will periodically release numbered versions (distributions) that bundle the best working version of each submodel as it exists at the time of release, allowing end-users (the community) to use well-defined, well-tested snapshots of the project.
- The model (and known parameter values) must be made publicly available as open source for maximum public benefit.
- The model will be made publicly available as an interactive web app to encourage community participation, to accelerate scientific dissemination, and to increase public educational benefit.
- We will use rapid prototyping to encourage a fast develop-test-refine cycle to build expertise and gain community feedback.
- We will encourage data and parameter sharing throughout this effort and with the user community after the model's "completion."
- We will develop the model to a point that it has correct qualitative behavior so that calibration is likely to succeed. This is the "product" for use in subsequent investigations by multiple teams. See the ***scoping*** statements above.
- After the model prototyping is complete (the goal of this paper), we will enter a maintenance and support phase to fix bugs, support scientist users, and add features identified by user community.

**Box 1: Guiding principles for the rapid prototyping a modular, multiscale model.**

### Critical questions for the model framework

While the model may not be able to address all of these scientific questions, the community determined that the work should be driven by these questions, and that each iteration of the model framework should aim to be amenable to an increasing number of these questions. A list of community-selected questions driving this work is in **Box 2**.

## Key biology for the simulation model

This rapid prototyping effort brings together specialists from a broad variety of domains: virology and infectious diseases, mathematical biology, computer science, high performance computing, data science, and other disciplines. Therefore, it is critical that all members of the project have access to a clear description of underlying biology that drive the model's assumptions. In this section we outline key aspects of viral replication and host response in functional terms needed for development of agent-based, multi-scale and multi-physics models.

### Cell infection and viral replication

The key cell-level process is viral infection of a single cell, followed by replication to create new virions:

1. SARS-CoV-2 is a single-stranded enveloped RNA virus[11]. A virion (single virus particle) has a lipid coating (envelope) that protects the virus
2. when outside a cell (or host). Each virus has dozens of spike glycoproteins that bind to ACE2 (receptors) on select cell membranes[3,11].
3. Virions travel in the tissue microenvironment to reach a cell membrane. The spike binds to an available ACE2 receptor on the cell's membrane. Both passive transport (e.g., via diffusion in fluids) and active transport (e.g., by cilia-driven advection mucus) may play a role at slow and fast time scales, as well as surface contact transmission between neighboring cells.
4. The cell internalizes the adhered virus via endocytosis into a vesicle.
5. The endocytosed virion—now residing in a vesicle with lowered pH—is uncoated to release its mRNA contents into the cell cytoplasm.
6. Copying viral RNA creates a (-) RNA template, which is used for (+) RNA production.

7. RNA is used to synthesize viral RNA and proteins.
8. Viral proteins are transported to the interior surface of the cell membrane.
9. Viral proteins at the cell membrane are assembled into virions.
10. Assembled virions are exported from the cell by exocytosis.
11. When a cell dies and lyses, some or all partly assembled and fully assembled virions can be released into the tissue microenvironment.

Once infected, an individual cell cannot "recover" (e.g., by actively degrading viral RNA and stopping endocytosis) to return to normal function. Rather, the cell is irreversibly committed to eventual death (by apoptosis, necroptosis, or pyroptosis[12]). For further detail, see review articles on RNA virus replication dynamics[13,14].

1. What are the critical "choke points" in viral infection, replication, and propagation?
2. Which interventions could most effectively leverage identified vulnerabilities in viral replication?
3. What unanticipated dynamics can emerge from a single molecular-scale inhibition?
4. Does the initial level of exposure to the virus affect the severity of the progression of the disease and how could this effect be ameliorated?
5. What are the key points of virus-immune system interactions that drive mild versus acute (e.g., ARDS) responses?
6. What are key differences at the target cell level during innate versus adaptive immune responses?
7. Are there threshold levels of infection at the cellular or tissue level that indicate a switch from asymptomatic to symptomatic or from mild to severe disease in a patient?
8. Through what mechanisms do certain patient characteristics, pre-existing conditions, or background medications increase the likelihood of adverse outcomes?
9. What interventions could accelerate building immunity?
10. What interventions can reduce or reverse adverse immune reactions?
11. At what stage is a given intervention most beneficial?
12. How does viral mutagenicity affect the robustness of a therapy or a therapeutic protocol?
13. How does cellular heterogeneity affect infection dynamics?
14. How does the nearby tissue environment, such as the mucus layer, affect infection dynamics?
15. How does the infection spread from its initial locus of infection to other tissues (in particular from upper respiratory to lungs)? How does stochasticity impact these dynamics?
16. How do tissues recover after clearance of local infection? Can scarring be minimized to reduce long-term adverse effects in recovered patients?
17. How do adverse effects in SARS-CoV-2 infected epithelia differ (mechanistically) from other infections and other modes of epithelial dysfunction?

**Box 2: Community-selected scientific questions driving the model's development.**

### Infected cell responses

While infected cells (e.g., type 1 or type 2 alveolar cells in the lung) cannot recover, they can respond to slow viral replication and reduce infection of nearby cells. Infected cells do this by secreting type I interferons (IFN-α,β), which diffuse and bind to receptors on nearby cells to slow viral replication, activate an inflammatory response, and induce gene transcription[15], to slow cycling or induce apoptosis in these cells[16]. Secreted IFN-α,β are important activators and regulators of the innate and adaptive immune responses[16]. Many respiratory viruses, including influenza and SARS[17], have evolved to inhibit or delay IFN activation[18], and evidence is emerging that certain non-structural proteins produced by SARS-CoV-2 infected cells interfere with type 1 IFN by inhibiting IFN production and suppressing IFN signaling[17,18].

Eventually, infected cells die (by apoptosis, necroptosis, or pyroptosis[12]), lyse, and release unassembled viral components[12]. While the mechanism of cell death in SARS-CoV-2 is currently unknown, in other RNA virus infections, cells can undergo apoptotic, necrotic, or pyroptotic death over the course of viral infection[19]. Disruption of cell metabolism and competition for critical substrates may also contribute to cell death[20,21].

### Inflammatory and immune responses

Lethal SARS and MERS in humans has been correlated with elevated IFN-α,β [22], myeloid activity, and impaired T and B cells[23,24], with the timing of type 1 IFN critical[25,26]. Type I IFNs secreted by infected cells or by immune cells diffuse to surrounding cells and recruit innate immune cells, such as macrophages and neutrophils, to the area. Recent studies comparing SARS-CoV-2 with SARS-CoV have revealed that SARS-CoV-2 replicates more efficiently in pneumocytes and alveolar macrophages, but that type 1 IFN secretion is blunted in SARS-CoV-2 infected cells[27]. In COVID-19 patients, decreased numbers of T cells, natural killer (NK) cells, and, to a lesser extent, B cells occur, and the extent of T cell depletion has been correlated with disease severity[2,3,28]. Excessive IFN-α,β activation results in increased macrophage and neutrophil presence, which correlates with lung dysfunction[29,30]. Delayed IFN-α,β production also promotes inflammatory macrophage recruitment that contributes to vascular leakage and impaired T- cell function[25,26]. Activated macrophages also produce other proinflammatory cytokines like IL-1, IL-6, and TNF-α, among others, that enhance infiltration of immune cells and interact with endothelial cells to cause vasodilation[31]. The excess production of IL-1 and IL-6 may be related to several viral proteins shown to directly activate the inflammasome pathway, the innate immune response responsible for IL-

1β secretion[32-34]. Moreover, epithelial tissue death can reduce tissue integrity, contributing to further immune infiltration, fluid leakage and edema, and acute respiratory distress[35-37].

In severe cases, a “cytokine storm” of pro-inflammatory cytokines (e.g., IL-2, IL-7, IL-10, G-CSF, IP-10, MCP-1, MIP-1A, and TNF-α) promotes extensive tissue damage[29]. During influenza virus infection, there is some evidence that ARDS is correlated with the extent of infection in the lower respiratory tract and increased cytokine activity resulting from exposure of the endothelium[38]. Other innate responses, such as neutrophils, have also been shown to be amplified in SARS-CoV-2[28]. These cells generally produce reactive oxygen species (ROS), which can induce the death of infected and healthy cells in the local environment[35]. Further, increases in neutrophil counts and the neutrophil-to-lymphocyte ratio (NLR) have been observed in patients with severe COVID-19[39]. The NLR has also been shown to be an important clinical predictor of disease severity[40], as it reflects the innate to adaptive immune response.

Coronaviruses have been shown to evade and modulate various host immune responses[41-43]. In addition to those discussed above, some evidence suggests that an antibody to spike protein enhances disease during SARS-CoV infection by inducing macrophage switching from a wound healing phenotype to an inflammatory phenotype[44]. Furthermore, influenza viruses and SARS-CoV—which also involve ACE2 receptors—are known to infect macrophages and T cells[3,45], raising the possibility for SARS-CoV-2 to similarly infect these cell types. However, it is not yet known whether SARS-CoV-2 infection of these cells is productive[29]. However, the ACE2 receptor has been linked to acute lung injury for both viruses[46,47].

### Inflammation and poor clinical outcomes

While the underlying risk-factors for an individual developing acute respiratory distress syndrome (ARDS) in response to SARS-CoV-2 infection have not yet been elucidated, it appears clear that a dysregulated immune response is central to this aspect of the disease[2,3,28,48]. In particular, chemokines are released following viral infection, which leads to the invasion of neutrophils and macrophages and release of reactive oxygen species (ROS). IL-6 levels have been associated with more severe disease as patients who required ventilation exhibited higher circulating IL-6 levels, as reported by studies from Germany and China[49-51]. In addition, replication in the lower airways and exposure of endothelial cells may further amplify the inflammatory response[38]. Collectively, this leads to extensive tissue damage and depletion of epithelial cells, which may be connected to lethality[52]. Within the alveolar tissue, and systemically, the feedback between viral load, adaptive and innate immune response and tissue damage is clearly a complex system. By utilizing a multi-scale framework to implement these interactions, we aim to connect circulating biomarkers, putative treatments, and clinically observed disease progression to pathophysiological changes at the cell and tissue level.

Coronavirus death is often correlated with pre-existing medical conditions such as diabetes, hypertension, cardiac diseases and obesity[6,53,54]. While the primary effect of SARS-CoV-2 is clearly the lung infection, several secondary effects play a role in the clinical outcome for a given patient. The complex interactions of viral infection, cytokine production, immune response and pre-existing diseases is an active field of current research.

## Anticipated data to drive development and validation

It is important that model development takes into account the types of measurements and biological observations that will be available for later model constraint, calibration, and validation. As participation by the virology and pharmacology communities broadens, we anticipate that this list will grow. While we will endeavor to constrain and validate sub-modules of the model independently, we anticipate human clinical data to not fully determine parameters of the model. To address this concern we will apply a ‘virtual population’ approach and sensitivity analysis to explore model variability within clinically relevant bounds[55,56]. We anticipate the following data:

### Organoid data for viral replication and targeted inhibition

Aarthi Narayanan’s virology lab is optimizing SARS-CoV-2 cultures in organoid model systems. These 3-D model systems infect epithelial cells co-cultured with fibroblasts and endothelial cells and track the viral replication kinetics under control conditions and after treatment by inhibitors. These experiments measure (at various time points) infectious viral titers and genomic copy numbers in supernatants (outside the cells), viral genomic copy numbers in the infected cells¸ host cell death and inflammatory responses, and ATP and mitochondrial disruptions. See Appendix 2 for further detail.

#### Inflammation, ACE2 binding, and host response data

Given the international focus on SARS-CoV-2, we anticipate an unprecedented amount of mechanistic clinical and preclinical data to emerge. Randomized controlled interventional trials in general or specific populations will be of particular value to test and refine the model. As of March 30 2020, there were 119 trials registered at clinicaltrial.gov under the search term "COVID-19+Drug". Within this 119, there are multiple interventions at different points of the pathophysiology, including, but not limited to: broad acting antivirals (e.g., remdesivir), hyperimmune plasma, IL-6 antibody (e.g. Tocilizumab), protease inhibitors (e.g. Lopinavir/ritonavir), chloroquine/hydroxychloroquine, and Janus kinase inhibitors (e.g. Baricitinib). As this platform develops, we anticipate predicting the responses to such therapies and refining the model with emerging data such that the range of clinical responses are captured with adequate fidelity. In addition, data collected from patients or animals during infection, including the presence of various immune cell subsets in lung tissue and systemic markers of inflammation, will serve to differentiate responses to SARS-CoV-2. These data will be similarly integrated to calibrate and validate the model to ensure accurate predictions of therapeutic outcomes based on clinical characteristics.

### Relevant prior modeling

Spurred initially by the emergence of HIV and relevant to the ensuing SARS-CoV-2 pandemic, the field of viral dynamics modelling has been instrumental for understanding the evolution of host-virus interactions[57-65], predicting treatment responses[66-70], and designing novel and more effective therapeutic approaches[71-73]. The classic within-host mathematical model of viral infection uses a system of ordinary differential equations (ODEs) to describe the dynamics between uninfected epithelial ("target") cells, infected cells in the eclipse phase, infected cells producing virus, and infectious virus[74]. This basic model has been shown to capture dynamics of both acute and chronic infection[75], and has been extended to also include multiple viral (potentially resistant) strains[71] and various aspects of host immune responses[76,77]. While such cell population-level models ODE models generally do not account for single-cell effects, they are effective for detailing viral load, host immune response, and pathology dynamics[78-83]. Moreover, they can often be used to constrain and estimate parameters for more detailed models, develop novel hypotheses, and design confirmatory experiments[84,85].

Some have modeled intracellular virus replication, including very detailed models used for understanding replication and intervention points[56,86], typically using systems of ODEs[87,88]. These models often include virus-receptor binding, receptor trafficking, endocytosis, viral uncoating, RNA transcription, protein synthesis, viral assembly, and viral exocytosis. However, to date no such model has been integrated with detailed spatiotemporal models of viral propagation in 3-D tissues with dynamical models of immune interactions.

Recently, agent-based models have been used to simulate viral propagation in 2-D tissues with simplified models of viral replication in individual cells, particularly in the field of influenza virus infection[89] a variety of other viral infections[90], and oncolytic viral therapies[91-94]. These models have generally not included detailed intracellular models of viral replication and individual cell responses to infection. However, they demonstrate the potential for including detailed intracellular models of viral replication in 2D and 3D tissues with the milieu of immune and epithelial cell types expected in actual patients, while also offering the opportunity to test hypotheses on the impact of viral mutagenicity and host cell heterogeneity on disease progression.

The rapid prototyping approach of this coalition will use a performance-driven agent-based modeling platform[95] to combine detailed intracellular models of viral endocytosis, replication, and exocytosis, disruption of cell processes (e.g. metabolism and compromised membranes) that culminate in cell death, inflammation signaling and immune responses, tissue damage, and other key effects outlined above in a comprehensive, open source simulation platform. We will deploy and refine interactive, web-hosted versions of the model to critical contributions by virologists, infectious disease modelers, and other domain experts. We will frequently update preprints to foster the fastest possible scientific dialog to iteratively refine this community resource.

#### Related modeling efforts and other future data sources

We are coordinating with related modeling efforts by a number of groups, such as early pilot work by David Odde and colleagues at the University of Minnesota, and early simulation work in Chaste[96,97] (James Osborne and colleagues), Morpheus[98] (Andreas Deutsch and colleagues), CompuCell3D[99], and Biocellion[100] (Ilya Shmulevich and co-workers). Thomas Hillen has hosted a COVID-19 Physiology Reading Group[101] to exchange information and progress. We are in regular contact with these communities to share data and biological hypotheses and to seek feedback, parameter insights, and data and code contributions.

The COVID-19 Cell Atlas[102] organizes a variety of cell-scale datasets relevant to COVID-19; these may be of particular importance to intracellular modeling components of the project. The Human Biomolecular Atlas Program (HuBMAP)[103] is creating detailed maps of the human respiratory system at cell- and molecular-scale resolution; this will be an excellent data source for tissue geometry in later versions of the model.

## Methods

### PhysiCell: agent-based cell modeling with extracellular coupling

*PhysiCell* is an open source simulation agent-based modeling framework for multicellular systems in 2D and 3D dynamical tissue environments[95]. (See Metzcar et al. (2019) for a general overview of agent-based modeling techniques in tissue-scale biology[104].) In this framework, each cell (of any type) is an off-lattice agent with independent cell cycle progression, death processes, volume changes, and mechanics-driven movement. Each cell agent can have independent data and models attached to it, allowing substantial flexibility in adapting the framework to problems in cancer biology, microbiology, tissue engineering, and other fields. PhysiCell is coupled to *BioFVM* (an open source biological diffusion solver)[105] to simulate the chemical microenvironment. As part of this coupling, each individual agent can secrete or uptake diffusing substrates and track the total amount of material entering and leaving the cell.

Relevant applications of PhysiCell-powered models have included modeling cancer nanotherapy[106], oncolytic virus therapies[107], tissue biomechanical feedbacks during tumor metastatic seeding [108], and cancer immunology[95,109,110]. The platform was built with a focus on computational efficiency and cross-platform compatibility: the same source code can be compiled and run without modification on Linux, OSX, and Windows, and simulations of up to 10 diffusing substrates on 10 $mm^3$ of tissue with $10^4$ to $10^6$ cells are routinely performed on desktop workstations. The platform has been combined with high-throughput computing[109] and active learning techniques[110] to power large-scale model exploration on high performance computing resources.

### Integration of intracellular models in PhysiCell agents

Custom functions can be attached to individual cell agents to model molecular-scale, intracellular processes and couple these with cell phenotypic parameters. These internal models are often implemented as systems of ODEs. For example, cell uptake of diffusing substrates can be coupled with a metabolism model that is defined by a system ODEs, and the resulting energy output can be used to set the cell's cycle progression and necrotic death probability[111]. For small systems of ODEs, these models are coded "by hand" with standard finite difference techniques. More complex models are written in systems biology markup language (SBML)[112] for reliable scientific communication. Development versions of PhysiCell can read and integrate an individual SBML-encoded model in each cell agent using *libRoadrunner*—a highly efficient SBML integrator[113]. Similar approaches have been used to integrate Boolean signaling networks[114] in PhysiCell in the PhysiBoSS extension[115].

These approaches will initially be used to assess (1) viral replication dynamics in each cell agent, (2) cell death responses to viral load, (3) cell responses to interferons, and (4) changes in the virion endocytosis rate based on the availability of ACE2 and its receptor trafficking dynamics.

## Cellular Immunity Agent-Based Model (CIABM)

As an independent model component, co-authors An, Becker, and Cockrell are developing CIABM: an agent-based model of immune system activation and expansion in lymph nodes in response to SARS-CoV-2 infections. This model will be coupled with the overall simulator to mechanistically drive immune expansion and infiltration during inflammatory responses.

The CIABM is intended to be a generalizable model of CD8 dynamics, designed to represent different disease states resulting from different perturbations (i.e. specific infections of specific pathogens, putative vaccines and their administration strategy). This is consistent with our philosophy of pathophysiological unification through modeling. We have developed multiple ABMs related to the immune response and diseases related to inflammation and immune dysfunction[116,117], and will leverage this experience to integrate various aspects of these models as components of the CIABM. Many of these models are based on the IIRABM[116], which is an abstract representation and simulation of the human inflammatory signaling network response to injury; the model has been calibrated such that it reproduces the general clinical trajectories seen in sepsis. The IIRABM operates by simulating multiple cell types and their interactions, including endothelial cells, macrophages, neutrophils, TH0, TH1, and TH2 cells and their associated precursor cells. The simulated system dies when total damage (defined as aggregate endothelial cell damage) exceeds 80%; this threshold represents the ability of current medical technologies to keep patients alive (i.e., through organ support machines) in conditions that previously would have been lethal. The IIRABM will be used in the CIABM to represent the innate and host tissue component of the CIABM.

The design principles of the CIABM are: 1) CD8-dynamics focused detail, 2) incorporation of the IIRABM as the innate-host tissue module, 3) incorporation of humoral and CD4 T-cell function, but at an abstracted level. The justification for aggregating humoral and CD4 functions is that they are primarily governed by interactions through the MHC2 complex, and therefore represent a distinct sensing/recognition capacity than CD8 MHC1-mediated recognition. While we recognize that CD4-helper activity is known to assist the generation of CD8s, at the outset of this project we will attempt to see how well we can reproduce our clinically-acquired data without having to explicitly represent CD-4-helper function. If we are unable to satisfactorily map CIABM behavior to our experimental/clinical data, we will then add these components.

As a general description, the initial components of the innate immune response represent the end-effector of the system, primarily responsible for interactions influencing tissue damage, microbial killing and abstracted tissue reconstitution. This component incorporates both pro-and anti-inflammatory components, consistent with a self-contained control structure befitting its role as a highly-evolutionarily conserved, fundamental function of multicellular organisms. These agent types are: tissue, viral antigen, polymorphonuclear neutrophil cells (PMNs), macrophages, dendritic cells, naïve and cytotoxic CD8 T-cells, and regulatory T-cells.

## HPC-driven model exploration and parameterization

The concurrent growth and advancements in the three areas of 1) mechanistic simulation modeling, 2) advanced, AI-driven model exploration algorithms, and 3) high-performance computing (HPC) provides the opportunity for large-scale exploration of the complex design spaces in detailed dynamical simulation models. However, if we don't take deliberate efforts to formally facilitate this intersection across our research communities, we risk producing a series of disparate individual efforts, limited in interoperability, transparency, reproducibility and scalability. The EMEWS (extreme model exploration with Swift) framework[118] was developed to directly address this issue and to provide a broadly applicable cyberinfrastructure to lower the barriers for utilization of advanced, large-scale model exploration on HPC resources. The EMEWS paradigm allows for the direct exploitation of cutting edge statistical and machine learning algorithms that make up the vibrant ecosystem of free and open source libraries that are continually added to and updated as research frontiers are expanded, all while controlling simulation workflows that can be run anywhere from desktops to campus clusters and to the largest HPC resources.

We have utilized EMEWS for learning-accelerated exploration of the parameter spaces of agent-based models of immunosurveillance against heterogeneous tumors[109,110]. The approach allowed for iterative and efficient discovery of optimal control and regression regions within biological and clinical constraints of the multi-scale biological systems. We have applied EMEWS across multiple science domains[119-122] and developed large-scale algorithms to improve parameter estimation through approximate Bayesian computation (ABC) approaches[123]. These approaches, applied to the multi-scale modeling of SARS-CoV-2 dynamics, will provide the ability to robustly characterize model behaviors and produce improved capabilities for their interpretation.

### nanoHUB platform

The nanoHUB platform (nanohub.org)[124] is a free, cloud-based service offering lectures, tutorials, and, of particular interest to us, interactive Web-based simulation tools. As its name implies, it is primarily focused on nanoscale science education and research. To make their simulation tools easier to use, nanoHUB provides a custom toolkit for developing graphical user interfaces (GUIs). However, since 2017, they have adopted and promoted the use of Jupyter notebooks[125], with accompanying Python modules to provide GUI widgets and visualization. Cloud-based computing and data analysis platforms are well established now, in both academic and commercial settings. For those who provide easy-to-use Web-based GUIs and APIs, and offer affordable pricing, it is likely their rate of adoption will continue to increase, especially among researchers who may lack the expertise or resources to install complex pieces of software.

### xml2jupyter and cloud deployment of PhysiCell models

Compiled PhysiCell models generate executable software that runs at the command line. Model parameters are set by editing XML (extensible markup language) configuration files, and the models save data as a combination of vector graphics outputs (scalable vector graphics: SVG) and XML and Matlab files based on the draft MultiCellDS data standard[126].

To facilitate rapid cloud-hosted dissemination of PhysiCell-powered models on the nanoHUB platform, we developed *xml2jupyter* to automatically generate a Jupyter-based graphical user interface (GUI) for any PhysiCell model[127]. The Jupyter notebook includes widgets to set parameters, initiate a simulation run, and visualize diffusing substrates and cell agents. In turn, we also developed a protocol to deploy the PhysiCell model and the Jupyter notebook interface on nanoHUB as a cloud-hosted, interactive model. This allows developers to rapidly convert a locally executable, command-line model to a cloud-hosted shared model with graphical interface in a matter of minutes to hours (depending upon testing speed on nanoHUB).

In our rapid prototyping, we use rapidly-generated nanoHUB apps for scientific communication across disciplines: virologists, pharmacologists, and other domain experts can explore and visualize the model prototypes without need to download, compile, and understand the code. This facilitates faster multidisciplinary dialog, and helps to draw in broader community feedback and contributions.

### Modular design

The model is being evolved with a modular architecture. The overall model and each individual model component (submodel) have a separate GitHub software repository in the pc4covid19 GitHub organization, available at https://github.org/pc4covid19.

Each module's repository consists of a *master* branch (which will always match the latest numbered release) and a *development* branch. Contributors will fork the development branch, complete their milestones, and submit a pull request to incorporate their progress in the development branch. Whenever the submodel team is ready to make a numbered release, they will use a pull request from the development branch to the master branch and create a numbered release.

The overall model framework and each submodel will keep a versioned design document to include:

- A unique name for the model component
- A clear version number and last update timestamp
- A list of contributors, including 1-2 chief scientists who serve as primary points of contact
- A "plain English" description of the primary purpose of the component

- A statement of model inputs with units of measure.
- A clear statement of the biological hypotheses and assumptions of the component
- A record of the current mathematical form of the model (generally maintained in a separate Overleaf LaTeX document), with a snapshot of the Equations in the main design document
- Any computational implementation details needed to understand the code
- A link to a GitHub repository
- A list of model parameters, units, biophysical meaning, best estimate, and data source(s) for the parameter estimate. (See the discussion in MultiCellDS[126].)
- A clear list of model outputs with units
- A set of qualitative and/or quantitative **unit** tests to ensure proper functionality of the module.

A snapshot of this design document will be included in each release of the (sub)model.

The overall model releases will include a clear list of the version of each submodel included in its release.

## Coalition structure

After group discussion and prioritization, coalition members self-assigned themselves to one or more subteams responsible for developing the submodels. Each **subteam** has 1-2 **chief scientists** in charge of managing development and approving pull requests from the subteam's contributors. The submodel chief scientist(s) meet regularly with their team to assign tasks, set milestones, and assess when to make a release. The submodel chief scientist(s) also coordinate their progress with the other submodel teams.

The **integration** team—consisting of the **overall leads** (as of April 1, 2020: Paul Macklin, Randy Heiland, and Yafei Wang) and other contributors—is responsible for developing and maintaining the overall integrated model, integrating and testing updated submodels, and providing technical support to the subteams.

The ***core team*** consists of the **overall leads** (as of April 1, 2020, this is) and the **chief scientists**. They meet to coordinate progress of the submodels, refine project scope, exchange ideas on model hypotheses, evaluate community feedback, and plan overall strategy. They cooperate with the overall leads to create model releases (which will always bundle the most stable version of each submodel), update the nanoHUB models, and update the *bioRxiv* preprint.

## Three main phases of community-driven development

### Phase 1: Building the coalition and model infrastructure

In the first phase, the overall and integration leads (Macklin, Heiland, and Wang) build the overall tissue model structure (an overall model that integrates several independent *submodels*), and create "placeholder" models that serve as working proof-of-concept starting points for further expansion. This phase also builds and organizes the subteams responsible for the submodels and provides them with training and documentation on the model and submodel architecture.

We anticipate that Phase 1 will require six-to-eight weeks.

### Phase 2: Community-driven development

In this phase, integration team transitions primary development of each of the submodels to appropriate domain experts in the subteams, to ensure that each submodel reflects the best available science. During this phase, the integration team supports each subteam in mathematical model development, PhysiCell implementation, and nanoHUB deployment for rapid subteam testing, dissemination, and community feedback on the submodels.

The integration team continues to lead overall model integration, testing, and deployment as a cloud-hosted model, as well as development of further infrastructure (e.g., HPC investigations) and PhysiCell and xml2jupyter refinements needed by the subteams (e.g., full support for systems biology markup language (SBML) for molecular-scale model integration).

Once the integrated model can qualitatively produce expected viral and immune behaviors (as determined by the core group) and receives no major domain expert or community critiques, the major goal of the coalition (and this paper) will be met: to create a SARS-CoV-2 modeling framework suitable for subsequent calibration, validation, and exploration by the community. It will be submitted to scientific peer review, disseminated to the community, and maintained. This will mark the conclusion of Phase 2.

We anticipate that Phase 2 will require three-to-nine months.

#### Phase 3: widespread scientific use and model maintenance

Once the overall model and submodels are largely complete, the model will be a mature, open source community resource available for use in scientific investigations. (Moreover, due to our iterative approach, we envision that teams will have begun using earlier versions of the model for investigations by this point.) The integration team will transition to supporting parallel investigations by independent groups using the models, and aggregating and sharing best data, parameter estimation, and results. The integration team and subteams will coordinate to encourage full scientific publication of the overall model and the submodels, as well as resulting scientific investigations.

This phase will also focus on code hardening, documentation, and development of training and outreach materials. This phase is critical for *knowledge capture*, so that the model remains usable beyond the involvement of the original teams and can be rapidly adapted to emerging health challenges in the future. We also envision continued refinement of the model to reflect improved biological knowledge.

### Iterative development

We use ***rapid prototyping***, using lessons learned from each step to drive iteration towards improving the model. Each submodel undergoes its own two-week development sprints, within a broader three-to-four week development cycle for the overall integrated model. See **Figure 1**.

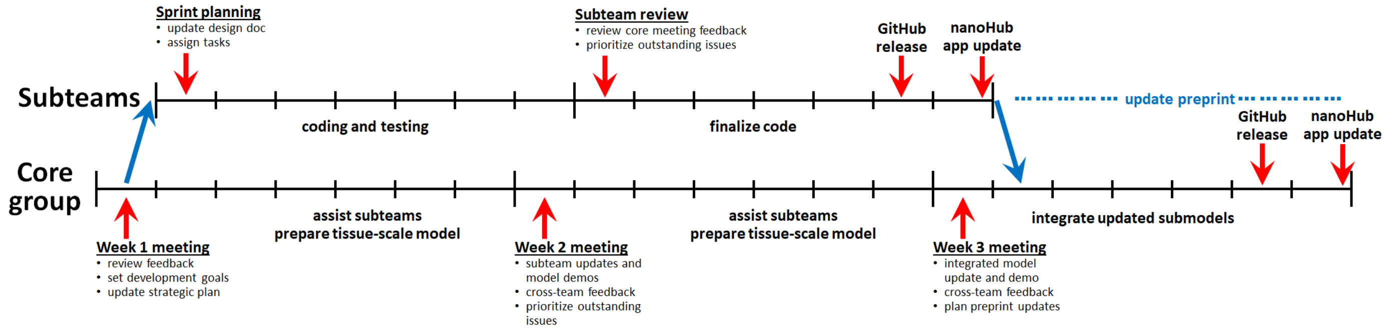

**Figure 1: Overall development cycle:** Throughout the overall 3-week development cycle, the **core team** (integration leads + subteam leads) set priorities and coordinate work of the subteams. Each **subteam** performs a 2-week sprint to update its submodel. At the end of the 3-week cycle, the **integration team** bundles the most up-to-date submodels for the next overall model release, while the subteams update the preprint and refine domain knowledge and mathematics for the next sprint.

#### Overall integrated model design cycle

We currently use a 3-week design cycle for the overall integrated model.

##### Week 1: start of cycle

The design cycle starts with an initial **core team meeting** where we discuss feedback from prior design cycles and set priorities for the current design cycle. In particular, we discuss:

- What changes are needed to the submodels? Prioritize changes can be made within a 7-10 day sprint.
- What changes are needed in the overall integrative framework to facilitate the improved submodels? Set framework goals for Week 1 and Week 2.
- Are any funding, personnel, scope, other changes needed?

Within the working week, the **subteams** meet to further set and accomplish their sprint goals. (See **Submodel design cycle**). The **integration team** (1) works on refinements to the PhysiCell and nanoHUB frameworks to facilitate subteam work, (2) provides technical consulting to the subteams to implement their model refinements, and (3) makes any final edits needed to the preprint from the last design cycle.

#### Week 2: mid-cycle advances

The design cycle continues with a **core team meeting** to discuss the current subteam model sprints:

- Each team gives a brief report on their model advances and a live demo of either the standalone C++ code or a nanoHUB submodel app.
- The teams "cross-pollinate" to exchange ideas and give feedback on each of the submodels
- The core team decides on any additional changes needed to continue the design cycle.
- The integration team and subteam chief scientists set final deadlines to end the sprints and release the updated submodels.

Within the working week, the **subteams** continue and complete their developing and testing for their respective sprints, create new submodel releases on GitHub, and update their submodel nanoHUB aps. The **integration team** continues support for the subteam work and completes any changes to the overall integrative model needed for the upcoming integration.

As the subteams advance towards their releases, the **integration team** updates and tests the respective sub-models in the overall framework and updates the overall nanoHUB app.

#### Week 3: cycle end

The design cycle nears completion with a **core team meeting** to discuss integration and preprinting:

- The **integration team** gives an update on current integration testing.
- The teams coordinate on any remaining submodel releases.
- The teams set plans for updating the preprint.

Within the working week, the **subteams** complete their releases (if not already complete in week 2) and begin revising the preprint. They also begin testing the integrated model as it comes online to integrate new simulation results and insights into the preprint.

The **integration team** updates the submodels, performs final testing, and creates both GitHub and nanoHUB releases. Once complete, it joins the subteams on preprint updates.

### Submodel design cycle

Each submodel is developed in parallel with a unified design cycle (a 7-14 day software *sprint*), in coordination with the other teams during the weekly core team meetings and via a dedicated slack workspace.

#### Week 1: start of sprint

The sprint cycle starts with an initial **subteam meeting** to communicate the results and priorities of the core team meeting to the subteam. The team edits the submodel design document, discusses any necessary changes to the mathematics and parameter values, and assigns implementation tasks. The team coordinates with the integration team via the slack workspace for any needed assistance on model implementation.

#### Week 2: end of sprint

The design cycle continues with a **core team meeting** to discuss the current subteam model sprints:

- Each team gives a brief report on their model advances and a live demo of either the standalone C++ code or a nanoHUB submodel app.
- The teams "cross-pollinate" to exchange ideas and give feedback on each of the submodels
- The core team decides on any additional changes needed to continue the design cycle.

- The integration team and subteam chief scientists set final deadlines to end the sprints and release the updated submodels.

Within the working week, the **subteams** continue and complete their developing and testing for their respective sprints, create new submodel releases on GitHub, and update their submodel nanoHUB aps. The **integration team** continues support for the subteam work and completes any changes to the overall integrative model needed for the upcoming integration.

As the subteams advance towards their releases, the **integration team** updates and tests the respective submodels in the overall framework and updates the overall nanoHUB app.

See **Appendix 4: Submodel development details** for more implementation details.

## Results

### Version 1 (March 25-March 31, 2020)

Version 1 was designed as proof of concept rapid prototype to capture essential (but highly simplified) elements of viral endocytosis, protein synthesis, viral assembly, release, and diffusion to infect other cells. The model was tailored to RNA viruses on a tissue monolayer (modeled as a layer of epithelium over a basement membrane). This version was kept deliberately simple to create an early starting framework to help coalesce community feedback and contributions. It was also designed to test the use of interactive cloud-hosted models to help accelerate feedback by virologists and other domain experts through live demos.

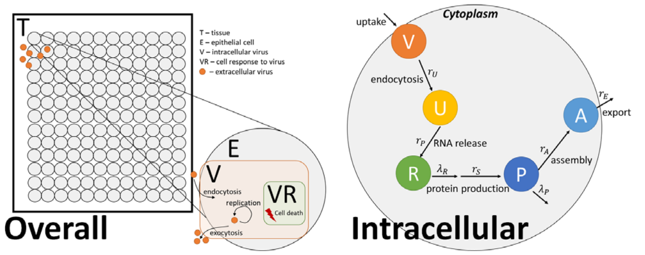

**Figure 2: Version 1 model schematic:** ***Left:*** In the overall model structure, a ***tissue component*** **(T)** contains multiple ***epithelial cells*** **(E)**. Viral particles diffuse through this domain and can enter the cells (by adhering as an uptake term) or be exported by cells. Each cell includes an intracellular ***virus*** model **(V)** for viral replication kinetics and a ***viral response*** model **(VR)** which uses a pharmacodynamic model to control cell apoptotic response to viral load. ***Right:*** Each epithelial cell individually models its viral kinetics: adhered virions complete endocytosis, are uncoated, functionalize their RNA, synthesize viral components, and assemble them into virions that are subsequently exported to the external tissue compartment (T).

The proof of concept model was created by the overall leads (Macklin, Heiland, Wang) while assembling the modeling coalition as an initial starting point and feasibility test for rapid prototyping. Feedback on this version drove the formulation of the design protocols reported above.

#### Submodels

The Version 1 model includes the following submodel components:

- **T:** tissue (which contains epithelial and other cells)
- **V:** viral endocytosis, replication, and exocytosis responses
- **VR:** cell response to viral replication, including cell death and IFN synthesis
- **E:** epithelial cell (incorporates V and VR).

The overall model components are summarized in **Figure 2**.

#### Biological hypotheses

In this proof of concept prototype, we modeled a simplified set of biological hypotheses:

1.T.1 Virus diffuses in the microenvironment with low diffusion coefficient
1.T.2 Virus adhesion to a cell stops its diffusion (acts as an uptake term)

1.V.1 Adhered virus undergoes endocytosis and then becomes uncoated
1.V.2 Uncoated virus (viral contents) lead to release of functioning RNA
1.V.3 RNA creates protein forever, unless it degrades

1.V.4 Protein is transformed to an assembled virus state
1.V.5 Assembled virus is released by the cell

1.VR.1 As a proxy for viral disruption of the cell, the probability of cell death increases with the total number of assembled virions
1.VR.2 Apoptosed cells lyse and release some or all of their contents

(In the above, X.C.Y denotes prototype X, modeling component C, biological hypothesis Y, allowing us to easily refer to any individual hypothesis or assumption in discussion and community feedback.) In the next version of this model, we will use the design document protocols for each of these components.

#### Unit tests

The first prototype should demonstrate the following behaviors for a single cell infected by a single virion:

- The virion progresses to the uncoated state.
- The uncoated virion progresses to the RNA state.
- With export and death off, RNA produces protein.
- With export and death turned off, protein produces and accumulates assembled virus (linearly).
- With export off and death on, cell undergoes apoptosis with increasing likelihood as assembled virus accumulates.
- With export on and death on, surrounding cells get infected and create virion.
- Cells nearest the initial cell are infected first.
- Apoptosis is most frequent nearest to the initial infected cell.

### Translation to mathematics, rules and model components

#### Extracellular virion transport (Tissue submodel ***T***)

To rapidly implement extracellular viral transport using existing model capabilities, we approximated the process as diffusion with a small diffusion coefficient as in prior nanoparticle models. Using the standard BioFVM formulation[105], if $\rho$ is the concentration of virions (virions / $\mu m^3$), then it is modeled as:

$$\frac{\partial \rho}{\partial t} = D\nabla^2\rho - \lambda\rho + \sum_{\text{cells } i} \delta(\mathbf{x} - \mathbf{x}_i)(-U_i V_i \rho + E_i),$$

where $D$ is the diffusion coefficient, $\lambda$ is the net decay rate (which can include other removal processes), U is the uptake rate (by adhering to ACE2 and initiating endocytosis), and E is the cell's virion export rate. (Here, delta is the Dirac delta function, $V_i$ is the cell's volume, and $\mathbf{x}_i$ is position of the cell's center.) Note that in the default BioFVM implementation, uptake processes are spread across the cell's volume.

Note that virus propagation may require more explicit modeling of cell-cell surface contact in later versions, as well as cilia-driven advective transport and virion deposition (e.g., through airway transport).

#### Intracellular viral replication dynamics (Virus intracellular model ***V***)

Within each cell, we track $V$ (adhered virions in the process of endocytosis), $U$ (uncoated viral RNA and proteins), R (viral RNA ready for protein synthesis; R = 1 denotes one virion's total mRNA), P (synthesized viral proteins; P = 1 denotes sufficient viral protein to assemble a complete virion), and A (total assembled virions ready for exocytosis). Virion import (a source term for V) is handled automatically by the mass conservation terms for PhysiCell in the PDE solutions.

We model these dynamics of internalized virions through a simplified system of ODEs:

$$\frac{dV}{dt} = -r_U V$$

$$\frac{dU}{dt} = r_U V - r_P U$$

$$\frac{dR}{dt} = r_P U - \lambda_R R$$

$$\frac{dP}{dt} = r_S R - r_A P - \lambda_P P$$

$$\frac{dA}{dt} = r_A P$$

We model exocytosis by setting the export rate E of the assembled virions, in units of virions per time:

$$E = r_E A$$

#### Cell response (Viral response submodel ***VR***)

In this proof of concept prototype, we modeled apoptotic response to cell disruption but did not model interferon processes. As a simplification, we modeled cell disruption as correlated with assembled virions *A*, and we used a Hill pharmacodynamic model to relate the cell's apoptosis rate to *A*:

$$e = \frac{A^n}{A_H^n + A^n} \ ,$$

where *e* is the effect, *n* is the Hill coefficient, and $A_H$ is the amount of virions at which half-max effect is achieved. We then set the apoptotic death rate at

$$r_{\text{death}} = r_{\max} e \ ,$$

where *e* is the maximum apoptosis rate (at full effect, *e* = 1). As analyzed for agent-based models with stochastic death rates[95,128], in any time interval [$t$, $t+\Delta t$], the cell has probability $r_{\text{death}}\Delta t$ of apoptosing, and the mean cell survival time (for fixed *e* and thus fixed $r_{\text{death}}$) is $1/r_{\text{death}}$.

In PhysiCell, we can set the lysing cells to release any fraction ($0 \le f_{\text{release}} \le 1$) of *V*, *A*, *U*, *R*, and *P* into the extracellular environment as diffusing substrates.

### Other implementation notes

To differentiate between incoming imported and exported virions, we actually modeled two diffusing fields (for extracellular concentrations of *V* and $\underline{A}$). At the end of each computational step, we manually move all of the exported assembled virions in each voxel into the concentration of diffusing virions.

We also created diffusing fields for uncoated virions, RNA, and viral proteins, for use in future models where these may be immunogenic or linked to measureable data.

### Software release

The core model associated with the v1 prototype is Version 0.1.3. The nanoHUB app associated with the v1 prototype is Version 1.0. GitHub releases and Zenodo snapshots are given in the appendix.

### Cloud-hosted model

We rapidly created and deployed a cloud-hosted model with an interactive Web-based graphical user interface (GUI) running on nanoHUB (nanohub.org) using xml2jupyter Version 1.1[127]. The web-hosted model can be run at https://nanohub.org/tools/pc4covid19.

This workflow uses a Python script that converts a PhysiCell configuration file (in XML) into a Jupyter notebook and adds additional Python modules for the GUI. The automated process of converting a standalone PhysiCell model into an interactive Jupyter notebook version (a GUI) takes just a few minutes. The resulting GitHub repository is shared with the nanoHUB system administrators who install it for testing as an online, executable

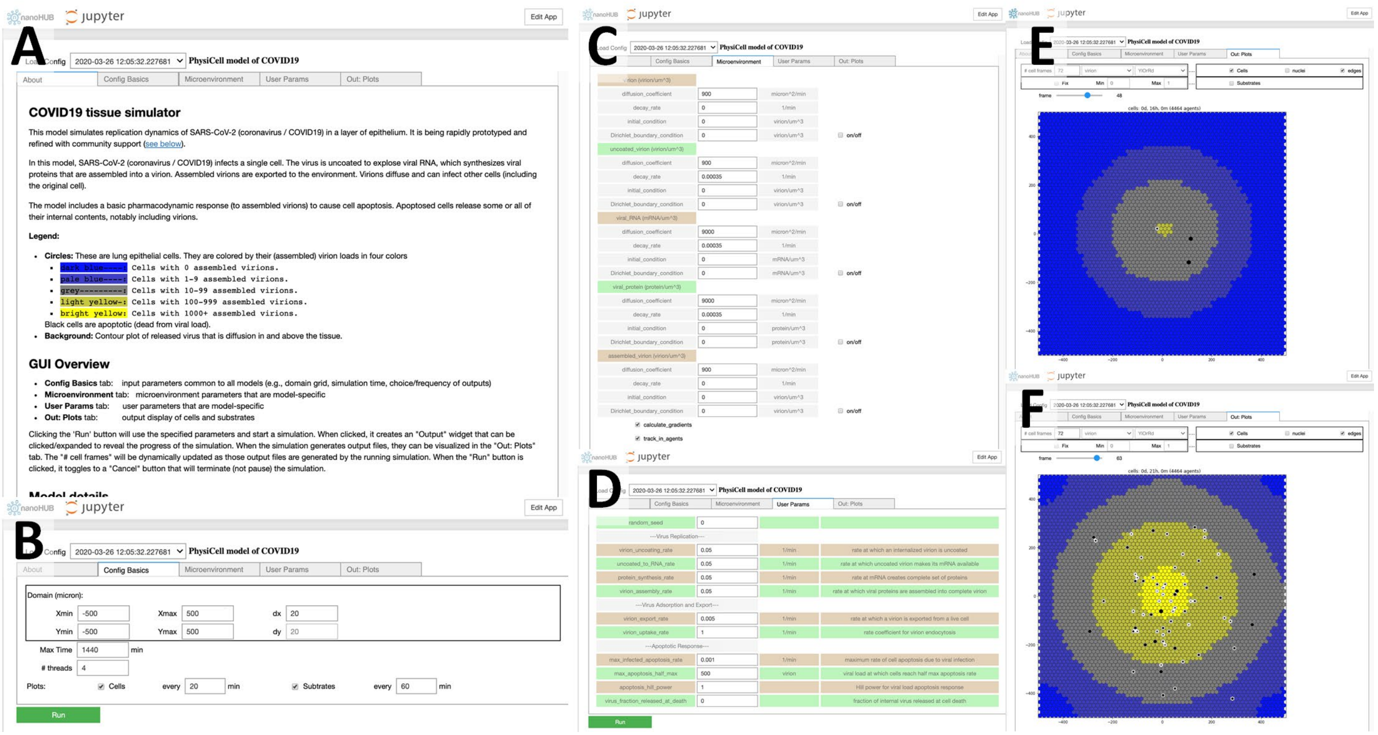

**Figure 3: Version 1 on nanoHUB: A.** The *About* tab includes background information on the model, run directions, and a legend. **B.** The *Config basics* tab allows users to set the domain size. (These should be left alone in this version.) **C.** The *Microenvironment* tab lets users set diffusion and decay coefficients, as well as initial and boundary conditions. The track_in_agents option must be enabled for the model to work correctly. **D.** The *User Params* tab sets key model parameters. **E-F.** Sample outputs in the *Out: Plots* tab. Users can scroll the "frame" tab to advance the simulation time, and choose whether to plot the cells, a diffusing substrate, or both. In these plots, we are looking at a sheet of epithelium (circles are individual cells). Brighter yellow cells contain more assembled virion. Black cells are apoptotic, and clear spaces show tissue damage (where cells have apoptosed and been removed.) This version of the nanoHUB model was released at DOI 10.21981/19BB-HM69.

model (an "app"). After we perform usability and other testing and finalize documentation, it is published and becomes available for public use. The whole process (including the initial development of the core PhysiCell model) took less than 12 hours for this particular app. See **Figure 3.**

### Model behavior: what does the current version teach us?

Except as noted below, all simulation results use the v1 model default parameters, which are supplied in the XML configuration parameter file of the version 0.1.2 core model repository.

In all plots, dark blue cells have 0 assembled virus, pale blue cells have 1-9 assembled virions, grey cells have 10-99 assembled virions, light yellow cells have 100-999 assembled virions, and bright yellow cells contain 1000 or more assembled virions. Black cells are apoptotic, and white spaces show regions devoid of cells (extensive tissue damage). See the legend in **Figure 4** (A).

#### Behavior with default parameters

Running the overall model (with virus release turned on and off as appropriate for the respective unit tests) shows that the v1 prototype satisfies all the qualitative unit tests: a single cell is infected with a virion in the center of the tissue. Over time, the virion is uncoated to create functionalized RNA, which is synthesized to viral proteins and assembled to functional virus. The graphical output shows this center cell turning to a bright yellow as assembled virions accumulate. By enabling the substrate plot, we can see the diffusive field of virions first has zero concentration (no virions have been released), but as the first cell's viral production increases, it releases virus particles that begin diffusing into the domain. See **Figure 4** (A).

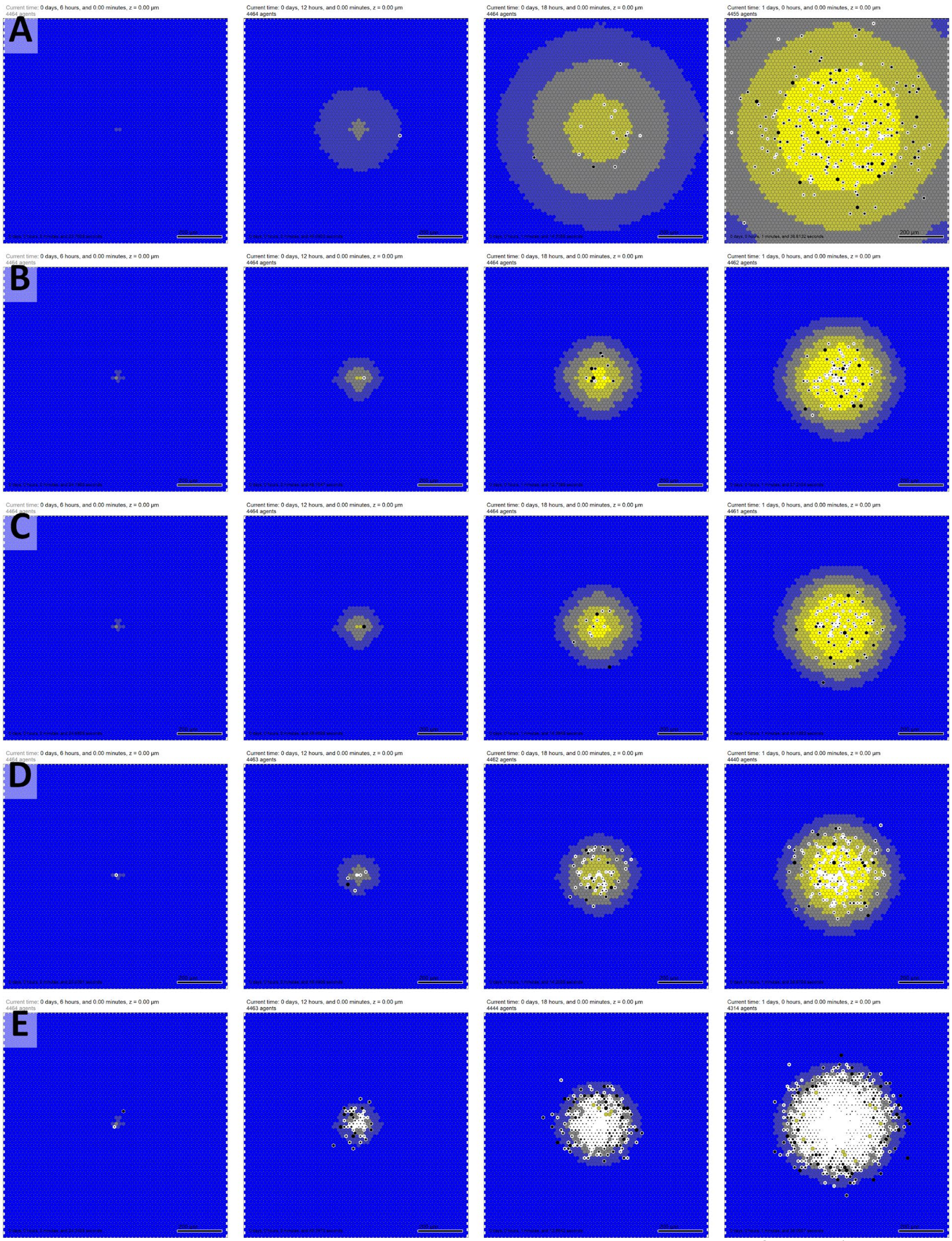

**Figure 4: Version 1 sample model results at 6, 12, 18, and 24 hours.** In all plots, epithelial cells are colored from blue (no assembled virions) to bright yellow (1000 or more virions). Black cells are apoptotic, and white regions show damaged tissues where apoptotic cells have degraded to expose (unmodeled) basement membrane. Bar: 200 $\mu$m. **A.** Simulation time course for the default parameters. Note the spread of the infection from an initial infected cell at the center, with apoptotic death events focused near the center. **B.** Decreasing the diffusion coefficient of virions by a factor of 10 drastically reduces the rate of spread, although focusing exocytosed virions in a smaller diffusion distance increases the number of virions infecting nearby cells, leading to faster apoptosis. **C.** Allowing apoptosed cells to release their assembled virions at lysis had a negligible effect for these parameters, given the dominant effects of releasing virions throughout the cells' survival times. **D.** Decreasing the cell's tolerance (half max) of assembled virions prior to apoptosis accelerates tissue damage but does not drastically accelerate the spread of the infection. **E.** Increasing the apoptosis rate (or decreasing the survival time) for infected cells drastically increases tissue degradation.

Over time, neighboring cells also become infected. They, too progress towards a higher viral load (increasingly bright shades of yellow). The infection propagates outward from the initially infected cell into the remaining tissue. As each cell's viral load (here measured as number of assembled virions) increases, the viral response model calculates the increasing effect *e*, and cells have greater probability of apoptosis. Cells nearest to the initial site of infection apoptose earliest. As these cells degrade, they are removed from the simulation, leading to the creation of a degraded, cell-free region near the center of the tissue. This degraded region spreads outwards from the initial site of infection over time.

See **Figure 4** (A) for a simulation with default parameters. The nanoHUB distribution of this model takes approximately 60-90 seconds to execute.

#### Impact of the virion diffusion coefficient

We next tested the effect of the viral diffusion coefficient by reducing it from 900 $\mu m^2$/min to 90 $\mu m^2$/min. Because the viral particles spread less distance after their release, they reach other cells more slowly, and the overall spread of the infection is slowed. See **Figure 4** (B).

We left $D$ = 90 $\mu m^2$/min for all subsequent investigations of the v1 model.

#### Impact of the viral release at cell death

We next tested the effect of releasing all assembled viral particles at the time of cell death by setting $f_{release}$ = 1. For this set of model parameters, the release of assembled virions had a negligent impact of the overall spread of infection: Compare the final frame of row B (no release: $f_{release}$ = 0) to row C (complete release: $f_{release}$ = 1) in **Figure 4**. This is because cells release far more virions during their infected lifetimes, so the effect is dominant over the one-time release of virions at cell death. We expect this behavior would change if the cells exocytosed virions more slowly.

#### Impact of the cell tolerance to viral load

We next decreased the cell tolerance to viral load by decreasing the pharmacodynamic half max $A_H$ from 500 virions to 10, while leaving $f_{release}$ = 1. As expected, cell death and tissue damage occurred much more quickly under these parameters. Interestingly (and contrary to intuition), this did not significantly alter the rate at which the infection spread through the tissue. Compare the final frame of row C (higher tolerance to viral load) to row D (lower tolerance to viral load) in **Figure 4**. This shows the importance of creating spatiotemporal models of viral replication in tissues, as the balance of competing processes can lead to unexpected dynamics at the tissue, organ, and organism levels.

#### Impact of the cell survival time under high viral loads

We next decreased the cell tolerance to viral load further by decreasing the mean cell survival time under high viral loads, which is equivalent to increasing the maximum apoptosis rate $r_{max}$. (Following prior analyses[95,128], Recall that $1/r_{max}$ is the mean expected survival time as $A \to \infty$.) We increased rmax from 0.001 $min^{-1}$ (1000 minute expected lifetime at high loads) to 0.01 $min^{-1}$ (100 minute expected lifetime at high viral loads).

This drastically accelerated the rate of tissue damage, leaving much more basement membrane (the assumed surface under the epithelial monolayer) exposed. In a later version of this model framework, we would expect this to lead to earlier onset of fluid leakage, edema, and ultimately adverse respiratory outcomes such as ARDS. Interestingly, this did not significantly increase the rate of spread of the infection. Compare the final frame of row D (higher tolerance to viral load) to row E (lower tolerance to viral load) in **Figure 4**.

### Key feedback from domain experts and the community

We gathered feedback from the multidisciplinary community, several of whom joined the coalition for future work. We summarize the feedback below.

**Aarthi Narayanan (virology):** More detail on endocytosis, viral uncoating, and synthesis would expose more actionable points in the replication cycle. Preliminary SARS-CoV-2 experiments in her laboratory suggest that the time course (and thus general order of magnitude of rate parameters) is very similar to Venezuelen equine encephalitis virus (VEEV) dynamics measured earlier[20,21]. The exponential progression matches observations: the first cell is infected with one virion and so at first produces virus slowly, but neighboring cells can be infected with multiple virions and thus create virus particles more quickly.

**Simon Parkinson** identified typographical errors in the original documentation, but verified that that mathematics in the C++ implementation were not affected. He emphasized the importance of implementing RNA decay (as a rate limiting step in virus replication) and the importance of integrating ACE2 receptor trafficking (as a rate limiting step in virus adhesion and endocytosis).

**Paul Macklin (multicellular systems biology, open source frameworks)** noted the potential to simplify the model by removing the diffusing $U$, $R$, and $P$ fields, and reported bugs in the initialization (where no cells are initially infected for some domain sizes, due to hard-coding of the initial seeding).

**Morgan Craig and Adrianne Jenner (mathematical biology, viral dynamics)** emphasized the importance of varying virion "uptake" with ACE2 receptor availability, and hence the need to integrate receptor trafficking.

**Amber Smith (mathematical biology and infectious diseases)** noted her prior work on other respiratory viruses will help estimate parameters and build initial immunologic regulation models. Lung pathology and disease severity are closely tied to the immunologic reaction, and prior data and images from influenza will help with calibrating spatial considerations. She noted that she expects animal and drug data available for SARS-CoV-2 in the coming months. She noted the importance of distinguishing between mild and severe infections and ARDS. Matching the output to data will be imperative, with one quick possibility to make this match data and distinguish between possibilities is to plot the resulting viral load.

She suggested that it would be helpful to show multi focal points of initial infection seeding (possibly of different initial seeding size) that merge together over time, which would match observations of lung histology. Future work will have a better impact if they use a true lung tissue geometry with immune cells limiting the peripheral spread. The current model seems more relevant to in vitro growth of a single plaque, which may be scrutinized.

**Richard Allen (quantitative systems pharmacology, Pfizer, Inc.)** pointed out the need for clearer scoping and diagrams to clearly lay out the design of each submodel component. We will need procedures to choose future incorporations and changes of scope. He also pointed out the need to understand what happens if you bind up a lot of ACE2 with receptor; there are early insights online[129].

**Ashlee N. Ford Versypt (mathematical biology, bioengineering, inflammation and tissue damage)** noted that the diffusion coefficient of 900 $\mu m^2/min$ = 15 $\mu m^2/s$ = 1.5e-11 $m^2/s$ is not particularly small; prior analyses[130] considered virion diffusion in an lung epithelial monolayer for influenza with D = 3.18e-15$m^2$ estimating from experimental data. The virions for SARS-CoV-2 could be more mobile though-it's uncertain. There are data about the diffusion coefficient for albumin in tissue being on the order of 10-50 $\mu m^2/s$; see this reference[131]. She stated that it makes sense for a virion to move more slowly than a protein with radius < 5 nm unless "diffusive transport" is encompassing an active or facilitated transport mode beyond just classic diffusion. She also noted that her laboratory has looked a lot at the renin-angiotensin-system systemically and in kidneys: the kinetics of AngII, ACE, and ACE2 in the lungs would be of interest for connecting the next iteration of the ACE2 receptor model to connect to ARDS. Pfizer may also have relevant related models.

**Courtney L. Davis (mathematical biology, infectious diseases and ecology)** noted that the model could study immune responses and the impact of mucosal structure in future versions. She suggested quantifying damage or disease metrics. She also noted that ultimately it would be useful to note which parameter estimates might be species-specific and which are not, to be able to switch between experimental and clinical systems. (e.g., it is worth recording if current estimates are from human, macaque, etc.)

She also noted that it may be important to determine if apoptotic cells are replaced or if there is permanent damage (in the model). If the model is run longer, it would be worthwhile to translate the visual sense of damage to a quantitative metric.

**Chase Cockrell and Gary An** noted their work on modeling immune expansion in "off screen" lymph nodes, and offered to link their model to our immune infiltration functions.

**James Glazier** noted the need for clearly specifying each model's assumptions, inputs and outputs, to drive robust parallel development. He noted that it is critical to consider information flow between submodels and revise these data flows as the iterations proceed. He suggested that we state separate execution of sub models as a key design goal to support parallel development. Lastly, he noted that software should be released in conjunction with validation data and methodologies

### Additional v1 feedback from the google survey:

A mathematics graduate student at Rutgers University (focus on application of methods of algebraic topology, e.g. Morse theory, to dynamical systems) is interested in contributing to this project with his mathematics expertise or programming skills.

### Core team discussion and priorities for v2

The core team met by virtual conference on April 1, 2020 to discuss the first preprint, model results, and feedback. The core team set as priorities (1) to formalize design specifications for each individual model component and interfaces between components, (2) form teams responsible for each component, (3) focus v2 development on refactoring into this modular format, (3) begin development of the submodels, and (4) begin refine parameter estimates. The clearer specification and organization of submodels was the top priority. As time permits, it was also viewed as important to begin a receptor trafficking model.

The core team agreed to keep working via the dedicated slack workspace to rapidly coalesce on the submodel teams. Each subteam will have a separate channel in the workspace.

## Version 2 (April 1-May 9, 2020)

Version 2 was incorporated key v1 feedback, with a focus on introducing a more modular design, improving default model parameters, better initialization options, and a new ACE2 receptor trafficking submodel. This design cycle lasted longer due in part to work spent on subteam organization. As with Version 1, the Version 2 model was developed by the overall leads (Macklin, Heiland, Wang) to refine key model infrastructure for the forming subteams. (See the discussion in **Three main phases of community-driven development**.)

Version 2 also began work to test the design documents that were first discussed by the core team during the v1 model feedback. The interactive nanoHUB model included new usability refinements, notably an option to animate the model outputs.

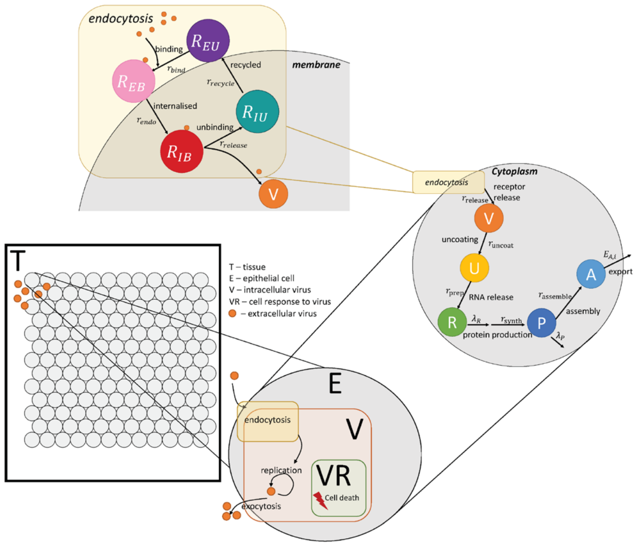

**Figure 5: Version 2 model schematic:** In the overall model structure, a ***tissue component*** **(T)** contains multiple ***epithelial cells*** **(E)**. Viral particles diffuse through this domain and can enter the cells (by adhering as an uptake term) or be exported by cells. Each cell includes an ACE2 ***receptor trafficking*** model (**RT**) where virus can bind an unoccupied surface receptor, which is endocytosed to release its virion and eventually return to the surface. Each cell's ***virus lifecycle*** model **(V)** simulates viral replication kinetics, and its ***viral response*** model **(VR)** uses a pharmacodynamic model to control cell apoptotic response to viral load.

### Model changes

The v2 model was expanded to include the following submodel components:

- **T:** tissue (which contains epithelial and other cells)
- **RT:** ACE2 receptor trafficking (including virus endocytosis)
- **V:** viral endocytosis, replication, and exocytosis responses
- **VR:** cell response to viral replication, including cell death and IFN synthesis

- **E:** epithelial cell (incorporates RT, V and VR).

See **Figure 5.**

Based on community feedback, the default virion diffusion coefficient was reduced by a factor of 10 to 90 $\mu m^2$/min. We may reduce this parameter further based upon oncolytic virus therapy modeling experience by Morgan Craig and Adrianne Jenner.

### Biological hypotheses

The v2 model was similar to v1, with a simplified set of biological hypotheses:

2.T.1 Virus diffuses in the microenvironment with low diffusion coefficient
2.T.2 Virus adhesion to a cell stops its diffusion (acts as an uptake term)

2.RT.1 Virus adheres to unbound external ACE2 receptor to become external (virus)-bound ACE2 receptor
2.RT.2 Bound external ACE2 receptor is internalized (endocytosed) to become internal bound ACE2 receptor
2.RT.3 Internalized bound ACE2 receptor releases its virion and becomes unbound internalized receptor. The released virus is available for use by the viral lifecycle model **V**
2.RT.4 Internalized unbound ACE2 receptor is returned to the cell surface to become external unbound receptor
2.RT.5 Each receptor can bind to at most one virus particle.

2.V.1 Internalized virus (previously released in 2.RT.3) is uncoated
2.V.2 Uncoated virus (viral contents) lead to release of functioning RNA
2.V.3 RNA creates viral protein forever, unless it degrades
2.V.4 Viral protein is transformed to an assembled virus state
2.V.5 Assembled virus is released by the cell (exocytosed)

2.VR.1 As a proxy for viral disruption of the cell, the probability of cell death increases with the total number of assembled virions
1.VR.2 Apoptosed cells lyse and release some or all of their contents

(In the above, X.C.Y denotes prototype X, modeling component C, biological hypothesis Y, allowing us to easily refer to any individual hypothesis or assumption in discussion and community feedback.) In the next version of this model, we will use the design document protocols for each of these components.

#### Unit tests

The v2 prototype had no changes in qualitative unit tests; once the ACE2 receptor trafficking model works correctly, the model will behave as in v1.

### Translation to mathematics, rules and model components

#### Extracellular virion transport (Tissue submodel ***T***)

There were no changes in this integration-scale tissue model (T) for v2.

#### ACE2 receptor trafficking (submodel ***RT***)

For each cell, we track $R_{EU}$ (external unbound ACE2 receptors), $R_{EB}$ (external virus-bound receptors), $R_{IB}$ (internalized virus-bound receptor), and $R_{IU}$ (internalized unbound receptor). We model hypotheses 2.RT.1-2.RT.5 as a system of ordinary differential equations:

$$\frac{dR_{EU}}{dt} = -r_{\text{bind}} n_V R_{EU} + r_{\text{recycle}} R_{IU}$$

$$\frac{dR_{EB}}{dt} = r_{\text{bind}} n_V R_{EU} - r_{\text{endo}} R_{EB}$$
$$\frac{dR_{IB}}{dt} = r_{\text{endo}} R_{EB} - r_{\text{release}} R_{IB}$$
$$\frac{dR_{IU}}{dt} = r_{\text{release}} R_{IB} - r_{\text{recycle}} R_{IU}$$

As in the v1 virus model, we estimate $n_V$ (the number of extracellular virions interacting with the cell) based upon consistency with the BioFVM implementation, and set

$$r_{\text{bind}} n_V R_{EU} = U_i V_i \rho$$

where $U$ is the cell's uptake rate and $V$ is its volume, and so

$$n_V \approx V_i \rho$$
$$U_i = r_{\text{bind}} R_{\text{EU}}$$

Thus, the virus endocytosis rate varies with the availability of unbound externalized ACE2 receptor, as expected. To link with the viral replication submodel, the unbinding of virus from internalized receptor must act as a source term for the internalized virus:

$$\text{Source}_V = r_{\text{release}} R_{IB}$$

#### Intracellular viral replication dynamics (Virus lifecycle model ***V***)

We make a small modification to the internalized virus model to account for the coupling with the receptor trafficking model:

$$\frac{dV}{dt} = \text{Source}_V - r_U V$$
$$\frac{dU}{dt} = r_U V - r_P U$$
$$\frac{dR}{dt} = r_P U - \lambda_R R$$
$$\frac{dP}{dt} = r_S R - r_A P - \lambda_P P$$
$$\frac{dA}{dt} = r_A P - E_A$$

We model exocytosis by setting the export rate $E_A$ of the assembled virions, in units of virions per time:

$$E = r_E A$$

#### Cell response (Viral response submodel ***VR***)

There were no changes from the v1 model.

### Initialization

In v2, we added the option to specify the *multiplicity of infection (MOI):* the ratio of initial virions to number of epithelial cells. These virions are placed randomly in the extracellular space. (We use a default MOI = 0.01 to model a fine mist of virions landing on the tissue.) Users can also set an option to only infect the centermost cell, which sets $V = 1$ for that cell.

### Refined parameter estimates

Detailed experimental characterization of ACE2 receptor trafficking in SARS-CoV-1[132] permit an initial estimation of key model parameters. This experimental work reported that endocytosed receptors are observed in 3 hours post infection, and that 10 hours later (13 hours elapsed time), receptors are observed in vesicles. This estimates the time scale of binding and endocytosis to be on the order 3 hours, and that virion release occurs on the order of 10 hours. Thus:

$$\frac{1}{r_{\text{bind}} R_{EU}(0)} + \frac{1}{r_{\text{endo}}} \sim 3 \text{ hours}$$

and

$$\frac{1}{r_{\text{release}}} \sim 10 \text{ hours.}$$

Supposing that binding is relatively fast compared to endocytosis, we set $\frac{1}{r_{\text{bind}} R_{EU}(0)} \sim 1 \text{ min}$, and so $\frac{1}{r_{\text{endo}}} \sim 3 \text{ hours}$. The work observed recycled receptors within 14 hours (1 hour after the appearance of endocytosed receptors), so we set $\frac{1}{r_{\text{recycle}}} \sim 1 \text{ hour}$. Assuming there are 1,000 to 10,000 ACE2 receptors per cell, we set the parameters (to order of magnitude) at

$$r_{\text{bind}} = 0.001 \text{ min}^{-1}$$

$$r_{\text{endo}} = 0.01 \text{ min}^{-1}$$

$$r_{\text{release}} = 0.001 \text{ min}^{-1}$$

$$r_{\text{recycle}} = 0.01 \text{ min}^{-1}$$

$$r_{\text{bind}} = 0.001 \text{ min}^{-1}$$

The report observed expression of viral proteins by 18 hours (5 hours after viral release from endocytosed ACE2 receptors). Assuming that $r_{\text{uncoat}} \sim r_{\text{prep}} \sim r_{\text{synth}}$, each parameter has magnitude $0.01 \text{ min}^{-1}$. We shall similarly set $r_{\text{assemble}} = r_{\text{exo}} = 0.01 \text{ min}^{-1}$ in the v2 model.

### Other implementation notes

To differentiate between incoming imported and exported virions, we actually modeled two diffusing fields (for extracellular concentrations of *V* and *A*). At the end of each computational step, we manually move all of the exported assembled virions in each voxel into the concentration of diffusing virions.

By setting the virus uptake rate *U* as noted above, PhysiCell (via BioFVM) will automatically remove the correct amount of virions from the extracellular diffusing field and place them in an internalized virus particle variable *n*. By PhysiCell's automated mass conservation:

$$\Delta n = \Delta t \, r_{\text{bind}} n_V R_{EU} = \Delta t \, U_i V_i \rho.$$

If *n* was previously set to zero, then the current value of *n* represents $\Delta n$. By assumption 2.RT.5, $\Delta n$ is equal to the change in the number of external virus-bound receptors (one virion per receptor). Thus, these receptors ($\Delta n$) represent the net increase in bound external receptors. So at each time step, we:

1) Increase $R_{EB}$ by *n*
2) Decrease $R_{EU}$ by *n*
3) Set *n* = 0 (since these virions have been "delivered" to the receptor trafficking model)

### Software release

The core model associated with the v2 prototype is Version 0.2.1. The nanoHUB app associated with the v2 prototype is Version 2.1. GitHub releases and Zenodo snapshots are given in the appendix.

The cloud-hosted interactive model can be run at https://nanohub.org/tools/pc4covid19.

### Model behavior: what does the current version teach us?

Except as noted below, all simulation results use the v2 model default parameters, which are supplied in the XML configuration parameter file of the version 0.2.1 core model repository.

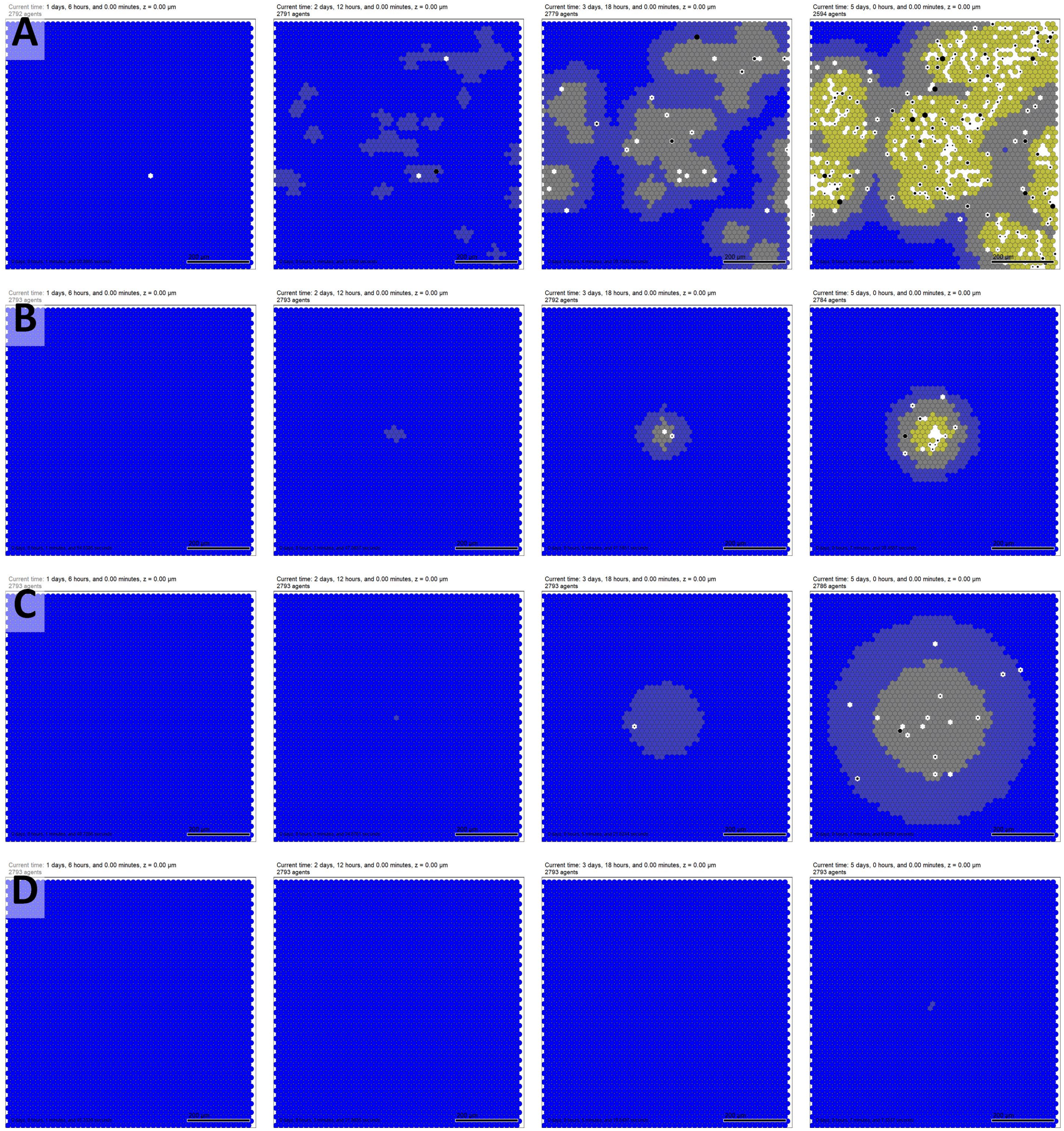

**Figure 6: Version 2 sample model results at 30, 60, 120, and 180 hours.** In all plots, epithelial cells are colored from blue (no assembled virions) to bright yellow (1000 or more virions). Black cells are apoptotic, and white regions show damaged tissues where apoptotic cells have degraded to expose (unmodeled) basement membrane. Bar: 200 $\mu$m. **A.** Simulation time course using the new initialization with an MOI (multiplicity of infection) of 0.01. As virions land randomly on the tissue, they initiate multiple infections that spread and merge. **B.** After infecting a single cell (with the new default parameters), the infected region (plaque) spreads radially as in the v1 model, but at a slower rate. As before, tissue degradation (black apoptotic cells and white cleared tissue) has greatest frequency near the original site of infection. **C.** If the number of ACE2 receptors is cut by a factor of 10, fewer virions infect cells, leading to slower viral replication. However, the reduced rate of virus binding and endocytosis leaves more extracellular viral particles to disperse, leading to a larger spread of the region of infection. **D.** Decreasing instead the rate of virus release from internalized ACE2 receptor drastically slows the viral dynamics.

In all plots, dark blue cells have 0 assembled virus, pale blue cells have 1-9 assembled virions, grey cells have 10-99 assembled virions, light yellow cells have 100-999 assembled virions, and bright yellow cells contain 1000 or more assembled virions. Black cells are apoptotic, and white spaces show regions devoid of cells (extensive tissue damage). See the legend in **Figure 4**.

#### Infection by a single virus versus a dispersion of virions

In **Figure 6** (A), we show a simulation using the new MOI initialization (MOI = 0.01), compared with the previous method of initially infecting a single cell with a single virion (B). When viral particles are randomly dispersed across the tissue (A), they nucleate multiple infections that spread as independent plaques that later merge. For higher MOIs, some cells can be infected by more than one virion, leading to faster viral replication.

#### Targeting the endocytosis cascade versus targeting ACE2 receptor

As more subcellular mechanisms are added to the model, we can ask *what if* questions about potential pharmacologic interventions[9]. Using the v2 model, we first investigated the impact of reducing the number of ACE2 receptors on each cell by a factor of 10 (e.g, by an intervention that targets ACE2 receptor or reduces its expression). We found that while this reduced the number of viral particles infecting each cell (thus slowing replication in individual cells, it paradoxically *accelerated* the spread of the infected region through the tissue. See **Figure 6** (C). This phenomenon can be understood by classical mathematical theory: the effective diffusional length scale $L$ of the virus particle is

$$L = \sqrt{\frac{D}{U}}$$

where $U$ is the uptake rate of the viral particles in the diffusion equation. In the v2 model, $U$ is proportional to the number of unbound external ACE2 receptors. If this number is reduced, then the diffusion length scale increases, leading to a faster dissemination of virus particles, exposing more tissue to virus particles, and ultimately infecting more cells earlier in the disease dynamic. On the other hand, with slower viral replication in individual cells, tissue damage may be delayed.

We similarly investigated whether decreasing the rate of viral release from virus-bound endocytosed receptors by reducing $r_{release}$ by a factor of 10. This drastically impaired the spread of the infection: ACE2 receptors trapped and internalized more viral particles, which then replicated more slowly, thus reducing the severity of the infection. See **Figure 6** (D).

### Key feedback from domain experts and the community

The core team reviewed the v2 model and project progress on April 8th, 13th, 20th, and 27th and on May 4th.

The team discussed the potential need for an improved viral replication model. In particular, for low virus counts early in a cell's infection, the continuum hypothesis needed for ordinary differential equations may not hold, and non-physical behaviors (e.g., infection by less than a single virus) may prevent the eradication of infections in the model. A discrete modeling approach may be required, although limiting mass transfers (e.g., from $R_{EU}$ to $R_{EB}$) to integer amounts could also help address this issue. The core team also reaffirmed the need to create a simplified immune system model to continue progress.

The core team also identified needed refinements in xml2jupyter, particularly the ability to run additional analytics on simulation outputs and visualize the results in the Jupyter notebook interface.

The core team formed the subteams, identified chief scientists, and organized the first subteam meetings. The core team also discussed the need to include subteam updates in the weekly core meetings. This was first implemented in the May 4, 2020 call, and the development cycle discussed above reflects these community-driven changes to team management.

### Core team discussion and priorities for v3

The highest priority for v3 is to start transitioning the development of the submodels to the subteams, thus moving the project from Phase 1 to Phase 2. In particular, the team was keen to implement a basic immune model.

## Discussion

Within three weeks of the World Health Organization's declaration of a global pandemic of COVID-19[133], community-based prototyping built upon an existing PhysiCell 3D cell-modeling framework to rapidly develop Version 1 of an intracellular and tissue-level model of SARS-CoV-2[95]. A growing coalition of domain experts from across STEM fields are working together to ensure accuracy and utility of this agent-based model of intracellular, extracellular, and multicellular SARS-CoV-2 infection dynamics. Version 1 development underscored the necessity of clearly explaining model components, defining scope, and communicating progress as it occurs for invaluable real-time feedback from collaborators and the broader community. This rapid prototyping already helped in growing the coalition and recruiting complementary expertise; for instance, a team modeling lymph node dynamics and immune infiltration joined during the Version 1 cycle after seeing initial progress.

The version 1 prototype also showed the scientific benefit of rapid prototyping: even a basic coupling between extracellular virion transport, intracellular replication dynamics, and viral response (apoptosis) showed the direct relationship between the extracellular virion transport rate and the spread of infection in a tissue. More importantly, it showed that for viruses that rapidly create and exocytose new virions, release of additional assembled virions at the time of cell death does not significantly speed the spread of infection. Moreover, decreasing the cell tolerance to viral load does not drastically change the rate at which the infection spreads, but it does accelerate the rate of tissue damage and loss, which could potentially trigger edema and ARDS earlier. This suggests that working to slow apoptosis may help preserve tissue integrity and delay adverse severe respiratory responses. That such a simple model could already point to actionable hypotheses for experimental and clinical investigations points to the value of rapid model iteration and investigation, rather than waiting for a "perfect" model that incorporates all processes with mechanistic molecular-scale detail.

Version 2 showed promise of increasing mechanistic details to evaluate potential inhibitors. For example, it was found that that reducing the expression of ACE2 receptors could paradoxically lead to faster spread of the infection across the tissue, although the individual infected cells would replicate virus more slowly. On the other hand, taking advantage of high receptor expression but interfering with viral release from internalized receptors may help slow infectious dynamics. Generally, adding sufficient actionable cell mechanisms to the model framework allows us to ask pharmacologic-driven questions on potential pharmacologic interventions, and how these finding are affected by heterogeneity, stochasticity, and the multiscale interactions in the simulated tissue.

As work on future versions progresses, teams will work in parallel on submodels to add, parameterize, and test new model components. It will be important to balance the need for new functionality with the requirement for constrained scope, while also balancing the importance of model validation with timely dissemination of results. Thus, this preprint will be updated with every development cycle to invite feedback and community contributions. Between cycles, the most up-to-date information about this model can be found at http://covid19.physicell.org.

### Getting involved

To get involved, we welcome biological expertise, especially related to model assumptions, hypotheses, infection dynamics, and interpretation of results. Mathematical contributions to the underlying model or model analysis as well as data contributions for crafting, parameterizing, and validating model predictions are particularly sought.

We encourage the community to test the web-hosted hosted model at https://nanohub.org/tools/pc4covid19. This model will be frequently updated to reflect progress, allowing the public to take advantage of this rapid prototyping effort.

We avidly encourage the community to test the model, offer feedback, and join our growing coalition via Google survey (https://forms.gle/12vmLR7aiMTHoD5YA), by direct messaging Paul Macklin on Twitter (@MathCancer), or by joining the pc4covid19 slack workspace (invitation link). Updates will frequently be disseminated on social media by Paul Macklin (@MathCancer), the PhysiCell project (@PhysiCell), the Society for Mathematical Biology subgroup for Immunobiology and Infection Subgroup (@smb_imin), and others.

We also encourage developers to watch the pc4covid19 GitHub organization and to contribute bug reports and software patches to the corresponding (sub)model repositories. See https://github.com/pc4covid19

We are encouraged by the fast recognition of the computational and infectious disease communities that we can make rapid progress against COVID-19 if we pool our expertise and resources. Together, we can make a difference in understanding viral dynamics and suggesting treatment strategies to slow infection, improve immune response, and minimize or prevent adverse immune responses. We note that this work will not only help us address SARS-CoV-2, but will also provide a framework for readiness for future emerging pathogens.

## Acknowledgements

PM thanks the Jayne Koskinas Ted Giovanis Foundation for Health and Policy for generous support. PM, RH, and YW thank the National Institutes of Health (U01-CA232137-01) for support. PM, RH, JAG, YW, and JFG thank the National Science Foundation for funding and resources via the nanoBIO Node for nanoHUB (1720625). AS thanks the NIH for support from the NIAID (R01 AI139088). MC and AJ were supported under NSERC Discovery Grant RGPIN-2018-04546. We thank the NCN CP for fast-tracked deployment of models on nanoHUB.

We thank the scientific community for model feedback, including Simon Parkinson, Richard Allen (Pfizer Inc.), David Dai (Pfizer Inc.), Rohit Rao (Pfizer Inc.) and the co-authors of this manuscript.

All the authors dedicate this work in memory of Bing Liu, our co-author, colleague, and friend. His insights and community-minded contributions are sorely missed.

## Appendices

### Appendix 1: Code availability

All code is being made available as open source under the standard 3-Clause BSD license. Users should cite this preprint (or the final published paper, as the case may be).

#### Core model releases

##### Version 1 model

###### Version 0.1.0 (released March 26, 2020)

**GitHub:** https://github.com/pc4covid19/COVID19/releases/tag/0.1.0
**Notes:** First release.

###### Version 0.1.1 (released March 26, 2020)

**GitHub:** https://github.com/pc4covid19/COVID19/tree/0.1.1
**Notes:** Minor bugfixes and first inclusion of "math" directory.

###### Version 0.1.2 (released March 26, 2020)

**GitHub:** https://github.com/pc4covid19/COVID19/releases/tag/0.1.2

**Zenodo:** https://doi.org/10.5281/zenodo.3733336
**Notes:** First release with Zenodo integration. Last release in 0.1.x chain (v1 model chain).

##### Version 0.1.3 (released April 1, 2020)

**GitHub:** https://github.com/pc4covid19/COVID19/tree/0.1.3
**Zenodo:** https://doi.org/10.5281/zenodo.3737166
**Notes:** First release after transferring the COVID19 tissue-level model (overall model) from Paul Macklin's personal GitHub account to the new pc4covid19 GitHub organization.

#### Version 2 model

##### Version 0.2.0 (released April 9, 2020)

**GitHub:** https://github.com/pc4covid19/COVID19/tree/0.2.0
**Zenodo:** https://doi.org/10.5281/zenodo.3747011
**Notes:** First v2 prototype. Introduced modular design and ACE2 receptor trafficking

##### Version 0.2.1 (released April 10, 2020)

**GitHub:** https://github.com/pc4covid19/COVID19/tree/0.2.1
**Zenodo:** https://doi.org/10.5281/zenodo.3747011
**Notes:** Minor bugfixes for cell visualization.

### nanoHUB cloud-hosted model releases

The latest version can always be accessed directly at https://nanohub.org/tools/pc4covid19

#### Version 1 model

##### Version 1.0 (released March 26, 2020):

**GitHub:** https://github.com/rheiland/pc4covid19/releases/tag/v1.0
**Zenodo:** https://zenodo.org/record/3733276#.XoOGa9NKi9t
**nanoHUB DOI:** http://dx.doi.org/doi:10.21981/19BB-HM69
**Notes:** First published version.

#### Version 2 model

##### Version 2.1 (released April 14, 2020):

**GitHub:** https://github.com/pc4covid19/pc4covid19/releases/tag/v2.1
**Zenodo:** https://zenodo.org/record/3766879#.XqWumJNKi9s
**nanoHUB DOI:** http://dx.doi.org/doi:10.21981/2B1H-GX51
**Notes:** Second published version. Moved GitHub repository to the pc4covid19 GitHub organization. Added another tab in the GUI for generating animation of cells (from SVG output files).

## Appendix 2: Organoid platform details

Aarthi Narayanan's virology lab is optimizing SARS-CoV-2 cultures in organoid model systems. The viral replication kinetics will be assessed by infection of different lung epithelial, fibroblast and endothelial cells, in addition to standard cell lines such as Vero cells, which are one of the cell lines in use for inhibitor assessment studies. Primary cells and/or cell lines will be infected with SARS-CoV-2 at increasing multiplicities of infection (MOI) and infectious viral titers in the supernatants assessed by plaque assays at multiple time points post infection (pi). This will stretch from ~3 hours post infection (phi) to 48 hpi depending on the cell type and the MOI.

In parallel, the viral genomic copy numbers will be assessed in the same supernatant samples by qRT-PCR with virus specific primers. This will provide information on how the production of infectious virions compares with the number of genomic copies available outside the cell. If the numbers are skewed in the direction of genomic copies (which may happen in the context of some kinds of inhibitors), it will shed light on the mechanisms of inhibition involving inhibition of infectivity of progeny virions.

The viral genomic copy numbers inside the cells will also be assessed by qRT-PCR and compared to the genomic copies outside the cell. This will provide direction on the efficacy of particle packaging and the extent of production of infectious versus noninfectious virus. While it will not provide directly pertinent information about the possibility of heterogeneity of released virus populations and quasispecies, it can provide initial clues in that direction, which can then trigger more specific questions and relevant approaches. These approaches will be pursued for cell lines, primary cells and, hopefully, subsequently transitioned to organoid platforms.

From a host response point of view, we will pursue two aspects: host cell death and inflammatory responses. For cell survival and death measurements, we will employ an assay that measures ATP activity in cells (hence a reflection of a live cell) in the context of infection and inhibitor treatments. For inflammatory responses, we will assess supernatants for inflammatory mediators by ELISA (multiplexed). The cells will be lysed to obtain RNA, which will be queried for transcription of several genes associated with inflammatory responses using gene expression arrays (multiplexed).

Additional host response events will include mitochondrial activity and ROS production assessments in the context of infection and inhibitor treatments. The impact of anti-inflammatory strategies on mitochondrial activity and cell survival will be assessed to determine correlations between viral replication dependent and independent events.

## Appendix 3: Overall design cycle development details

In each prototyping or design cycle:

1. The core team sets priorities for the design iteration:
    a. Discuss feedback and identify highest priority model refinements.
    b. Collaborate to update the submodel design documents to address feedback
    c. Update the overall model design document as needed.
    d. Assess new data to refine parameter estimates.
    e. Refine submodel input/output formats as necessary.
    f. Assess next release dates for the submodels.
2. Submodel teams meet to refine their code and put out their next releases. The chief scientists communicate releases to the overall leads.
3. The integration team integrates the latest submodel releases into a new release candidate for the overall model.
    a. Address any bug reports.
    b. Test new or altered functions. Satisfy all qualitative and/or quantitative unit tests.
    c. Qualitatively test the model for new or improved behaviors over the last iteration.
4. The integration team prepares a software release:
    a. Update documentation.
    b. Create a new numbered release on GitHub.
    c. Update list of available validation data and best parameter estimates.
    d. Create a Zenodo snapshot.
    e. Announce on Twitter (via @PhysiCell, @MathCancer, and @SMB_imin).
5. The integration team updates the cloud-hosted model for multidisciplinary testing:
    a. Update the nanoHUB app repository with new code.
    b. Run xml2jupyter to update the Jupyter interface.
    c. Update project on nanoHUB, test/refine until successful release
    d. Update documentation, numbered GitHub release, zenodo snapshot of deployed model.
    e. Perform live demos with domain experts and community to gather feedback.

6. The whole team seeks additional community feedback via twitter and the pc4covid19 slack workspace. The team integrates comments received from scientific peer review as appropriate.
7. The core team evaluates progress:
    a. Distill feedback to assess the need for new model hypotheses, behaviors, or components.
    b. Assess which biological behaviors are currently exhibited by the model.
    c. Refine the design protocol (e.g., with refined model specification methods) as necessary.
    d. Assess the need for an additional design iteration.
8. Update preprint for scientific dissemination. Return to Step 1 if there is substantial feedback, or if the core team determines that further refinements are within project scope.

## Appendix 4: Submodel development details

In each software sprint, each submodel team:

1. sets priorities for the design iteration:
    a. Discuss feedback and identify highest priority model refinements.
    b. Refine model assumptions and hypotheses.
    c. Assess new data to refine parameter estimates.
2. “translates” biological hypotheses into agent model rules and other mathematical model components:
    a. Run the new hypotheses and rules by domain experts as their time permits.
    b. Define new qualitative and/or quantitative unit tests for new behaviors and functions.
    c. Assign implementation tasks.
3. performs computational implementation of refined mathematical model (and submodels):
    a. Address any bug reports.
    b. Add or modify functions based on new rules in steps 1-2.
    c. Test new or altered functions. Satisfy all qualitative and/or quantitative unit tests.
    d. Qualitatively test the model for new or improved behaviors over the last iteration.
4. creates a software release:
    a. Update documentation.
    b. Create a new numbered release on GitHub.
    c. Update list of available validation data and best parameter estimates.
    d. Create a Zenodo snapshot.
    e. Communicate with the core team on the software release.
5. creates a cloud-hosted submodel for multidisciplinary testing:
    a. Update the nanoHUB app repository with new code.
    b. Run xml2jupyter to update the Jupyter interface.
    c. Update project on nanoHUB, test/refine until successful release
    d. Update documentation, numbered GitHub release, Zenodo snapshot of deployed model.
    e. Perform live demos with the core team as needed.

While waiting for the start of the next software sprint, each submodel team:

1. performs model evaluation:
    a. Distill feedback to assess the need for new model hypotheses, behaviors, or components.
    b. Assess which biological behaviors are currently exhibited by the model.
    c. Refine the design protocol (e.g., with refined model specification methods) as necessary.
    d. Assess the need for an additional design iteration.
2. Helps update the preprint for scientific dissemination.

In the next software sprint, Return to Step 1 if there is substantial feedback, or if the core team determines that further refinements are within project scope.

## References

1 Dong, E., Du, H. & Gardner, L. An interactive web-based dashboard to track COVID-19 in real time. *The Lancet Infectious Diseases*, doi:10.1016/s1473-3099(20)30120-1 (2020).

2 Wu, F. *et al.* A new coronavirus associated with human respiratory disease in China. *Nature* **579**, 265-269, doi:10.1038/s41586-020-2008-3 (2020).

3 Zhou, P. *et al.* A pneumonia outbreak associated with a new coronavirus of probable bat origin. *Nature* **579**, 270-273, doi:10.1038/s41586-020-2012-7 (2020).

4 Bouadma, L., Lescure, F.-X., Lucet, J.-C., Yazdanpanah, Y. & Timsit, J.-F. Severe SARS-CoV-2 infections: practical considerations and management strategy for intensivists. *Intensive Care Medicine* **46**, 579-582, doi:10.1007/s00134-020-05967-x (2020).

5 Yang, X. *et al.* Clinical course and outcomes of critically ill patients with SARS-CoV-2 pneumonia in Wuhan, China: a single-centered, retrospective, observational study. *The Lancet Respiratory Medicine*, doi:10.1016/s2213-2600(20)30079-5 (2020).

6 Zhou, F. *et al.* Clinical course and risk factors for mortality of adult inpatients with COVID-19 in Wuhan, China: a retrospective cohort study. *The Lancet* **395**, 1054-1062, doi:10.1016/s0140-6736(20)30566-3 (2020).

7 Zhang, J.-j. *et al.* Clinical characteristics of 140 patients infected with SARS-CoV-2 in Wuhan, China. *Allergy*, doi:10.1111/all.14238 (2020).

8 Liang, W. *et al.* Cancer patients in SARS-CoV-2 infection: a nationwide analysis in China. *The Lancet Oncology* **21**, 335-337, doi:10.1016/s1470-2045(20)30096-6 (2020).

9 Macklin, P. When Seeing Isn't Believing: How Math Can Guide Our Interpretation of Measurements and Experiments. *Cell Systems* **5**, 92-94, doi:10.1016/j.cels.2017.08.005 (2017).

10 Macklin, P. Key challenges facing data-driven multicellular systems biology. *GigaScience* **8**, doi:10.1093/gigascience/giz127 (2019).

11 Wrapp, D. *et al.* Cryo-EM structure of the 2019-nCoV spike in the prefusion conformation. *Science* **367**, 1260-1263, doi:10.1126/science.abb2507 (2020).

12 Danthi, P. Viruses and the Diversity of Cell Death. *Annual Review of Virology* **3**, 533-553, doi:10.1146/annurev-virology-110615-042435 (2016).

13 Sahin, A. R. 2019 Novel Coronavirus (COVID-19) Outbreak: A Review of the Current Literature. *Eurasian Journal of Medicine and Oncology*, doi:10.14744/ejmo.2020.12220 (2020).

14 White, K. A., Enjuanes, L. & Berkhout, B. RNA virus replication, transcription and recombination. *RNA Biology* **8**, 182-183, doi:10.4161/rna.8.2.15663 (2014).

15 Stark, G. R., Kerr, I. M., Williams, B. R. G., Silverman, R. H. & Schreiber, R. D. How Cells Respond to Interferons. *Annual Review of Biochemistry* **67**, 227-264, doi:10.1146/annurev.biochem.67.1.227 (1998).

16 Perry, A. K., Chen, G., Zheng, D., Tang, H. & Cheng, G. The host type I interferon response to viral and bacterial infections. *Cell Research* **15**, 407-422, doi:10.1038/sj.cr.7290309 (2005).

17 Spiegel, M. *et al.* Inhibition of Beta Interferon Induction by Severe Acute Respiratory Syndrome Coronavirus Suggests a Two-Step Model for Activation of Interferon Regulatory Factor 3. *Journal of Virology* **79**, 2079-2086, doi:10.1128/jvi.79.4.2079-2086.2005 (2005).

18 Hale, B. G., Randall, R. E., Ortín, J. & Jackson, D. The multifunctional NS1 protein of influenza A viruses. *Journal of General Virology* **89**, 2359-2376, doi:10.1099/vir.0.2008/004606-0 (2008).

19 Yue, Y. *et al.* SARS-Coronavirus Open Reading Frame-3a drives multimodal necrotic cell death. *Cell Death & Disease* **9**, doi:10.1038/s41419-018-0917-y (2018).

20 Keck, F. *et al.* Altered mitochondrial dynamics as a consequence of Venezuelan Equine encephalitis virus infection. *Virulence* **8**, 1849-1866, doi:10.1080/21505594.2016.1276690 (2017).

21 Keck, F. *et al.* Mitochondrial-Directed Antioxidant Reduces Microglial-Induced Inflammation in Murine In Vitro Model of TC-83 Infection. *Viruses* **10**, doi:10.3390/v10110606 (2018).

22 Thiel, V. *et al.* Lack of Innate Interferon Responses during SARS Coronavirus Infection in a Vaccination and Reinfection Ferret Model. *PLoS ONE* **7**, e45842, doi:10.1371/journal.pone.0045842 (2012).

23 Widagdo, W., Okba, N. M. A., Stalin Raj, V. & Haagmans, B. L. MERS-coronavirus: From discovery to intervention. *One Health* **3**, 11-16, doi:10.1016/j.onehlt.2016.12.001 (2017).

24 Channappanavar, R. *et al.* Dysregulated Type I Interferon and Inflammatory Monocyte-Macrophage Responses Cause Lethal Pneumonia in SARS-CoV-Infected Mice. *Cell Host & Microbe* **19**, 181-193, doi:10.1016/j.chom.2016.01.007 (2016).
25 Channappanavar, R. *et al.* IFN-I response timing relative to virus replication determines MERS coronavirus infection outcomes. *Journal of Clinical Investigation* **129**, 3625-3639, doi:10.1172/jci126363 (2019).
26 Al-Hazmi, A. Challenges presented by MERS corona virus, and SARS corona virus to global health. *Saudi Journal of Biological Sciences* **23**, 507-511, doi:10.1016/j.sjbs.2016.02.019 (2016).
27 Yuen, K.-Y. *et al.* Comparative replication and immune activation profiles of SARS-CoV-2 and SARS-CoV in human lungs: an ex vivo study with implications for the pathogenesis of COVID-19. *Clinical Infectious Diseases*, doi:10.1093/cid/ciaa410 (2020).
28 Qin, C. *et al.* Dysregulation of Immune Response in Patients with COVID-19 in Wuhan, China. *SSRN Electronic Journal*, doi:10.2139/ssrn.3541136 (2020).
29 Prompetchara, E., Ketloy, C. & Palaga, T. Immune responses in COVID-19 and potential vaccines: Lessons learned from SARS and MERS epidemic. *Asian Pacific Journal of Allergy and Immunology*, doi:10.12932/ap-200220-0772 (2020).
30 Channappanavar, R. & Perlman, S. Pathogenic human coronavirus infections: causes and consequences of cytokine storm and immunopathology. *Seminars in Immunopathology* **39**, 529-539, doi:10.1007/s00281-017-0629-x (2017).
31 Nahrendorf, M., Pittet, M. J. & Swirski, F. K. Monocytes: Protagonists of Infarct Inflammation and Repair After Myocardial Infarction. *Circulation* **121**, 2437-2445, doi:10.1161/circulationaha.109.916346 (2010).
32 Fung, S.-Y., Yuen, K.-S., Ye, Z.-W., Chan, C.-P. & Jin, D.-Y. A tug-of-war between severe acute respiratory syndrome coronavirus 2 and host antiviral defence: lessons from other pathogenic viruses. *Emerging Microbes & Infections* **9**, 558-570, doi:10.1080/22221751.2020.1736644 (2020).
33 Siu, K. L. *et al.* Severe acute respiratory syndrome Coronavirus ORF3a protein activates the NLRP3 inflammasome by promoting TRAF3-dependent ubiquitination of ASC. *The FASEB Journal* **33**, 8865-8877, doi:10.1096/fj.201802418R (2019).
34 Chen, I. Y., Moriyama, M., Chang, M.-F. & Ichinohe, T. Severe Acute Respiratory Syndrome Coronavirus Viroporin 3a Activates the NLRP3 Inflammasome. *Frontiers in Microbiology* **10**, doi:10.3389/fmicb.2019.00050 (2019).
35 Camp, J. V. & Jonsson, C. B. A Role for Neutrophils in Viral Respiratory Disease. *Frontiers in Immunology* **8**, doi:10.3389/fimmu.2017.00550 (2017).
36 Weitnauer, M., Mijošek, V. & Dalpke, A. H. Control of local immunity by airway epithelial cells. *Mucosal Immunology* **9**, 287-298, doi:10.1038/mi.2015.126 (2015).
37 Crane, M. J., Lee, K. M., FitzGerald, E. S. & Jamieson, A. M. Surviving Deadly Lung Infections: Innate Host Tolerance Mechanisms in the Pulmonary System. *Frontiers in Immunology* **9**, doi:10.3389/fimmu.2018.01421 (2018).
38 Teijaro, John R. *et al.* Endothelial Cells Are Central Orchestrators of Cytokine Amplification during Influenza Virus Infection. *Cell* **146**, 980-991, doi:10.1016/j.cell.2011.08.015 (2011).
39 Liu, J. *et al.*, doi:10.1101/2020.02.16.20023671 (2020).
40 Liu, J. *et al.*, doi:10.1101/2020.02.10.20021584 (2020).
41 Shokri, S., Mahmoudvand, S., Taherkhani, R. & Farshadpour, F. Modulation of the immune response by Middle East respiratory syndrome coronavirus. *Journal of Cellular Physiology* **234**, 2143-2151, doi:10.1002/jcp.27155 (2018).
42 Kong, W. p. *et al.* Modulation of the Immune Response to the Severe Acute Respiratory Syndrome Spike Glycoprotein by Gene-Based and Inactivated Virus Immunization. *Journal of Virology* **79**, 13915-13923, doi:10.1128/jvi.79.22.13915-13923.2005 (2005).
43 Cruz, J. L. G. *et al.* Alphacoronavirus Protein 7 Modulates Host Innate Immune Response. *Journal of Virology* **87**, 9754-9767, doi:10.1128/jvi.01032-13 (2013).
44 Liu, L. *et al.* Anti–spike IgG causes severe acute lung injury by skewing macrophage responses during acute SARS-CoV infection. *JCI Insight* **4**, doi:10.1172/jci.insight.123158 (2019).
45 Leong, A. S. Y. *et al.* Multiple organ infection and the pathogenesis of SARS. *Journal of Experimental Medicine* **202**, 415-424, doi:10.1084/jem.20050828 (2005).

46 Yang, P. *et al.* Angiotensin-converting enzyme 2 (ACE2) mediates influenza H7N9 virus-induced acute lung injury. *Scientific Reports* **4**, doi:10.1038/srep07027 (2014).
47 Kuba, K. *et al.* A crucial role of angiotensin converting enzyme 2 (ACE2) in SARS coronavirus–induced lung injury. *Nature Medicine* **11**, 875-879, doi:10.1038/nm1267 (2005).
48 Stebbing, J. *et al.* COVID-19: combining antiviral and anti-inflammatory treatments. *The Lancet Infectious Diseases* **20**, 400-402, doi:10.1016/s1473-3099(20)30132-8 (2020).
49 Herold, T. *et al.*, doi:10.1101/2020.04.01.20047381 (2020).
50 Zhang, C., Wu, Z., Li, J.-W., Zhao, H. & Wang, G.-Q. The cytokine release syndrome (CRS) of severe COVID-19 and Interleukin-6 receptor (IL-6R) antagonist Tocilizumab may be the key to reduce the mortality. *International Journal of Antimicrobial Agents*, 105954, doi:10.1016/j.ijantimicag.2020.105954 (2020).
51 Schett, G., Sticherling, M. & Neurath, M. F. COVID-19: risk for cytokine targeting in chronic inflammatory diseases? *Nature Reviews Immunology*, doi:10.1038/s41577-020-0312-7 (2020).
52 Sanders, C. J. *et al.* Compromised respiratory function in lethal influenza infection is characterized by the depletion of type I alveolar epithelial cells beyond threshold levels. *American Journal of Physiology-Lung Cellular and Molecular Physiology* **304**, L481-L488, doi:10.1152/ajplung.00343.2012 (2013).
53 Madjid, M., Safavi-Naeini, P., Solomon, S. D. & Vardeny, O. Potential Effects of Coronaviruses on the Cardiovascular System: A Review. *JAMA Cardiol*, doi:10.1001/jamacardio.2020.1286 (2020).
54 Wadman, M., Couzin-Frankel, J., Kaiser, J. & Matacic, C. A rampage through the body. *Science* **368**, 356-360, doi:10.1126/science.368.6489.356 (2020).
55 Allen, R. J., Rieger, T. R. & Musante, C. J. Efficient Generation and Selection of Virtual Populations in Quantitative Systems Pharmacology Models. *CPT: Pharmacometrics & Systems Pharmacology* **5**, 140-146, doi:10.1002/psp4.12063 (2016).
56 Cassidy, T. & Craig, M. Determinants of combination GM-CSF immunotherapy and oncolytic virotherapy success identified through in silico treatment personalization. *PLOS Computational Biology* **15**, doi:10.1371/journal.pcbi.1007495 (2019).
57 Nowak, M. *et al.* Antigenic diversity thresholds and the development of AIDS. *Science* **254**, 963-969, doi:10.1126/science.1683006 (1991).
58 Wei, X. *et al.* Viral dynamics in human immunodeficiency virus type 1 infection. *Nature* **373**, 117-122, doi:10.1038/373117a0 (1995).
59 Rosenbloom, D. I. S., Hill, A. L., Laskey, S. B. & Siliciano, R. F. Re-evaluating evolution in the HIV reservoir. *Nature* **551**, E6-E9, doi:10.1038/nature24634 (2017).
60 Reeves, D. B. *et al.* A majority of HIV persistence during antiretroviral therapy is due to infected cell proliferation. *Nature Communications* **9**, doi:10.1038/s41467-018-06843-5 (2018).
61 Koelle, K., Farrell, A. P., Brooke, C. B. & Ke, R. Within-host infectious disease models accommodating cellular coinfection, with an application to influenza†. *Virus Evolution* **5**, doi:10.1093/ve/vez018 (2019).
62 Perelson, A. S., Neumann, A. U., Markowitz, M., Leonard, J. M. & Ho, D. D. HIV-1 Dynamics in Vivo: Virion Clearance Rate, Infected Cell Life-Span, and Viral Generation Time. *Science* **271**, 1582-1586, doi:10.1126/science.271.5255.1582 (1996).
63 Smith, A. M. & Ribeiro, R. M. Modeling the Viral Dynamics of Influenza A Virus Infection. *Critical Reviews™ in Immunology* **30**, 291-298, doi:10.1615/CritRevImmunol.v30.i3.60 (2010).
64 Smith, A. M. & Perelson, A. S. Influenza A virus infection kinetics: quantitative data and models. *Wiley Interdisciplinary Reviews: Systems Biology and Medicine* **3**, 429-445, doi:10.1002/wsbm.129 (2011).
65 Smith, A. M., McCullers, J. A. & Adler, F. R. Mathematical model of a three-stage innate immune response to a pneumococcal lung infection. *Journal of Theoretical Biology* **276**, 106-116, doi:10.1016/j.jtbi.2011.01.052 (2011).
66 Bonhoeffer, S., May, R. M., Shaw, G. M. & Nowak, M. A. Virus dynamics and drug therapy. *Proceedings of the National Academy of Sciences* **94**, 6971-6976, doi:10.1073/pnas.94.13.6971 (1997).
67 Hill, A. L., Rosenbloom, D. I. S., Fu, F., Nowak, M. A. & Siliciano, R. F. Predicting the outcomes of treatment to eradicate the latent reservoir for HIV-1. *Proceedings of the National Academy of Sciences* **111**, 13475-13480, doi:10.1073/pnas.1406663111 (2014).
68 Rosenbloom, D. I. S., Hill, A. L., Rabi, S. A., Siliciano, R. F. & Nowak, M. A. Antiretroviral dynamics determines HIV evolution and predicts therapy outcome. *Nature Medicine* **18**, 1378-1385, doi:10.1038/nm.2892 (2012).

69 Mueller, S. N. *et al.* Mathematical Modeling Predicts that Increased HSV-2 Shedding in HIV-1 Infected Persons Is Due to Poor Immunologic Control in Ganglia and Genital Mucosa. *Plos One* **11**, e0155124, doi:10.1371/journal.pone.0155124 (2016).

70 Perelson, A. S. *et al.* Decay characteristics of HIV-1-infected compartments during combination therapy. *Nature* **387**, 188-191, doi:10.1038/387188a0 (1997).

71 Kirtane, A. R. *et al.* Development of an oral once-weekly drug delivery system for HIV antiretroviral therapy. *Nature Communications* **9**, doi:10.1038/s41467-017-02294-6 (2018).

72 Schiffer, J. T. & Gottlieb, S. L. Biologic interactions between HSV-2 and HIV-1 and possible implications for HSV vaccine development. *Vaccine* **37**, 7363-7371, doi:10.1016/j.vaccine.2017.09.044 (2019).

73 Schiffer, J. T. *et al.* Mathematical modeling of herpes simplex virus-2 suppression with pritelivir predicts trial outcomes. *Science Translational Medicine* **8**, 324ra315-324ra315, doi:10.1126/scitranslmed.aad6654 (2016).

74 Perelson, A. S. Modelling viral and immune system dynamics. *Nature Reviews Immunology* **2**, 28-36, doi:10.1038/nri700 (2002).

75 Hill, A. L., Rosenbloom, D. I. S., Nowak, M. A. & Siliciano, R. F. Insight into treatment of HIV infection from viral dynamics models. *Immunological Reviews* **285**, 9-25, doi:10.1111/imr.12698 (2018).

76 Wang, Y., Zhou, Y., Brauer, F. & Heffernan, J. M. Viral dynamics model with CTL immune response incorporating antiretroviral therapy. *Journal of Mathematical Biology* **67**, 901-934, doi:10.1007/s00285-012-0580-3 (2012).

77 Schiffer, J. T. & Corey, L. Rapid host immune response and viral dynamics in herpes simplex virus-2 infection. *Nature Medicine* **19**, 280-288, doi:10.1038/nm.3103 (2013).

78 Jenner, A. L., Yun, C.-O., Kim, P. S. & Coster, A. C. F. Mathematical Modelling of the Interaction Between Cancer Cells and an Oncolytic Virus: Insights into the Effects of Treatment Protocols. *Bulletin of Mathematical Biology* **80**, 1615-1629, doi:10.1007/s11538-018-0424-4 (2018).

79 Möhler, L., Flockerzi, D., Sann, H. & Reichl, U. Mathematical model of influenza A virus production in large-scale microcarrier culture. *Biotechnology and Bioengineering* **90**, 46-58, doi:10.1002/bit.20363 (2005).

80 Schulze-Horsel, J., Schulze, M., Agalaridis, G., Genzel, Y. & Reichl, U. Infection dynamics and virus-induced apoptosis in cell culture-based influenza vaccine production—Flow cytometry and mathematical modeling. *Vaccine* **27**, 2712-2722, doi:10.1016/j.vaccine.2009.02.027 (2009).

81 Banks, H. T., Bortz, D. M. & Holte, S. E. Incorporation of variability into the modeling of viral delays in HIV infection dynamics. *Mathematical Biosciences* **183**, 63-91, doi:10.1016/s0025-5564(02)00218-3 (2003).

82 Culshaw, R. V., Ruan, S. & Webb, G. A mathematical model of cell-to-cell spread of HIV-1 that includes a time delay. *Journal of Mathematical Biology* **46**, 425-444, doi:10.1007/s00285-002-0191-5 (2003).

83 Li, M. Y. & Shu, H. Impact of Intracellular Delays and Target-Cell Dynamics on In Vivo Viral Infections. *SIAM Journal on Applied Mathematics* **70**, 2434-2448, doi:10.1137/090779322 (2010).

84 Smith, A. M. Validated models of immune response to virus infection. *Current Opinion in Systems Biology* **12**, 46-52, doi:10.1016/j.coisb.2018.10.005 (2018).

85 Smith, A. M. Host-pathogen kinetics during influenza infection and coinfection: insights from predictive modeling. *Immunological Reviews* **285**, 97-112, doi:10.1111/imr.12692 (2018).

86 Guedj, J. *et al.* Modeling shows that the NS5A inhibitor daclatasvir has two modes of action and yields a shorter estimate of the hepatitis C virus half-life. *Proceedings of the National Academy of Sciences* **110**, 3991-3996, doi:10.1073/pnas.1203110110 (2013).

87 Heldt, F. S., Frensing, T. & Reichl, U. Modeling the Intracellular Dynamics of Influenza Virus Replication To Understand the Control of Viral RNA Synthesis. *Journal of Virology* **86**, 7806-7817, doi:10.1128/jvi.00080-12 (2012).

88 Sidorenko, Y. & Reichl, U. Structured model of influenza virus replication in MDCK cells. *Biotechnology and Bioengineering* **88**, 1-14, doi:10.1002/bit.20096 (2004).

89 Fachada, N., Lopes, V. V. & Rosa, A. Simulating antigenic drift and shift in influenza A. 2093, doi:10.1145/1529282.1529744 (2009).

90 Bauer, A. L., Beauchemin, C. A. A. & Perelson, A. S. Agent-based modeling of host–pathogen systems: The successes and challenges. *Information Sciences* **179**, 1379-1389, doi:10.1016/j.ins.2008.11.012 (2009).

91 Wilke, C. O. *et al.* Complex Spatial Dynamics of Oncolytic Viruses In Vitro: Mathematical and Experimental Approaches. *PLoS Computational Biology* **8**, e1002547, doi:10.1371/journal.pcbi.1002547 (2012).
92 Sun, G.-Q. *et al.* Spatiotemporal Dynamics of Virus Infection Spreading in Tissues. *Plos One* **11**, e0168576, doi:10.1371/journal.pone.0168576 (2016).
93 Jenner, A. L., Frascoli, F., Coster, A. C. F. & Kim, P. S. Enhancing oncolytic virotherapy: Observations from a Voronoi Cell-Based model. *Journal of Theoretical Biology* **485**, 110052, doi:10.1016/j.jtbi.2019.110052 (2020).
94 Quintela, B. M. *et al.* An Age-based Multiscale Mathematical Model of the Hepatitis C Virus Life-cycle During Infection and Therapy: Including Translation and Replication. **60**, 508-511, doi:10.1007/978-981-10-4086-3_128 (2017).
95 Ghaffarizadeh, A., Heiland, R., Friedman, S. H., Mumenthaler, S. M. & Macklin, P. PhysiCell: An open source physics-based cell simulator for 3-D multicellular systems. *PLoS Comput Biol* **14**, e1005991, doi:10.1371/journal.pcbi.1005991 (2018).
96 Cooper, F. *et al.* Chaste: Cancer, Heart and Soft Tissue Environment. *Journal of Open Source Software* **5**, 1848, doi:10.21105/joss.01848 (2020).
97 Prlic, A. *et al.* Chaste: An Open Source C++ Library for Computational Physiology and Biology. *PLoS Computational Biology* **9**, e1002970, doi:10.1371/journal.pcbi.1002970 (2013).
98 Starruß, J., de Back, W., Brusch, L. & Deutsch, A. Morpheus: a user-friendly modeling environment for multiscale and multicellular systems biology. *Bioinformatics* **30**, 1331-1332, doi:10.1093/bioinformatics/btt772 (2014).
99 Sego, T. J. *et al.* A Modular Framework for Multiscale Spatial Modeling of Viral Infection and Immune Response in Epithelial Tissue. *bioRxiv*, 2020.2004.2027.064139, doi:10.1101/2020.04.27.064139 (2020).
100 Kang, S., Kahan, S., McDermott, J., Flann, N. & Shmulevich, I. Biocellion: accelerating computer simulation of multicellular biological system models. *Bioinformatics* **30**, 3101-3108, doi:10.1093/bioinformatics/btu498 (2014).
101 Hillen, T. *COVID-19 Physiology Group*, <https://sites.google.com/ualberta.ca/cov-pg/home> (2020).
102 *COVID-19 Cell Atlas*, <https://www.covid19cellatlas.org/> (2020).
103 Hu, B. C. The human body at cellular resolution: the NIH Human Biomolecular Atlas Program. *Nature* **574**, 187-192, doi:10.1038/s41586-019-1629-x (2019).
104 Metzcar, J., Wang, Y., Heiland, R. & Macklin, P. A Review of Cell-Based Computational Modeling in Cancer Biology. *JCO Clin Cancer Inform* **3**, 1-13, doi:10.1200/CCI.18.00069 (2019).
105 Ghaffarizadeh, A., Friedman, S. H. & Macklin, P. BioFVM: an efficient, parallelized diffusive transport solver for 3-D biological simulations. *Bioinformatics* **32**, 1256-1258, doi:10.1093/bioinformatics/btv730 (2016).
106 Wang, Y., Heiland, R. & Macklin, P. *pc4nanobio: cancer nanotherapy simulator [nanoHUB app, Version 0.9.1]*, <https://nanohub.org/resources/pc4nanobio> (2019).
107 Jenner, A. L. *Replication Competent Oncolytic Virus expressing secretable trimeric TRAIL: hypothesis testing [nanoHUB app, Version 0.3]*, <https://nanohub.org/resources/iu399sp19p101> (2019).
108 Wang, Y., Heiland, R. & Macklin, P. *Physicell: liver tissue mechanobiology [nanoHUB app, Version 1.2]*, <https://nanohub.org/resources/pc4livermedium> (2019).
109 Ozik, J. *et al.* High-throughput cancer hypothesis testing with an integrated PhysiCell-EMEWS workflow. *BMC Bioinformatics* **19**, 483, doi:10.1186/s12859-018-2510-x (2018).
110 Ozik, J., Collier, N., Heiland, R., An, G. & Macklin, P. Learning-accelerated discovery of immune-tumour interactions. *Mol Syst Des Eng* **4**, 747-760, doi:10.1039/c9me00036d (2019).
111 Macklin, P. *UCI Systems Biology Foundation Short Course - PhysiCell Short Course*, <https://github.com/physicell-training/UCI-sysbio-2020> (2020).
112 Hucka, M. *et al.* The systems biology markup language (SBML): a medium for representation and exchange of biochemical network models. *Bioinformatics* **19**, 524-531, doi:10.1093/bioinformatics/btg015 (2003).
113 Somogyi, E. T. *et al.* libRoadRunner: a high performance SBML simulation and analysis library. *Bioinformatics* **31**, 3315-3321, doi:10.1093/bioinformatics/btv363 (2015).

114 Stoll, G. *et al.* MaBoSS 2.0: an environment for stochastic Boolean modeling. *Bioinformatics* **33**, 2226-2228, doi:10.1093/bioinformatics/btx123 (2017).
115 Letort, G. *et al.* PhysiBoSS: a multi-scale agent-based modelling framework integrating physical dimension and cell signalling. *Bioinformatics* **35**, 1188-1196, doi:10.1093/bioinformatics/bty766 (2019).
116 Cockrell, C. & An, G. Sepsis reconsidered: Identifying novel metrics for behavioral landscape characterization with a high-performance computing implementation of an agent-based model. *J Theor Biol* **430**, 157-168, doi:10.1016/j.jtbi.2017.07.016 (2017).
117 Cockrell, R. C. & An, G. Examining the controllability of sepsis using genetic algorithms on an agent-based model of systemic inflammation. *PLOS Computational Biology* **14**, e1005876, doi:10.1371/journal.pcbi.1005876 (2018).
118 Ozik, J., Collier, N. T., Wozniak, J. M. & Spagnuolo, C. in *2016 Winter Simulation Conference (WSC)* 206-220 (2016).
119 Ozik, J., Collier, N. T., Wozniak, J. M., Macal, C. M. & An, G. Extreme-Scale Dynamic Exploration of a Distributed Agent-Based Model With the EMEWS Framework. *IEEE Transactions on Computational Social Systems* **5**, 884-895, doi:10.1109/tcss.2018.2859189 (2018).
120 Wozniak, J. M. *et al.* CANDLE/Supervisor: a workflow framework for machine learning applied to cancer research. *BMC Bioinformatics* **19**, doi:10.1186/s12859-018-2508-4 (2018).
121 Khanna, A. S. *et al.* A modeling framework to inform preexposure prophylaxis initiation and retention scale-up in the context of 'Getting to Zero' initiatives. *Aids* **33**, 1911-1922, doi:10.1097/qad.0000000000002290 (2019).
122 Tatara, E. *et al.* in *2019 Winter Simulation Conference (WSC)* 1008-1019 (2019).
123 Rutter, C. M., Ozik, J., DeYoreo, M. & Collier, N. Microsimulation model calibration using incremental mixture approximate Bayesian computation. *The Annals of Applied Statistics* **13**, 2189-2212, doi:10.1214/19-aoas1279 (2019).
124 Madhavan, K. *et al.* nanoHUB.org: cloud-based services for nanoscale modeling, simulation, and education. *Nanotechnology Reviews* **2**, doi:10.1515/ntrev-2012-0043 (2013).
125 Kluyver, T. *et al.* in *ELPUB.* 87-90.
126 Friedman, S. H. *et al.* MultiCellDS: a community-developed standard for curating microenvironment-dependent multicellular data [**Preprint**]. *bioRxiv* **090456**, doi:10.1101/090456 (2016).
127 Heiland, R., Mishler, D., Zhang, T., Bower, E. & Macklin, P. xml2jupyter: Mapping parameters between XML and Jupyter widgets. *J Open Source Softw* **4**, doi:10.21105/joss.01408 (2019).
128 Macklin, P., Edgerton, M. E., Thompson, A. M. & Cristini, V. Patient-calibrated agent-based modelling of ductal carcinoma in situ (DCIS): from microscopic measurements to macroscopic predictions of clinical progression. *J Theor Biol* **301**, 122-140, doi:10.1016/j.jtbi.2012.02.002 (2012).
129 Lowe, D. *Angiotensin and the Coronavirus*, <https://blogs.sciencemag.org/pipeline/archives/2020/03/17/angiotensin-and-the-coronavirus> (2020).
130 Beauchemin, C., Forrest, S. & Koster, F. T. 23-36 (Springer Berlin Heidelberg).
131 Sniekers, Y. H. & van Donkelaar, C. C. Determining Diffusion Coefficients in Inhomogeneous Tissues Using Fluorescence Recovery after Photobleaching. *Biophysical Journal* **89**, 1302-1307, doi:10.1529/biophysj.104.053652 (2005).
132 Wang, H. *et al.* SARS coronavirus entry into host cells through a novel clathrin- and caveolae-independent endocytic pathway. *Cell Research* **18**, 290-301, doi:10.1038/cr.2008.15 (2008).
133 Organization, W. H. *WHO Director-General's opening remarks at the media briefing on COVID-19 - 11 March 2020*, <https://www.who.int/dg/speeches/detail/who-director-general-s-opening-remarks-at-the-media-briefing-on-covid-19---11-march-2020> (2020).
