# Iterative community-driven development of a SARS-CoV-2 tissue simulator

**DOI:** 10.1101/2020.04.02.019075

**Published:** 2021-04-29

**Authors:** Michael Getz, Yafei Wang, Gary An, Maansi Asthana, Andrew Becker, Chase Cockrell, Nicholson Collier, Morgan Craig, Courtney L. Davis, James R. Faeder, Ashlee N. Ford Versypt, Tarunendu Mapder, Juliano F. Gianlupi, James A. Glazier, Sara Hamis, Randy Heiland, Thomas Hillen, Dennis Hou, Mohammad Aminul Islam, Adrianne L. Jenner, Furkan Kurtoglu, Caroline I. Larkin, Bing Liu, Fiona Macfarlane, Pablo Maygrundter, Penelope A Morel, Aarthi Narayanan, Jonathan Ozik, Elsje Pienaar, Padmini Rangamani, Ali Sinan Saglam, Jason Edward Shoemaker, Amber M. Smith, Jordan J.A. Weaver, Paul Macklin

**Affiliations:** 1 Department of Intelligent Systems Engineering, Indiana University. Bloomington, IN USA; 2 The University of Vermont Medical Center, Burlington, VT USA; 3 Decision and Infrastructure Sciences, Argonne National Laboratory. Lemont, IL USA; 4 Consortium for Advanced Science and Engineering, University of Chicago. Chicago, IL USA; 5 Department of Mathematics, University of Montreal. Montreal, QC Canada; 6 CHU Sainte-Justine Research Centre, Montreal, QC Canada; 7 Natural Science Division, Pepperdine University, Malibu, CA USA; 8 Department of Computational and Systems Biology, University of Pittsburgh. Pittsburgh, PA USA; 9 School of Chemical Engineering, Oklahoma State University, Stillwater, OK USA; 10 Oklahoma Center for Respiratory and Infectious Diseases, Oklahoma State University, Stillwater, OK USA; 11 Department of Chemical and Biological Engineering, University at Buffalo, The State University of New York, Buffalo, NY USA; 12 School of Mathematics and Statistics, University of St Andrews, St Andrews, Scotland; 13 Department of Mathematical and Statistical Sciences, University of Alberta. Edmonton, AB Canada; 14 Department of Mathematics, Rutgers University. New Brunswick, NJ USA; 15 Citizen scientist. Austin, TX USA; 16 Department of Immunology, University of Pittsburgh. Pittsburgh, PA USA; 17 National Center for Biodefense and Infectious Disease, George Mason University. Manassas, VA USA; 18 Weldon School of Biomedical Engineering, Purdue University. West Lafayette, IN USA; 19 Department of Mechanical and Aerospace Engineering, University of California. San Diego, CA USA; 20 Department of Chemical and Petroleum Engineering, University of Pittsburgh. Pittsburgh, PA USA; 21 Department of Pediatrics, University of Tennessee Health Science Center, Memphis, TN USA; 22 Division of Clinical Pharmacology, Department of Medicine, Indiana University School of Medicine, Indianapolis, IN, USA; 23 Agricultural and Biological Engineering, Purdue University. West Lafayette, IN USA

## Abstract

The 2019 novel coronavirus, SARS-CoV-2, is a pathogen of critical significance to international public health. Knowledge of the interplay between molecular-scale virus-receptor interactions, single-cell viral replication, intracellular-scale viral transport, and emergent tissue-scale viral propagation is limited. Moreover, little is known about immune system-virus-tissue interactions and how these can result in low-level (asymptomatic) infections in some cases and acute respiratory distress syndrome (ARDS) in others, particularly with respect to presentation in different age groups or pre-existing inflammatory risk factors. Given the nonlinear interactions within and among each of these processes, multiscale simulation models can shed light on the emergent dynamics that lead to divergent outcomes, identify actionable “choke points” for pharmacologic interventions, screen potential therapies, and identify potential biomarkers that differentiate patient outcomes. Given the complexity of the problem and the acute need for an actionable model to guide therapy discovery and optimization, we introduce and iteratively refine a prototype of a multiscale model of SARS-CoV-2 dynamics in lung tissue. The first prototype model was built and shared internationally as open source code and an online interactive model in under 12 hours, and community domain expertise is driving regular refinements. In a sustained community effort, this consortium is integrating data and expertise across virology, immunology, mathematical biology, quantitative systems physiology, cloud and high performance computing, and other domains to accelerate our response to this critical threat to international health. More broadly, this effort is creating a reusable, modular framework for studying viral replication and immune response in tissues, which can also potentially be adapted to related problems in immunology and immunotherapy.

## Introduction

The ongoing pandemic caused by the novel severe acute respiratory syndrome coronavirus 2 (SARS-CoV-2) has illuminated the global public health threat posed by highly pathogenic coronaviruses that emerge from zoonotic sources. With the majority of the world’s population immunologically naïve and no available antivirals or vaccines, over 144,000,000 infections and 3,000,000 deaths amassed worldwide by the end of April 2021^1^. Coronavirus disease 2019 (COVID-19)—caused by SARS-CoV-2 infection—is characterized by a range of respiratory symptoms, including fever and cough^2,3^, that can progress to acute respiratory distress syndrome (ARDS) in some patients^4,5^. Age and comorbidities appear to be the main risk factors for development of severe disease^6–8^. However, the dynamics of virus replication, interaction with host immune responses, and spread within the respiratory tract are still being established. Despite the fact that several vaccines are now available, these are not yet widely distributed and there remains a critical need to further understand the infection in order to quickly identify pharmacologic interventions and optimal therapeutic designs that work to lessen virus replication and disease severity. However, this requires an international community effort that integrates expertise across a variety of domains and a platform that can be iteratively updated as new information and data arises.

To aid this effort, we have assembled an international, multi-disciplinary coalition to rapidly develop an open source, multi-scale tissue simulator that can be used to investigate mechanisms of intracellular viral replication, infection of epithelial cells, host immune response, and tissue damage. The aim of this project is to concentrate community modeling efforts to create a comprehensive multiscale simulation framework that can subsequently be calibrated, validated, and used to rapidly explore and optimize therapeutic interventions for COVID-19. Once the prototype has been completed (after several design iterations), this coalition will transition to maintain and support the simulation framework and aggregate calibrated/validated parameter values.

To address the acute need for rapid access to an actionable model, we are using a community-driven coalition and best open science practices to build and iteratively refine the model:

1. **Modular and reusable:** The model is built from mechanistic “first principles” based on domain expertise in immunology, virology, and tissue biology. Separate generalized modules for key processes (virus-receptor binding and endocytosis, replication, interferon responses, cell death, and local and systemic responses) ensure that the model can be adapted and calibrated to new viruses and new treatments without the need for relearning model rules from fresh data.
2. **Open source and GitHub:** All simulation source code is shared as open source on GitHub, with well-defined, versioned, and documented releases, and Zenodo-generated archives and DOIs.
3. **Interactive cloud-hosted models:** Every prototype version is rapidly transformed into a cloud-hosted, interactive model to permit faster scientific communication across communities, particularly with virologists, immunologists, pharmacologists, and others who have essential insights but ordinarily would not directly run the simulation models.
4. **Social media and virtual feedback:** We enlist community participation (feedback, modeling contributions, software contributions, and data contributions) through social media, virtual seminars, web forms, a dedicated Slack workspace, and weekly team meetings. We particularly encouraging feedback and data contributions by domain experts in virology, epidemiology, immunology, and mathematical biology.
5. **Frequent preprint updates:** Each model iteration is accompanied by a cloud-hosted, interactive app (see #3) and an updated preprint on *bioRxiv*.
6. **Integration of feedback:** All community feedback is evaluated to plan the next set of model refinements and recorded in an updated *bioRxiv* preprint.

Our first test of this workflow saw a first proof-of-concept software release (Steps 2–3) in 12 hours, and the first integration of community feedback and preprint dissemination was complete within a week. We have begun integrating community feedback, and it is our intention to continue iterate refinement.

### Goals and guiding principles

This project is community-driven, including the following contributions:

1. **Community priorities:** The community helps define the driving research questions, set the project scope, and select the critical biological components to be modeled.
2. **Consensus hypotheses:** The community drives a shared, clearly-written consensus specification of the underlying biological hypotheses.
3. **Mathematical modeling:** The community helps develop, review, and refine the mathematical interpretation of the biological hypotheses.
4. **Computational implementation:** The computational implementation is shared as open source with community definition of specifications, unit tests, coding, and code review.
5. **Community feedback:** Community feedback on the model realism, hypotheses, mathematics, computational implementation, and techniques is encouraged throughout the development process.
6. **Community parameters and data:** Community contributions of parameter estimates and data contributions are aggregated to assist in model development and constraint.

#### Project scope

While by definition the project scope can be refined by the community, the initial project scope is to:

1. Develop the general computational framework sufficiently to address many of the community-driven research questions.
2. Deliver a working simulation framework for use by others to perform calibration and validation. That is, the prototyping aims of this project are complete once the model is capable of demonstrating essentialbiological behaviors qualitatively.
3. To provide a software framework whose underlying hypotheses, mathematics, and computational implementation have been rigorously assessed by appropriate domain experts.

In particular, while this project will work to constrain, estimate, and calibrate parameters to the greatest extent possible, it is *not* within scope to delay software release until full calibration and validation. Those tasks are within scope of fully funded teams with dedicated experiments.

This project aims to deliver software that one can reasonably expect to calibrate and validate, thus freeing funded investigations from expensive early software development while providing a broad community consensus on key biological hypotheses. By rapidly prototyping this software, we aim to accelerate many funded research efforts.

#### Essential model components

As part of defining the project scope, we have identified the following critical model components:

1. Virus dissemination in epithelial tissue
2. Virus binding, endocytosis, replication, and exocytosis
3. Infected cell responses, including changes to metabolism, secreted signals, and death
4. Inflammatory response
5. Ramp up of the immune response (particularly in lymph nodes)
6. Immune cell infiltration
7. Immune cell predation of infected and other cells
8. Tissue damage by death of cells due to infection or host response

#### Guiding principles

The coalition aims to model not merely the disease endpoints, but the disease *dynamics*. This will allow scientists to investigate mechanistic “what if” questions on potential interventions: *What if* we could inhibit endocytosis? *What if* we could introduce a cytokine early or late in the disease course? *What if* the infected cell apoptosis could be accelerated or delayed?

To accomplish this, we use a modular design: an overall tissue-scale model integrates an array of targeted *submodels* that simulate critical processes (e.g., receptor binding and trafficking and virus replication). Each submodel is clearly specified to enable interoperability and to make it feasible for subteams to simultaneously develop and test the model components in parallel. Throughout development, we use open source methodologies that enhance communication, transparency, and reproducibility. See Box 1.

#### Critical questions for the model framework

The community identified a series of driving biological questions concerning COVID-19 to guide the development of the model framework (see Box 2). It is expected that the model will not initially be able to address all the questions listed; rather, the development plan envisions that with each iteration of the model framework it will expand in its representational capacity as directed by the topics listed in Box 2. Furthermore, as with all modeling projects, we anticipate that as the framework develops it will generate new questions and/or be responsive to the rapidly evolving knowledge-base concerning COVID-19.

### Key biology for the simulation model

This rapid prototyping effort brings together specialists from a broad variety of domains: virology and infectious diseases, mathematical biology, computer science, high performance computing, data science, and other disciplines. Therefore, it is critical that all members of the project have access to a clear description of underlying biology that drive the model’s assumptions. In this section we outline key aspects of viral replication and host response in functional terms needed for development of agent-based, multi-scale and multi-physics models.

#### Cell infection and viral replication

The key cell-level process is viral infection of a single cell, followed by replication to create new virions:

1. SARS-CoV-2 is a single-stranded enveloped RNA virus^11^. A virion (single virus particle) has a lipid coating (envelope) that protects the virus when outside a cell (or host). Each virus has dozens of spike glycoproteins that bind to ACE2 (receptors) on select cell membranes^3,11^.
2. Virions travel in the tissue microenvironment to reach a cell membrane. The spike binds to an available ACE2 receptor on the cell membrane. Prior to binding to the ACE2 receptor, the spike is cleaved by the protease, TMPRSS2, which is required for efficient cell entry^12^. Multiple modes of transport (e.g., passive diffusion in fluids and porous tissues, mucociliary clearance, chemotaxis, ultrafiltration driven by hydro-static and oncotic pressure through permeable cell junctions, and cellular active transport) may play a role at slow and fast time scales. There may also be surface contact transmission between neighboring cells.
3. The cell internalizes the adhered virus via endocytosis into a vesicle.
4. The endocytosed virion—now residing in a vesicle with lowered pH—is uncoated to release its mRNA contents into the cell cytoplasm.
5. Copying viral RNA creates a (−) RNA template, which is used for (+) RNA production.
6. RNA is used to synthesize viral RNA and proteins.
7. Viral proteins are transported to the interior surface of the cell membrane.
8. Viral proteins at the cell membrane are assembled into virions.
9. Assembled virions are exported from the cell by exocytosis.
10. When a cell dies and lyses, some or all partly and fully assembled virions can be released into the tissue microenvironment.

Once infected, an individual cell cannot “recover” (e.g., by actively degrading viral RNA and stopping endocytosis) to return to normal function. Rather, the cell is irreversibly committed to eventual death. For further detail, see review articles on RNA virus replication dynamics^13,14^.

#### Infected cell responses

Although infected cells (e.g., type 1 or type 2 alveolar cells in the lung) cannot recover, their respond can slow viral replication and reduce infection of nearby cells. Infected cells do this by secreting type I interferons (IFN-α,β), which diffuse and bind to receptors on nearby cells to reduce viral replication following infection, activate an inflammatory response, and induce gene transcription^15^ to minimize cycling and/or induce apoptosis in these cells^16^. Secreted IFN-α,β are important activators and regulators of the innate and adaptive immune responses^16^. Many respiratory viruses, including influenza and SARS-CoVs^17^, have evolved to inhibit IFN activation^18^, and evidence is emerging that certain non-structural proteins produced by SARS-CoV-2 infected cells interfere with IFN-*α*,*β* and chemokines by inhibiting production and suppressing signaling^17,18^.

Eventually, infected cells die (by apoptosis, necroptosis, or pyroptosis^19^), lyse, and release unassembled viral components^19^. While the mechanism of cell death in SARS-CoV-2 is currently unknown, in other RNA virus infections, cells can undergo apoptotic, necrotic, or pyroptotic death over the course of viral infection^20^. Disruption of cell metabolism and competition for critical substrates may also contribute to cell death^21,22^.

#### Inflammatory and immune responses

Lethal SARS and MERS in humans has been correlated with elevated IFN-α,β^23^, myeloid activity, and impaired T and B cells^24,25^, with the timing of IFN-*α*,*β* critical^26,27^. IFN-*α*,*β*s secreted by infected cells or by immune cells diffuse to surrounding cells and recruit innate immune cells, such as macrophages and neutrophils, to the area. Recent studies comparing SARS-CoV-2 with SARS-CoV have revealed that SARS-CoV-2 replicates more efficiently in pneumocytes and alveolar macrophages, but IFN-*α*,*β* secretion is blunted in SARS-CoV-2 infected cells^28^. In COVID-19 patients, decreased numbers of T cells, natural killer (NK) cells, and, to a lesser extent, B cells occur, and the extent of depletion of T cells has been correlated with disease severity^2,3,29^. Rapid inhibition of viral replication requires early and high levels of IFN-*α*,*β* secretion and activation^30^. The production of excess inflammatory cytokines, such as IL-1, IL-6 and TNF-*α* and other chemokines by infected cells results in increased macrophage and neutrophil presence, which correlates with lung dysfunction^31,32^. Delayed IFN-α,β production also promotes inflammatory macrophage recruitment that contributes to vascular leakage and impaired T-cell function^26,27^. Activated macrophages also produce other proinflammatory cytokines like IL-1, IL-6, and TNF-α, among others, that enhance infiltration of immune cells and interact with endothelial cells to cause vasodilation^33^. The excess production of IL-1 and IL-6 may be related to several viral proteins shown to directly activate the inflammasome pathway, the innate immune response responsible for IL-1β secretion^34–36^. Moreover, epithelial tissue death can reduce tissue integrity, contributing to further immune infiltration, fluid leakage and edema, and acute respiratory distress^37–39^.

In severe cases, a “cytokine storm” of pro-inflammatory cytokines (e.g., IL-2, IL-7, IL-10, G-CSF, IP-10, MCP-1, MIP-1A, and TNF-α) induces extensive tissue damage^31^. During influenza virus infection, there is some evidence that ARDS is correlated with the extent of infection in the lower respiratory tract and increased cytokine activity resulting from exposure of the endothelium^40^. Increases in neutrophil counts and the neutrophil-to-lymphocyte ratio (NLR) have been observed in patients with severe COVID-19^41^. The NLR has also been shown to be an important clinical predictor of disease severity^42^, as it reflects the innate to adaptive immune response. Neutrophils generally produce reactive oxygen species ROS, which can induce the death of infected and healthy cells in the local environment, further contributing to tissue damage^37^.

Coronaviruses have been shown to evade and modulate various host immune responses^43–45^. In addition to those discussed above, some evidence suggests that an antibody to spike protein enhances disease during SARS-CoV infection by inducing macrophage switching from a wound healing phenotype to an inflammatory phenotype^46^. Furthermore, influenza viruses and other SARS-CoVs are known to infect macrophages and T cells^3,47^, raising the possibility for SARS-CoV-2 to similarly infect these cell types. However, it has not yet been shown that SARS-CoV-2 infection of these cells is productive or simply alters their cytokine expression^31^. However, the ACE2 receptor has been linked to acute lung injury for both influenza and SARS-CoV viruses^48,49^.

#### Links between inflammation and poor clinical outcomes

Coronavirus death is often correlated with pre-existing medical conditions such as diabetes, hypertension, cardiac disease and obesity^6,50,51^. While the primary effect of SARS-CoV-2 is clearly the lung infection, several secondary effects play a role in the clinical outcome for a given patient. The complex interactions of viral infection, cytokine production, immune response, and pre-existing diseases is an active field of current research. Although the underlying risk factors for an individual to develop ARDS in response to SARS-CoV-2 infection have not yet been elucidated, it appears that a dysregulated immune response is central to this aspect of the disease^2,3,29,52^. In particular, chemokines are released following viral infection, which leads to the invasion of neutrophils and macrophages and release of ROS. IL-6 levels have been associated with more severe disease as patients who required ventilation exhibit increased systemic IL-6 levels, as reported by studies from Germany and China^53–55^. In addition, replication in the lower airways and exposure of endothelial cells may further amplify the inflammatory response^40^. Collectively, this leads to extensive tissue damage and depletion of epithelial cells, which may be connected to lethality^56^. Within the alveolar tissue and systemically, the feedback between viral load, adaptive and innate immune responses, and tissue damage is clearly a complex system. By utilizing a multi-scale framework to implement these interactions, we aim to connect circulating biomarkers, putative treatments, and clinically observed disease progression to pathophysiological changes at the cell and tissue level.

### Anticipated data to drive development and validation

It is important that model development considers the types of measurements and biological observations that will be available for later model constraint, calibration, and validation. As participation by the virology and pharmacology communities broadens, we anticipate that this list will grow. While we will endeavor to constrain and validate submodels of the model framework independently, we anticipate human clinical data to not fully determine parameters of the model. To address this concern, we will apply a “virtual population” approach and sensitivity analysis to explore model variability within clinically relevant bounds^57,58^. We anticipate the following data:

#### Organoid data for viral replication and targeted inhibition

Aarthi Narayanan’s virology lab is optimizing SARS-CoV-2 cultures in organoid model systems. These 3D model systems infect epithelial cells co-cultured with fibroblasts and endothelial cells and track the viral replication kinetics under control conditions and after treatment by inhibitors. These experiments measure (at various time points) infectious viral titers and genomic copy numbers in supernatants (outside the cells), viral genomic copy numbers in the infected cellş host cell death and inflammatory responses, and ATP and mitochondrial disruptions. See Appendix 2 for further detail.

#### Inflammation, ACE2 binding, and host response data

The international focus on SARS-CoV-2 is generating an unprecedented amount of mechanistic clinical and preclinical data. Randomized controlled interventional trials in general or specific populations will be of particular value to test and refine the model. As of April 30 2021, there were 2,852 trials registered at clinicaltrials.gov under the search term “COVID-19+Drug”. Within these 2,852 trials, there are multiple interventions at different points of the pathophysiology, including, but not limited to: broad acting antivirals (e.g., remdesivir), hyperimmune plasma, IL-6 antibody (e.g., tocilizumab), protease inhibitors (e.g., lopinavir/ritonavir), chloroquine/hydroxychloroquine, and Janus kinase inhibitors (e.g., baricitinib). As this platform develops, we anticipate predicting the responses to such therapies and refining the model with emerging data such that the range of clinical responses are captured with adequate fidelity. In addition, data collected from patients or animals during infection, including the presence of various immune cell subsets in lung tissue and systemic markers of inflammation, will serve to differentiate responses to SARS-CoV-2. These data will be similarly integrated to calibrate and validate the model to ensure accurate predictions of therapeutic outcomes based on clinical characteristics.

### Relevant prior modeling

Spurred initially by the emergence of HIV and relevant to the present SARS-CoV-2 pandemic, the field of viral dynamics modeling has been instrumental for understanding the evolution of host-virus interactions^59–67^, predicting treatment responses^68–72^, and designing novel and more effective therapeutic approaches^73–75^. The classic within-host mathematical model of viral infection uses a system of ordinary differential equations (ODEs) to describe the dynamics between uninfected epithelial (“target”) cells, infected cells in the eclipse phase, infected cells producing virus, and infectious virus^76^. This basic model has been shown to capture dynamics of both acute and chronic infection^77^, and has been extended to also include multiple viral (potentially resistant) strains^73^ and various aspects of host immune responses^78,79^. While such cell population-level ODE models generally do not account for single-cell effects, they are effective for detailing viral load, host immune response, and pathology dynamics^80–85^. Moreover, they can often be used to constrain and estimate parameters for more detailed models, develop novel hypotheses, and design confirmatory experiments^86,87^.

Some have modeled intracellular virus replication, including detailed models used for understanding replication and intervention points^58,88^, typically using systems of ODEs^89,90^. These models often include virus-receptor binding, receptor trafficking, endocytosis, viral uncoating, RNA transcription, protein synthesis, viral assembly, and viral exocytosis. However, to date no such model has been integrated with detailed spatiotemporal models of viral propagation in 3D tissues with dynamical models of immune interactions.

Agent-based models have been used to simulate viral propagation in 2D tissues with simplified models of viral replication in individual cells, particularly in the field of influenza virus infection^91–95^, spatial patterns from single-cell infections for a variety of other viral infections^96–98^ such as SARS^99^, and oncolytic viral therapies^100–103^. These models have generally not included detailed intracellular models of viral replication and individual cell responses to infection. However, they demonstrate the potential for including detailed intracellular models of viral replication in 2D and 3D tissues with the milieu of immune and epithelial cell types expected in actual patients, while also offering the opportunity to test hypotheses on the impact of viral mutagenicity and host cell heterogeneity on disease progression.

Agent-based models have also been used extensively to characterize the inflammatory dysfunction that produces the most severe manifestations of COVID19. The dynamics of forward feedback inflammation-induced tissue damage was examined with an early agent-based model of systemic inflammation^104^; this model was further developed into the Innate Immune Response ABM (IIRABM), which was used to perform in silico trials of anti-mediator/cytokine interventions (not too different from the types being tried for COVID19)^105^. More recently, the IIRABM has been used as a test platform for the application of genetic algorithms^106^ and model-based deep reinforcement learning^107^ to discover multi-modal and potentially adaptive mediator-directed therapies for acute systemic inflammation; this work is particularly relevant given the attempts to use anti-cytokine biologics during the current COVID19 pandemic. Finally, the IIRABM, as an endothelial-based model, was integrated with models of epithelial dysfunction to simulate individual and multiple organ dysfunction and failure seen with systemic hyper-inflammation^108^. As noted above, there are significant differences between the pathophysiology of bacterial sepsis and that of severe viral infections, but it appears that at some level of tissue damage the dynamics of the innate system come to fore in terms of the clinical significance.

The rapid prototyping approach of this coalition will use a performance-driven agent-based modeling platform^109^ to combine detailed intracellular models of viral endocytosis, replication, and exocytosis, disruption of cell processes (e.g., metabolism and compromised membranes) that culminate in cell death, inflammation signaling and immune responses, tissue damage, and other key effects outlined above in a comprehensive, open source simulation platform. We will deploy and refine interactive, web-hosted versions of the model to critical contributions by virologists, infectious disease modelers, and other domain experts. We will frequently update preprints to foster the fastest possible scientific dialog to iteratively refine this community resource.

#### Related modeling efforts and other future data sources

We are coordinating with related modeling efforts by a number of groups, such as early pilot work by David Odde and colleagues at the University of Minnesota, and early simulation work in Chaste^110,111^ (James Osborne and colleagues), Morpheus^112^ (Andreas Deutsch and colleagues), CompuCell3D^113^, and Biocellion^114^ (Ilya Shmulevich and co-workers). Thomas Hillen has hosted a COVID-19 Physiology Reading Group^115^ to exchange information and progress. Andrew Becker, Gary An, and Chase Cockrell recently adapter their prior multiscale agent-based modeling framework (CIABM) to simulate immune responses to viral respiratory infections, with a focus on SARS-CoV-2^116^. We are in regular contact with these communities to share data and biological hypotheses and to seek feedback, parameter insights, and data and code contributions.

The COVID-19 Cell Atlas^116^ organizes a variety of cell-scale datasets relevant to COVID-19; these may be of particular importance to intracellular modeling components of the project. The COVID-19 Disease Map^117^ also has a wealth of host-pathogen interaction data. The Human Biomolecular Atlas Program (HuBMAP)^118^ is creating detailed maps of the human respiratory system at cell- and molecular-scale resolution; this will be an excellent data source for tissue geometry in later versions of the model.

## Methods

### PhysiCell: agent-based cell modeling with extracellular coupling

PhysiCell is an open source simulation agent-based modeling framework for multicellular systems in 2D and 3D dynamical tissue environments^109^. (See Metzcar et al. (2019) for a general overview of agent-based modeling techniques in tissue-scale biology^119^.) In this framework, each cell (of any type) is an off-lattice agent with independent cell cycle progression, death processes, volume changes, and mechanics-driven movement. Each cell agent can have independent data and models attached to it, allowing substantial flexibility in adapting the framework to problems in cancer biology, microbiology, tissue engineering, and other fields. PhysiCell is coupled to BioFVM (an open source biological diffusion solver)^120^ to simulate the chemical microenvironment. As part of this coupling, each individual agent can secrete or uptake diffusing substrates and track the total amount of material entering and leaving the cell.

Relevant applications of PhysiCell-powered models have included modeling cancer nanotherapy^121^, oncolytic virus therapies^122^, tissue biomechanical feedbacks during tumor metastatic seeding^123^, and cancer immunology^109,124,125^. The platform was built with a focus on computational efficiency and cross-platform compatibility: the same source code can be compiled and run without modification on Linux, MacOS, and Windows, and simulations of up to 10 diffusing substrates on 10 mm^3^ of tissue with 10^4^ to 10^6^ cells are routinely performed on desktop workstations. The platform has been combined with high-throughput computing^124^ and active learning techniques^125^ to power large-scale model exploration on high performance computing resources.

### Integration of intracellular models in PhysiCell agents

Custom functions can be attached to individual cell agents to model molecular-scale, intracellular processes and to couple these with cell phenotypic parameters. These internal models are often implemented as systems of ODEs. For example, cell uptake of diffusing substrates can be coupled with a metabolism model that is defined by a system of ODEs, and the resulting energy output can be used to set the cycle progression and necrotic death probability of a cell^126^. For small systems of ODEs, these models are currently coded “by hand” with standard finite difference techniques. More complex models are written in systems biology markup language (SBML)^127^ for reliable scientific communication. Development versions of PhysiCell can read and integrate an individual SBML-encoded model in each cell agent using *libRoadrunner*—a highly efficient SBML integrator^128^. Similar approaches have been used to integrate Boolean signaling networks^129^ in PhysiCell in the PhysiBoSS extension^130^.

These approaches will initially be used to assess (1) viral replication dynamics in each cell agent, (2) cell death responses to viral load, (3) cell responses to interferons, and (4) changes in the virion endocytosis rate based on the availability of ACE2 and its receptor trafficking dynamics.

### Systems-scale lymphatic systems modeling

The adaptive immune response to viral infection at any tissue is triggered by the underlying lymph node(LN)-based systemic immune system when the infected/activated antigen presenting cells such as the dendritic cells start to migrate from the tissue to the lymphatic circulation. The time course of arrival of the dendritic cells in the lymph node is set by the departure of activated dendritic cells from the spatial model. In the LN, the proliferation, activation and clearance of the two types of helper T-cells (*T*_*H1*_ and *T*_*H2*_) and cytotoxic T-cells (T_C_) are simulated by a set of ODEs (Eq. 55–58) which then go to inform the spatial model of arrival rates of both helper T-cells and cytotoxic T-cells. The LN interaction network among the DC, Th1, Th2 are adopted assuming the entire secretory cascades of cytokines and interleukins are implicit to their concentrations. We assume that the DCs start secreting the primary pro-inflammatory cytokines while the Th1 and Th2 initiate the secondary pro- and anti-inflammatory secretions. On the consequence, all these secretions regulate the activities of the helper and cytotoxic T-cells^131^. Any transport between the LN and PhysiCell model is done only in integers and these events are performed before any diffusion or continuum process to attempt to reduce the error in decoupled solvers^132^.

### HPC-driven model exploration and parameterization

The concurrent growth and advancements in the three areas of 1) mechanistic simulation modeling, 2) advanced, AI-driven model exploration algorithms, and 3) high-performance computing (HPC) provides the opportunity for large-scale exploration of the complex design spaces in detailed dynamical simulation models. However, if we do not take deliberate efforts to formally facilitate this intersection across our research communities, we risk producing a series of disparate individual efforts, limited in interoperability, transparency, reproducibility and scalability. The EMEWS (extreme model exploration with Swift) framework^133^ was developed to directly address this issue and to provide a broadly applicable cyberinfrastructure to lower the barriers for utilization of advanced, large-scale model exploration on HPC resources. The EMEWS paradigm allows for the direct exploitation of cutting edge statistical and machine learning algorithms that make up the vibrant ecosystem of free and open source libraries that are continually added to and updated as research frontiers are expanded, all while controlling simulation workflows that can be run anywhere from desktops to campus clusters and to the largest HPC resources.

We have utilized EMEWS for learning-accelerated exploration of the parameter spaces of agent-based models of immunosurveillance against heterogeneous tumors^124,125^. The approach allowed for iterative and efficient discovery of optimal control and regression regions within biological and clinical constraints of the multi-scale biological systems. We have applied EMEWS across multiple science domains^134–136^ and developed large-scale algorithms to improve parameter estimation through approximate Bayesian computation (ABC) approaches^137^. These approaches, applied to the multi-scale modeling of SARS-CoV-2 dynamics, will provide the ability to robustly characterize model behaviors and produce improved capabilities for their interpretation.

### nanoHUB platform

The nanoHUB platform (nanohub.org)^138^ is a free, cloud-based service offering lectures, tutorials, and, of particular interest to us, interactive Web-based simulation tools. As its name implies, it is primarily focused on nanoscale science education and research. To make their simulation tools easier to use, nanoHUB provides a custom toolkit for developing graphical user interfaces (GUIs). However, since 2017, they have adopted and promoted the use of Jupyter notebooks^139^, with accompanying Python modules to provide GUI widgets and visualization. Cloud-based computing and data analysis platforms are well established now, in both academic and commercial settings. Those platforms, such as nanoHUB, that provide easy-to-use web-based GUIs and APIs and offer affordable pricing will likely have their rate of adoption continue to increase, especially among researchers who may lack the expertise or resources to install complex pieces of software.

### xml2jupyter and cloud deployment of PhysiCell models

Compiled PhysiCell models generate executable software that runs at the command line. Model parameters are set by editing XML (extensible markup language) configuration files, and the models save data as a combination of vector graphics outputs (scalable vector graphics: SVG) and XML and MATLAB data (.mat) files based on the draft MultiCellDS data standard^140^.

To facilitate rapid cloud-hosted dissemination of PhysiCell-powered models on the nanoHUB platform, we developed *xml2jupyter* to automatically generate a Jupyter-based GUI for any PhysiCell model^141^. The Jupyter notebook includes widgets to set parameters, initiate a simulation run, and visualize diffusing substrates and cell agents. In turn, we also developed a protocol to deploy the PhysiCell model and the Jupyter notebook interface on nanoHUB as a cloud-hosted, interactive model. This allows developers to rapidly convert a locally executable command-line model to a cloud-hosted shared model with graphical interface in a matter of minutes to hours (depending upon testing speed on nanoHUB).

In our rapid prototyping, we use rapidly-generated nanoHUB apps for scientific communication across disciplines; virologists, pharmacologists, and other domain experts can explore and visualize the model prototypes without need to download, compile, or understand the code. This facilitates faster multidisciplinary dialog and helps to draw in broader community feedback and contributions.

### Modular design

The model is being evolved with a modular architecture. The overall model and each individual model component (submodel) have a separate GitHub software repository in the pc4COVID-19 GitHub organization, available at https://github.org/pc4COVID-19.

Each submodel repository consists of a *master* branch that matches the latest numbered release and a *development* branch. Contributors will fork the development branch, complete their milestones, and submit a pull request to incorporate their progress in the development branch. Whenever a submodel team is ready to make a numbered release, they will use a pull request from the development branch to the master branch and create a numbered release.

The overall model framework and each submodel will keep a versioned design document to include:

- A unique name for the model component
- A clear version number and last update timestamp
- A list of contributors, including 1–2 chief scientists who serve as primary points of contact
- A “plain English” description of the primary purpose of the component
- A statement of model inputs with units of measure
- A clear statement of the biological hypotheses and assumptions of the component
- A record of the current mathematical form of the model (generally maintained in a separate Overleaf LaTeX document), with a snapshot of the equations in the main design document
- Any computational implementation details needed to understand the code
- A link to a GitHub repository
- A list of model parameters, units, biophysical meaning, best estimate, and data source(s) for the parameter estimate (see the discussion in MultiCellDS^140^)
- A clear list of model outputs with units
- A set of qualitative and/or quantitative **unit** tests to ensure proper functionality of the module.

A snapshot of this design document will be included in each release of the (sub)model.

The overall model releases will include a clear list of the version of each submodel included in its release.

### Coalition structure

After group discussion and prioritization, coalition members self-assigned themselves to one or more subteams responsible for developing the submodels. Each *subteam* has 1–2 chief scientists in charge of managing development, while a technical contact approves pull requests from the subteam’s contributors and coordinates with the integration team (see below). The submodel chief scientist(s) meet regularly with their team to assign tasks, set milestones, and assess when to make a release. The submodel chief scientist(s) also coordinate their progress with the other submodel teams.

The *integration team*—consisting of the overall leads (as of April 1, 2020: Paul Macklin, Randy Heiland, and Yafei Wang) and other contributors—is responsible for developing and maintaining the overall integrated model, integrating and testing updated submodels, and providing technical support to the subteams.

The *core team* consists of the overall leads and the chief scientists. They meet to coordinate progress of the submodels, refine project scope, exchange ideas on model hypotheses, evaluate community feedback, and plan overall strategy. They cooperate with the overall leads to create model releases (which will always bundle the most stable version of each submodel), update the nanoHUB models, and update the *bioRxiv* preprint.

The current list of subteams can be found in Box 3.

### Three main phases of community-driven development

#### Phase 1: Building the coalition and model infrastructure

In the first phase, the overall and integration leads build the overall tissue model structure (a model that integrates several independent *submodels*) and create “placeholder” models that serve as working proof-of-concept starting points for further expansion. This phase also builds and organizes the subteams responsible for the submodels and provides them with training and documentation on the model and submodel architecture.

We anticipate that Phase 1 will require six-to-eight weeks, although Phases 1 and 2 may overlap as individual subteams assume full leadership of their submodel code repositories.

#### Phase 2: Community-driven development

In this phase, the integration team transitions the primary development of each of the submodels to appropriate domain experts in the subteams, to ensure that each submodel reflects the best available science. During this phase, the integration team supports each subteam in mathematical model development, PhysiCell implementation, and nanoHUB deployment for rapid subteam testing, dissemination, and community feedback on the submodels.

The integration team continues to lead overall model integration, testing, and deployment as a cloud-hosted model, and development of further infrastructure (e.g., HPC investigations) and PhysiCell and xml2jupyter refinements needed by the subteams (e.g., full support for SBML for molecular-scale model integration).

Once the integrated model can qualitatively produce expected viral and immune behaviors (as determined by the core group) and receives no major domain expert or community critiques, the major goal of the coalition (and this paper) will be met: to create a SARS-CoV-2 modeling framework suitable for subsequent calibration, validation, and exploration by the community. It will be submitted to scientific peer review, disseminated to the community, and maintained. This will mark the conclusion of Phase 2.

We anticipate that Phase 2 will require six to twelve months.

#### Phase 3: widespread scientific use and model maintenance

Once the overall model and submodels are largely complete, the model will be a mature, open source community resource available for use in scientific investigations. Moreover, due to our iterative approach, we envision that teams will have begun using earlier versions of the model for investigations by this point. The integration team will transition to supporting parallel investigations by independent groups using the models, and aggregating and sharing best data, parameter estimation, and results. The integration team and subteams will coordinate to encourage full scientific publication of the overall model, submodels, and resulting scientific investigations.

This phase will also focus on code hardening, documentation, and development of training and outreach materials. This phase is critical for *knowledge capture*, so that the model remains usable beyond the involvement of the original teams and can be rapidly adapted to emerging health challenges in the future. We also envision continued refinement of the model to reflect improved biological knowledge.

### Iterative development

**Fig 0.1:**
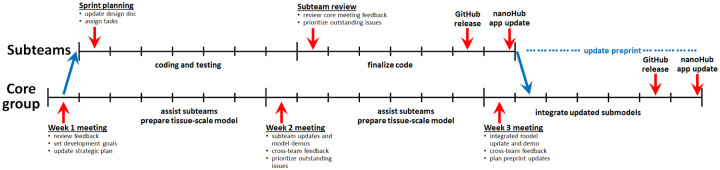
Overall development cycle (3-week example): Throughout the overall development cycle, the core team (integration leads + subteam leads) set priorities and coordinate work of the subteams. Each subteam performs a short sprint to update its submodel. At the end of the development cycle, the integration team bundles the most up-to-date submodels for the next overall model release, while the subteams update the preprint and refine domain knowledge and mathematics for the next sprint.

We use ***rapid prototyping*** using lessons learned from each step to drive iteration towards improving the model. Each submodel undergoes its own development sprints, contained within a broader development cycle for the overall integrated model (See Fig. 0.1.).

#### Overall integrated model development cycle

We aim for a short development cycle for the overall integrated model, although development cycles may last longer to accommodate building computational infrastructure, development of new core features to support model development, and training.

##### Start of cycle

The design cycle starts with an initial core team meeting where we discuss feedback from prior design cycles and set priorities for the current design cycle. In particular, we discuss:

- What changes are needed to the submodels? Prioritize changes that can be made within a 7–10 day sprint.
- What changes are needed in the overall integrative framework to facilitate the improved submodels? Set framework goals for early and mid-cycle development.
- Are any funding, personnel, scope, or other changes needed?

Within the working week, the subteams meet to further set and accomplish their sprint goals. (See Submodel design cycle). The integration team (1) works on refinements to the PhysiCell and nanoHUB frameworks to facilitate subteam work, (2) provides technical consulting to the subteams to implement their model refinements, and (3) makes any final edits needed to the preprint from the last design cycle.

##### Mid-cycle advances

The design cycle continues with a core team meeting to discuss the current subteam model sprints:

- Each team gives a brief report on their model advances and a live demo of either the standalone C++ code or a nanoHUB submodel app.
- The teams “cross-pollinate” to exchange ideas and give feedback on each of the submodels.
- The core team decides on any additional changes needed to continue the design cycle.
- The integration team and subteam chief scientists set final deadlines to end the sprints and release the updated submodels.

Within the working week, the subteams continue and complete their developing and testing for their respective sprints, create new submodel releases on GitHub, and update their submodel nanoHUB apps. The integration team continues support for the subteam work and completes any changes to the overall integrative model needed for the upcoming integration.

As the subteams advance towards their releases, the integration team updates and tests the respective submodels in the overall framework and updates the overall nanoHUB app.

##### End of cycle

The design cycle nears completion with a core team meeting to discuss integration and preprinting:

- The integration team gives an update on current integration testing.
- The team coordinates any remaining submodel releases.
- The team sets plans for updating the preprint.

Within the working week, the **subteams** complete their releases (if not already complete in week 2) and begin revising the preprint. They also begin testing the integrated model as it comes online to integrate new simulation results and insights into the preprint.

The **integration team** updates the submodels, performs final testing, and creates both GitHub and nanoHUB releases. Once complete, the integration team joins the subteams on preprint updates. We note that in non-summer months, the coalition was not able to maintain a three-week development pace.

#### Submodel design cycle

Each submodel is developed in parallel with a unified design cycle (a 7-to-14-day software *sprint*) in coordination with the other subteams during the weekly core team meetings and via a dedicated Slack workspace.

##### Start of sprint

The sprint cycle starts with an initial subteam meeting to communicate the results and priorities of the core team meeting to the subteam. The team edits the submodel design document, discusses any necessary changes to the mathematics and parameter values, and assigns implementation tasks. The team coordinates with the integration team via the Slack workspace for any needed assistance on model implementation.

##### End of sprint

The design cycle continues with a core team meeting to discuss the current subteam model sprints:

- Each team gives a brief report on their model advances and a live demo of either the standalone C++ code or a nanoHUB submodel app.
- The teams “cross-pollinate” to exchange ideas and give feedback on each of the submodels.
- The core team decides on any additional changes needed to continue the design cycle.
- The integration team and subteam chief scientists set final deadlines to end the sprints and release the updated submodels.

Within the working week, the **subteams** continue and complete their developing and testing for their respective sprints, create new submodel releases on GitHub, and update their submodel nanoHUB aps. The **integration team** continues support for the subteam work and completes any changes to the overall integrative model needed for the upcoming integration.

As the subteams advance towards their releases, the **integration team** updates and tests the respective submodels in the overall framework and updates the overall nanoHUB app.

See Appendix 4**: Submodel development details** for more implementation details.

## Results

### Summary of Versions 1–3

The **Version 1** model prototyped the initial modular framework: a layer of susceptible, uninfected cells was modeled as cell agents. Each cell agent had an independent viral replication model (a system of five ordinary differential equations), and a (death) response model to viral load. As virus was assembled in each cell, it was released into the extracellular environment. Extracellular viral dissemination was modeled as a reaction-diffusion equation, and virus binding to (susceptible) cells was modeled as an uptake term that transferred extracellular diffusing virus to the intracellular ODE model. The model was initialized by placing a single virion in the center of the (extracellular) simulation domain. The model was able to capture a single expanding viral plaque, with greatest (single-cell) viral load in the center and expanding outwards, with greatest death and tissue disruption in the center of the plaque.

The **Version 2** model further modularized the framework into more distinct software modules of the submodels, while also introducing an ACE2 receptor trafficking model (four ODEs for internal and external bound and unbound receptors) to each cell agent. The model also introduced a more realistic starting condition of multiple virions entering the extracellular at random locations according to a user-defined multiplicity of infection (MOI). This model was able to further explore the impact of receptor trafficking limits on the rate of viral spread.

Versions 1–2 were performed during Phase 1 (building the coalition and model infrastructure). The **Version 3** model was the first released developed in Phase 2 (community-driven development). Its major change was to introduce an agent-based immune model of macrophages, neutrophils, and effector T cells. Resident macrophages, upon phagocytosing dead infected cells, secreted pro-inflammatory factors to stochastically recruit neutrophils and effector T cells that could hunt and kill infected cells. These model additions allowed us to explore the impact of increasing or decreasing resident macrophage and T cell populations.

Full details on these prior model versions can be found in the supplementary materials.

### Version 4 (developed July 16, 2020-November 20, 2020)

The most significant change in Version 4 was the integration of a lymphatic system model: a systems of ordinary differential equations now represent arriving dendritic cells that drive expansion of T cell populations. Immune cells traffic between the spatially-resolved tissue model (Versions 1–3) and this new lymphatic compartment.

Version 4 also refined and expanded the version 3 minimal model of the tissue-level immune response to SARS-CoV-2. Dendritic cells and CD4^+^ T cells have been added to capture antigen presentation dynamics and the interplay between macrophages and T cell signaling. Macrophage activation and phagocytosis mechanisms have also been refined, and we have introduced a model of cell death via pyroptosis. Fibroblast-mediated collagen deposition has been included to account for the fibrosis at the damaged site in response to immune response-induced tissue injury. Epithelial cells’ production of Type-I Interferons and Interferon Stimulated Genes’ effects on viral replication have been included.

While the version 4 code was released in November 2020, simulation runs, analysis, and scientific documentation continued through April 2021 concurrently with Version 5 code development.

#### Biological hypotheses

The v4 immune submodel introduces dendritic cells and CD4^+^ T cells as a result of (assumed) antigen presentation in the lymph node as well as the resulting recruitment of CD8^+^ and CD4^+^ T cells to tissue. Immune cell recruitment is expanded. Rules regarding macrophage function have been altered to allow for variable engulfment or activation states as well as T cell modulation of macrophage activity. This version also introduces a model of cell death via pyroptosis. Fibroblast recruitment and collagen secretion are also considered for fibrosis. Below, assumptions are indicated by X.C.Y, where X denotes prototype, C denoted modeling component, and Y denotes a biological hypothesis, for easy reference. Assumptions in **bold** have been added or modified from the previous model version.

4.T.1 Virus diffuses in the microenvironment with low diffusion coefficient
4.T.2 Virus adhesion to a cell stops its diffusion (acts as an uptake term)
4.T.3 Pro-inflammatory cytokine diffuses in the microenvironment
4.T.4 Pro-inflammatory cytokine is taken up by recruited immune cells
4.T.5 Pro-inflammatory cytokine is eliminated or cleared
4.T.6 Chemokine diffuses in the microenvironment
4.T.7 Chemokine is taken up by immune cells during chemotaxis
4.T.8 Chemokine is eliminated or cleared
4.T.9 Debris diffuses in the microenvironment
4.T.10 Debris is taken up by macrophages and neutrophils during chemotaxis
4.T.11 Debris is eliminated or cleared
**4.T.12** Anti-inflammatory cytokine is triggered for constant secretion at the site and time that a CD8^+^ T cell kills an epithelial cell
**4.T.13** Anti-inflammatory cytokine diffuses in the microenvironment
**4.T.14** Anti-inflammatory cytokine is eliminated or cleared
**4.T.15** Collagen is non-diffusive
4.RT.1 Virus adheres to unbound external ACE2 receptor to become external (virus)-bound ACE2 receptor
4.RT.2 Bound external ACE2 receptor is internalized (endocytosed) to become internal bound ACE2 receptor
4.RT.3 Internalized bound ACE2 receptor releases its virion and becomes unbound internalized receptor. The released virus is available for use by the viral lifecycle model **V**
4.RT.4 Internalized unbound ACE2 receptor is returned to the cell surface to become external unbound receptor
4.RT.5 Each receptor can bind to at most one virus particle.
4.V.1 Internalized virus (previously released in 4.RT.3) is uncoated
4.V.2 Uncoated virus (viral contents) lead to release of functioning RNA
4.V.3 RNA creates viral protein at a constant rate unless it degrades
**4.V.4** Viral RNA is replicated at a rate that saturates with the amount of viral RNA
**4.V.5** Viral RNA undergoes constitutive (first order) degradation
4.V.6 Viral protein is transformed to an assembled virus state
4.V.7 Assembled virus is released by the cell (exocytosis)
4.VR.1 After infection, cells secrete chemokine
4.VR.2 As a proxy for viral disruption of the cell, the probability of cell death increases with the total number of assembled virions
4.VR.3 Apoptosed cells lyse and release some or all of their contents
**4.VR.4** Once viral RNA exceeds a particular threshold *(max_apoptosis_half_max)*, the cell enters the pyroptosis cascade
**4.VR.5** Once pyropotosis begins, the intracellular cascade is modelled by a system of ODEs monitoring cytokine production and cell volume swelling
**4.VR.6** Cell secretion rate for pro-inflammatory increases to include secretion rate of IL-18
**4.VR.7** Cell secretes IL-1β which causes a bystander effect initiating pyroptosis in neighboring cells
**4.VR.8** Cell lyses (dying and releasing its contents) once its volume has exceed 1.5× the homeostatic volume
4.E.1 Live epithelial cells undergo apoptosis after sufficient cumulative contact time with adhered CD8^+^ T cells.
4.E.2 Live epithelial cells follow all rules of RT
4.E.3 Live epithelial cells follow all rules of V
4.E.4 Live epithelial cells follow all rules of VR
4.E.5 Dead epithelial cells follow all rules of D.
**4.E.6** Infected epithelial cells secrete pro-inflammatory cytokine
**4.E.7** Antigen presentation in infected cells is a function of intracellular viral protein
4.D.1 Dead cells produce debris
4.MPhi.1 Resident (unactivated) and newly recruited macrophages move along debris gradients.
**4.MPhi.2** Macrophages phagocytose dead cells. Time taken for material phagocytosis is proportional to the size of the debris
4.MPhi.3 Macrophages break down phagocytosed materials
4.MPhi.4 After phagocytosing dead cells, macrophages activate and secrete pro-inflammatory cytokines
4.MPhi.5 Activated macrophages can decrease migration speed
4.MPhi.6 Activated macrophages have a higher apoptosis rate
4.MPhi.7 Activated macrophages migrate along chemokine and debris gradients
4.MPhi.8 Macrophages are recruited into tissue by pro-inflammatory cytokines
**4.MPhi.9** Macrophages can die and become dead cells only if they are in an exhausted state
**4.MPhi.10** Macrophages become exhausted (stop phagocytosing) if internalised debris is above a threshold
**4.MPhi.11** CD8^+^ T cell contact stops activated macrophage secretion of pro-inflammatory cytokine
**4.MPhi.12** CD4^+^ T cell contact induces activated macrophage phagocytosis of live infected cells
4.N.1 Neutrophils are recruited into the tissue by pro-inflammatory cytokines
4.N.2 Neutrophils die naturally and become dead cells
4.N.3 Neutrophils migrate locally in the tissue along chemokine and debris gradients
4.N.4 Neutrophils phagocytose dead cells and activate
4.N.5 Neutrophils break down phagocytosed materials
4.N.6 Activated neutrophils reduce migration speed
4.N.7 Neutrophils uptake virus
**4.DC.1** Resident DCs exist in the tissue
**4.DC.2** DCs are activated by infected cells and/or virus
**4.DC.3** Portion of activated DCs leave the tissue to travel to the lymph node
**4.DC.4** DCs chemotaxis up chemokine gradient
**4.DC.5** Activated DCs present antigen to CD8s increasing their proliferation rate and killing efficacy (doubled proliferation rate and attachment rate)
**4.DC.6** Activated DCs also regulate the CD8 level in within a threshold by enhancing CD8 clearance.
4.CD8.1 CD8^+^ T cells are recruited into the tissue by pro-inflammatory cytokines
4.CD8.2 CD8^+^ T cells apoptose naturally and become dead cells
4.CD8.3 CD8^+^ T cells move locally in the tissue along chemokine gradients
4.CD8.4 CD8^+^ T cells adhere to infected cells. Cumulated contact time with adhered CD8^+^ T cells can induce apoptosis (See 4.E.1)
**4.CD8.5** Activated DCs present antigen to CD8 T cells, which increases the CD8 T cell proliferation rate
**4.CD8.6** Activated DCs also regulate the CD8 level in within a threshold by enhancing CD8 clearance.
**4.CD4.1** CD4^+^ T cells are recruited into the tissue by pro-inflammatory cytokines
**4.CD4.2** CD4^+^ T cells apoptose naturally and become dead cells
**4.CD4.3** CD4^+^ T cells move locally in the tissue along chemokine gradients
**4.CD4.4** CD4^+^ T cells are activated in the lymph node by three signals: (1) antigenic presentation by the DCs, (2) direct activation by cytokines secreted by DCs, (3) direct activation by cytokines secreted by CD4^+^ T cells.
**4.CD4.5** CD4^+^ T cells are suppressed directly by cytokines secreted by CD4^+^ T cells.
**4.F.1** Fibroblast cells are recruited into the tissue by anti-inflammatory cytokine
**4.F.2** Fibroblast cells apoptose naturally and become dead cells
**4.F.3** Fibroblast cells move locally in the tissue along up gradients of anti-inflammatory cytokine
**4.F.4** Fibroblast cells deposit collagen continuously

#### Mathematical details retained from Version 3

##### Extracellular virion transport (Tissue submodel *T*)

To rapidly implement extracellular viral transport using existing model capabilities, we approximated the process as diffusion with a small diffusion coefficient as in prior nanoparticle models. Using the standard BioFVM formulation^120^, if *ρ* is the concentration or population density of virions (virions / μm^3^), then the population balance is modeled as a partial differential equation (PDE) for diffusion, decay, and sources and sinks from interactions with cells:

(1) $$\frac{\partial \rho }{\partial t}=D\nabla^{2}\rho -\lambda \rho +\underset{\text{cells }i}{\sum }\delta \left(x-x_{i}\right)\left(-U_{i}V_{i}\rho +E_{i}\right),$$

where *D* is the diffusion coefficient, *λ* is the net decay rate (which can include other removal processes), *δ* is the Dirac delta function, **x**_*i*_ is the position of the center of cell *i*, *U* is the uptake rate (by adhering to ACE2 and initiating endocytosis), *V* is the volume of cell *i*, and *E* is the virion export rate from the cell. Note that in the default BioFVM implementation, uptake processes are spread across the volume of a cell.

Note that virus propagation may require more explicit modeling of cell-cell surface contact in later versions, and cilia-driven advective transport and virion deposition (e.g., through airway transport).

##### Intracellular viral replication dynamics (Virus intracellular model *V*)

Within each cell, we track *V* (adhered virions in the process of endocytosis), *U* (uncoated viral RNA and proteins), *R* (viral RNA ready for protein synthesis; *R* = 1 denotes the total mRNA of one virion), P (synthesized viral proteins; P = 1 denotes sufficient viral protein to assemble a complete virion), and A (total assembled virions ready for exocytosis). Virion import (a source term for V) is handled automatically by the mass conservation terms for PhysiCell in the PDE solutions.

We model these dynamics of internalized virions through a simplified system of ODEs:

(2) $$\frac{dV}{dt}=-r_{U}V$$

(3) $$\frac{dU}{dt}=r_{U}V-r_{P}U$$

(4) $$\frac{dR}{dt}=r_{P}U-\lambda_{R}R$$

(5) $$\frac{dP}{dt}=r_{S}R-r_{A}P-\lambda_{P}P$$

(6) $$\frac{dA}{dt}=r_{A}P$$

Here, *r*_*U*_ is the viral uncoating rate, *r*_*P*_ is the rate of preparing uncoated viral RNA for protein synthesis, *r*_*S*_ is the rate of protein synthesis, *r*_*A*_ is the rate of virion assembly, *λ*_*R*_ is the degradation rate of RNA, and *λ*_*P*_ is the degradation rate of viral protein. We model exocytosis by setting the net export *E* of the assembled virions, in units of virions per time:

(7) $$E=r_{E}A,$$

where *r*_*E*_ is the assembled virus export rate.

##### Cell response (Viral response submodel *VR*)

In this proof of concept prototype, we modeled the cell death response to cell disruption but did not model interferon processes. As a simplification, we modeled cell disruption as correlated with assembled virions *A*, and we used a Hill function to relate the apoptosis rate of a cell to *A*:

(8) $$e=\frac{A^{n}}{A_{H}^{n}+A^{n}}$$

where *e* is the effect, *n* is the Hill coefficient, and *A*_*H*_ is the amount of virions at which half of the maximum effect is achieved. After calculating this effect *e*, we set the (non-necrotic) death rate as

(9) $$r_{\text{death }}=r_{\text{max}}e$$

where *r*_max_ is the maximum death rate (at full effect, *e* = 1). As analyzed for agent-based models with stochastic death rates^109,142^, in any time interval [*t*, *t*+Δ*t*], the cell has probability *r*_death_Δ*t* of starting a death process, and the mean cell survival time (for fixed *e* and thus fixed *r*_death_) is 1/*r*_death_.

In PhysiCell, we can set the lysing cells to release any fraction (0 ≤ *f*_release_ ≤ 1) of *V*, *A*, *U*, *R*, and *P* into the extracellular environment as diffusing substrates.

##### ACE2 receptor trafficking (submodel *RT*)

For each cell, we track *R*_EU_ (external unbound ACE2 receptors), *R*_EB_ (external virus-bound receptors), *R*_IB_ (internalized virus-bound receptor), and *R*_IU_ (internalized unbound receptor). We model hypotheses 2.RT.1–2.RT.5 as a system of ordinary differential equations:

(10) $$\frac{dR_{EU}}{dt}=-r_{\text{bind}}n_{V}R_{EU}+r_{\text{recycle}}R_{IU}$$

(11) $$\frac{dR_{EB}}{dt}=r_{\text{bind}}n_{V}R_{EU}-r_{\text{endo}}R_{EB}$$

(12) $$\frac{dR_{IB}}{dt}=r_{\text{endo}}R_{EB}-r_{\text{release}}R_{IB}$$

(13) $$\frac{dR_{IU}}{dt}=r_{\text{release}}R_{IB}-r_{\text{recycle}}R_{IU}$$

As in the v1 virus model, we estimate *n*_V_ (the number of extracellular virions interacting with the cell) based upon consistency with the BioFVM implementation and set

(14) $$r_{\text{bind}}n_{V}R_{EU}=U_{i}V_{i}\rho$$

where *U* is the cellular uptake rate and *V* is the volume of the cell, and so

(15) $$n_{V}\approx V_{i}\rho$$

(16) $$U_{i}=r_{\text{bind}}R_{\text{EU}}$$

Thus, the virus endocytosis rate varies with the availability of unbound externalized ACE2 receptor, as expected. To link with the viral replication submodel, the unbinding of virus from internalized receptor must act as a source term for the internalized virus:

(17) $${\text{Source}}_{V}=r_{\text{release}}R_{IB}$$

##### Intracellular viral replication dynamics (Virus lifecycle model *V*)

We make a small modification to the internalized virus model to account for the coupling with the receptor trafficking model:

(18) $$\frac{dV}{dt}={\text{Source}}_{V}-r_{U}V$$

(19) $$\frac{dU}{dt}=r_{U}V-r_{p}U$$

(20) $$\frac{dR}{dt}=r_{P}U-\lambda_{R}R$$

(21) $$\frac{dP}{dt}=r_{S}R-r_{A}P-\lambda_{P}P$$

(22) $$\frac{dA}{dt}=r_{A}P-E_{A}$$

We model exocytosis by setting the export rate *E*_*A*_ of the assembled virions, in units of virions per time:

(23) $$E=r_{E}A$$

##### Extracellular transport (Tissue submodel *T*)

Extracellular densities of pro-inflammatory cytokine and chemokine were modelled using the standard BioFVM formulation^120^, similar to that for extracellular virus (introduced above), i.e.:

(24) $$\frac{\partial \rho }{\partial t}=D\nabla^{2}\rho -\lambda \rho +\underset{\text{cells }i}{\sum }\delta \left(x-x_{i}\right)\left(S_{i}\left(\rho_{i}^{*}-\rho \right)-U_{i}\rho \right)V_{i},$$

where *D* is the diffusion coefficient of each substrate, *λ* is the net decay rate, *δ* is the discrete Dirac delta function, *x*_*i*_ is the position of the centre of cell *i*, *S*_*i*_ is the secretion rate of cell *i*, $\rho_{i}^{*}$ is the saturation density at which cell *i* stops secreting, *U*_*i*_ is the uptake rate of the substrate by cell *i*, and *V*_*i*_ is the volume of cell *i*. The concentration *ρ*, represents the density of pro-inflammatory cytokine *ρ*_cytokine_, chemokine *ρ*_chemokine_ or dead cell debris *ρ*_debris_. Similarly, diffusion, decay, secretion, and uptake parameters are all substrate specific rates, i.e. the diffusion coefficients are *D*_cytokine_, *D*_chemokine_ and *D*_debris_; the decay rates are *λ*_cytokine_, *λ*_chemokine_ and *λ*_debris_; the secretion rates are *S*_cytokine_, *S*_chemokine_ and *S*_debris_; the uptake rates are *U*_cytokine_, *U*_chemokine_ and *U*_debris_; and the saturation densities are $\rho_{\text{cytokine}}^{*}$, $\rho_{\text{chemokine}}^{*}$ and $\rho_{\text{debris}}^{*}$.

##### Cell response (Viral response submodel *VR*)

We made a small addition to the cell response model. After infection, cells start secreting chemokine at a rate

(25) $$S_{\text{chemokine}}\;\text{min}\left(1,\frac{A}{A_{H}}\right)$$

where *A* is the intracellular assembled virion count and *A*_*H*_ is the amount of assembled virions at which half of the maximum effect of virus-induced cell apoptosis is achieved. Secretion continues until the cell dies either through lysis or CD8^+^ T cell induced apoptosis.

##### Signaling, degradation, and phagocytosis of apoptotic cells (Dead cell dynamics *D*)

Cells that die release debris that attracts phagocytes and signals that that they can be cleared from the microenvironment. They secret these signals at a rate *S*_debris_.

##### Chemotaxis (Chemotaxis model *MPhi, N*, and *CD8*)

Macrophages and neutrophils undergo chemotaxis up the chemokine gradient and dead-cell debris gradients released by infected cells and dead cells respectively. The velocity of cell chemotaxis is

(26) $${\vec{v}}_{\text{mot}}=s_{\text{mot}}\frac{(1-b)\xi +b\vec{b}}{\left||(1-b)\xi +b\vec{b}|\right|}$$

where *s*_*mot*_ is the speed of chemotaxis (cell-type-specific), 0 ≤ *b* ≤ 1 is the migration bias (also cell-type-specific), *ξ* is a random unit vector direction in 3D (or 2D) and $\vec{b}$ is the migration bias direction defined by

(27) $$\vec{b}=\frac{\sigma_{\text{chemokine}}\nabla \rho_{\text{chemokine}}+\sigma_{\text{debris}}\nabla \rho_{\text{debris}}}{\left||\sigma_{\text{chemokine}}\nabla \rho_{\text{chemokine}}+\sigma_{\text{debris}}\nabla \rho_{\text{debris}}\right\|}$$

where *σ*_*chemokine*_ and *σ*_*debris*_ are the sensitivity of chemotaxis along either the chemokine or dead-cell debris gradient. CD8^+^ T cells also undergo chemotaxis, but along the chemokine gradient, i.e. *σ*_*debris*_ = 0 and *σ*_*chemokine*_ = 1. Chemotaxing cells take up chemokine at a rate *U*_*chemokine*_.

##### Phagocytosis dynamics (Phagocytosis of apoptotic cells *MPhi* and *N*)

Once a macrophage or neutrophil has found a cell to phagocytose, it reduces its speed from *s*_*mot,a*_ (active chemotaxis speed) to *s*_*mot,p*_ (phagocytosis/attached speed) and starts searching locally for material to phagocytose.

If there is a dead cell in contact with a macrophage or neutrophil (i.e., if there is a dead cell in the cell’s ~30 *μ*m mechanical interaction voxel as in PhysiCell^109^), the immune cell will phagocytose the dead cell with rate *r*_*phag*_, which is cell-type specific and reflects the efficacy with which each immune cell subtype clears debris. If the immune cell is in contact with a dead cell over a period of [*t*, *t* + Δ*t*], then the probability of phagocytosis is *r*_*phag*_Δ*t*. When an immune cell phagocytoses a dead cell, the immune cell absorbs the volume of that cell and subsequently increases its volume, i.e., the phagocytosing cell gains:

- all of the dead cell’s fluid volume;
- all of the dead cell’s nuclear solid and cytoplasmic solid volume (which are added to the nuclear cytoplasmic solid volume)

This implies that after phagocytosis within time Δ*t*, the volume of a macrophage or neutrophil *i* that phagocytoses a dead cell *j* will be given by

(28) $$V_{cs,i}(t+\Delta t)=V_{cs,i}(t)+V_{cs,j}(t)+V_{ns,j}(t),\;V_{cf,i}(t+\Delta t)=V_{cf,i}(t)+V_{Vf,j}(t),$$

where *V*_*cs,k*_ is the volume of the cytoplasmic solid volume in cell *k*, *V*_*ns,k*_ is the volume of nuclear solid volume in cell *k*, *V*_*cf,k*_ is the cytoplasmic fluid volume in cell *k*, and *V*_*Vf,k*_ is the total fluid volume of cell *k*. Because this will typically increase the cell’s volume above its “target” equilibrium volume, the standard PhysiCell volume model^109^ will begin to shrink the cell’s volume back towards its resting volume, allowing us to model degradation of phagocytosed materials. After phagocytosing dead material, macrophages start secreting pro-inflammatory cytokines at a rate *S*_*cytokine*_.

##### Neutrophil viral clearance (*N*)

Neutrophils take up extracellular virus at a rate *U*. We assume this uptake rate is equivalent to the ACE2 receptor binding rate *r*_*bind*_.

##### Immune cell recruitment (*Mphi, N*, and *CD8*)

Macrophages, neutrophils and CD8^+^ T cells are recruited to the tissue by pro-inflammatory cytokines through capillaries/vasculature in the lung. The density of vasculature accounts for approximately 8.8% of the tissue^143^. Accordingly, at the start of each simulation we randomly assign 8.8% of the tissue voxels as vasculature points through which immune cells arrive randomly throughout the course of the simulation. (Note that the v1-v3 models simulate a single layer of epithelium where immune cells are allowed to move freely through or just above the tissue; this 2-D formulation is implemented as a single layer of 3D voxels^109^.)

At regular time intervals Δ*t*_immune_, we integrate the recruitment signal to determine the number of immune cells recruited to the tissue. The number of cells recruited into the tissue between *t* and *t* + Δ*t*_immune_ varies with the pro-inflammatory cytokine recruitment signal across the tissue:

(29) $$\text{\# of recruited cells}=r_{\text{recruit}}\int_{\Omega }\text{min}\left(1,\text{max}\left(0,\frac{\rho_{\text{cytokine}}-\rho_{\text{min}}}{\rho_{\text{sat}}-\rho_{\text{min}}}\right)\right)dV\;\Delta t_{\text{immune}}$$

where Ω is the computational domain, *r*_recruit_ is the recruitment rate (per volume), *ρ*_min_ is the minimum recruitment signal, *ρ*_sat_ is the maximum (or saturating), and *ρ*_cytokine_ is the pro-inflammatory cytokine concentration. The value of *ρ*_min_, ρ_*sat*_, and *r*_recruit_ are cell-type specific, i.e. macrophages, neutrophils, and CD8^+^ T cells have different minimum and saturating recruitment signal concentrations which results in heterogenous arrival times into the tissue.

Recruited cells are randomly seeded at vessel locations. In the v3 model, we set Δ*t*_immune_ = 10 min. Notice that the number of recruited cells scales with duration of the time interval and the size of the tissue.

##### CD8^+^ T cell induction of infected cell apoptosis (*CD8* dynamic model)

When a CD8^+^ T cell is in contact with a cell (based on PhysiCell’s mechanical interaction testing; see the note in phagocytosis above) with intracellular assembled virion is greater than 1, i.e. *A* > 1, the T cell attempts to attach to the infected cell with an attachment rate *r*_attach_. Following prior immune modeling work^124,125^, if the cell is in contact for a duration Δ*t*, then the probability of forming an attachment in that time period is *r*_attach_Δ*t*. While the cells are attached, the immune cell’s cumulative CD8^+^ T cell contact time is increased by Δ*t*. The T cell has a mean attachment lifetime *T*_attach_. Between t and *t* + Δ*t*, the probability of detaching is given by Δ*t*/*T*_attach_.

We assume that an infected cell will undergo apoptosis after its cumulative attachment time exceeds a threshold *T*_CD8_contact_death_. This can be either from a single or multiple T cell attachments. All attached T cells detach when a cell undergoes apoptosis. When CD8^+^ T cells adhere to another cell, their motility is turned off, i.e. *s*_*mot,p*_ = 0, and when they detach from a cell, their speed returns to their active chemotaxis speed *s*_*mot,a*_.

#### Immune sub-model

##### Summary and key hypotheses

The overall aim of this submodel is to include features of the immune response to SARS-CoV-2 that are specific to the local tissue environment. The main immune cellular components included at this stage are tissue-resident macrophages, infiltrating neutrophils, dendritic cells, CD8^+^ and CD4^+^ T cells, all of which are recruited as the infection progresses. The general pattern of events that this model encompasses are summarized here. When epithelial cells in the tissue become infected with SARS-CoV-2, they secrete chemokines that cause macrophages to migrate towards them following a chemokine gradient; they also produce pro-inflammatory cytokines to aid in immune cell recruitment. It is assumed that immune actions only affect infected cells that are past the eclipse phase and are thus productively generating virus. The infected cells may die by apoptosis or pyroptosis following infection or by apoptosis as a result of immune killing (see the cell response model ***VR***), and dead cells will release factors that cause macrophages to migrate towards them.

Macrophages are innate immune cells that phagocytose dead cells to remove them from the tissue. During phagocytosis (which takes some time to complete), macrophages grow in size relative to the material they engulf. If the amount of internalized debris crosses a pre-determined threshold, macrophages become exhausted and stop phagocytosing new material until they either die or reduce in size so they return to the phagocytic pool after losing internalized material. When macrophages phagocytose dead cells, they also begin to secrete pro-inflammatory cytokines that recruit neutrophils and other immune cells into the tissue. Neutrophils are short-lived innate immune cells that kill infected and bystander epithelial cells and are replenished in the tissue as long as pro-inflammatory cytokines are still being produced.

The presence of virus and infected cells in the tissue induces dendritic cells to activate and egress out of tissue to lymph nodes, where they present antigen to induce activation and proliferation of virus-specific CD4^+^ and CD8^+^ T cells; a model of immune dynamics in the lymph node is being developed within the consortium (see lymph node model LN) but are not yet explicitly integrated. Some activated dendritic cells remain in tissue and can increase proliferation and cytotoxic function of recruited CD8^+^ T cells. CD8^+^ T cells and CD4^+^ T cells, presumed to be specific for SARS-CoV-2, are activated by antigen presentation in the lymph node and enter the tissue in response to pro-inflammatory cytokines around 4 days after infection. The role of CD8^+^ T cells is to kill infected cells. Death of an infected cell is more likely after prolonged contact with one or more CD8^+^ T cells. Both types of T cells additionally interact with macrophages that have phagocytosed dead cells or virus and modulate macrophage activity in two ways: CD8^+^ T cells inhibit pro-inflammatory cytokine production by macrophages, and contact with CD4^+^ T cells induces a hyperactive state in macrophages that enables them to phagocytose live infected cells. Other ways in which CD4^+^ T cells can coordinate the adaptive immune response include: different Th cell subsets, i.e. Th1 Th2, Th17 and Treg, which all may play a role in various stages of the disease by secreting distinct sets of cytokines. The ability of the CD4^+^ T cells to induce a hyperactive state would most likely be specific to Th1, whereas Th2 and Treg cells may inhibit macrophage activity. The presence of Th17 cells would likely increase the number of neutrophils. There is evidence from recent profiling of clinical COVID-19 cases that cytokine dysregulation plays an important role in determining severity. Thus, it will be important in future model versions to consider varying the functions of CD4^+^ and CD8^+^ T cells to reflect these different scenarios.

Tissue damage activates the anti-inflammatory cytokines which then in turn recruit fibroblasts. Fibroblast chemotaxis towards damage site and deposit collagen to repair the tissue. Yet, excess deposition of collagen can lead to fibrosis. See below for further details.

Immune cells travel in a biased correlated random walk along chemical gradients^109^. To control for spatial migration, the immune submodel contains three diffusing chemicals in addition to free virions. Pro-inflammatory cytokines, which are secreted by macrophages and post-eclipse phase infected cells, recruit immune cells into the tissue from circulation or lymph nodes. We assume all immune cells migrate in the tissue toward infected cells along a chemokine gradient. For simplicity, chemokines are assumed to solely be secreted by infected cells. Macrophages also migrate up a debris gradient released by apoptotic cells. Infected cells and macrophages also secrete IFN-I, which will reduce viral burst size from neighboring infected cells in future versions of the model. As described below, simulations have been performed using a field of infected epithelial cells, and the behavior of the immune cells in this tissue appears to follow the established rules.

##### Model changes

**Fig. 4.3:**
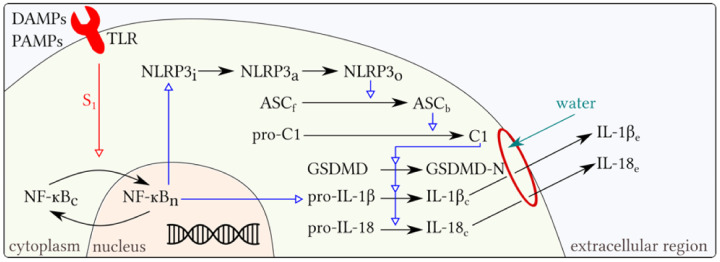
Pyroptosis pathway. Viral RNA levels within the cell act as a DAMP/PAMP that initiates the pyroptosis cascade. The intracellular processes result in the secretion of cytokines IL-1*β* and IL-18, as well as cell swelling and rupture.

The model was expanded to now include the following submodel components overall (Fig. 4.1 - 4.3):

- **T:** tissue (which contains epithelial and other cells, and diffusible factors)
- **RT:** ACE2 receptor trafficking (including virus endocytosis)
- **V:** viral endocytosis, replication, and exocytosis responses
- **VR:** cell response to viral replication, including cell death and IFN synthesis
- **E:** epithelial cell (includes RT, V and VR).
- **D:** dead cell
- **MPhi:** macrophage
- **N:** neutrophil
- **DC**: Dendritic cell
- **CD8:** CD8^+^ T cell
- **CD4**: CD4^+^ T cell
- **F**: Fibroblast

##### Chemotaxis (Chemotaxis model *DC* and *CD4*)

DCs and CD4^+^ T cells in the tissue undergo chemotaxis up the chemokine gradient using the same chemotaxis model for CD8^+^ T cells. They follow the same cell velocity and migration bias defined in Eq. (26) and (27).

##### Phagocytosis dynamics (Phagocytosis of apoptotic cells *MPhi* and *N*)

The time for macrophages to phagocytose material is proportional to the size of the apoptotic material^144,145^ they are ingesting. Given an apoptotic cell volume *V*_*apop*_ and an internalization rate *r*_*internalise*_, the time for a macrophage to internalize an apoptotic cell is *t*_*phag*_ = *V*_*apop*_/*r*_*internalise*_. During this internalization period, a macrophage is unable to phagocytose anything else.

The phagocytic capacity of an individual macrophage is finite, and experiments have shown that macrophages reach a point of saturation or exhaustion, beyond which their phagocytic activity is impaired^146^. To model this, if the volume of a particular macrophage exceeds the threshold volume *K*_*threshold*_, then it enters an exhausted state in which it can no longer phagocytose material. The standard PhysiCell volume model^109^ will shrink the cell’s volume back below this threshold (see Ghaffarizadeh *et al.*^147^ for cell-volume change details) and once the macrophages volume is again below *K*_*threshold*_, it will begin phagocytosis again. All other phagocytosis rules described previously still apply.

##### CD8^+^ and CD4^+^ T cell interaction with phagocytosing macrophages (*MPhi*)

Activated macrophages (i.e. macrophages that have phagocytosed at least one apoptotic body) interact with CD4^+^ or CD8^+^ cells. This interaction will only occur if the distance between the activated macrophage and the CD4^+^ or CD8^+^ T cell is less than the sum of their radii multiplied by the factor *ϵ*_*distance*_. A CD4^+^ T cell in the neighborhood of an activated macrophage (i.e. macrophages that have phagocytosed at least one apoptotic body) induces a hyperactive state in that macrophage. Hyperactive macrophages are able to phagocytose infected cells with at least one intracellular viral protein. Phagocytosis of infected cells follows the same phagocytosis model rules as apoptotic bodies. A CD8^+^ T cell in the neighborhood of an activated macrophage stops the macrophage’s secretion of pro-inflammatory cytokine, i.e. *s*_*chemokine*_ = 0.

##### Activation and migration of dendritic cells (*DC*)

Naïve DCs in the tissue are activated if the number of virions in the voxel containing the DC exceeds *v*_*DC*_, or if there is an infected cell in their neighborhood for the definition of neighborhood, see Ghaffarizadeh *et al.*^147^ with at least 1 viral protein. Once activated, DCs have a probability of departure to the lymph node such that the average departure time is *t*_*DC,exit*_ after activation. The amount of DCs entering the lymph node is then scaled by some scaling factor set to the number of epithelial cells present.

##### DC and CD8^+^ T cell interaction (*DC*)

An activated DC will present antigen which will induce CD8^+^ T cell proliferation at rate *γ*_*CD*8*prolif*_. Additionally, through this interaction, a CD8^+^ T cell will also increase its infected cell attachment rate *r*_attach*_. This interaction will only occur if the distance between the DC and CD8^+^ T cell is less than the sum of their radii multiplied by the factor *ϵ*_*distance*_.

##### Estimates for immune parameters

Initial number of DCs in lung tissue *DC*_0_ is approximately in the ratio of one DC to every 100 epithelial tissue cells^148^. DCs have been reported to have a body diameter ranging from 10–15*μm*^149^ however inverted microscopy and Wright-Giemsa staining show the extent to which dendrites reach outside the DC body^150^. In the 3D agent-based cellular model of lymph node DCs and T cells, Gong *et al.*^151^ modelled DCs as 4 times the size of T cells. A such, we assign DCs a diameter of 15*μm* (3.7 fold larger volume than T cells). We assumed DC migration speed in the tissue was 2–3*μm*/*min*^152^ and persistence time equivalent to CD8s (details in V3). We assume that DCs become activated if there are at least *v*_*DC*_ = 10 virions in their voxel.

In Marino *et al.’s*^153^ model for tuberculosis, they estimated that it takes an activated DC between 1hr and 24hrs to exit the lung through lymphatics. In line with this estimate, we model the exit time of activated DCs by assigning a random exit time *t*_*DC,exit*_ hours drawn from a uniform distribution *U*(1,24).

The macrophage internalization rate of dead cell debris (*r*_*internalise*_) is set to be 1*μm*^3^/*s*^144,145^. The macrophage exhaustion volume is arbitrarily chosen to be *K*_*threshold*_ = 6500, which is a 1.34 fold increase on homeostatic volume. The average diameter of a CD4^+^ T cell from Cyto-Trol is about 4.8*μm*^154^, significantly smaller than the CD8^+^ T cell diameter of 9.7*μm*. Similar to DCs, CD4^+^ T cell migration speed in the tissue was equivalent to that of CD8^+^ cells. CD8^+^ T cell interaction with an activated (antigen-presenting) DC induces CD8^+^ T cell proliferation at rate *γ*_*CD*8*prolif*_ = 0.00208 *min*^−1^. This value was estimated from a mathematical model of CD8^+^ T cell response during acute lymphocytic choriomeningitis virus^155^. We assume that DC interaction would increase CD8^+^ T cell to infected cell attachment rate *r*_*attach*_ by 50%.

A designated interaction between two immune cells will occur if the Euclidean distance between the cell centres is less than *ϵ*_*distance*_(*r*_1_ + *r*_2_), where *r*_1_ and *r*_2_ are the radii of the two immune cells potentially interaction. We chose *ϵ*_*distance*_ = 1.75, to allow cells to interact outside a small distance from their boundary.

##### Unit tests

To confirm the dynamics of the immune model qualitatively reproduce the *in-situ* dynamics, we monitored the population numbers of immune cells (macrophages, neutrophils, dendritic cells, CD8^+^ T cells, and CD4^+^ T cells) over time and compared with our biological expectations.

#### Viral binding, endocytosis, replication, and exocytosis

##### Translation to mathematics, rules, and model components

There were no changes to the ACE2 receptor trafficking model ***RT***, extracellular transport tissue model ***T*,** dead cell dynamics ***D***, chemotaxis model ***MPhi, N*** and ***CD8***, neutrophil viral clearance ***N***, and CD8 T cell induction of apoptosis model ***CD8***. Additional rules or changes to previously described rules are detailed below.

##### Minor changes to recruitment and viral uptake

Previously recruitment of immune cells used to round to the nearest int (see Eq. 29), this has been changed to always round down then do a probability draw to see if an extra cell is recruited. This leads to a smoother transition in the earlier states of recruitment.

(30) α=rate×Δtime−floor(rate×Δtime)

if *U*(0,1) < *α*,

numberofnewcells=floor(rate×Δtime)+1

In addition, to attempt to deal with a discrete to continuum transition the rule for low virion uptake by cells was changed to a probability draw when flux values are under one (i.e less than one virion will be recruited in a time-step)

(31) $$dv_{t}=r_{\text{bind}}*V_{\text{cell}}*\text{Virio}n_{\text{external}}*\text{Recepto}r_{\text{unbound}}$$

If 0 < *dv*_*t*_ < 1

(32) $$\text{virio}n_{\text{nearest}\;\text{voxel}}=\text{virio}n_{\text{nearest}\;\text{voxel}}-V_{\text{voxel}}^{-1}$$

(33) $$\text{Recepto}r_{\text{bound}}=\text{Recepto}r_{\text{bound}}+1$$

If *dv*_*t*_ > 1

(34) $$\alpha =dv_{t}-\text{floor}\left(dv_{t}\right)$$

(35) $$\Delta v_{\text{external}}=-\text{floor}\left(dv_{t}\right)*V_{\text{voxel}}^{-1}$$

(36) $$\Delta \text{Recepto}r_{\text{bound}}=dv_{t}$$

if *U*(0,1) < *α*,

(37) $$\text{virio}n_{\text{nearest}\;\text{voxel}}=\text{virio}n_{\text{nearest}\;\text{voxel}}-V_{\text{voxel}}^{-1}$$

(38) $$\text{Recepto}r_{\text{bound}}=\text{Recepto}r_{\text{bound}}+1$$

##### Minor changes to viral replication

The model now incorporates viral RNA replication within the host cell. Only the viral RNA ordinary differential equation is directly modified as follows:

(39) $$\frac{dR}{dt}=r_{p}U+\frac{r_{rep\;max}R}{R+r_{rep\;\text{half}}}-\lambda_{R}R$$

Here, r_rep max_ is the maximum replication rate of viral RNA and r_rep half_ represents the viral RNA concentration where the viral replication rate is half of r_rep max_.

#### Cell response (Viral response submodel *VR*)

##### IFN response

An early version of the IFN model was added in v4. In this model IFN interferes with protein synthesis reducing the rate of viral protein synthesis.

(40) $$r_{\text{synthesis}}=r_{\text{synthesis}}\left(1-IFN_{\text{voxel}}*IFN_{\text{maximum}}^{-1}*r_{IFN\;\text{activation}\;}\right)$$

Where $IFN_{\text{voxel}}*IFN_{\text{maximum}}^{-1}$ is bounded to 1.

IFN secretion is controlled through RNA detection and paracrine signals.

(41) $$\Delta IFN_{\text{voxel}}=\frac{RNA-RNA_{\text{detect}}}{RNA_{\text{maximum}}-RNA_{\text{detect}}}*r_{IFN\;\text{infection}\;\text{secretion}\;}+\frac{IFN_{\text{voxel}}}{IFN_{\text{maximum}}}*r_{\text{maxparacrine}}$$

Where the fractions are bounded to 1.

##### Pyroptosis

Once the viral RNA levels within a cell exceed the threshold (R≥ 200), or IL-1β levels in the microenvironment reach the threshold (Cytokine≥ 100), the cell can undergo pyroptosis, a form of inflammatory cell death^156^. The pyroptosis cascade within each cell is modelled via a system of ODEs capturing the key components of the pathway.

Many aspects of the pathway are dependent on whether the inflammasome base is still forming (*F*_*ib*_ = 1) or whether it has formed (*F*_*ib*_ = 0).

This then initiates the translocation of NF-κB into the nucleus at the rate $k_{nfkb_{ctn}}$. The NF-κB can translocate back to the cytoplasm at the rate $k_{nfkb_{ntc}}$. Therefore, we describe the evolution of nuclear NF-κB, *NFkB*_*n*_, through the equation:

(42) $$\frac{\text{dNFk}B_{n}}{dt}=F_{ib}k_{nfkb_{ctn}}\left(1-NFkB_{n}\right)-k_{nfkb_{ntc}}NFkB_{n}$$

Nuclear NF-κB then regulates the transcription of inactive NLRP3 protein, we assume this transcription follows a standard hill function form with a transcription coefficient of *a*_*nlrp*3_. This inactive NLRP3 can then become activated at the rate $k_{\text{nlrp}3_{ita}}$. We additionally assume some natural decay of the NLRP3 at the rate *d*_*nlrp*3_. We therefore describe the evolution of inactive NLRP3, *NLRP*3_*i*_, through the equation:

(43) $$\frac{\text{dNLRP}3_{i}}{dt}=a_{\text{nlrp}3}\frac{NFkB_{n}^{\gamma_{\text{nfkb}}}}{H_{\text{nfkb}}^{\gamma_{\text{nfkb}}}+NFkB_{n}{}^{\gamma_{\text{nfkb}}}}-k_{\text{nlrp}3_{ita}}\;\text{NLRP}3_{i}-d_{\text{nlrp}3}\;\text{NLRP}3_{i}.$$

Once activated NLRP3 can oligomerize to form the inflammasome base, at the rate $k_{\text{nlrp}3_{atb}}$. This process continues until enough NLRP3 has oligomerised/bound together to form the inflammasome base when *F*_*ib*_ switches to zero. We describe the evolution of active NLRP3, *NLRP*3_*a*_, through:

(44) $$\frac{\text{dNLRP}3_{a}}{dt}=k_{\text{nlrp}3_{ita}}\text{NLRP}3_{i}-k_{\text{nlrp}3_{atb}}F_{ib}\;\text{NLRP}3_{a}-d_{\text{nlrp}3}\text{NLRP}3_{a}.$$

The evolution of bound NLRP3, *NLRP*3_*b*_, is described through:

(45) $$\frac{\text{dNLRP}3_{b}}{dt}=k_{\text{nlrp}3_{atb}}F_{ib}\text{NLRP}3_{a}.$$

Once *NLRP*3_*b*_ ≥ 1, then the inflammasome base has formed and *F*_*ib*_ switches to zero.

Once the inflammasome base has formed, ASC protein is recruited and binds to the inflammasome at the rate $k_{asc_{ftb}}$. The change of bound ASC, *ADC*_*b*_, is described through:

(46) $$\frac{dASC_{b}}{dt}=k_{asc_{ftb}}\left(1-F_{ib}\right)\text{NLRP}3_{b}\left(1-ASC_{b}\right).$$

Pro-caspase 1 is then recruited to the inflammasome site, and is cleaved by bound ASC to become caspase 1, at the rate $k_{c1_{ftb}}$. Therefore, caspase 1, *C*_1_, evolves through:

(47) $$\frac{dC_{1}}{dt}=k_{c1_{ftb}}ASC_{b}\left(1-C_{1}\right).$$

Caspase 1 has the capacity to cleave gasdermin and pro-interleukins within the cell. We assume that caspase cleavage of these molecules follows a hill function with coefficients *a*_…_. Therefore, the cleaved N terminal of gasdermin, *GSDMD*_*n*_, evolves through:

(48) $$\frac{\text{dGSDM}D_{n}}{dt}=a_{\text{gsdmd}}\frac{C_{1}^{\gamma_{c1}}}{\left(H_{c1}^{\gamma_{c1}}+C_{1}^{\gamma_{c1}}\right)}\left(1-\text{GSDM}D_{n}\right).$$

Similarly to NLRP3, we assume that pro-interleukin 1β, *IL*1*b*_*p*_, is transcribed by NF-κB and decays at the rate *d*_*il*_. The pro-form is then cleaved by caspase 1 to form the cytoplasmic form, that is:

(49) $$\frac{dIL1b_{p}}{dt}=a_{il1b_{p}}\frac{NFkB_{n}{}^{\gamma_{\text{nfkb}}}}{H_{\text{nfkb}}^{\gamma_{\text{nfkb}}}+NFkB_{n}{}^{\gamma_{\text{nfkb}}}}-a_{il1b_{c}}\frac{C_{1}^{\gamma_{c1}}}{H_{c1}^{\gamma_{c1}}+C_{1}^{\gamma_{c1}}}IL1b_{p}-d_{il}IL1b_{p}.$$

Cytoplasmic interleukin 1β, *IL*1*b*_*c*_, can also decay in the same manner as the pro-form. Additionally, *IL*1*b*_*c*_, can transport out of the cell via pores formed by gasdermin on the cell surface, at the rate $k_{il1b_{cte}}$. That is, *IL*1*b*_*c*_, evolves via:

(50) $$\frac{dIL1b_{c}}{dt}=a_{il1b_{c}}\frac{C_{1}^{\gamma_{c1}}}{H_{c1}^{\gamma_{c1}}+C_{1}^{\gamma_{c1}}}IL1b_{p}-k_{il1b_{cte}}\text{GSDM}D_{n}IL1b_{c}-d_{il}IL1b_{c}.$$

External interleukin 1β levels only depend on transport out of the cell in the ODE model,

(51) $$\frac{dIL1b_{e}}{dt}=k_{il1b_{cte}}\text{GSDM}D_{n}\;IL1b_{c}.$$

We only consider the level of cytoplasmic interleukin 18, *IL*18_*c*_, which is cleaved from its pro-form by caspase 1 and transports out of the cell via gasdermin pores, in the same way as interleukin 1β. That is, the evolution can be described by:

(52) $$\frac{dIL18_{c}}{dt}=a_{il18}\frac{C_{1}^{\gamma_{c1}}}{\left(H_{c1}^{\gamma_{c1}}+C_{1}^{\gamma_{c1}}\right)}\left(1-IL18_{c}-IL18_{e}\right)-k_{il18_{cte}}\text{GSDM}D_{n}\;IL18_{c}.$$

External interleukin 18 levels only depend upon transport out of the cell in the ODE model,

(53) $$\frac{dIL18_{e}}{dt}=k_{il18_{cte}}\text{GSDM}D_{n}\;IL18_{c}.$$

Finally, once gasdermin pores form on the cell surface external material can enter the cell causing the cell to swell. Therefore, we allow the cell volume, *V*, to increase through the equation:

(54) $$\frac{dV}{dt}=k_{vol_{c}}\text{GSDM}D_{n}\;V.$$

Once the cell volume reaches 1.5 times the original value, then the cell bursts and all cellular processes cease.

The cytokines released by the cell, IL-1β and IL-18 are then modelled in the epithelial environment. We allow IL-1β to potentially initiate pyroptosis in nearby epithelial cells, through a bystander effect^156–158^. Whereas, IL-18 acts as a chemoattractant for immune cells, directing them to the local environment^156,157,159–161^. Both cytokines are modelled as a diffusible field.

###### Estimation for pyroptosis parameters

NLRP3 has a half-life of approximately 6hrs, therefore, we choose a decay rate *d*_*nlrp*3_=0.002 min^−1 162^. Pro-IL-1β has a half-life of approximately 2.5 hrs, therefore we choose a decay rate *d*_*il*_=0.004 min^−1 163^.

For the remaining parameters, we use data from three experimental studies Bagaev et. al.^164^, de Vasconcelos et. al.^165^, Martín-Sánchez et. al.^166^ to estimate a timeline for the events regarded in our mathematical model.

Bagaev et. al. reported that the nuclear NF-κB concentration peaked at 10 minutes post-activation, after which it decreased to a half-maximal level in a gradual manner over 100 minutes. de Vasconcelos et. al^165^ indicate that following pyroptosis, cell volume increased to around 1.5 times the original cell volume, gradually for approximately 13 minutes prior to membrane rupture. Furthermore, their results suggest that the time between pyroptosis beginning and complete membrane rupture is approximately 2 hours. Additionally, we can estimate from this data that the NLRP3 inflammasome base is formed somewhere between 56 to 30 minutes prior to cell rupture. In the model, we use the average value (43 minutes) as a time-point for when the inflammasome starts forming (i.e.when the concentration of *NLRP*3_*b*_ reaches the threshold value).

In Martín-Sánchez et. al.^166^ their results highlighted that the release of IL-1β coincided with membrane permeability (pores forming), and eventually all of the pro-IL-1β present at the start of the experiments was cleaved and released from the cell, with approximately 90% released within 120 minutes. Therefore, we fit our parameters to result in a large proportion of cytokines to be released from the cell following pyroptosis.

#### Lymph node model

The DCs, migrated to the lymph node, are responsible for the development of adaptive immune response in a broad spectrum, where T lymphocytes contribute a major area. In parallel with the CD8^+^ cytotoxic T-cells mediated infected cell-killing, the CD4^+^ helper T-cells functions in two different domains. The type I helper T-cells (Th1) deal with the inflammation and cellular immune response, where type II helper T-cells (Th2) provide help to B-cells in antibody production. In the current version of this study, we do not include the B-cell activation and antibody response, but mostly the activation and proliferation of CD8^+^ and CD4^+^ T-cells are focused. The migrated DCs present the antigen to the precursor/naive T-cells while producing the primary inflammatory cytokines^131^. Stimulated by the antigen presenting cells (DCs) and corresponding to the ambient cytokines environment, the naïve T-cells start differentiate into two major Th1 and Th2 effector cells. During this time period, previously activated memory helper T-cells also recirculate and start to proliferate.

The mutual regulations of Th1 and Th2 are mediated by the secreted pro- and anti-inflammatory cytokines, although the effects of the cytokines in the current lymph node submodel have been substituted in terms of effective Th1 and Th2 cells. Th1 and Th2 cells have autoregulatory proliferations, where Th2 represses the Th1 cells proliferation. Th1 activation and clearance are regulated indirectly by DCs and directly by the cytokines from both Th1 and Th2, where Th2 clears naturally.

The time course of arrival of the dendritic cells to the LN can be presented as

(55) $$\frac{dD_{M}}{dt}=k_{D}D\left(t-\tau_{D}\right)-\delta_{D_{M}}D_{M}$$

where, *D* presents the number of dendritic cells in the tissue and *D*_*M*_ is that migrated into the LN. *k*_*D*_ and *τ*_*D*_ are the antigen presentation rate by dendritic cells and time taken by the DCs to migrate into the LN. The natural death rate of the *D*_*M*_ is denoted as $\delta_{D_{M}}$.

In the LN, the proliferation, activation and clearance of the two types of helper T-cells (*T*_*H1*_ and *T*_*H2*_) and cytotoxic T-cells (T_C_) are demonstrated in the following ODEs

(56) $$\frac{dT_{H1}}{dt}=\sigma_{T_{H1}}\frac{T_{H1}}{{\left(1+T_{H2}\right)}^{2}}+\pi_{T_{H1}}\frac{D_{M}T_{H1}{}^{2}}{{\left(1+T_{H2}\right)}^{2}}-\delta_{T_{H1}}\frac{D_{M}T_{H1}{}^{3}}{\left(1+T_{H2}\right)}-\mu_{T_{H}}T_{H1}$$

(57) $$\frac{dT_{H2}}{dt}=\sigma_{T_{H2}}\frac{T_{H2}}{\left(1+T_{H2}\right)}+\pi_{T_{H2}}\frac{\left(\rho +T_{H1}\right)}{\left(1+T_{H2}\right)}\frac{D_{M}T_{H2}{}^{2}}{\left(1+T_{H1}+T_{H2}\right)}-\mu_{T_{H}}T_{H2}$$

(58) $$\frac{dT_{C}}{dt}=\rho_{T_{1}}\frac{D_{M}T_{C}}{\left(\rho_{T_{1}}+D_{M}\right)}-\delta_{T_{1}}\frac{D_{M}T_{C}}{\left(\delta_{T_{1}}+D_{M}\right)}-\delta_{C}T_{C}$$

where, the $\sigma_{T_{H}}$ ’s and $\pi_{T_{H}}$ ’s are the proliferation and activation rate constants for type 1 and type 2 helper T-cells, respectively. $\delta_{T_{H1}}$ represents the DC-mediated deactivation of type 1 helper T-cells, while both have the same natural death rate of $\mu_{T_{H}}$. The TCs/CD8^+^ T-cells are simultaneously become activated as well as cleared by the DCs by two TC population-dependent rates with rate constants $\rho_{T_{1}}$, $\rho_{T_{2}}$ and $\delta_{T_{1}}$, $\delta_{T_{2}}$. As it currently stands all time delays are set to zero until functionality is built in to the solver.

#### Tissue fibrosis model

The accumulation of fibroblasts in the damaged site and excess deposition of fibrillar collagen leads to fibrosis during tissue regeneration. Sites of alveolar epithelial cell death activate latent anti-inflammatory cytokine (TGF-β), which recruites the fibroblasts^167,168^. We assume that the anti-inflammatory cytokine is activated and secreted continuously at the location of dead epithelial cells killed by CD8^+^ T cells. Fibroblasts chemotax towards the gradient of anti-inflammatory cytokines and deposit collagen. The pathological deposition of collagen can lead to acute or chronic fibrosis. The fibroblast recruitment is mediated by anti-inflammatory cytokine and a correlation for the dependence is adopted from experimental observation^169,170^ described by

(59) $$F_{g}\left(T_{\beta }\right)=0.0492T_{\beta }^{3}-0.9868T_{\beta }^{2}+6.5408T_{\beta }+7.1092$$

Where, *T*_*β*_ is the concentration of the anti-inflammatory cytokines and *F*_*g*_(*T*_*β*_) is the recruitment signal for fibroblast depending on the concentration of the anti-inflammatory cytokines. We replace ρ_cytokine_ in Eq. (29) with *F*_*g*_(*T*_*β*_) and set minimum recruitment signal ρ_min_ to 7.1092 (baseline value) and ρ_max_ to 12. The value of ρ_max_ was selected based on the experimental observation of fibroblast density during myocardial infarction^167^.

##### Estimates for fibrosis parameters

Fibroblast recruitment occurs during the tissue regeneration phase. So, we select a simulation condition where tissue is not completely destroyed after initial infection. We set the condition at faster T cell recruitment, faster T cell kill rate, and MOI = 0.01 (same as v3 Fig. 3.3).

The diffusion coefficient for anti-inflammatory cytokine is set at 555.56 μm^2^/min (same as pro-inflammatory cytokine), and we assume that collagen is non-diffusive. Decay rate for anti-inflammatory cytokine is set to 1.04 × 10^−2^ min^−1 171^. We vary the secretion rate of anti-inflammatory cytokine (1 min^−1^ and 15 min^−1^) to observe the change in the concentration of collagen deposition (Fig. 4.4 – supplemental 1).

Fibroblasts have an average diameter of 13 μm^172^, and the volume of the fibroblast nucleus is assumed to be 10% of total cell volume. The migration rate of fibroblast along anti-inflammatory cytokine gradient is 1 μm/min^172,173^. Fibroblasts undergo apoptosis at a rate of 8.3 × 10^−5^ min^−1 174^. The collagen deposition rate of fibroblast is 0.014 μg/cell/min^170,175^.

##### Initialization

An initial population of *MPhi*_0_ naïve macrophages and *DC*_0_ naïve dendritic cells are seeded randomly throughout the tissue.

#### Other implementation notes

This simplified immune model does not yet include many key immune agents, including natural killer (NK) cells, B cells, antibodies, the complement system, and most cytokines. No anti-inflammatory cytokines are modeled, nor can this model return to homeostasis following potential infection clearance. Dynamics of cytokine binding and unbinding to receptors are also omitted. The model does not yet incorporate known SARS-CoV-2 immune evasion techniques, such as a delayed IFN-I response and lymphopenia (decreased CD8^+^ T cells) from early in infection. In addition, the antigen presentation and subsequent T cell activation in the lymph node is not yet explicitly modeled. Many of these important mechanisms are planned for inclusion in future versions. See further discussion in the modeling results below.

#### Software release

The core model associated with the v4 prototype is Version 0.4.0. The nanoHUB app associated with the v4 prototype is Version 4.3. GitHub releases and Zenodo snapshots are given in the Appendix.

The cloud-hosted interactive model can be run at https://nanohub.org/tools/pc4COVID-19.

#### Model behavior: what does the current version teach us?

Except as noted below, all simulation results use the v4 model default parameters, which are supplied in the XML configuratiozǹ parameter file of the version 0.4.0 core model repository.

##### Overview of v4 model results with base parameters

Initial simulations with all the added model features used the base parameters described above, and the results are shown in Figure 4.4. At time point 0 (Fig 4.4A) resident macrophages and DC can be seen. Following the introduction of virus, the evolution of the infection over time is shown in Figure 4.4B–D. The simulations demonstrate good survival of the tissue in the early time points (2.5 days Fig 4.4B), but survival is not maintained at later time points due to CD8 cells arriving *en masse* to kill the remaining infected cells (10 days, Figure 4.4D). The kinetics of collagen deposition (Fig 4.4E) follows the time course of tissue damage and rises rapidly after 8 days. Interferon (IFN) production (Fig 4.4F) occurs very rapidly and is maintained for 8 days, until cell death increases. Fig 4.4G and H show dynamics of the DC and T cell populations in the LN. It can be seen that there is a constant arrival of DC into the LN starting around day 1 and this is maintained throughout the course of the infection (Fig 4.4G). CD8 cells appear to rise exponentially after day 5 (Fig 4.4G) as do Th2 cells (Fig 4.4H), whereas there appears to be very little Th1 response (Fig 4.4H).

##### Impact of interferon signaling

It was clear from these simulations that the base set of parameters was not sufficient for longer-term tissue survival and recovery, and that there was an over exuberant CD8 T cells response and that the IFN response was not optimal. As a first step the parameters controlling the IFN response were varied. Three parameters were examined: 1) the maximum IFN secretion level (*r*_*IFN infection secretion*_); 2) the relative paracrine secretion, which reflects the ability of uninfected cells to secrete IFN (*r*_*maxparacrine*_); and 3) the maximum inhibition of viral protein synthesis by IFN (*r*_*INF activation*_).

**Figure 4.5:**
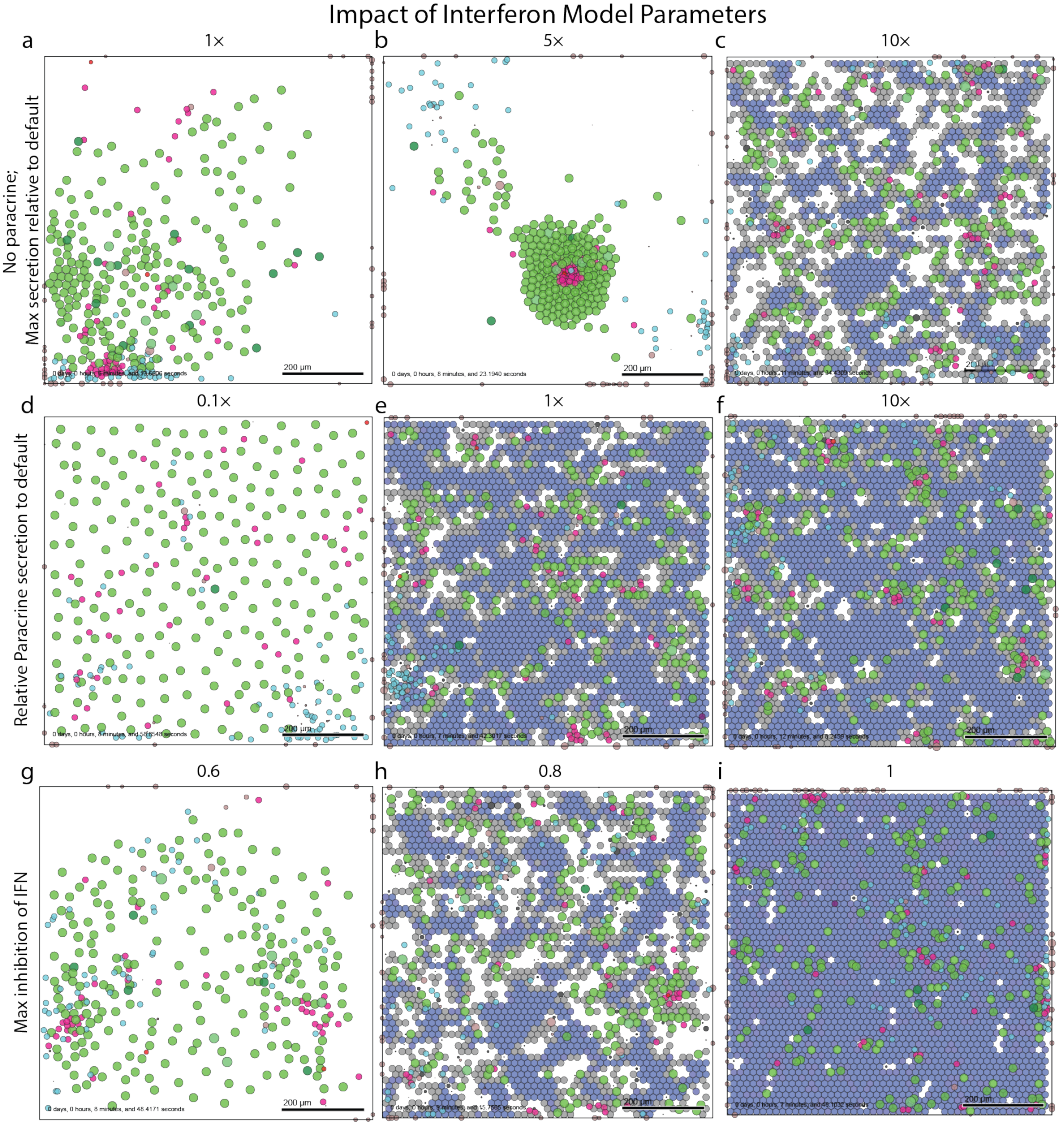
COVID-19 parameter simulations with current base parameters. (**a-d**) Still images of time points of the COVID simulation at 0(a), 2.5(b), 7.5(c), and 10(d) days. (**e**) Corresponding time plots of Collagen across the simulated 10 days. Low Collagen is present in the system until post 6 days when mass cell death starts to initiate leading to a buildup of collagen. (**f**) Corresponding time plots of interferon (IFN) across 10 days. IFN count rapidly increases in the first day after infection was detected and maintains at a high level through the simulated 10 days. (**g-h**) Lymph node plots showing DC, Tc, Th1, and Th2 counts contained within the lymph node.

The maximum IFN secretion rate was examined in the scenario where the paracrine secretion was set to zero (Fig 4.5A–C). In the absence of the paracrine effect and with the default IFN secretion parameter all cells are dead by Day 6 (Fig 4.5A). Increasing the secretion rate 5-fold has little effect (Fig 4.5B) but a 10-fold increase results in better survival (Fig 4.5C). Addition of the paracrine effect to the base IFN secretion rate (Fig 4.5E) significantly improves survival. This is marginally improved if the paracrine effect is increased 10-fold (Fig 4.5F), whereas a 10-fold reduction of the base paracrine effect results in a loss of all tissue (Fig 4.5D). Varying the IFN-induced inhibition of viral protein synthesis also has a dramatic effect on tissue survival (Fig 4.5G–I). The best survival is seen when IFN can inhibit 100% of protein synthesis (Fig 4.5I). Reduction to 80% reduces survival somewhat (Fig 4.5 H), and no tissue survives if this value is reduced to 60% (Fig 4.5G).

**Figure 4.6:**
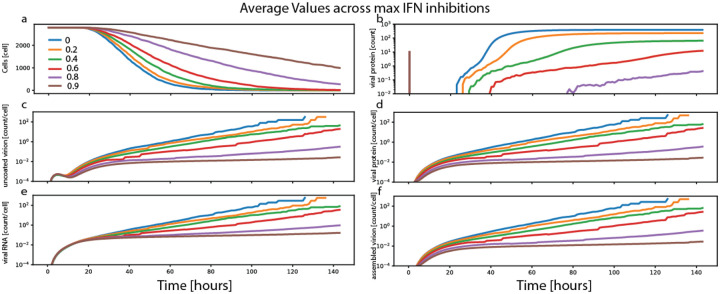
Time plots of various IFN max inhibition values across 6 days. (a) amount of living epithelial cells (b)external viral protein. (c-f) cellular load plots normalized against the living number of cells to generate a running average value. (c) uncoated virion (d)viral protein (e) viral RNA (f) assembled virion.

The impact of varying the protein synthesis by IFN on cell survival and viral dynamics was also examined (Fig 4.6A–F). From these plots the inhibition of protein synthesis by IFN has to be at least 80% in order to have any significant impact on viral production or cell survival (Fig 4.6A–F). High IFN action sees near 0, or even negative concentrations of extracellular virion (a result of our uptake assumption of only full virions). Rolling average of cellular infectivity shows the cells in the current model do not recover (Fig 4.6C–F), the system seems to only recover after mass cell death.

##### Further exploration dendritic cell and CD8 T cell dynamics

**Figure 4.7:**
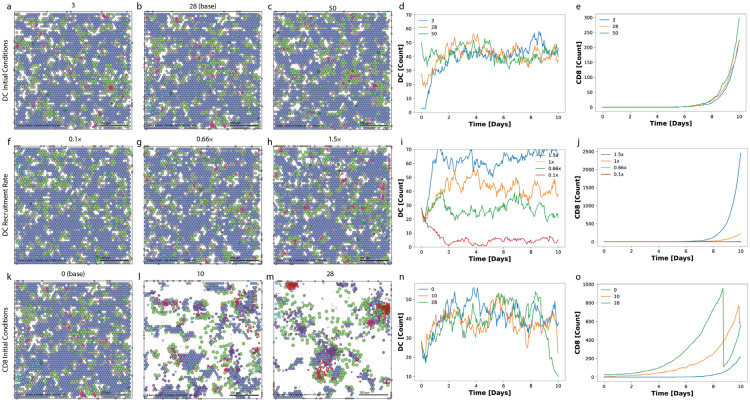
Multiple variations of immune cell recruitment rates. (a-c) DC initial condition still images at 6 days are shown for 3 (a), 28 (b; base parameter value), and 50 (c) DCs. Plots of DC counts (d) and CD8^+^ T Cells (e). (f-h) DC recruitment rate still images at 6 days are shown for 0.1x (f), 0.66x (g), and 1.5x (h) the default recruitment rate. Plots of DC counts (i) and CD8^+^ T Cells (j) at the different recruitment rates. (k-m) CD8^+^ T cell initial condition still images at 6 days are shown for 0 (k; base parameter value), 10 (l), and 28 (m) CD8^+^ T cells. Plots of DC counts (n) and CD8^+^ T cells (o) for the different CD8 T cell initial conditions.

The parameters controlling the DC and CD8 T cell dynamics were also examined with the aim of identifying factors that contribute to the massive influx of CD8 T cells late in the infection. These included: 1) the initial number of DCs in the tissue; 2) the rate of recruitment of DCs to the tissue; 3) the presence of tissue resident CD8 T cells prior to infection. Changing the number of DCs present in the tissue from 3 – 50 (Fig 4.7A–C) had little impact on the infection. As seen in Fig 4.7D, the number of DCs in the tissue rapidly converged to a constant level, and this did not impact the number of CD8 T cells recruited to the tissue (Fig 4.7E). In contrast, varying the DC recruitment rate did not appear to affect tissue damage (Fig 4.7F–H) but had a major impact on DC numbers (Fig 4.7I) and CD8 T cell expansion (Fig 4.7J).

The introduction of tissue resident CD8 T cells at the start of the infection (Fig. 4.7K–M) resulted in increased tissue damage, presumably due to the presence of CD8 cells killing infected cells. The number of DCs in the tissue was not really affected by preexisting CD8 T cells (Fig 4.7N) except at the later time points, and a similar trend was seen with the CD8 T cell count (Fig 4.7O). It appears that in these scenarios, the infection spreads faster than the CD8 cell killing, which leads to large areas of destroyed tissue. Thus, it appears from these simulations that varying the DC recruitment rate has the most impact on controlling CD8 T cell expansion.

#### Discussion of current model version

This version of the model has included many new components, including more detailed models of viral replication, the introduction of the type 1 IFN response and a LN model that includes DC migration and activation of CD4 and CD8 T cells. In addition, different macrophage activation states, a pyroptosis death model and the generation of fibrosis in response to tissue damage. The addition of these components has introduced many parameters that creates challenges in calibrating the model.

The inclusion of a more realistic viral replication model has created an infection that spreads very rapidly when compared to previous versions. This has resulted in an inability to clear virus under even the most favorable immune response conditions. Under the present conditions, new infected cells always appear and these cells are killed due to infection or by CD8 T cells and macrophages that are recruited to the tissue *en masse*. There are multiple approaches to address this issue. For one, better slowing viral spread through free parameters (i.e., viral uptake at the current state is underdeveloped which may be causing rapid viral uptake). Even initial viral seeding could be a solution: at present virus is introduced at the start of the simulation randomly across the whole tissue at an MOI of 0.1. This results in widespread virus seeding, which then spreads rapidly before the immune response begins. A more realistic approach may be to seed the virus in a discrete local area, and this will be explored in the next version of this model.

Another concern has been the rapid and exponential expansion of CD8 T cells, which then leads to excessive killing of infected cells. This is driven, in part, by the fact that under the present conditions, DCs continuously migrate to the LN to stimulate CD8 T cells. This could be remedied by tempering the infection as described above. Once the infection is cleared, DCs no longer migrate to the LN and the response is controlled. Data from influenza infections may be used to calibrate this aspect of the model^176^.

In addition, some changes to the LN model might be considered. It is known that Th1 cells are necessary for optimal CD8 responses and the present model does not include this requirement. The model could be refined by adding the requirement that Th1 cells are necessary for the proliferation and expansion of CD8 T cells. In this scenario, activated DCs carrying viral antigen migrate to the LN where they activate CD8 T cells and induce the expansion of Th1 and Th2 cells. Th1 cells interact with CD8 T cells to induce their proliferation and expansion. Th1 and Th2 cells are mutually inhibitory^177^, but in the present model the inhibition of Th2 cells by Th1 cells is not included, which results in the domination of the Th2 response. The Th2 response is necessary for optimal antibody production and thus it will be important to model this accurately. Recent data suggest that patients with severe COVID-19 disease had robust but delayed IgG anti-spike antibody responses^178^. Thus, the balance and timing between the Th1 and Th2 responses may be critical in determining whether the disease is mild or severe.

#### Priorities for Version 5

After extensive discussion of the Version 4 model results, the coalition set priorities for Version 5 development. First, it was recognized the cell killing by neutrophils can have a “bystander” killing effect: reactive oxygen species (ROSs) created and released by neutrophils and macrophages may kill nearby cells^179–181^. This could have multiple effects on the viral plaques: increased killing could accelerate tissue damage, but killing nearby infected cells could prevent them from successfully replicating and releasing virus. Thus, localized bystander killing could potentially have a protective role as well. Given the importance of antibodies to COVID-19 clinical care (via monoclonal antibody treatments^179^, convalescent plasma^182^, or the vaccine-driven immunity^183^), the next release will include a model for production, transport, and function of antibodies. The coalition also recognized that as a viral infection is brought under control, negative regulators must initiate an anti-inflammatory response to prevent sustained tissue damage; thus, Version 5 is expected to include anti-inflammatory responses.

## Discussion

Within three weeks of the World Health Organization’s declaration of a global pandemic of COVID-19^184^, community-based prototyping built upon an existing PhysiCell 3D cell-modeling framework to rapidly develop Version 1 of an intracellular and tissue-level model of SARS-CoV-2^109^. A growing coalition of domain experts from across STEM fields are working together to ensure accuracy and utility of this agent-based model of intracellular, extracellular, and multicellular SARS-CoV-2 infection dynamics. Version 1 development underscored the necessity of clearly explaining model components, defining scope, and communicating progress as it occurs for invaluable real-time feedback from collaborators and the broader community. This rapid prototyping already helped in growing the coalition and recruiting complementary expertise; for instance, a team modeling lymph node dynamics and immune infiltration joined during the Version 1 cycle after seeing initial progress.

The version 1 prototype also showed the scientific benefit of rapid prototyping: even a basic coupling between extracellular virion transport, intracellular replication dynamics, and viral response (apoptosis) showed the direct relationship between the extracellular virion transport rate and the spread of infection in a tissue. More importantly, it showed that for viruses that rapidly create and exocytose new virions, release of additional assembled virions at the time of cell death does not significantly speed the spread of infection. Moreover, decreasing the cell tolerance to viral load does not drastically change the rate at which the infection spreads, but it does accelerate the rate of tissue damage and loss, which could potentially trigger edema and ARDS earlier. This suggests that working to slow apoptosis may help preserve tissue integrity and delay adverse severe respiratory responses. That such a simple model could already point to actionable hypotheses for experimental and clinical investigations points to the value of rapid model iteration and investigation, rather than waiting for a “perfect” model that incorporates all processes with mechanistic molecular-scale detail.

Version 2 showed promise of increasing mechanistic details to evaluate potential inhibitors. For example, it was found that that reducing the expression of ACE2 receptors could paradoxically lead to faster spread of the infection across the tissue, although the individual infected cells would replicate virus more slowly. On the other hand, taking advantage of high receptor expression but interfering with viral release from internalized receptors may help slow infectious dynamics. Generally, adding sufficient actionable cell mechanisms to the model framework allows us to ask pharmacologically-driven questions on potential pharmacologic interventions, and how these findings are affected by heterogeneity, stochasticity, and the multiscale interactions in the simulated tissue.

Version 3 allowed our first investigations of immune system responses. We found that T cell behaviors are critical to controlling the spread of an infection through the tissue. In particular, rapid recruitment as well as the presence of “educated” CD8^+^ T cells prior to infection (e.g., after responding to infection in a nearby tissue) had a significant protective effect, even in the current model that does not explicitly model antibodies. This is consistent with emerging studies that link T cell responses to patients with the best recovery^185–187^.

Version 4 added multiscale interaction with the lymphatic system, particularly to allow dendritic cells to present antigens to T cells to drive expansion and immune response, and trafficking of dendritic cells and T cells between the local, spatially resolved tissue and the lymphatic system. It also introduced interferon responses, which were found to have a profound impact on the spread of viral plaques. Refined models of infected cell death (pyroptosis) and tissue damage (fibrosis) were also introduced.

As work on future versions progresses, teams will work in parallel on submodels to add, parameterize, and test new model components. It will be important to balance the need for new functionality with the requirement for constrained scope, while also balancing the importance of model validation with timely dissemination of results. Thus, this preprint will be updated with every development cycle to invite feedback and community contributions.

As of April 2021, the coalition anticipates that Version 5 will be the last version to introduce significant new features, with a focus on antibody responses, anti-inflammatory responses, bystander cell killing due to reactive oxygen species, and other refinements to the immune system model. We anticipate that Version 6 will transition us to Phase III (widespread community use), with a focus on code hardening, documentation, training materials, and improved parameter estimates based on community-wide data sharing.

### Getting involved

To get involved, we welcome biological expertise, especially related to model assumptions, hypotheses, infection dynamics, and interpretation of results. Mathematical contributions to the underlying model or model analysis and data contributions for crafting, parameterizing, and validating model predictions are particularly sought.

We encourage the community to test the web-hosted hosted model at https://nanohub.org/tools/pc4COVID-19. This model will be frequently updated to reflect progress, allowing the public to take advantage of this rapid prototyping effort.

We avidly encourage the community to test the model, offer feedback, and join our growing coalition via Google survey (https://forms.gle/12vmLR7aiMTHoD5YA), by direct messaging Paul Macklin on Twitter (@MathCancer), or by joining the pc4COVID-19 Slack workspace (invitation link). Updates will frequently be disseminated on social media by Paul Macklin (@MathCancer), the PhysiCell project (@PhysiCell), the Society for Mathematical Biology subgroup for Immunobiology and Infection Subgroup (@smb_imin), and others.

We also encourage developers to watch the pc4COVID-19 GitHub organization and to contribute bug reports and software patches to the corresponding (sub)model repositories. See https://github.com/pc4COVID-19

We are encouraged by the fast recognition of the computational and infectious disease communities that we can make rapid progress against COVID-19 if we pool our expertise and resources. Together, we can make a difference in understanding viral dynamics and suggesting treatment strategies to slow infection, improve immune response, and minimize or prevent adverse immune responses. We note that this work will not only help us address SARS-CoV-2 but will also provide a framework for readiness for future emerging pathogens.

## Supplementary Material

Supplement 1

## Figures and Tables

**Fig. 4.1:**
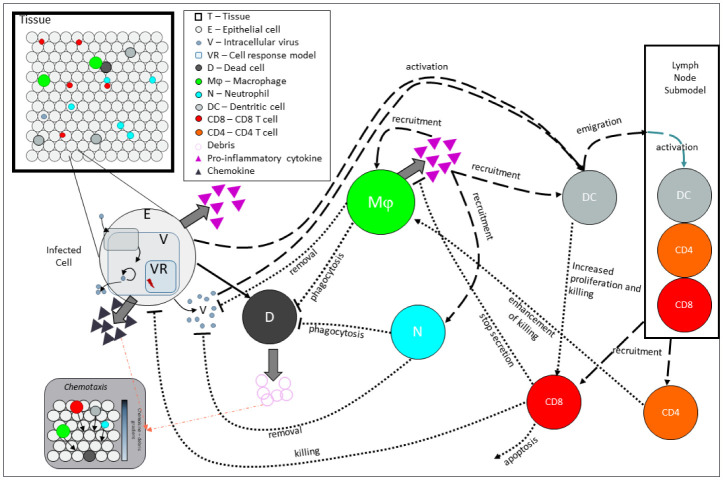
Immune submodel schematic. Immune cells (macrophages, neutrophils, dendritic cells, CD8^+^ T cells, and CD4^+^ T cells) patrol within the *tissue component* (T), containing multiple *epithelial cells* (E). Cells infected by virus secrete chemokine, which attracts immune cells along the chemokine gradient. CD8^+^ T cells induce apoptosis in infected cells, creating dead cells that are phagocytosed by macrophages and neutrophils that are attracted along debris gradients. Upon activation, macrophages secrete pro-inflammatory cytokine that recruits other immune cell types. Dendritic cells emigrate to present viral antigen and activate T cells.

**Fig. 4.2:**
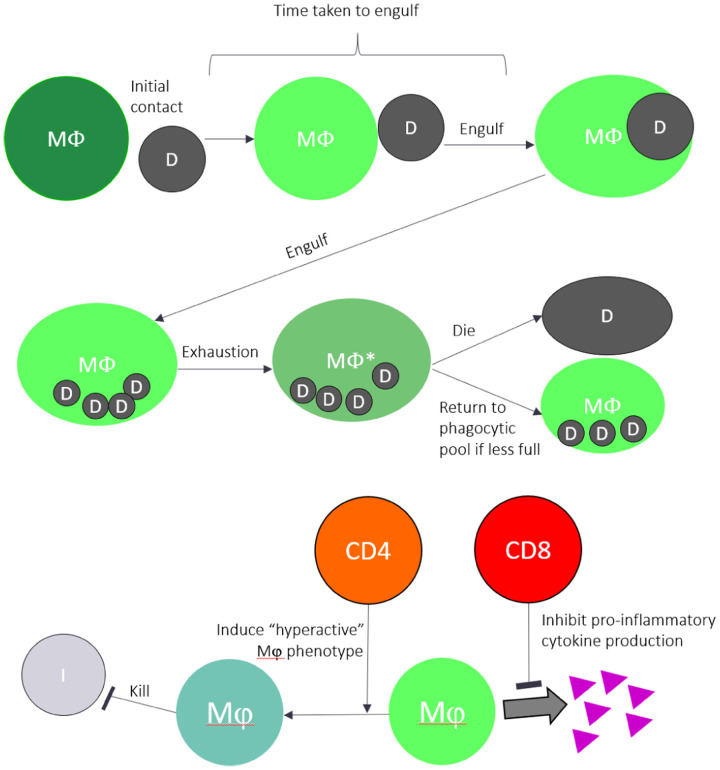
Macrophage Modulation. Macrophages engulf dead cell material and grow in size. Too much internalized debris can induce macrophage exhaustion, which causes death or a pause in new phagocytosis. Macrophages that previously engulfed ma-

**Fig. 4.1 - supplemental 1:**
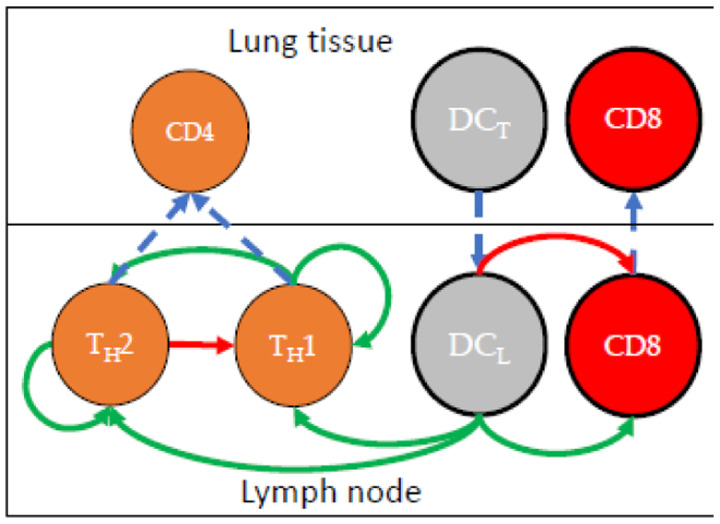
Lymph node model. Once in the lymph node DCs work to recruit CD4 and CD8 cells to the tissue. Green and red arrows in the figure represent activation and repression. The dotted blue arrows are migration across the tissue barrier.

**Figure 4.4:**
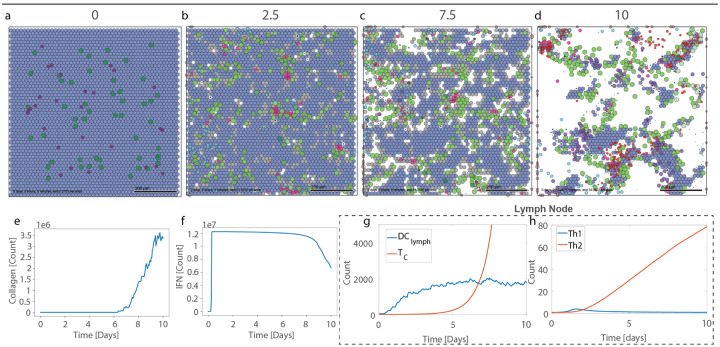
COVID-19 parameter simulations with current base parameters. (**a-d**) Multiple still images of time points of the COVID simulation at 0(a), 2.5(b), 7.5(c), and 10(d) days. (**e**) Corresponding time plots of Collagen across the simulated 10 days. Low Collagen is present in the system until post 6 days when mass cell death starts to initiate leading to a buildup of collagen. (**f**) Corresponding time plots of interferon (IFN) across 10 days. IFN count rapidly increases in the first day after infection was detected and maintains at a high level through the simulated 10 days. (**g-h**) Lymph node plots showing DC, Tc, Th1, and Th2 counts within the lymph node.

**Fig.4.4 - supplemental 1:**
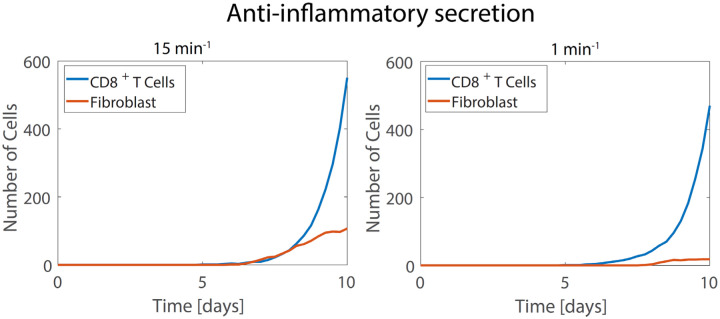
Dynamics of collagen deposition, CD8+ T cells, and fibroblast at anti-inflammatory cytokine secretion rate 15 min^−1^ (Left), and 1 min^−1^ (Right).

## Box 1: Guiding principles for the rapid prototyping a modular, multiscale model.

### Guiding principles: motivation

- The model should investigate the dynamics of infection and treatment, not merely endpoints.
- The model should help the community ask “what if” questions to guide experiments and interventions^9,10^.

### Guiding principles: approach

- Community consensus will be gathered and efforts pooled into a “standardized” model that captures key SARS-CoV-2 dynamics. The model will be supplied to the community for parallel studies by multiple labs.
- The model framework will be built with relatively sparse data, relying upon domain expertise and observations to choose its general form and assess its qualitative behavior.
- The model will be modular. Each submodel will have well-defined inputs and outputs, allowing parallel development and replacement of submodels with improved versions without change to the overall integrated model or other submodels.
- As part of the model formulation, documentation, and dissemination, we will craft clearly delineated “conceptual model and hypotheses” to encourage development of independent models with distinct methodologies and software frameworks.
- The submodels will be independently executable and verifiable, allowing parallel development.
- The overall model framework will be released periodically released numbered versions (distributions) that bundle the best working version of each submodel as it exists at the time of release, allowing end-users (the community) to use well-defined, well-tested snapshots of the project.
- The model (and known parameter values) will be made publicly available as open source for maximum public benefit.
- The model will be made publicly available as an interactive web app to encourage community participation, to accelerate scientific dissemination, and to increase public educational benefit.
- Rapid prototyping will be used to encourage an iterative develop-test-refine cycle, build expertise, and gain community feedback.
- Data and parameter sharing is encouraged throughout this effort for use following the model’s “completion.”
- The model will be developed to a point that it has correct qualitative behavior so that calibration is likely to succeed. This is the “product” for use in subsequent investigations by multiple teams. See the ***scoping*** statements above.
- After the model prototyping is complete (the goal of this paper), a maintenance and support phase will be entered to fix bugs, support scientist users, and add features identified by the user community.

## Box 2: Community-selected scientific questions driving the model’s development.

1. What are the critical “choke points” in viral infection, replication, and propagation?
2. Which interventions could most effectively leverage identified vulnerabilities in viral replication?
3. What unanticipated dynamics can emerge from a single molecular-scale inhibition?
4. Does the initial level of exposure to the virus affect the severity of the progression of the disease and how could this be ameliorated?
5. What are the key points of virus-immune interactions that drive mild versus severe (e.g., ARDS) responses?
6. What are key differences at the target cell level during innate versus adaptive immune responses?
7. Are there threshold levels of infection at the cellular or tissue level that indicate a switch from asymptomatic to symptomatic or from mild to severe disease in a patient?
8. Through what mechanisms do certain patient characteristics, pre-existing conditions, or background medications increase the likelihood of adverse outcomes?
9. What interventions could accelerate immunity?
10. What interventions can reduce or reverse adverse immune reactions?
11. At what stage is an intervention most beneficial?
12. How does viral mutagenicity affect the robustness of a therapy or a therapeutic protocol?
13. How does cellular heterogeneity affect infection dynamics?
14. How does the nearby tissue environment, such as the mucus layer, affect infection dynamics?
15. How does the infection spread from its initial locus of infection to other tissues (in particular, from upper respiratory tract to the bronchi, bronchioles, and alveoli within lungs)? How does stochasticity impact these dynamics?
16. How do tissues recover after clearance of local infection? Can scarring be minimized to reduce long-term adverse effects in recovered patients?
17. How do adverse effects in SARS-CoV-2 infected epithelia differ (mechanistically) from other infections and other modes of epithelial dysfunction?

## Box 3: Current subteams.

### Integration:

Coordinates overall project and leads multiscale model integration and dissemination.

*Chief scientist(s):* Paul Macklin, Michael Getz

*Technical contact(s):* Michael Getz, Randy Heiland, Yafei Wang

*Members:* Paul Macklin, Randy Heiland, Michael Getz, Yafei Wang

### Viral Replication:

Builds the submodel of viral replication (and release) within individual cells.

*Chief scientist(s):* James Faeder

*Technical contact(s):* Yafei Wang, Ali Sinan Saglam

*Members:* Jim Faeder, Yafei Wang, Paul Macklin, Ali Sinan Saglam, Caroline Larkin

### Infected cell response:

Builds the submodel of individual cell responses to infection, such as secretion of chemokines and apoptosis.

*Chief scientist(s):* Jason Shoemaker, James Glazier, Sara Hamis, Fiona Macfarlane

*Technical contact(s):* Jordan Weaver, Josua Aponte, Sara Hamis, Fiona Macfarlane

*Members:* Jason Shoemaker, Jim Faeder, Penny Morel, James Glazier, Ashok Prasad, Elsje Pienaar, Jordan Weaver, T.J. Sego, Josua Aponte, Yafei Wang, Sara Hamis, Fiona Macfarlan

### Pharmacodynamics:

Modifies the submodels to simulate pharmacologic interventions.

*Chief scientist(s):* Robert Stratford, Morgan Craig

*Technical contact(s):* Tarunendu Mapder, Yafei Wang, Sara Hamis, Fiona Macfarlane

*Members:* Robert Stratford, Morgan Craig, Sara Quinney, Mark AJ Chaplain, Tarunendu Mapder, Yafei Wang, Sara Hamis, Fiona Macfarlane, Richard F. Bergstrom

### Receptor trafficking:

Builds the submodel of ACE2 receptor trafficking, including virus binding and endocytosis.

*Chief scientist(s):* Padmini Rangamani

*Technical contact(s):* Andy Somogyi

*Members:* Padmini Rangamani, Andy Somogyi

### Tissue immune response:

Builds the submodels of individual immune cells and their interactions within an infected tissue.

*Chief scientist(s):* Morgan Craig, Courtney Davis, Amber Smith, Adrianne Jenner, Penny Morel

*Technical contact(s):* Adrianne Jenner

*Members:* Adrianne Jenner, Courtney Davis, Morgan Craig, Amber Smith, Penny Morel, Sofia Alfonso, Rosemary Aogo, Elsje Pienaar, Dennis Hou

### Lymph node:

Builds the submodel of immune cell expansion at nearby lymph nodes to drive immune cell recruitment.

*Chief scientist(s):* Gary An, Tarunendu Mapder

*Technical contact(s):* Tarunendu Mapder

*Members:* Gary An, Chase Cockrell, Marc-Andre Rousseau, James Glazier, T.J. Sego, Tarunendu Mapde, Juliano Ferrari Gianlupi

### Tissue damage:

Builds models of tissue damage (and potentially recovery).

*Chief scientist(s):* Ashlee Ford Versypt, Amber Smith

*Technical contact(s):* Mohammad Aminul Islam

*Members:* Amber Smith, Ashlee Ford Versypt, Thomas Hillen, Mohammad Aminul Islam

### Drug testing/experiment:

Explores drug inhibiting virus endocytosis and replication in cell culture

*Chief scientist(s)*: Aarthi Narayanan

*Technical contact(s):* Kenneth Risner

*Members:* Aarthi Narayanan, Kenneth Risner

### SBML integration:

Refines PhysiCell integration with libRoadrunner to support direct execution of SBML models

*Chief scientist(s):* Randy Heiland

*Technical contact(s):* Randy Heiland, Andy Somogyi

*Members:* Andy Somogyi, Randy Heiland, Furkan Kurtoglu, Pablo Maygrundter, Jim Faeder

### Visualization and analytics:

Refines standalone and integrated visualization and analytics for nanoHUB apps.

*Chief scientist(s):* Randy Heiland, Amber Smith, Courtney Davis

*Technical contact(s):* Randy Heiland, Dennis Hou

*Members:* Randy Heiland, Amber Smith, Courtney Davis , Hadi Taghvafard, Andy Somogyi, Furkan Kurtoglu, Pablo Mayrgundter, Dennis Hou

## Appendices

### Appendix 1: Code availability

All code is being made available as open source under the standard 3-Clause BSD license. Users should cite this preprint (or the final published paper, as the case may be).

#### Core model releases

##### Version 1 model

###### Version 0.1.0 (released March 26, 2020)

**GitHub:** https://github.com/pc4COVID-19/COVID-19/releases/tag/0.1.0

**Notes:** First release.

###### Version 0.1.1 (released March 26, 2020)

**GitHub:** https://github.com/pc4covid19/COVID19/releases/tag/0.1.1

**Notes:** Minor bugfixes and first inclusion of “math” directory.

###### Version 0.1.2 (released March 26, 2020)

**GitHub:** https://github.com/pc4covid19/COVID19/releases/tag/0.1.2

**Zenodo:** https://doi.org/10.5281/zenodo.3733336

**Notes:** First release with Zenodo integration. Last release in 0.1.x chain (v1 model chain).

###### Version 0.1.3 (released April 1, 2020)

**GitHub:** https://github.com/pc4covid19/COVID19/releases/tag/0.1.3

**Zenodo:** https://doi.org/10.5281/zenodo.3737166

**Notes:** First release after transferring the COVID-19 tissue-level model (overall model) from Paul Macklin’s per-sonal GitHub account to the new pc4COVID-19 GitHub organization.

##### Version 2 model

###### Version 0.2.0 (released April 9, 2020)

**GitHub:** https://github.com/pc4covid19/COVID19/releases/tag/0.2.0

**Zenodo:** https://doi.org/10.5281/zenodo.3747011

**Notes:** First v2 prototype. Introduced modular design and ACE2 receptor trafficking

###### Version 0.2.1 (released April 10, 2020)

**GitHub:** https://github.com/pc4covid19/COVID19/releases/tag/0.2.1

**Zenodo:** https://doi.org/10.5281/zenodo.3747011

**Notes:** Minor bugfixes for cell visualization.

##### Version 3 model

###### Version 0.3.0 (released July 3, 2020)

**GitHub:** https://github.com/pc4covid19/COVID19/releases/tag/0.3.0

**Zenodo:** http://doi.org/10.5281/zenodo.3929320

**Notes:** First v3 prototype. First integration of new immune submodel. Upgrade to PhysiCell Version 1.7.1, allowing use of XML-based cell definitions to define the behavior of immune cell types. Upgrade to PhysiCell Version 1.7.2 beta to improve multithreaded performance, add new cell-cell interaction features, and fix concurrency issues on some platforms.

###### Version 0.3.1 (released July 3, 2020)

**GitHub:** https://github.com/pc4covid19/COVID19/releases/tag/0.3.1

**Zenodo:** http://doi.org/10.5281/zenodo.3929726

**Notes:** This release improves parameter estimates for digestion of phagocytosed material and has an immune model refinement to prevent runaway macrophage death.

###### Version 0.3.2 (released July 15, 2020)

**GitHub:** https://github.com/pc4covid19/COVID19/releases/tag/0.3.2

**Zenodo:** https://dx.doi.org/10.5281/zenodo.3946820

**Notes:** This release simplifies the macrophage rules.

###### Version 0.4.0 (released Nov 20, 2020)

**GitHub:** https://github.com/pc4covid19/COVID19/releases/tag/0.4.0

**Zenodo:** https://dx.doi.org/10.5281/zenodo.4282875

Notes:

#### nanoHUB cloud-hosted model releases

The latest version can always be accessed directly at https://nanohub.org/tools/pc4COVID-19

##### Version 1 model

###### Version 1.0 (released March 26, 2020):

**GitHub:** https://github.com/rheiland/pc4COVID-19/releases/tag/v1.0

**Zenodo:** http://doi.org/10.5281/zenodo.3733276

**nanoHUB DOI:** http://dx.doi.org/doi:10.21981/19BB-HM69

**Notes:** First published version.

##### Version 2 model

###### Version 2.1 (released April 14, 2020):

**GitHub:** https://github.com/pc4covid19/pc4covid19/releases/tag/v2.1

**Zenodo:** http://doi.org/10.5281/zenodo.3766879

**nanoHUB DOI:** http://dx.doi.org/doi:10.21981/2B1H-GX51

**Notes:** Second published version. Moved GitHub repository to the pc4COVID-19 GitHub organization. Added another tab in the GUI for generating animation of cells (from SVG output files).

##### Version 3 model

###### Version 3.0 (released July 3, 2020):

**GitHub:** https://github.com/pc4covid19/pc4covid19/releases/tag/v3.0

**Zenodo:** http://doi.org/10.5281/zenodo.392953 9

**nanoHUB DOI:** http://dx.doi.org/doi:10.21981/V52J-0S03

**Notes:** The major change to the GUI in this release is the addition of a “Cell Types” tab. This allows editing parameters associated with <cell_definitions> in the configuration file. This version also includes a <style> block in the Jupyter notebook that fixed an unwanted scrollbar in the lengthy About tab.

###### Version 3.1 (released July 3, 2020):

**GitHub:** https://github.com/pc4covid19/pc4covid19/releases/tag/v3.1

**Zenodo:** http://doi.org/10.5281/zenodo.3929960

**nanoHUB DOI:** http://dx.doi.org/doi:10.21981/YNWZ-GE50

**Notes:** Minor updates to “About” text, e.g., explaining nature of stochastic results. Edits to immune_submodels.cpp (see details in the core model repository).

###### Version 3.2 (released July 21, 2020):

**GitHub:** https://github.com/pc4covid19/pc4covid19/releases/tag/v3.2

**Zenodo:** http://doi.org/10.5281/zenodo.3954019

**nanoHUB DOI:** http://dx.doi.org/doi:10.21981/843E-JE78

**Notes:** Update to use core model 0.3.2

##### Version 4 model

###### Version 4.0 (released November 20, 2020):

**GitHub:** https://github.com/pc4covid19/pc4covid19/releases/tag/v4.0

**Zenodo:** https://doi.org/10.5281/zenodo.4283005

**nanoHUB DOI:** http://dx.doi.org/doi:10.21981/8T5J-9G97

**Notes:** Update to use core model 0.4.0

###### Version 4.1 (released November 25, 2020):

**GitHub:** https://github.com/pc4covid19/pc4covid19/releases/tag/v4.1

**Zenodo:** https://doi.org/10.5281/zenodo.4290732

**nanoHUB DOI:** http://dx.doi.org/doi:10.21981/M5GC-6E79

**Notes:** Bug fix: Cell Types custom_data were not setting/getting values to/from XML.

###### Version 4.2 (released January 20, 2021):

**GitHub:** https://github.com/pc4covid19/pc4covid19/releases/tag/v4.2

**Zenodo:** https://doi.org/10.5281/zenodo.4453795

**nanoHUB DOI:** http://dx.doi.org/doi:10.21981/2WY0-SX97

**Notes:** Bug fix: Selectable colormaps and fixed ranges.

## Appendix 2: Organoid platform details

Aarthi Narayanan’s virology lab is optimizing SARS-CoV-2 cultures in organoid model systems. The viral replication kinetics will be assessed by infection of different lung epithelial, fibroblast and endothelial cells, in addition to standard cell lines such as Vero cells, which are one of the cell lines in use for inhibitor assessment studies. Primary cells and/or cell lines will be infected with SARS-CoV-2 at increasing multiplicities of infection (MOI) and infectious viral titers in the supernatants assessed by plaque assays at multiple time points post infection (pi). This will stretch from ~3 hours post infection (phi) to 48 hpi depending on the cell type and the MOI.

In parallel, the viral genomic copy numbers will be assessed in the same supernatant samples by qRT-PCR with virus specific primers. This will provide information on how the production of infectious virions compares with the number of genomic copies available outside the cell. If the numbers are skewed in the direction of genomic copies (which may happen in the context of some kinds of inhibitors), it will shed light on the mechanisms of inhibition involving inhibition of infectivity of progeny virions.

The viral genomic copy numbers inside the cells will also be assessed by qRT-PCR and compared to the genomic copies outside the cell. This will provide direction on the efficacy of particle packaging and the extent of production of infectious versus noninfectious virus. While it will not provide directly pertinent information about the possibility of heterogeneity of released virus populations and quasispecies, it can provide initial clues in that direction, which can then trigger more specific questions and relevant approaches. These approaches will be pursued for cell lines, primary cells and, hopefully, subsequently transitioned to organoid platforms.

From a host response point of view, we will pursue two aspects: host cell death and inflammatory responses. For cell survival and death measurements, we will employ an assay that measures ATP activity in cells (hence a reflection of a live cell) in the context of infection and inhibitor treatments. For inflammatory responses, we will assess supernatants for inflammatory mediators by ELISA (multiplexed). The cells will be lysed to obtain RNA, which will be queried for transcription of several genes associated with inflammatory responses using gene expression arrays (multiplexed).

Additional host response events will include mitochondrial activity and ROS production assessments in the context of infection and inhibitor treatments. The impact of anti-inflammatory strategies on mitochondrial activity and cell survival will be assessed to determine correlations between viral replication dependent and independent events.

## Appendix 3: Overall design cycle development details

In each prototyping or design cycle:

1. The core team sets priorities for the design iteration:
  - Discuss feedback and identify highest priority model refinements.
  - Collaborate to update the submodel design documents to address feedback.
  - Update the overall model design document as needed.
  - Assess new data to refine parameter estimates.
  - Refine submodel input/output formats as necessary.
  - Assess next release dates for the submodels.
2. Submodel teams meet to refine their code and put out their next releases. The chief scientists communicate releases to the overall leads.
3. The integration team integrates the latest submodel releases into a new release candidate for the overall model.
  - Address any bug reports.
  - Test new or altered functions.
  - Satisfy all qualitative and/or quantitative unit tests.
  - Qualitatively test the model for new or improved behaviors over the last iteration.
4. The integration team prepares a software release:
  - Update documentation.
  - Create a new numbered release on GitHub.
  - Update list of available validation data and best parameter estimates.
  - Create a Zenodo snapshot.
  - Announce on Twitter (via @PhysiCell, @MathCancer, and @SMB_imin).
5. The integration team updates the cloud-hosted model for multidisciplinary testing:
  - Update the nanoHUB app repository with new code.
  - Run xml2jupyter to update the Jupyter interface.
  - Update project on nanoHUB, test/refine until successful release.
  - Update documentation, numbered GitHub release, Zenodo snapshot of deployed model.
  - Perform live demos with domain experts and community to gather feedback.
6. The whole team seeks additional community feedback via Twitter and the pc4COVID-19 Slack workspace. The team integrates comments received from scientific peer review as appropriate.
7. The core team evaluates progress:
  - Distill feedback to assess the need for new model hypotheses, behaviors, or components.
  - Assess which biological behaviors are currently exhibited by the model.
  - Refine the design protocol (e.g., with refined model specification methods) as necessary.
  - Assess the need for an additional design iteration.
8. Update preprint for scientific dissemination. Return to Step 1 if there is substantial feedback, or if the core team determines that further refinements are within project scope.

## Appendix 4: Submodel development details

In each software sprint, each submodel team will

1. Set priorities for the design iteration:
  - Discuss feedback and identify highest priority model refinements.
  - Refine model assumptions and hypotheses.
  - Assess new data to refine parameter estimates.
2. “Translate” biological hypotheses into agent model rules and other mathematical model components:
  - Run the new hypotheses and rules by domain experts as their time permits.
  - Define new qualitative and/or quantitative unit tests for new behaviors and functions.
  - Assign implementation tasks.
3. Perform computational implementation of refined mathematical model (and submodels):
  - Address any bug reports.
  - Add or modify functions based on new rules in steps 1–2.
  - Test new or altered functions.
  - Satisfy all qualitative and/or quantitative unit tests.
  - Qualitatively test the model for new or improved behaviors over the last iteration.
4. Create a software release:
  - Update documentation.
  - Create a new numbered release on GitHub.
  - Update list of available validation data and best parameter estimates.
  - Create a Zenodo snapshot.
  - Communicate with the core team on the software release.
5. Create a cloud-hosted submodel for multidisciplinary testing:
  - Update the nanoHUB app repository with new code.
  - Run xml2jupyter to update the Jupyter interface.
  - Update project on nanoHUB, test/refine until successful release.
  - Update documentation, numbered GitHub release, and Zenodo snapshot of deployed model.
  - Perform live demos with the core team as needed.

While waiting for the start of the next software sprint, each submodel team will

1. Perform model evaluation:
  - Distill feedback to assess the need for new model hypotheses, behaviors, or components.
  - Assess which biological behaviors are currently exhibited by the model.
  - Refine the design protocol (e.g., with refined model specification methods) as necessary.
  - Assess the need for an additional design iteration.
2. Help update the preprint for scientific dissemination.

In the next software sprint, return to Step 1 if there is substantial feedback, or if the core team determines that further refinements are within project scope.

## References

[1] Dong E., Du H. & Gardner L. An interactive web-based dashboard to track COVID-19 in real time. The Lancet Infectious Diseases, doi:10.1016/s1473-3099(20)30120-1 (2020). PMC7159018 32087114

[2] Rozenblatt-Rosen O. The Human Tumor Atlas Network: Charting Tumor Transitions across Space and Time at Single-Cell Resolution. Cell 181, 236–249, doi:10.1016/j.cell.2020.03.053 (2020). 32302568 PMC7376497

[3] Zhou P. A pneumonia outbreak associated with a new coronavirus of probable bat origin. Nature 579, 270–273, doi:10.1038/s41586-020-2012-7 (2020). 32015507 PMC7095418

[4] Bouadma L., Lescure F.-X., Lucet J.-C., Yazdanpanah Y. & Timsit J.-F. Severe SARS-CoV-2 infections: practical considerations and management strategy for intensivists. Intensive Care Medicine 46, 579–582, doi:10.1007/s00134-020-05967-x (2020). 32103284 PMC7079839

[5] Yang X. Clinical course and outcomes of critically ill patients with SARS-CoV-2 pneumonia in Wuhan, China: a single-centered, retrospective, observational study. The Lancet Respiratory Medicine, doi:10.1016/s2213-2600(20)30079-5 (2020). PMC7102538 32105632

[6] Zhou F. Clinical course and risk factors for mortality of adult inpatients with COVID-19 in Wuhan, China: a retrospective cohort study. The Lancet 395, 1054–1062, doi:10.1016/s0140-6736(20)30566-3 (2020). PMC7270627 32171076

[7] Zhang J. j. Clinical characteristics of 140 patients infected with SARS-CoV-2 in Wuhan, China. Allergy, doi:10.1111/all.14238 (2020). 32077115

[8] Liang W. Cancer patients in SARS-CoV-2 infection: a nationwide analysis in China. The Lancet Oncology 21, 335–337, doi:10.1016/s1470-2045(20)30096-6 (2020). 32066541 PMC7159000

[9] Macklin P. When Seeing Isn’t Believing: How Math Can Guide Our Interpretation of Measurements and Experiments. Cell Systems 5, 92–94, doi:10.1016/j.cels.2017.08.005 (2017). 28837815

[10] Macklin P. Key challenges facing data-driven multicellular systems biology. GigaScience 8, doi:10.1093/gigascience/giz127 (2019). PMC6812467 31648301

[11] Wrapp D. Cryo-EM structure of the 2019-nCoV spike in the prefusion conformation. Science 367, 1260–1263, doi:10.1126/science.abb2507 (2020). 32075877 PMC7164637

[12] Hoffmann M. SARS-CoV-2 Cell Entry Depends on ACE2 and TMPRSS2 and Is Blocked by a Clinically Proven Protease Inhibitor. Cell 181, 271–280.e278, doi:10.1016/j.cell.2020.02.052 (2020). 32142651 PMC7102627

[13] Sahin A. R. 2019 Novel Coronavirus (COVID-19) Outbreak: A Review of the Current Literature. Eurasian Journal of Medicine and Oncology, doi:10.14744/ejmo.2020.12220 (2020).

[14] White K. A., Enjuanes L. & Berkhout B. RNA virus replication, transcription and recombination. RNA Biology 8, 182–183, doi:10.4161/rna.8.2.15663 (2014). PMC3127097 21593586

[15] Stark G. R., Kerr I. M., Williams B. R. G., Silverman R. H. & Schreiber R. D. How Cells Respond to Interferons. Annual Review of Biochemistry 67, 227–264, doi:10.1146/annurev.biochem.67.1.227 (1998). 9759489

[16] Perry A. K., Chen G., Zheng D., Tang H. & Cheng G. The host type I interferon response to viral and bacterial infections. Cell Research 15, 407–422, doi:10.1038/sj.cr.7290309 (2005). 15987599

[17] Spiegel M. Inhibition of Beta Interferon Induction by Severe Acute Respiratory Syndrome Coronavirus Suggests a Two-Step Model for Activation of Interferon Regulatory Factor 3. Journal of Virology 79, 2079–2086, doi:10.1128/jvi.79.4.2079-2086.2005 (2005). 15681410 PMC546554

[18] Hale B. G., Randall R. E., Ortín J. & Jackson D. The multifunctional NS1 protein of influenza A viruses. Journal of General Virology 89, 2359–2376, doi:10.1099/vir.0.2008/004606-0 (2008). 18796704

[19] Danthi P. Viruses and the Diversity of Cell Death. Annual Review of Virology 3, 533–553, doi:10.1146/annurev-virology-110615-042435 (2016). 27501259

[20] Yue Y. SARS-Coronavirus Open Reading Frame-3a drives multimodal necrotic cell death. Cell Death & Disease 9, doi:10.1038/s41419-018-0917-y (2018). PMC6125346 30185776

[21] Keck F. Altered mitochondrial dynamics as a consequence of Venezuelan Equine encephalitis virus infection. Virulence 8, 1849–1866, doi:10.1080/21505594.2016.1276690 (2017). 28075229 PMC5810500

[22] Keck F. Mitochondrial-Directed Antioxidant Reduces Microglial-Induced Inflammation in Murine In Vitro Model of TC-83 Infection. Viruses 10, doi:10.3390/v10110606 (2018). PMC6266753 30400156

[23] Thiel V. Lack of Innate Interferon Responses during SARS Coronavirus Infection in a Vaccination and Reinfection Ferret Model. PLoS ONE 7, e45842, doi:10.1371/journal.pone.0045842 (2012). 23029269 PMC3454321

[24] Widagdo W., Okba N. M. A., Stalin Raj V. & Haagmans B. L. MERS-coronavirus: From discovery to intervention. One Health 3, 11–16, doi:10.1016/j.onehlt.2016.12.001 (2017). 28616497 PMC5454172

[25] Channappanavar R. Dysregulated Type I Interferon and Inflammatory Monocyte-Macrophage Responses Cause Lethal Pneumonia in SARS-CoV-Infected Mice. Cell Host & Microbe 19, 181–193, doi:10.1016/j.chom.2016.01.007 (2016). 26867177 PMC4752723

[26] Channappanavar R. IFN-I response timing relative to virus replication determines MERS coronavirus infection outcomes. Journal of Clinical Investigation 129, 3625–3639, doi:10.1172/jci126363 (2019). PMC6715373 31355779

[27] Al-Hazmi A. Challenges presented by MERS corona virus, and SARS corona virus to global health. Saudi Journal of Biological Sciences 23, 507–511, doi:10.1016/j.sjbs.2016.02.019 (2016). 27298584 PMC4890194

[28] Yuen K.-Y. Comparative replication and immune activation profiles of SARS-CoV-2 and SARS-CoV in human lungs: an ex vivo study with implications for the pathogenesis of COVID-19. Clinical Infectious Diseases, doi:10.1093/cid/ciaa410 (2020). PMC7184390 32270184

[29] Qin C. Dysregulation of Immune Response in Patients with COVID-19 in Wuhan, China. SSRN Electronic Journal, doi:10.2139/ssrn.3541136 (2020).

[30] Jamilloux Y. Should we stimulate or suppress immune responses in COVID-19? Cytokine and anti-cytokine interventions. Autoimmunity Reviews 19, 102567, doi:10.1016/j.autrev.2020.102567 (2020). 32376392 PMC7196557

[31] Prompetchara E., Ketloy C. & Palaga T. Immune responses in COVID-19 and potential vaccines: Lessons learned from SARS and MERS epidemic. Asian Pacific Journal of Allergy and Immunology, doi:10.12932/ap-200220-0772 (2020). 32105090

[32] Channappanavar R. & Perlman S. Pathogenic human coronavirus infections: causes and consequences of cytokine storm and immunopathology. Seminars in Immunopathology 39, 529–539, doi:10.1007/s00281-017-0629-x (2017). 28466096 PMC7079893

[33] Nahrendorf M., Pittet M. J. & Swirski F. K. Monocytes: Protagonists of Infarct Inflammation and Repair After Myocardial Infarction. Circulation 121, 2437–2445, doi:10.1161/circulationaha.109.916346 (2010). 20530020 PMC2892474

[34] Fung S.-Y., Yuen K.-S., Ye Z.-W., Chan C.-P. & Jin D.-Y. A tug-of-war between severe acute respiratory syndrome coronavirus 2 and host antiviral defence: lessons from other pathogenic viruses. Emerging Microbes & Infections 9, 558–570, doi:10.1080/22221751.2020.1736644 (2020). 32172672 PMC7103735

[35] Siu K. L. Severe acute respiratory syndrome Coronavirus ORF3a protein activates the NLRP3 inflammasome by promoting TRAF3‐dependent ubiquitination of ASC. The FASEB Journal 33, 8865–8877, doi:10.1096/fj.201802418R (2019). 31034780 PMC6662968

[36] Chen I. Y., Moriyama M., Chang M.-F. & Ichinohe T. Severe Acute Respiratory Syndrome Coronavirus Viroporin 3a Activates the NLRP3 Inflammasome. Frontiers in Microbiology 10, doi:10.3389/fmicb.2019.00050 (2019). PMC6361828 30761102

[37] Camp J. V. & Jonsson C. B. A Role for Neutrophils in Viral Respiratory Disease. Frontiers in Immunology 8, doi:10.3389/fimmu.2017.00550 (2017). PMC5427094 28553293

[38] Weitnauer M., Mijošek V. & Dalpke A. H. Control of local immunity by airway epithelial cells. Mucosal Immunology 9, 287–298, doi:10.1038/mi.2015.126 (2015). 26627458

[39] Crane M. J., Lee K. M., FitzGerald E. S. & Jamieson A. M. Surviving Deadly Lung Infections: Innate Host Tolerance Mechanisms in the Pulmonary System. Frontiers in Immunology 9, doi:10.3389/fimmu.2018.01421 (2018). PMC6024012 29988424

[40] Teijaro John R. Endothelial Cells Are Central Orchestrators of Cytokine Amplification during Influenza Virus Infection. Cell 146, 980–991, doi:10.1016/j.cell.2011.08.015 (2011). 21925319 PMC3176439

[41] Liu J. , doi:10.1101/2020.02.16.20023671 (2020).

[42] Liu J. , doi:10.1101/2020.02.10.20021584 (2020).

[43] Shokri S., Mahmoudvand S., Taherkhani R. & Farshadpour F. Modulation of the immune response by Middle East respiratory syndrome coronavirus. Journal of Cellular Physiology 234, 2143–2151, doi:10.1002/jcp.27155 (2018). 30146782 PMC7166610

[44] Kong W. p. Modulation of the Immune Response to the Severe Acute Respiratory Syndrome Spike Glycoprotein by Gene-Based and Inactivated Virus Immunization. Journal of Virology 79, 13915–13923, doi:10.1128/jvi.79.22.13915-13923.2005 (2005). 16254327 PMC1280202

[45] Cruz J. L. G. Alphacoronavirus Protein 7 Modulates Host Innate Immune Response. Journal of Virology 87, 9754–9767, doi:10.1128/jvi.01032-13 (2013). 23824792 PMC3754097

[46] Liu L. Anti–spike IgG causes severe acute lung injury by skewing macrophage responses during acute SARS-CoV infection. JCI Insight 4, doi:10.1172/jci.insight.123158 (2019). PMC6478436 30830861

[47] Leong A. S. Y. Multiple organ infection and the pathogenesis of SARS. Journal of Experimental Medicine 202, 415–424, doi:10.1084/jem.20050828 (2005). PMC2213088 16043521

[48] Yang P. Angiotensin-converting enzyme 2 (ACE2) mediates influenza H7N9 virus-induced acute lung injury. Scientific Reports 4, doi:10.1038/srep07027 (2014). PMC4229671 25391767

[49] Kuba K. A crucial role of angiotensin converting enzyme 2 (ACE2) in SARS coronavirus–induced lung injury. Nature Medicine 11, 875–879, doi:10.1038/nm1267 (2005). PMC7095783 16007097

[50] Madjid M., Safavi-Naeini P., Solomon S. D. & Vardeny O. Potential Effects of Coronaviruses on the Cardiovascular System: A Review. JAMA Cardiol, doi:10.1001/jamacardio.2020.1286 (2020). 32219363

[51] Wadman M., Couzin-Frankel J., Kaiser J. & Matacic C. A rampage through the body. Science 368, 356–360, doi:10.1126/science.368.6489.356 (2020). 32327580

[52] Stebbing J. COVID-19: combining antiviral and anti-inflammatory treatments. The Lancet Infectious Diseases 20, 400–402, doi:10.1016/s1473-3099(20)30132-8 (2020). 32113509 PMC7158903

[53] Herold T. , doi:10.1101/2020.04.01.20047381 (2020).

[54] Zhang C., Wu Z., Li J.-W., Zhao H. & Wang G.-Q. The cytokine release syndrome (CRS) of severe COVID-19 and Interleukin-6 receptor (IL-6R) antagonist Tocilizumab may be the key to reduce the mortality. International Journal of Antimicrobial Agents, 105954, doi:10.1016/j.ijantimicag.2020.105954 (2020). 32234467 PMC7118634

[55] Schett G., Sticherling M. & Neurath M. F. COVID-19: risk for cytokine targeting in chronic inflammatory diseases? Nature Reviews Immunology, doi:10.1038/s41577-020-0312-7 (2020). PMC7186927 32296135

[56] Sanders C. J. Compromised respiratory function in lethal influenza infection is characterized by the depletion of type I alveolar epithelial cells beyond threshold levels. American Journal of Physiology-Lung Cellular and Molecular Physiology 304, L481–L488, doi:10.1152/ajplung.00343.2012 (2013). 23355384 PMC3627938

[57] Allen R. J., Rieger T. R. & Musante C. J. Efficient Generation and Selection of Virtual Populations in Quantitative Systems Pharmacology Models. CPT: Pharmacometrics & Systems Pharmacology 5, 140–146, doi:10.1002/psp4.12063 (2016). 27069777 PMC4809626

[58] Cassidy T. & Craig M. Determinants of combination GM-CSF immunotherapy and oncolytic virotherapy success identified through in silico treatment personalization. PLOS Computational Biology 15, doi:10.1371/journal.pcbi.1007495 (2019). PMC6880985 31774808

[59] Nowak M. Antigenic diversity thresholds and the development of AIDS. Science 254, 963–969, doi:10.1126/science.1683006 (1991). 1683006

[60] Wei X. Viral dynamics in human immunodeficiency virus type 1 infection. Nature 373, 117–122, doi:10.1038/373117a0 (1995). 7529365

[61] Rosenbloom D. I. S., Hill A. L., Laskey S. B. & Siliciano R. F. Re-evaluating evolution in the HIV reservoir. Nature 551, E6–E9, doi:10.1038/nature24634 (2017). 29168805 PMC6103791

[62] Reeves D. B. A majority of HIV persistence during antiretroviral therapy is due to infected cell proliferation. Nature Communications 9, doi:10.1038/s41467-018-06843-5 (2018). PMC6240116 30446650

[63] Koelle K., Farrell A. P., Brooke C. B. & Ke R. Within-host infectious disease models accommodating cellular coinfection, with an application to influenza†. Virus Evolution 5, doi:10.1093/ve/vez018 (2019). PMC6613536 31304043

[64] Perelson A. S., Neumann A. U., Markowitz M., Leonard J. M. & Ho D. D. HIV-1 Dynamics in Vivo: Virion Clearance Rate, Infected Cell Life-Span, and Viral Generation Time. Science 271, 1582–1586, doi:10.1126/science.271.5255.1582 (1996). 8599114

[65] Smith A. M. & Ribeiro R. M. Modeling the Viral Dynamics of Influenza A Virus Infection. Critical Reviews^™^ in Immunology 30, 291–298, doi:10.1615/CritRevImmunol.v30.i3.60 (2010). 20370636

[66] Smith A. M. & Perelson A. S. Influenza A virus infection kinetics: quantitative data and models. Wiley Interdisciplinary Reviews: Systems Biology and Medicine 3, 429–445, doi:10.1002/wsbm.129 (2011). 21197654 PMC3256983

[67] Smith A. M., McCullers J. A. & Adler F. R. Mathematical model of a three-stage innate immune response to a pneumococcal lung infection. Journal of Theoretical Biology 276, 106–116, doi:10.1016/j.jtbi.2011.01.052 (2011). 21300073 PMC3066295

[68] Bonhoeffer S., May R. M., Shaw G. M. & Nowak M. A. Virus dynamics and drug therapy. Proceedings of the National Academy of Sciences 94, 6971–6976, doi:10.1073/pnas.94.13.6971 (1997). PMC21269 9192676

[69] Hill A. L., Rosenbloom D. I. S., Fu F., Nowak M. A. & Siliciano R. F. Predicting the outcomes of treatment to eradicate the latent reservoir for HIV-1. Proceedings of the National Academy of Sciences 111, 13475–13480, doi:10.1073/pnas.1406663111 (2014). PMC4169952 25097264

[70] Rosenbloom D. I. S., Hill A. L., Rabi S. A., Siliciano R. F. & Nowak M. A. Antiretroviral dynamics determines HIV evolution and predicts therapy outcome. Nature Medicine 18, 1378–1385, doi:10.1038/nm.2892 (2012). PMC3490032 22941277

[71] Mueller S. N. Mathematical Modeling Predicts that Increased HSV-2 Shedding in HIV-1 Infected Persons Is Due to Poor Immunologic Control in Ganglia and Genital Mucosa. Plos One 11, e0155124, doi:10.1371/journal.pone.0155124 (2016). 27285483 PMC4902308

[72] Perelson A. S. Decay characteristics of HIV-1-infected compartments during combination therapy. Nature 387, 188–191, doi:10.1038/387188a0 (1997). 9144290

[73] Kirtane A. R. Development of an oral once-weekly drug delivery system for HIV antiretroviral therapy. Nature Communications 9, doi:10.1038/s41467-017-02294-6 (2018). PMC5760734 29317618

[74] Schiffer J. T. & Gottlieb S. L. Biologic interactions between HSV-2 and HIV-1 and possible implications for HSV vaccine development. Vaccine 37, 7363–7371, doi:10.1016/j.vaccine.2017.09.044 (2019). 28958807 PMC5867191

[75] Schiffer J. T. Mathematical modeling of herpes simplex virus-2 suppression with pritelivir predicts trial outcomes. Science Translational Medicine 8, 324ra315–324ra315, doi:10.1126/scitranslmed.aad6654 (2016). PMC4880060 26843190

[76] Perelson A. S. Modelling viral and immune system dynamics. Nature Reviews Immunology 2, 28–36, doi:10.1038/nri700 (2002). 11905835

[77] Hill A. L., Rosenbloom D. I. S., Nowak M. A. & Siliciano R. F. Insight into treatment of HIV infection from viral dynamics models. Immunological Reviews 285, 9–25, doi:10.1111/imr.12698 (2018). 30129208 PMC6155466

[78] Wang Y., Zhou Y., Brauer F. & Heffernan J. M. Viral dynamics model with CTL immune response incorporating antiretroviral therapy. Journal of Mathematical Biology 67, 901–934, doi:10.1007/s00285-012-0580-3 (2012). 22930342

[79] Schiffer J. T. & Corey L. Rapid host immune response and viral dynamics in herpes simplex virus-2 infection. Nature Medicine 19, 280–288, doi:10.1038/nm.3103 (2013). PMC3981536 23467247

[80] Jenner A. L., Yun C.-O., Kim P. S. & Coster A. C. F. Mathematical Modelling of the Interaction Between Cancer Cells and an Oncolytic Virus: Insights into the Effects of Treatment Protocols. Bulletin of Mathematical Biology 80, 1615–1629, doi:10.1007/s11538-018-0424-4 (2018). 29644518

[81] Möhler L., Flockerzi D., Sann H. & Reichl U. Mathematical model of influenza A virus production in large-scale microcarrier culture. Biotechnology and Bioengineering 90, 46–58, doi:10.1002/bit.20363 (2005). 15736163

[82] Schulze-Horsel J., Schulze M., Agalaridis G., Genzel Y. & Reichl U. Infection dynamics and virus-induced apoptosis in cell culture-based influenza vaccine production—Flow cytometry and mathematical modeling. Vaccine 27, 2712–2722, doi:10.1016/j.vaccine.2009.02.027 (2009). 19428884

[83] Banks H. T., Bortz D. M. & Holte S. E. Incorporation of variability into the modeling of viral delays in HIV infection dynamics. Mathematical Biosciences 183, 63–91, doi:10.1016/s0025-5564(02)00218-3 (2003). 12604136

[84] Culshaw R. V., Ruan S. & Webb G. A mathematical model of cell-to-cell spread of HIV-1 that includes a time delay. Journal of Mathematical Biology 46, 425–444, doi:10.1007/s00285-002-0191-5 (2003). 12750834

[85] Li M. Y. & Shu H. Impact of Intracellular Delays and Target-Cell Dynamics on In Vivo Viral Infections. SIAM Journal on Applied Mathematics 70, 2434–2448, doi:10.1137/090779322 (2010).

[86] Smith A. M. Validated models of immune response to virus infection. Current Opinion in Systems Biology 12, 46–52, doi:10.1016/j.coisb.2018.10.005 (2018). 31723715 PMC6853615

[87] Smith A. M. Host-pathogen kinetics during influenza infection and coinfection: insights from predictive modeling. Immunological Reviews 285, 97–112, doi:10.1111/imr.12692 (2018). 30129197 PMC6175135

[88] Guedj J. Modeling shows that the NS5A inhibitor daclatasvir has two modes of action and yields a shorter estimate of the hepatitis C virus half-life. Proceedings of the National Academy of Sciences 110, 3991–3996, doi:10.1073/pnas.1203110110 (2013). PMC3593898 23431163

[89] Heldt F. S., Frensing T. & Reichl U. Modeling the Intracellular Dynamics of Influenza Virus Replication To Understand the Control of Viral RNA Synthesis. Journal of Virology 86, 7806–7817, doi:10.1128/jvi.00080-12 (2012). 22593159 PMC3421648

[90] Sidorenko Y. & Reichl U. Structured model of influenza virus replication in MDCK cells. Biotechnology and Bioengineering 88, 1–14, doi:10.1002/bit.20096 (2004). 15384040

[91] Fachada N., Lopes V. V. & Rosa A. Simulating antigenic drift and shift in influenza A. 2093, doi:10.1145/1529282.1529744 (2009).

[92] Levin D. A spatial model of the efficiency of T cell search in the influenza-infected lung. Journal of Theoretical Biology 398, 52–63, doi:10.1016/j.jtbi.2016.02.022 (2016). 26920246 PMC4862360

[93] Beauchemin C., Samuel J. & Tuszynski J. A simple cellular automaton model for influenza A viral infections. Journal of Theoretical Biology 232, 223–234, doi:10.1016/j.jtbi.2004.08.001 (2005). 15530492

[94] Beauchemin C. Probing the effects of the well-mixed assumption on viral infection dynamics. Journal of Theoretical Biology 242, 464–477, doi:10.1016/j.jtbi.2006.03.014 (2006). 16650441

[95] Beauchemin C., Forrest S. & Koster F. T. Modeling Influenza Viral Dynamics in Tissue. 4163, 23–36, doi:10.1007/11823940_3 (2006).

[96] Akpinar F., Inankur B., Yin J. & Lyles D. S. Spatial-Temporal Patterns of Viral Amplification and Interference Initiated by a Single Infected Cell. Journal of Virology 90, 7552–7566, doi:10.1128/jvi.00807-16 (2016). 27279621 PMC4984635

[97] Bauer A. L., Beauchemin C. A. A. & Perelson A. S. Agent-based modeling of host–pathogen systems: The successes and challenges. Information Sciences 179, 1379–1389, doi:10.1016/j.ins.2008.11.012 (2009). 20161146 PMC2731970

[98] Medyukhina A., Timme S., Mokhtari Z. & Figge M. T. Image-based systems biology of infection. Cytometry Part A 87, 462–470, doi:10.1002/cyto.a.22638 (2015). 25641512

[99] Bankhead A. A simulation framework to investigate in vitro viral infection dynamics. Journal of Computational Science 4, 127–134, doi:10.1016/j.jocs.2011.08.007 (2013). 23682300 PMC3652481

[100] Wilke C. O. Complex Spatial Dynamics of Oncolytic Viruses In Vitro: Mathematical and Experimental Approaches. PLoS Computational Biology 8, e1002547, doi:10.1371/journal.pcbi.1002547 (2012). 22719239 PMC3375216

[101] Sun G.-Q. Spatiotemporal Dynamics of Virus Infection Spreading in Tissues. Plos One 11, e0168576, doi:10.1371/journal.pone.0168576 (2016). 27997613 PMC5173377

[102] Jenner A. L., Frascoli F., Coster A. C. F. & Kim P. S. Enhancing oncolytic virotherapy: Observations from a Voronoi Cell-Based model. Journal of Theoretical Biology 485, 110052, doi:10.1016/j.jtbi.2019.110052 (2020). 31626813

[103] Quintela B. M. An Age-based Multiscale Mathematical Model of the Hepatitis C Virus Life-cycle During Infection and Therapy: Including Translation and Replication. 60, 508–511, doi:10.1007/978-981-10-4086-3_128 (2017).

[104] An G. Agent-based computer simulation and sirs: building a bridge between basic science and clinical trials. Shock 16, 266–273, doi:10.1097/00024382-200116040-00006 (2001). 11580108

[105] An G. In silico experiments of existing and hypothetical cytokine-directed clinical trials using agent-based modeling*. Critical Care Medicine 32, 2050–2060, doi:10.1097/01.ccm.0000139707.13729.7d (2004). 15483414

[106] Hunt C. A., Cockrell R. C. & An G. Examining the controllability of sepsis using genetic algorithms on an agent-based model of systemic inflammation. PLOS Computational Biology 14, e1005876, doi:10.1371/journal.pcbi.1005876 (2018). 29447154 PMC5813897

[107] Petersen B. K. Deep Reinforcement Learning and Simulation as a Path Toward Precision Medicine. Journal of Computational Biology 26, 597–604, doi:10.1089/cmb.2018.0168 (2019). 30681362 PMC6590719

[108] An G. Introduction of an agent-based multi-scale modular architecture for dynamic knowledge representation of acute inflammation. Theoretical Biology and Medical Modelling 5, doi:10.1186/1742-4682-5-11 (2008). PMC2442588 18505587

[109] Ghaffarizadeh A., Heiland R., Friedman S. H., Mumenthaler S. M. & Macklin P. PhysiCell: An open source physics-based cell simulator for 3-D multicellular systems. PLoS Comput Biol 14, e1005991, doi:10.1371/journal.pcbi.1005991 (2018). 29474446 PMC5841829

[110] Cooper F. Chaste: Cancer, Heart and Soft Tissue Environment. Journal of Open Source Software 5, 1848, doi:10.21105/joss.01848 (2020). PMC7614534 37192932

[111] Prlic A. Chaste: An Open Source C++ Library for Computational Physiology and Biology. PLoS Computational Biology 9, e1002970, doi:10.1371/journal.pcbi.1002970 (2013). 23516352 PMC3597547

[112] Starruß J., de Back W., Brusch L. & Deutsch A. Morpheus: a user-friendly modeling environment for multiscale and multicellular systems biology. Bioinformatics 30, 1331–1332, doi:10.1093/bioinformatics/btt772 (2014). 24443380 PMC3998129

[113] Sego T. J. A Modular Framework for Multiscale Spatial Modeling of Viral Infection and Immune Response in Epithelial Tissue. bioRxiv, 2020.2004.2027.064139, doi:10.1101/2020.04.27.064139 (2020). PMC7785254 33347439

[114] Kang S., Kahan S., McDermott J., Flann N. & Shmulevich I. Biocellion: accelerating computer simulation of multicellular biological system models. Bioinformatics 30, 3101–3108, doi:10.1093/bioinformatics/btu498 (2014). 25064572 PMC4609016

[115] Hillen T. COVID-19 Physiology Group, <https://sites.google.com/ualberta.ca/cov-pg/home> (2020).

[116] Becker A., An G. & Cockrell C. The Cellular Immunity Agent Based Model (CIABM): Replicating the cellular immune response to viral respiratory infection. bioRxiv, 663930, doi:10.1101/663930 (2020).

[117] Ostaszewski M. COVID-19 Disease Map, building a computational repository of SARS-CoV-2 virus-host interaction mechanisms. Scientific Data 7, doi:10.1038/s41597-020-0477-8 (2020). PMC7200764 32371892

[118] Hu B. C. The human body at cellular resolution: the NIH Human Biomolecular Atlas Program. Nature 574, 187–192, doi:10.1038/s41586-019-1629-x (2019). 31597973 PMC6800388

[119] Metzcar J., Wang Y., Heiland R. & Macklin P. A Review of Cell-Based Computational Modeling in Cancer Biology. JCO Clin Cancer Inform 3, 1–13, doi:10.1200/CCI.18.00069 (2019). PMC6584763 30715927

[120] Ghaffarizadeh A., Friedman S. H. & Macklin P. BioFVM: an efficient, parallelized diffusive transport solver for 3-D biological simulations. Bioinformatics 32, 1256–1258, doi:10.1093/bioinformatics/btv730 (2016). 26656933 PMC4824128

[121] Wang Y., Heiland R. & Macklin P. pc4nanobio: cancer nanotherapy simulator [nanoHUB app, Version 0.9.1], <https://nanohub.org/resources/pc4nanobio> (2019).

[122] Jenner A. L. Replication Competent Oncolytic Virus expressing secretable trimeric TRAIL: hypothesis testing [nanoHUB app, Version 0.3], <https://nanohub.org/resources/iu399sp19p101> (2019).

[123] Wang Y., Heiland R. & Macklin P. Physicell: liver tissue mechanobiology [nanoHUB app, Version 1.2], <https://nanohub.org/resources/pc4livermedium> (2019).

[124] Ozik J. High-throughput cancer hypothesis testing with an integrated PhysiCell-EMEWS workflow. BMC Bioinformatics 19, 483, doi:10.1186/s12859-018-2510-x (2018). 30577742 PMC6302449

[125] Ozik J., Collier N., Heiland R., An G. & Macklin P. Learning-accelerated discovery of immune-tumour interactions. Mol Syst Des Eng 4, 747–760, doi:10.1039/c9me00036d (2019). 31497314 PMC6690424

[126] Wang Y. Rapid community-driven development of a SARS-CoV-2 tissue simulator. bioRxiv, 10.1101/2020.1104.1102.019075, doi:10.1101/2020.04.02.019075 (2020).

[127] Hucka M. The systems biology markup language (SBML): a medium for representation and exchange of biochemical network models. Bioinformatics 19, 524–531, doi:10.1093/bioinformatics/btg015 (2003). 12611808

[128] Somogyi E. T. libRoadRunner: a high performance SBML simulation and analysis library. Bioinformatics 31, 3315–3321, doi:10.1093/bioinformatics/btv363 (2015). 26085503 PMC4607739

[129] Stoll G. MaBoSS 2.0: an environment for stochastic Boolean modeling. Bioinformatics 33, 2226–2228, doi:10.1093/bioinformatics/btx123 (2017). 28881959

[130] Letort G. PhysiBoSS: a multi-scale agent-based modelling framework integrating physical dimension and cell signalling. Bioinformatics 35, 1188–1196, doi:10.1093/bioinformatics/bty766 (2019). 30169736 PMC6449758

[131] Yates A., Bergmann C., Leo Van Hemmen J., Stark J. & Callard R. Cytokine-modulated Regulation of Helper T Cell Populations. Journal of Theoretical Biology 206, 539–560, doi:10.1006/jtbi.2000.2147 (2000). 11013114

[132] Schaff J. C., Gao F., Li Y., Novak I. L. & Slepchenko B. M. Numerical Approach to Spatial Deterministic-Stochastic Models Arising in Cell Biology. PLOS Computational Biology 12, e1005236, doi:10.1371/journal.pcbi.1005236 (2016). 27959915 PMC5154471

[133] Ozik J., Collier N. T., Wozniak J. M. & Spagnuolo C. in 2016 Winter Simulation Conference (WSC) 206–220 (2016). 10.1109/WSC.2016.7822090 PMC6592281 31239603

[134] Ozik J., Collier N. T., Wozniak J. M., Macal C. M. & An G. Extreme-Scale Dynamic Exploration of a Distributed Agent-Based Model With the EMEWS Framework. IEEE Transactions on Computational Social Systems 5, 884–895, doi:10.1109/tcss.2018.2859189 (2018). 30349868 PMC6195352

[135] Khanna A. S. A modeling framework to inform preexposure prophylaxis initiation and retention scale-up in the context of ‘Getting to Zero’ initiatives. Aids 33, 1911–1922, doi:10.1097/qad.0000000000002290 (2019). 31490212 PMC6760326

[136] Tatara E. in 2019 Winter Simulation Conference (WSC) 1008–1019 (2019). 10.1109/wsc40007.2019.9004747 PMC7335458 32624641

[137] Rutter C. M., Ozik J., DeYoreo M. & Collier N. Microsimulation model calibration using incremental mixture approximate Bayesian computation. The Annals of Applied Statistics 13, 2189–2212, doi:10.1214/19-aoas1279 (2019). PMC8534811 34691351

[138] Madhavan K. nanoHUB.org: cloud-based services for nanoscale modeling, simulation, and education. Nanotechnology Reviews 2, doi:10.1515/ntrev-2012-0043 (2013).

[139] Kluyver T. in ELPUB. 87–90.

[140] Friedman S. H. MultiCellDS: a community-developed standard for curating microenvironment-dependent multicellular data [Preprint]. bioRxiv 090456, doi:10.1101/090456 (2016).

[141] Heiland R., Mishler D., Zhang T., Bower E. & Macklin P. xml2jupyter: Mapping parameters between XML and Jupyter widgets. J Open Source Softw 4, doi:10.21105/joss.01408 (2019). PMC6656392 31342010

[142] Macklin P., Edgerton M. E., Thompson A. M. & Cristini V. Patient-calibrated agent-based modelling of ductal carcinoma in situ (DCIS): from microscopic measurements to macroscopic predictions of clinical progression. J Theor Biol 301, 122–140, doi:10.1016/j.jtbi.2012.02.002 (2012). 22342935 PMC3322268

[143] Pienaar E. A computational tool integrating host immunity with antibiotic dynamics to study tuberculosis treatment. Journal of Theoretical Biology 367, 166–179, doi:10.1016/j.jtbi.2014.11.021 (2015). 25497475 PMC4332617

[144] Cannon G. J. & Swanson J. A. The macrophage capacity for phagocytosis. J Cell Sci 101 (Pt 4), 907–913 (1992). 1527185 10.1242/jcs.101.4.907

[145] Jr R. A. F. Nanomedicine, Volume IIA: Biocompatibility. (Landes Bioscience, 2003).

[146] Zent C. S. & Elliott M. R. Maxed out macs: physiologic cell clearance as a function of macrophage phagocytic capacity. The FEBS Journal 284, 1021–1039, doi:10.1111/febs.13961 (2017). 27863012 PMC5378628

[147] Ghaffarizadeh A., Heiland R., Friedman S. H., Mumenthaler S. M. & Macklin P. PhysiCell: An open source physics-based cell simulator for 3-D multicellular systems. PLOS Computational Biology 14, e1005991, doi:10.1371/journal.pcbi.1005991 (2018). 29474446 PMC5841829

[148] Cook P. C. & MacDonald A. S. Dendritic cells in lung immunopathology. Seminars in Immunopathology 38, 449–460, doi:10.1007/s00281-016-0571-3 (2016). 27256370 PMC4896986

[149] Dumortier H. Antigen Presentation by an Immature Myeloid Dendritic Cell Line Does Not Cause CTL Deletion In Vivo, but Generates CD8+ Central Memory-Like T Cells That Can Be Rescued for Full Effector Function. The Journal of Immunology 175, 855–863, doi:10.4049/jimmunol.175.2.855 (2005). 16002683

[150] Beikzadeh B. & Delirezh N. Phenotypic and functional comparison of two distinct subsets of programmable cell of monocytic origin (PCMOs)-derived dendritic cells with conventional monocyte-derived dendritic cells. Cellular & Molecular Immunology 13, 160–169, doi:10.1038/cmi.2014.135 (2016). 25661728 PMC4786623

[151] Gong C. Predicting lymph node output efficiency using systems biology. Journal of Theoretical Biology 335, 169–184, doi:10.1016/j.jtbi.2013.06.016 (2013). 23816876 PMC3783027

[152] Miller M. J., Hejazi A. S., Wei S. H., Cahalan M. D. & Parker I. T cell repertoire scanning is promoted by dynamic dendritic cell behavior and random T cell motility in the lymph node. Proceedings of the National Academy of Sciences of the United States of America 101, 998–1003, doi:10.1073/pnas.0306407101 (2004). 14722354 PMC327133

[153] Marino S. & Kirschner D. E. A Multi-Compartment Hybrid Computational Model Predicts Key Roles for Dendritic Cells in Tuberculosis Infection. Computation 4, 39 (2016). 28989808 10.3390/computation4040039 PMC5627612

[154] Wang M. Quantifying CD4 receptor protein in two human CD4+ lymphocyte preparations for quantitative flow cytometry. Clinical Proteomics 11, 43, doi:10.1186/1559-0275-11-43 (2014). 25593565 PMC4277840

[155] De Boer R. J. Recruitment Times, Proliferation, and Apoptosis Rates during the CD8+ T-Cell Response to Lymphocytic Choriomeningitis Virus. Journal of Virology 75, 10663–10669, doi:10.1128/jvi.75.22.10663-10669.2001 (2001). 11602708 PMC114648

[156] Stutz A., Golenbock D. T. & Latz E. Inflammasomes: too big to miss. Journal of Clinical Investigation 119, 3502–3511, doi:10.1172/jci40599 (2009). PMC2786809 19955661

[157] Bergsbaken T., Fink S. L. & Cookson B. T. Pyroptosis: host cell death and inflammation. Nature Reviews Microbiology 7, 99–109, doi:10.1038/nrmicro2070 (2009). 19148178 PMC2910423

[158] Wang W. & Zhang T. Caspase-1-Mediated Pyroptosis of the Predominance for Driving CD4 $$^{+}$$ +. Bulletin of Mathematical Biology 80, 540–582, doi:10.1007/s11538-017-0389-8 (2018). 29349609

[159] He Y., Hara H. & Núñez G. Mechanism and Regulation of NLRP3 Inflammasome Activation. Trends in Biochemical Sciences 41, 1012–1021, doi:10.1016/j.tibs.2016.09.002 (2016). 27669650 PMC5123939

[160] Zalinger Z. B., Elliott R. & Weiss S. R. Role of the inflammasome-related cytokines Il-1 and Il-18 during infection with murine coronavirus. Journal of NeuroVirology 23, 845–854, doi:10.1007/s13365-017-0574-4 (2017). 28895072 PMC5726909

[161] O’Shea J. J., Gadina M., Siegel R. M. & Farber J. in Rheumatology (Sixth Edition) (eds Hochberg Marc C. ) 99–112 (Mosby, 2015).

[162] Han S. Lipopolysaccharide Primes the NALP3 Inflammasome by Inhibiting Its Ubiquitination and Degradation Mediated by the SCFFBXL2 E3 Ligase. Journal of Biological Chemistry 290, 18124–18133, doi:10.1074/jbc.m115.645549 (2015). PMC4505057 26037928

[163] Moors M. A. & Mizel S. B. Proteasome-mediated regulation of interleukin-1β turnover and export in human monocytes. Journal of Leukocyte Biology 68, 131–136, doi:10.1189/jlb.68.1.131 (2000). 10914500

[164] Bagaev A. V. Elevated pre-activation basal level of nuclear NF-κB in native macrophages accelerates LPS-induced translocation of cytosolic NF-κB into the cell nucleus. Scientific reports 9, 4563 (2019). < 10.1038/s41598-018-36052-5>. 30872589 PMC6418260

[165] de Vasconcelos N. M., Van Opdenbosch N., Van Gorp H., Parthoens E. & Lamkanfi M. Single-cell analysis of pyroptosis dynamics reveals conserved GSDMD-mediated subcellular events that precede plasma membrane rupture. Cell Death & Differentiation 26, 146–161, doi:10.1038/s41418-018-0106-7 (2019). 29666477 PMC6294780

[166] Martín-Sánchez F. Inflammasome-dependent IL-1β release depends upon membrane permeabilisation. Cell Death & Differentiation 23, 1219–1231, doi:10.1038/cdd.2015.176 (2016). 26868913 PMC4946890

[167] Yang F. Myocardial Infarction and Cardiac Remodelling in Mice. Experimental Physiology 87, 547–555, doi:10.1113/eph8702385 (2002). 12481929

[168] Sakai N. & Tager A. M. Fibrosis of two: Epithelial cell-fibroblast interactions in pulmonary fibrosis. Biochimica et Biophysica Acta (BBA) - Molecular Basis of Disease 1832, 911–921, doi:10.1016/j.bbadis.2013.03.001 (2013). 23499992 PMC4041487

[169] Wahl S. M. Transforming growth factor type beta induces monocyte chemotaxis and growth factor production. Proceedings of the National Academy of Sciences 84, 5788, doi:10.1073/pnas.84.16.5788 (1987). PMC298948 2886992

[170] Jin Y.-F., Han H.-C., Berger J., Dai Q. & Lindsey M. L. Combining experimental and mathematical modeling to reveal mechanisms of macrophage-dependent left ventricular remodeling. BMC Systems Biology 5, 60, doi:10.1186/1752-0509-5-60 (2011). 21545710 PMC3113236

[171] Zhang H., Ahmad M. & Gronowicz G. Effects of transforming growth factor-beta 1 (TGF-β1) on in vitro mineralization of human osteoblasts on implant materials. Biomaterials 24, 2013–2020, doi:10.1016/S0142-9612(02)00616-6 (2003). 12628820

[172] R. A. F. Jr Nanomedicine, Volume I: basic capabilities., (Landes Bioscience, 1999).

[173] Trepat X., Chen Z. & Jacobson K. in Comprehensive Physiology (2012). 10.1002/cphy.c110012 PMC4457291 23720251

[174] Darby I. A., Bisucci T., Hewitson T. D. & MacLellan D. G. Apoptosis is increased in a model of diabetes-impaired wound healing in genetically diabetic mice. The International Journal of Biochemistry & Cell Biology 29, 191–200, doi:10.1016/S1357-2725(96)00131-8 (1997). 9076954

[175] Ceresa M., Olivares A. L., Noailly J. & González Ballester M. A. Coupled Immunological and Biomechanical Model of Emphysema Progression. Frontiers in Physiology 9, doi:10.3389/fphys.2018.00388 (2018). PMC5917021 29725304

[176] Toapanta F. R. & Ross T. M. Impaired immune responses in the lungs of aged mice following influenza infection. Respir Res 10, 112, doi:10.1186/1465-9921-10-112 (2009). 19922665 PMC2785782

[177] Zhu J. & Paul W. E. Peripheral CD4+ T-cell differentiation regulated by networks of cytokines and transcription factors. Immunological Reviews 238, 247–262, doi:10.1111/j.1600-065X.2010.00951.x (2010). 20969597 PMC2975272

[178] Lucas C. Kinetics of antibody responses dictate COVID-19 outcome. medRxiv, 2020.2012.2018.20248331, doi:10.1101/2020.12.18.20248331 (2020).

[179] Mayadas T. N. & Cullere X. Neutrophil β2 integrins: moderators of life or death decisions. Trends in Immunology 26, 388–395, doi:10.1016/j.it.2005.05.002 (2005). 15922663

[180] Reznikov K. Clustering of apoptotic cells via bystander killing by peroxides. The FASEB Journal 14, 1754–1764, doi:10.1096/fj.99-0890com (2000). 10973925

[181] Wright H. L., Moots R. J., Bucknall R. C. & Edwards S. W. Neutrophil function in inflammation and inflammatory diseases. Rheumatology 49, 1618–1631, doi:10.1093/rheumatology/keq045 (2010). 20338884

[182] Sullivan H. C. & Roback J. D. Convalescent Plasma: Therapeutic Hope or Hopeless Strategy in the SARS-CoV-2 Pandemic. Transfusion Medicine Reviews 34, 145–150, doi:10.1016/j.tmrv.2020.04.001 (2020). 32359788 PMC7179481

[183] Kyriakidis N. C., López-Cortés A., González E. V., Grimaldos A. B. & Prado E. O. SARS-CoV-2 vaccines strategies: a comprehensive review of phase 3 candidates. npj Vaccines 6, 28, doi:10.1038/s41541-021-00292-w (2021). 33619260 PMC7900244

[184] Organization W. H. WHO Director-General’s opening remarks at the media briefing on COVID-19 – 11 March 2020, <https://www.who.int/dg/speeches/detail/who-director-general-s-opening-remarks-at-the-media-briefing-on-covid-19---11-march-2020> (2020).

[185] Grifoni A. Targets of T Cell Responses to SARS-CoV-2 Coronavirus in Humans with COVID-19 Disease and Unexposed Individuals. Cell 181, 1489–1501.e1415, doi:10.1016/j.cell.2020.05.015 (2020). 32473127 PMC7237901

[186] Sette A. & Crotty S. Pre-existing immunity to SARS-CoV-2: the knowns and unknowns. Nature Reviews Immunology 20, 457–458, doi:10.1038/s41577-020-0389-z (2020). PMC7339790 32636479

[187] Liao M. Single-cell landscape of bronchoalveolar immune cells in patients with COVID-19. Nature Medicine 26, 842–844, doi:10.1038/s41591-020-0901-9 (2020). 32398875

