# Supplementary material for: Iterative community-driven development of a SARS-CoV-2 tissue simulator
Source: bioRxiv. 2021 Apr 29:2020.04.02.019075. Preprint. [Version 4] doi: 10.1101/2020.04.02.019075 (PMC7239052; doi:10.1101/2020.04.02.019075)
Supplement: Supplement 1 [file media-1.pdf]

Supplementary material for Iterative community-driven development of a SARS-CoV-2 tissue simulator

Michael Getz[1,**], Yafei Wang[1,**], Gary An[2,*], Maansi Asthana[23,*], Andrew Becker[2,*], Chase Cockrell[2,*], Nicholson Collier[3,4,*], Morgan Craig[5,6*], Courtney L. Davis[7,*], James R. Faeder[8,*], Ashlee N. Ford Versypt[9,10,11*], Tarunendu Mapder[22*], Juliano F. Gianlupi[1,*], James A. Glazier[1,*], Sara Hamis[12,*], Randy Heiland[1,*], Thomas Hillen[13,*], Dennis Hou[14,*], Mohammad Aminul Islam[9,11*], Adrianne L. Jenner[5,6,*], Furkan Kurtoglu[1,*], Caroline I. Larkin[8,*], Bing Liu[8,*,†], Fiona Macfarlane[12,*], Pablo Maygrundter[15,*], Penelope A Morel[16,*], Aarthi Narayanan[17,*], Jonathan Ozik[3,4,*], Elsje Pienaar[18,*], Padmini Rangamani[19,*], Ali Sinan Saglam[8,*], Jason Edward Shoemaker[20,*], Amber M. Smith[21,*], Jordan J.A. Weaver[20,*], Paul Macklin[1,***]

[1] Department of Intelligent Systems Engineering, Indiana University. Bloomington, IN USA
[2] The University of Vermont Medical Center, Burlington, VT USA
[3] Decision and Infrastructure Sciences, Argonne National Laboratory. Lemont, IL USA
[4] Consortium for Advanced Science and Engineering, University of Chicago. Chicago, IL USA
[5] Department of Mathematics, University of Montreal. Montreal, QC Canada
[6] CHU Sainte-Justine Research Centre, Montreal, QC Canada
[7] Natural Science Division, Pepperdine University, Malibu, CA USA
[8] Department of Computational and Systems Biology, University of Pittsburgh. Pittsburgh, PA USA
[9] School of Chemical Engineering, Oklahoma State University, Stillwater, OK USA
[10] Oklahoma Center for Respiratory and Infectious Diseases, Oklahoma State University, Stillwater, OK USA
[11] Department of Chemical and Biological Engineering, University at Buffalo, The State University of New York, Buffalo, NY USA
[12] School of Mathematics and Statistics, University of St Andrews, St Andrews, Scotland
[13] Department of Mathematical and Statistical Sciences, University of Alberta. Edmonton, AB Canada
[14] Department of Mathematics, Rutgers University. New Brunswick, NJ USA
[15] Citizen scientist. Austin, TX USA
[16] Department of Immunology, University of Pittsburgh. Pittsburgh, PA USA
[17] National Center for Biodefense and Infectious Disease, George Mason University. Manassas, VA USA
[18] Weldon School of Biomedical Engineering, Purdue University. West Lafayette, IN USA
[19] Department of Mechanical and Aerospace Engineering, University of California. San Diego, CA USA
[20] Department of Chemical and Petroleum Engineering, University of Pittsburgh. Pittsburgh, PA USA
[21] Department of Pediatrics, University of Tennessee Health Science Center, Memphis, TN USA
[22] Division of Clinical Pharmacology, Department of Medicine, Indiana University School of Medicine, Indianapolis, IN, USA
[23] Agricultural and Biological Engineering, Purdue University. West Lafayette, IN USA

* contributed equally to this work
** equally contributing lead authors
† in memoriam
*** corresponding author: macklinp@iu.edu, @MathCancer

Previous Version Results

Version 1 (March 25-March 31, 2020)

Version 1 was designed as proof of concept rapid prototype to capture essential (but highly simplified) elements of viral endocytosis, protein synthesis, viral assembly, release, and diffusion to infect other cells. The model was tailored to RNA viruses on a tissue monolayer (modeled as a layer of epithelium over a basement membrane). This version was kept deliberately simple to create an early starting framework to help coalesce community feedback and contributions. It was also designed to test the use of interactive cloud-hosted models to help accelerate feedback by virologists and other domain experts through live demos.

The proof of concept model was created by the overall leads (Macklin, Heiland, Wang) while assembling the modeling coalition as an initial starting point and feasibility test for rapid prototyping. Feedback on this version drove the formulation of the design protocols reported above.

Submodels

The Version 1 model includes the following submodel components:

- **T:** tissue (which contains epithelial and other cells)
- **V:** viral endocytosis, replication, and exocytosis responses
- **VR:** cell response to viral replication, including cell death and IFN synthesis
- **E:** epithelial cell (incorporates V and VR).

The overall model components are summarized in **Fig 1.1**.

Biological hypotheses

In this proof of concept prototype, we modeled a simplified set of biological hypotheses:

1.T.1 Virus diffuses in the microenvironment with low diffusion coefficient
1.T.2 Virus adhesion to a cell stops its diffusion (acts as an uptake term)

1.V.1 Adhered virus undergoes endocytosis and then becomes uncoated
1.V.2 Uncoated virus (viral contents) lead to release of functioning RNA
1.V.3 RNA creates protein at a constant rate, unless it is degraded
1.V.4 Protein is transformed to an assembled virus state
1.V.5 Assembled virus is released by the cell

1.VR.1 As a proxy for viral disruption of the cell, the probability of cell death increases with the total number of assembled virions
1.VR.2 Apoptosed cells lyse and release some or all of their contents

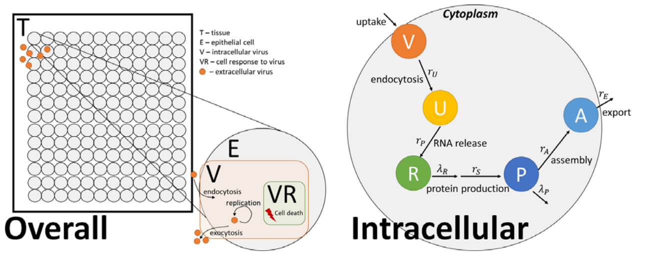

Fig. 1.1: Version 1 model schematic: ***Left:*** In the overall model structure, a ***tissue component*** **(T)** contains multiple ***epithelial cells*** **(E)**. Viral particles diffuse through this domain and can enter the cells (by adhering as an uptake term) or be exported by cells. Each cell includes an intracellular ***virus*** model **(V)** for viral replication kinetics and a ***viral response*** model **(VR)** which uses a pharmacodynamic model to control cell apoptotic response to viral load. ***Right:*** Each epithelial cell individually models its viral kinetics: adhered virions complete endocytosis, are uncoated, functionalize their RNA, synthesize viral components, and assemble them into virions that are subsequently exported to the external tissue compartment (T).

(In the above, X.C.Y denotes prototype X, model component C, biological hypothesis Y, allowing us to easily refer to any individual hypothesis or assumption in discussion and community feedback.) In the next version of this

model, we will use the design document protocols for each of these components.

Unit tests

The first prototype should demonstrate the following behaviors for a single cell infected by a single virion:

- The virion progresses to the uncoated state.
- The uncoated virion progresses to the RNA state.
- With export and death off, RNA produces protein.
- With export and death turned off, protein produces and accumulates assembled virus (linearly).
- With export off and death on, cell undergoes apoptosis with increasing likelihood as assembled virus accumulates.
- With export on and death on, surrounding cells get infected and create virion.
- Cells nearest the initial cell are infected first.
- Apoptosis is most frequent nearest to the initial infected cell.

Translation to mathematics, rules, and model components

Other implementation notes

To differentiate between incoming imported and exported virions within the computational implementation, we modeled two diffusing fields (for extracellular concentrations of V and A). However, the models only require extracellular V. At the end of each computational step (advancing by one diffusional time step), we iterate through each voxel and transfer all of the extracellular diffusing A to V. We also created diffusing fields for uncoated virions, RNA, and viral proteins, although these were removed from later model versions.

Software release

The core model associated with the v1 prototype is Version 0.1.3. The nanoHUB app associated with the v1 prototype is Version 1.0. GitHub releases and Zenodo snapshots are given in the Appendix.

Cloud-hosted model

We rapidly created and deployed a cloud-hosted model with an interactive web-based GUI running on nanoHUB (nanohub.org) using xml2jupyter Version 1.1[1]. The web-hosted model can be run at:

https://nanohub.org/tools/pc4COVID-19.

This workflow uses a Python script that converts a PhysiCell configuration file (in XML) into a Jupyter notebook and adds additional Python modules for the GUI. The automated process of converting a standalone PhysiCell model into an interactive Jupyter notebook version (a GUI) takes just a few minutes. The resulting GitHub repository is shared with the nanoHUB system administrators who install it for testing as an online, executable model (an "app"). After we perform usability and other testing and finalize documentation, it is published and becomes available for public use. The whole process (including the initial development of the core PhysiCell model) took less than 12 hours for the Version 1 GUI on nanoHUB (**Fig. 1.2**).

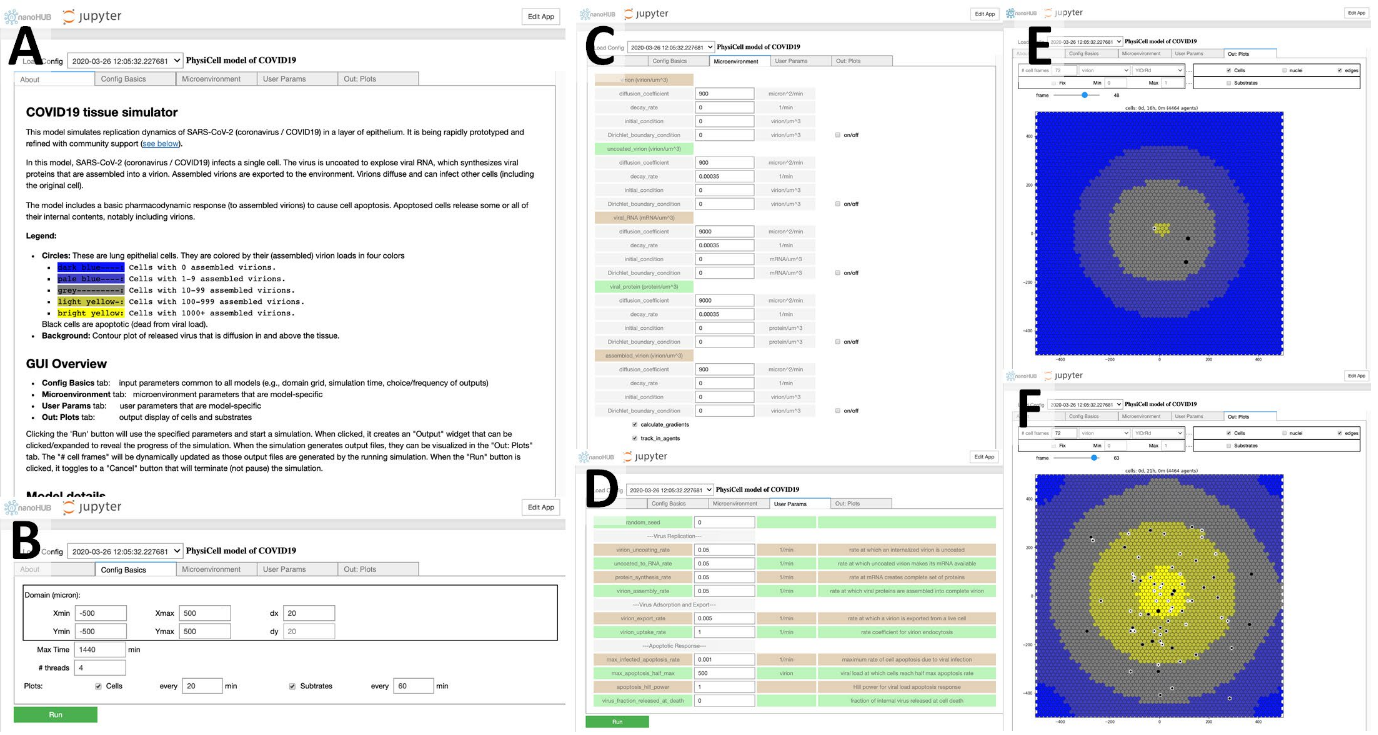

Fig. 1.2: Version 1 on nanoHUB: A. The *About* tab includes background information on the model, run directions, and a legend. **B.** The *Config basics* tab allows users to set the domain size. (These should be not be edited in this version.) **C.** The *Microenvironment* tab lets users set diffusion and decay coefficients, as well as initial and boundary conditions. The track_in_agents option must be enabled for the model to work correctly. **D.** The *User Params* tab sets key model parameters. **E-F.** Sample outputs in the *Out: Plots* tab. Users can scroll the "frame" tab to advance the simulation time and choose whether to plot the cells, a diffusing substrate, or both. These plots display a sheet of epithelium (circles are individual cells). Brighter yellow cells contain more assembled virion. Black cells are apoptotic, and clear spaces show tissue damage (where cells have apoptosed and have been removed.) This version of the nanoHUB model was released at DOI 10.21981/19BB-HM69.

Model behavior: what does the current version teach us?

Except as noted below, all simulation results use the v1 model default parameters, which are supplied in the XML configuration parameter file of the version 0.1.2 core model repository.

In all plots, dark blue cells have 0 assembled virus, pale blue cells have 1-9 assembled virions, grey cells have 10-99 assembled virions, light yellow cells have 100-999 assembled virions, and bright yellow cells contain 1000 or more assembled virions. Black cells are apoptotic, and white spaces show regions devoid of cells (extensive tissue damage). See the legend in **Fig. 1.2** (A) and the caption in **Fig. 1.3** (A).

Behavior with default parameters

Running the overall model (with virus release turned on and off as appropriate for the respective unit tests) shows that the v1 prototype satisfies all the qualitative unit tests. A single cell is infected with a virion in the center of the tissue. Over time, the virion is uncoated to create functionalized RNA, which is synthesized to viral proteins and assembled to functional virus. The graphical output shows this center cell turning to a bright yellow as assembled virions accumulate. By enabling the substrate plot, we can see the diffusive field of virions first has zero concentration (no virions have been released), but as the first cell's viral production increases, it releases virus particles that begin diffusing into the domain (**Fig. 1.3 A**).

Over time, neighboring cells also become infected and progress towards a higher viral load (increasingly bright shades of yellow). The infection propagates outward from the initially infected cell into the remaining tissue. As each cell's viral load (here measured as number of assembled virions) increases, the viral response model calculates the increasing effect *e*, and cells have greater probability of apoptosis. Cells nearest to the initial site of infection apoptose earliest. As these cells degrade, they are removed from the simulation, leading to the creation

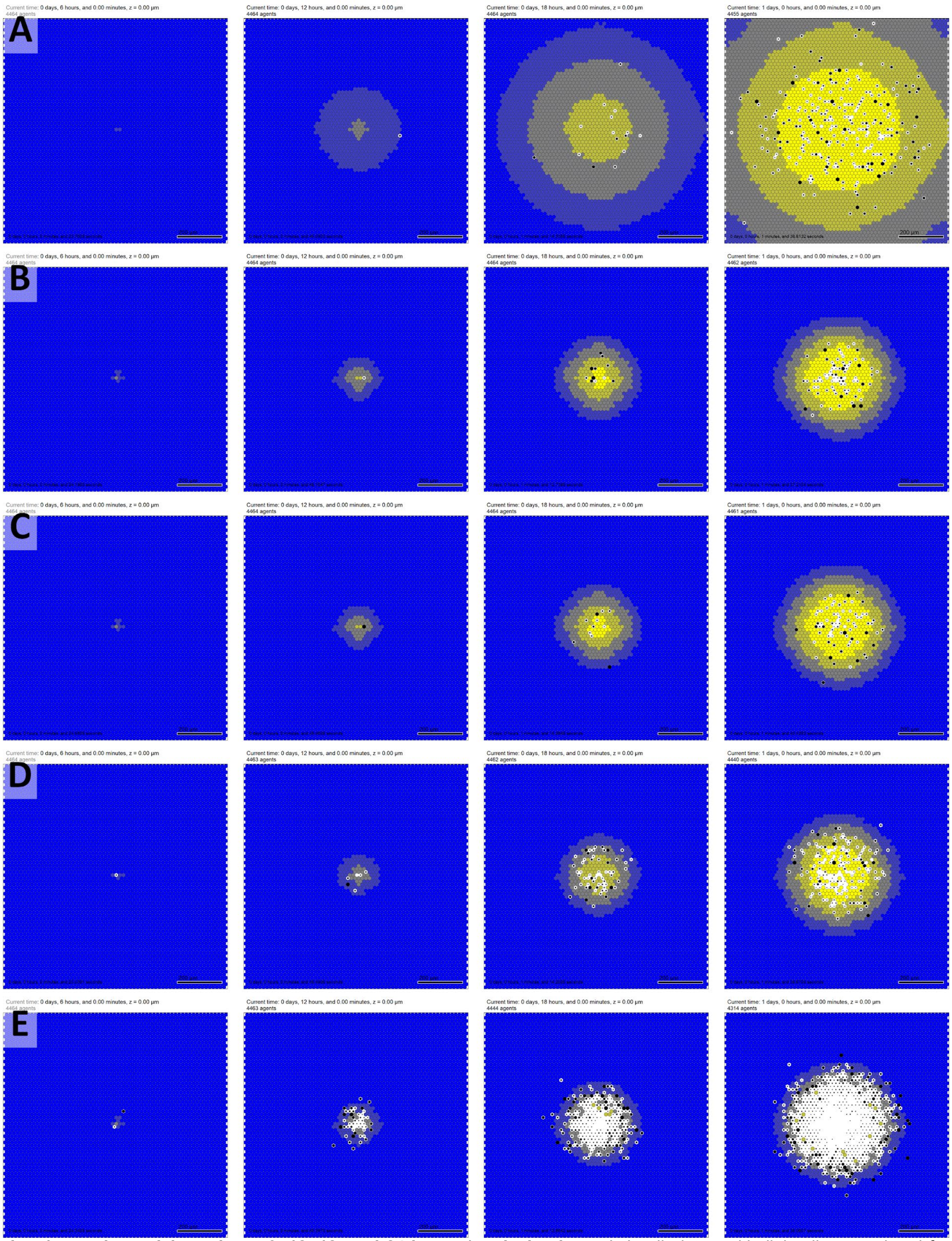

Fig. 1.3: Version 1 sample model results at 6, 12, 18, and 24 hours (vertical columns). In all plots, epithelial cells are colored from blue (no assembled virions) to bright yellow (1000 or more virions). Black cells are apoptotic, and white regions show damaged tissues where apoptotic cells have degraded to expose (unmodeled) basement membrane. Bar: 200 μm. **A.** Simulation time course for the default parameters. Note the spread of the infection from an initial infected cell at the center, with apoptotic death events focused near the center. **B.** Decreasing the diffusion coefficient of virions by a factor of 10 drastically reduces the rate of spread, although focusing exocytosed virions in a smaller diffusion distance increases the number of virions infecting nearby cells, leading to faster apoptosis. **C.** Allowing apoptosed cells to release their assembled virions at lysis had a negligible effect for these parameters, given the dominant effects of releasing virions throughout the cell survival times. **D.** Decreasing the tolerance (half max) of cells to assembled virions prior to apoptosis accelerates tissue damage but does not drastically accelerate the spread of the infection. **E.** Increasing the apoptosis rate (or decreasing the survival time) for infected cells drastically increases tissue degradation.

of a degraded, cell-free region near the center of the tissue. This degraded region spreads outwards from the

initial site of infection over time.

See **Fig. 1.3 A** for a simulation with default parameters. The nanoHUB distribution of this model takes approximately 60-90 seconds to execute.

Impact of the virion diffusion coefficient

We tested the effect of the viral diffusion coefficient by reducing it from 900 μm^2/min to 90 μm^2/min. Because the viral particles spread more slowly after their release, the overall spread of the infection is slowed (**Fig. 1.3 B**). We left D = 90 μm^2/min for all subsequent investigations of the v1 model.

Impact of the viral release at cell death

We tested the effect of releasing all assembled viral particles at the time of cell death by setting $f_{\text{release}} = 1$. For this set of model parameters, the release of assembled virions had a negligent impact of the overall spread of infection: Compare the final frame of **Fig. 1.3 B** (no release: $f_{\text{release}} = 0$) to **Fig. 1.3 C** (complete release: $f_{\text{release}} = 1$). This is because cells release far more virions during their infected lifetimes, so the effect is dominant over the one-time release of virions at cell death. We expect this behavior would change if the cells exocytosed virions more slowly.

Impact of the cell tolerance to viral load

We decreased the cell tolerance to viral load by decreasing the A_H of Equation **Error! Reference source not found.** from 500 virions to 10, while leaving $f_{\text{release}} = 1$. As expected, cell death and tissue damage occurred much more quickly under these parameters (**Fig. 1.3 D**). Interestingly (and contrary to intuition), this did not significantly alter the rate at which the infection spread through the tissue. Compare the final frame of **Fig. 1.3 C** (higher tolerance to viral load) to **Fig. 1.3 D** (lower tolerance to viral load). This shows the importance of creating spatiotemporal models of viral replication in tissues, as the balance of competing processes can lead to unexpected dynamics at the tissue, organ, and organism levels.

Impact of the cell survival time under high viral loads

We decreased the cell tolerance to viral load further by decreasing the mean cell survival time under high viral loads, which is equivalent to increasing the maximum apoptosis rate $r_{\max}$. Following prior analyses[2,3], $1/r_{\max}$ is the mean expected survival time as $A \to \infty$. We increased $r_{\max}$ from 0.001 min^{-1} (1000 minute expected lifetime at high loads) to 0.01 min^{-1} (100 minute expected lifetime at high viral loads). This drastically accelerated the rate of tissue damage, leaving much more basement membrane (the assumed surface under the epithelial monolayer) exposed (**Fig. 1.3 E**). In a later version of this model framework, we would expect this to lead to earlier onset of fluid leakage, edema, and ultimately adverse respiratory outcomes such as ARDS. Interestingly, this did not significantly increase the rate of spread of the infection. Compare the final frame of **Fig. 1.3 D** (higher tolerance to viral load) to **Fig. 1.3 E** (lower tolerance to viral load).

Selected feedback from domain experts and the community

We gathered feedback from the multidisciplinary community, several of whom joined the coalition for future work. We summarize the feedback below.

A virologist noted that more detail on endocytosis, viral uncoating, and synthesis would expose more actionable points in the replication cycle. Preliminary SARS-CoV-2 experiments in her laboratory suggest that the time course (and thus general order of magnitude of rate parameters) is very similar to Venezuelan equine encephalitis virus (VEEV) dynamics measured earlier[4,5]. The exponential progression matches observations: the first cell is infected with one virion and so at first produces virus slowly, but neighboring cells can be infected with multiple virions and thus create virus particles more quickly.

A community member identified typographical errors in the original documentation but verified that that mathematics in the C++ implementation were not affected. He emphasized the importance of implementing RNA decay (as a rate limiting step in virus replication) and the importance of integrating ACE2 receptor trafficking (as a rate

limiting step in virus adhesion and endocytosis).

A mathematician noted the potential to simplify the model by removing the diffusing *U, R,* and *P* fields and reported bugs in the initialization (where no cells are initially infected for some domain sizes, due to hard-coding of the initial seeding). Other mathematicians emphasized the importance of varying virion "uptake" with ACE2 receptor availability and hence the need to integrate receptor trafficking.

A mathematical biologist noted prior work on other respiratory viruses will help estimate parameters and build initial immunologic regulation models. Lung pathology and disease severity are closely tied to the immunologic reaction, and prior data and images from influenza will help with calibrating spatial considerations. She expects animal and drug data available for SARS-CoV-2 in the coming months. She noted the importance of distinguishing between mild and severe infections and ARDS. Matching the output to data will be imperative, with one quick possibility to make this match data and distinguish between possibilities is to plot the resulting viral load. She suggested that it would be helpful to show multi focal points of initial infection seeding (possibly of different initial seeding size) that merge together over time, which would match observations of lung histology. Future work will have a better impact if the models uses a true lung tissue geometry with immune cells limiting the peripheral spread. The current model seems more relevant to in vitro growth of a single plaque, which may be scrutinized.

A quantitative systems pharmacologist pointed out the need for clearer scoping and diagrams to clearly lay out the design of each submodel component. We will need procedures to choose future incorporations and changes of scope. He also pointed out the need to understand what happens if you bind up a lot of ACE2 with receptor; there are early insights online[6].

A bioengineer with tissue damage and inflammation expertise noted that the diffusion coefficient of 900 μm^2/min = 15 μm^2/s = 1.5e-11 m^2/s is not particularly small; prior analyses[7] considered virion diffusion in an lung epithelial monolayer for influenza with D = 3.18e-15m^2/s estimating from experimental data. The virions for SARS-CoV-2 could be more mobile though; it is uncertain. There are data[8] about the diffusion coefficient for albumin in tissue being on the order of 10-50 μm^2/s. She stated that it makes sense for a virion (radius of 25-100 nm) to move more slowly than a protein with radius < 5 nm unless "diffusive transport" in the model is encompassing an active or facilitated transport mode beyond just classic diffusion. She also noted that her laboratory has looked a lot at the renin-angiotensin-system systemically and in kidneys: the kinetics of AngII, ACE, and ACE2 in the lungs would be of interest for connecting the next iteration of the ACE2 receptor model to connect to ARDS. Pfizer may also have relevant related models.

A mathematical biologist with expertise in infectious diseases noted that the model could study immune responses and the impact of mucosal structure in future versions. She suggested quantifying damage or disease metrics. She also noted that ultimately it would be useful to note which parameter estimates might be species-specific and which are not, to be able to switch between experimental and clinical systems, e.g., it is worth recording if current estimates are from human, macaque, etc. She also noted that it may be important to determine if apoptotic cells are replaced or if there is permanent damage (in the model). If the model is run longer, it would be worthwhile to translate the visual sense of damage to a quantitative metric.

An independent team of clinically-focused modelers noted their work on modeling immune expansion in "off screen" lymph nodes and offered to link their model to our immune infiltration functions.

A mathematical biologist with a focus on model and data standards noted the need for clearly specifying each model's assumptions, inputs, and outputs to drive robust parallel development. He noted that it is critical to consider information flow between submodels and revise these data flows as the iterations proceed. He suggested that we state separate execution of submodels as a key design goal to support parallel development. Lastly, he noted that software should be released in conjunction with validation data and methodologies.

Core team discussion and priorities for v2

The core team met by virtual conference on April 1, 2020 to discuss the first preprint, model results, and feedback. The core team set as priorities (1) to formalize design specifications for each individual model component and interfaces between components, (2) form teams responsible for each component, (3) focus v2 development on refactoring into this modular format, (3) begin development of the submodels, and (4) begin refine parameter

estimates. The clearer specification and organization of submodels was the top priority. As time permits, it was also viewed as important to begin a receptor trafficking model.

The core team agreed to keep working via the dedicated Slack workspace to rapidly coalesce on the submodel teams. Each subteam has a separate channel in the workspace.

Version 2 (April 1-May 9, 2020)

Version 2 incorporated key v1 feedback, with a focus on introducing a more modular design, improving default model parameters, better initialization options, and a new ACE2 receptor trafficking submodel. This design cycle lasted longer due in part to work spent on subteam organization. As with Version 1, the Version 2 model was developed by the overall leads (Macklin, Heiland, Wang) to refine key model infrastructure for the forming subteams. (See main text section **Three main phases of community-driven development**.)

Version 2 also began work to test the design documents that were first discussed by the core team during the v1 model feedback. The interactive nanoHUB model included new usability refinements, notably an option to animate the model outputs.

Model changes

The v2 model was expanded to include the following submodel components (**Fig. 2.1**):

- **T:** tissue (which contains epithelial and other cells)
- **RT:** ACE2 receptor trafficking (including virus endocytosis)
- **V:** viral endocytosis, replication, and exocytosis responses
- **VR:** cell response to viral replication, including cell death and IFN synthesis
- **E:** epithelial cell (incorporates RT, V and VR).

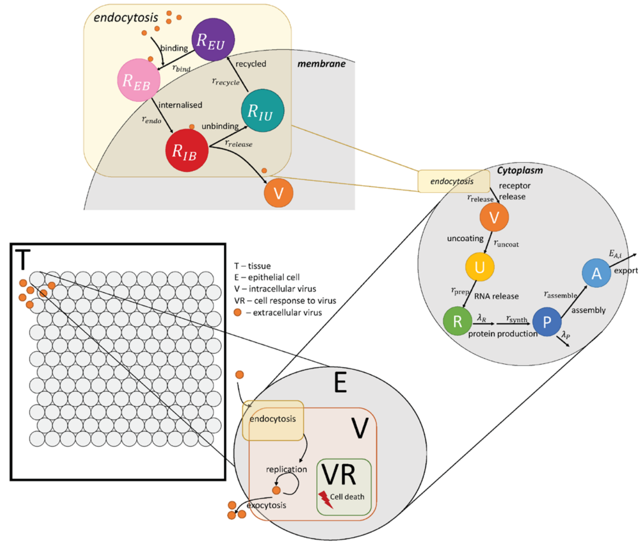

Fig. 2.1: Version 2 model schematic: In the overall model structure, a ***tissue component*** **(T)** contains multiple ***epithelial cells*** **(E)**. Viral particles diffuse through this domain and can enter the cells (by adhering as an uptake term) or be exported by cells. Each cell includes an ACE2 ***receptor trafficking*** model (**RT**) where virus can bind an unoccupied surface receptor, which is endocytosed to release its virion and eventually return to the surface. Each cell's ***virus lifecycle*** model **(V)** simulates viral replication kinetics, and its ***viral response*** model **(VR)** uses a dose-response model to control cell apoptotic response to viral load.

Based on community feedback, the default virion diffusion coefficient was reduced by a factor of 10 to 90 μm^2/min. We may reduce this parameter further based upon oncolytic virus therapy modeling experience by Morgan Craig and Adrianne Jenner.

Biological hypotheses

The v2 model was similar to v1 with a simplified set of biological hypotheses:

2.T.1 Virus diffuses in the microenvironment with low diffusion coefficient
2.T.2 Virus adhesion to a cell stops its diffusion (acts as an uptake term)

2.RT.1 Virus adheres to unbound external ACE2 receptor to become external (virus)-bound ACE2 receptor
2.RT.2 Bound external ACE2 receptor is internalized (endocytosed) to become internal bound ACE2 receptor
2.RT.3 Internalized bound ACE2 receptor releases its virion and becomes unbound internalized receptor; the released virus is available for use by the viral lifecycle model **V**

2.RT.4 Internalized unbound ACE2 receptor is returned to the cell surface to become external unbound receptor
2.RT.5 Each receptor can bind to at most one virus particle

2.V.1 Internalized virus (previously released in 2.RT.3) is uncoated
2.V.2 Uncoated virus (viral contents) lead to release of functioning RNA
2.V.3 RNA creates viral protein at a constant rate unless it degrades
2.V.4 Viral protein is transformed to an assembled virus state
2.V.5 Assembled virus is released by the cell (exocytosed)

2.VR.1 As a proxy for viral disruption of the cell, the probability of cell death increases with the total number of assembled virions
1.VR.2 Apoptosed cells lyse and release some or all of their contents

(In the above, X.C.Y denotes prototype X, model component C, biological hypothesis Y, allowing us to easily refer to any individual hypothesis or assumption in discussion and community feedback.) In the next version of this model, we will use the design document protocols for each of these components.

Unit tests

The v2 prototype had no changes in qualitative unit tests; once the ACE2 receptor trafficking model works correctly, the model will behave as in v1.

Translation to mathematics, rules and model components

Cell response (Viral response submodel ***VR***)

There were no changes from the v1 model.

Initialization

In v2, we added the option to specify the *multiplicity of infection (MOI):* the ratio of initial virions to number of epithelial cells. These virions are placed randomly in the extracellular space. We use a default MOI = 0.01 to model a fine mist of virions landing on the tissue. Users can also set an option to only infect the centermost cell, which sets $V = 1$ for that cell.

Refined parameter estimates

Detailed experimental characterization of ACE2 receptor trafficking in SARS-CoV[9] permits an initial estimation of key model parameters. This experimental work reported that endocytosed receptors were observed in 3 hours post infection, and that 10 hours later (13 hours elapsed time), receptors were observed in vesicles. This estimates the time scale of binding and endocytosis to be on the order of 3 hours, and that virion release occurs on the order of 10 hours. Thus:

$$\frac{1}{r_{\text{bind}} R_{EU}(0)} + \frac{1}{r_{\text{endo}}} \sim 3 \text{ hours} \tag{1}$$

and

$$\frac{1}{r_{\text{release}}} \sim 10 \text{ hours.} \tag{2}$$

Supposing that binding is relatively fast compared to endocytosis, we set $\frac{1}{r_{\text{bind}} R_{EU}(0)} \sim 1 \text{ min}$, and so $\frac{1}{r_{\text{endo}}} \sim$

3 hours. Recycled receptors were observedwithin 14 hours (1 hour after the appearance of endocytosed receptors), so we set $\frac{1}{r_{\text{recycle}}} \sim 1$ hour. Assuming there are 1,000 to 10,000 ACE2 receptors per cell, we set the parameters (to order of magnitude) at

$$r_{\text{bind}} = 0.001 \text{ min}^{-1} \quad (3)$$

$$r_{\text{endo}} = 0.01 \text{ min}^{-1} \quad (4)$$

$$r_{\text{release}} = 0.001 \text{ min}^{-1} \quad (5)$$

$$r_{\text{recycle}} = 0.01 \text{ min}^{-1} \quad (6)$$

$$r_{\text{bind}} = 0.001 \text{ min}^{-1} \quad (7)$$

The report observed expression of viral proteins by 18 hours (5 hours after viral release from endocytosed ACE2 receptors). Assuming that $r_{\text{uncoat}} \sim r_{\text{prep}} \sim r_{\text{synth}}$, each parameter has magnitude 0.01 min^{-1}. We similarly set $r_{\text{assemble}} = r_{\text{exo}} = 0.01 \text{ min}^{-1}$ in the v2 model.

Other implementation notes

To differentiate between incoming imported and exported virions within the computational implementation, we modeled two diffusing fields (for extracellular concentrations of *V* and *A*). However, the models only require extracellular *V*. At the end of each computational step (advancing by one diffusional time step), we iterate through each voxel and transfer all of the extracellular diffusing *A* to *V*. We also created diffusing fields for uncoated virions, RNA, and viral proteins, although these were removed from later model versions.

By setting the virus uptake rate *U* as noted above, PhysiCell (via BioFVM) automatically removes the correct amount of virions from the extracellular diffusing field and places them in an internalized virus particle variable *n*. By PhysiCell's automated mass conservation:

$$\Delta n = \Delta t \, r_{\text{bind}} n_V R_{EU} = \Delta t \, U_i V_i \rho. \quad (8)$$

If *n* was previously set to zero, then the current value of *n* represents Δn. By assumption 2.RT.5, Δn is equal to the change in the number of external virus-bound receptors (one virion per receptor). Thus, these receptors (Δn) represent the net increase in bound external receptors. So at each time step, we:

1) Increase R_{EB} by *n*
2) Decrease R_{EU} by *n*
3) Set *n* = 0 (because these virions have been "delivered" to the receptor trafficking model)

Software release

The core model associated with the v2 prototype is Version 0.2.1. The nanoHUB app associated with the v2 prototype is Version 2.1. GitHub releases and Zenodo snapshots are given in the Appendix.

The cloud-hosted interactive model can be run at https://nanohub.org/tools/pc4COVID-19.

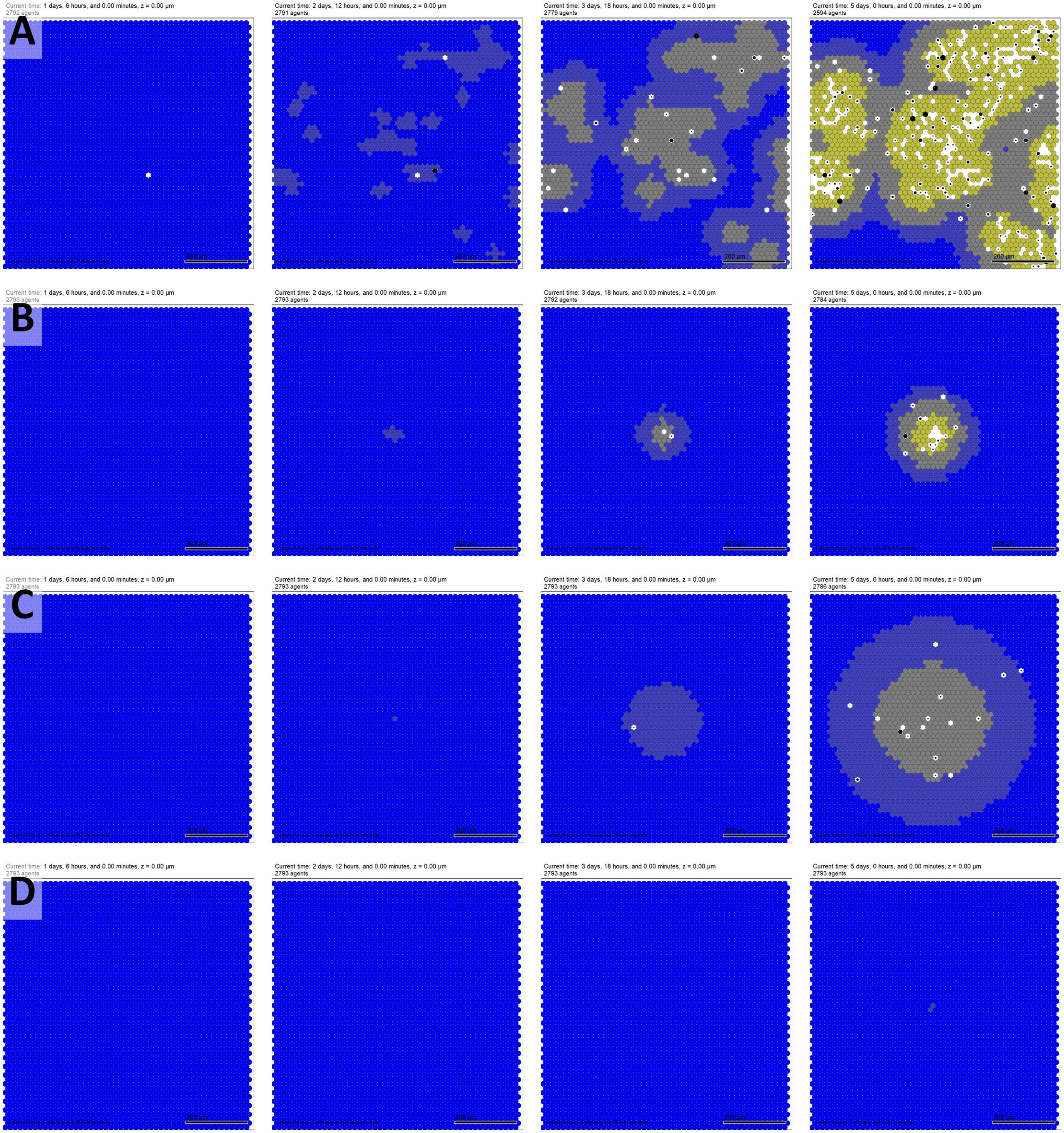

Fig. 2.2: Version 2 sample model results at 30, 60, 120, and 180 hours. In all plots, epithelial cells are colored from blue (no assembled virions) to bright yellow (1000 or more virions). Black cells are apoptotic, and white regions show damaged tissues where apoptotic cells have degraded to expose (unmodeled) basement membrane. Bar: 200 μm. **A.** Simulation time course using the new initialization with an MOI (multiplicity of infection) of 0.01. As virions land randomly on the tissue, they initiate multiple infections that spread and merge. **B.** After infecting a single cell (with the new default parameters), the infected region (plaque) spreads radially as in the v1 model, but at a slower rate. As before, tissue degradation (black apoptotic cells and white cleared tissue) has greatest frequency near the original site of infection. **C.** If the number of ACE2 receptors is cut by a factor of 10, fewer virions infect cells, leading to slower viral replication. However, the reduced rate of virus binding and endocytosis leaves more extracellular viral particles to disperse, leading to a larger spread of the region of infection. **D.** Decreasing instead the rate of virus release from internalized ACE2 receptor drastically slows the viral dynamics.

Model behavior: what does the current version teach us?

Except as noted below, all simulation results use the v2 model default parameters, which are supplied in the XML configuration parameter file of the version 0.2.1 core model repository.

In all plots, dark blue cells have 0 assembled virus, pale blue cells have 1-9 assembled virions, grey cells have 10-99 assembled virions, light yellow cells have 100-999 assembled virions, and bright yellow cells contain 1000 or more assembled virions. Black cells are apoptotic, and white spaces show regions devoid of cells (extensive tissue damage).

Infection by a single virus versus a dispersion of virions

Compared to the previous method of initially infecting a single cell with a single virion, the v2 model simulation using the new MOI initialization (MOI=0.01) showed viral particles nucleating multiple infections spread as independent plaques that later merge (**Fig. 2.2 A** and **Fig. 2.2 B**). For higher MOIs, some cells can be infected by more than one virion, leading to faster viral replication.

Targeting the endocytosis cascade versus targeting ACE2 receptor

As more subcellular mechanisms are added to the model, we can ask *what if* questions about potential pharmacologic interventions[10]. Using the v2 model, we first investigated the impact of reducing the number of ACE2 receptors on each cell by a factor of 10 (e.g., by an intervention that targets ACE2 receptor or reduces its expression). We found that while this reduced the number of viral particles infecting each cell (thus slowing replication in individual cells), it paradoxically *accelerated* the spread of the infected region through the tissue (**Fig. 2.2 C**). This phenomenon can be understood by dimensional analysis: the effective transport length scale L of the virus particle is $L = \sqrt{\frac{D}{U}}$, where U is the uptake rate of the viral particles. In the v2 model, U is proportional to the number of unbound external ACE2 receptors. If this number is reduced, then the length scale increases, leading to a faster dissemination of virus particles, exposing more tissue to virus particles, and ultimately infecting more cells earlier in the disease time course. On the other hand, with slower viral replication in individual cells, tissue damage may be delayed. (**Fig. 2.2 D**).

We similarly investigated whether decreasing the rate of viral release from virus-bound endocytosed receptors by reducing $r_{release}$ by a factor of 10. This drastically impaired the spread of the infection: ACE2 receptors trapped and internalized more viral particles, which then replicated more slowly, thus reducing the severity of the infection.

Selected feedback from domain experts within the coalition and the community

The core team reviewed the v2 model and project progress on weekly between April 8, 2020 and May 4, 2020.

The team discussed the potential need for an improved viral replication model. In particular, for low virus counts early in cellular infection, the continuum hypothesis needed for ordinary differential equations may not hold, and non-physical behaviors (e.g., infection by less than a single virus) may prevent the eradication of infections in the model. A discrete modeling approach may be required, although limiting mass transfers (e.g., from R_{EU} to R_{EB}) to integer amounts could also help address this issue. The core team also reaffirmed the need to create a simplified immune system model to continue progress.

The core team also identified needed refinements in xml2jupyter, particularly the ability to run additional analytics on simulation outputs and visualize the results in the Jupyter notebook interface.

The core team formed the subteams, identified chief scientists, and organized the first rounds of subteam meetings. The core team also discussed the need to include subteam updates in the weekly core meetings. This was first implemented in the May 4, 2020 call, and the development cycle discussed above reflects these community-driven changes to team management.

We received additional feedback from the community from a postdoctoral fellow at Barcelona Supercomputing

Center (BSC), who noted the number of virion particles should be constrained to integer values. He also suggested a branch of the sub-models may be reimplemented as stochastic differential equation. In addition, the fellow pointed out that BSC is developing COVID-19 molecular disease maps, mainly by curating interactions between viral and cellular proteins from several data sources and domain experts. It may be possible to "translate" these process descriptions to activity flow models for Boolean network simulations in PhysiBoSS[11]. Future collaborations could test the COIVD-19 tissue simulator developed by this coalition in PhyiBoSS.

Core team discussion and priorities for v3

The highest priority for v3 is to start transitioning the development of the submodels to the subteams, thus moving the project from Phase 1 to Phase 2. In particular, the team was keen to implement a basic immune model.

Version 3 (May 10 - July 27, 2020)

Version 3 focused on implementing a realistic representation of the tissue-level immune response to SARS-CoV-2 and transitioning development of the submodels to the subteams. Due to the complexity of the immune system, a significant portion of time was spent developing a realistic minimal model of the immune response.

This also represents the first model release to begin the transition from Phase 1 to Phase 2: the immune team took on primary development of the C++ for their submodel. This development cycle also performed software hardening on the core PhysiCell toolkit to facilitate complex immune behaviors (particularly phagocytosis and $CD8^+$ T cell attacks on infected cells) while improving multithreading safety and cross-platform compatibility. Moreover, we performed a code refactoring to take advantage of new *cell definition* functionality in PhysiCell 1.7.1, which eased the development of the immune model with multiple cell types.

Key hypotheses

The overall aim of this submodel is to include features of the immune response to SARS-CoV-2 that are specific to the local tissue environment. The main immune cellular components included at this stage are tissue-resident macrophages, infiltrating neutrophils, and $CD8^+$ T cells, which are recruited as the infection progresses. The general pattern of events that this model encompasses are summarized here. When epithelial cells in the tissue become infected with SARS-CoV-2, they secrete chemokines that cause macrophages to migrate towards them following a chemokine gradient. In addition, the infected cells may die as a result of the infection (see the cell response model ***VR***), and dead cells will release factors that cause macrophages to migrate towards them. Macrophages phagocytose dead cells and remove them from the tissue. When macrophages encounter dead cells, they begin to secrete pro-inflammatory cytokines, and phagocytose any dead cell material they find. The result of pro-inflammatory cytokine secretion is the in-

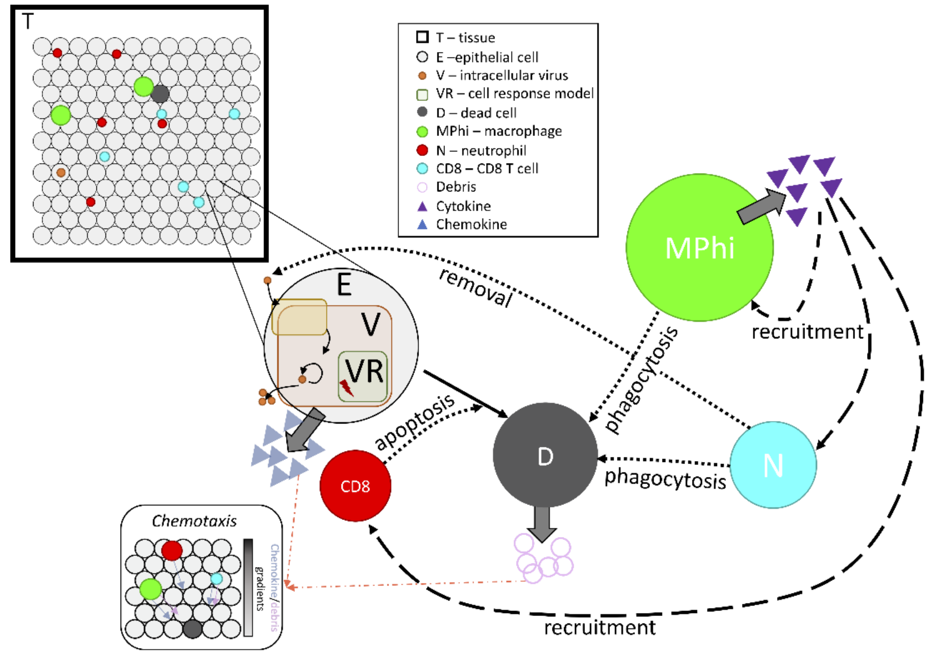

Fig. 3.1: Immune submodel schematic. Immune cells (macrophages, neutrophils, and $CD8^+$ T cells) patrol within the *tissue component* (T), containing multiple *epithelial cells* (E). Cells infected by virus secrete chemokine, which attracts immune cells along the chemokine gradient. $CD8^+$ T cells induce apoptosis in infected cells, creating dead cells that are phagocytosed by macrophages and neutrophils that are attracted along debris gradients. Upon activation, macrophages secrete cytokine that recruits other immune cell types.

filtration of neutrophils into the tissue. Neutrophils are short-lived cells and are replenished in the tissue as long as pro-inflammatory cytokines are still being produced. $CD8^+$ T cells, presumed to be specific for SARS-CoV-2, enter the tissue at a later time, and their role is to kill infected cells. $CD8^+$ T cell entry is dependent upon the presence of pro-inflammatory cytokines. Death of an infected cell is more likely after prolonged contact with one or more $CD8^+$ T cells. $CD8^+$ T cells may also interact with macrophages that have phagocytosed dead cells or virus as these macrophages will now be able to present viral antigens to both $CD4^+$ and $CD8^+$ T cells; however antigen presentation is not directly included in this initial version of the model. As described below, simulations were performed using a field of already infected epithelial cells, and the behavior of the immune cells in this tissue appears to follow the established rules.

Owing to the immune response cascade outlined above, we first integrated macrophages, neutrophils, and $CD8^+$ T cells into the SARS-CoV-2 tissue model. The adaptive immune response in a naïve host begins a few days after innate immune action. For this version, we simplify dynamics by modeling only $CD8^+$ T cells, and we do not yet model antigen presentation via dendritic cells or macrophages. Thus, we assume that $CD8^+$ T cells are recruited around day 4 of infection in response to infected cells and pro-inflammatory cytokine production, where infected cells are killed in response to sustained total contact with one or more $CD8^+$ T cells. It is assumed that immune actions only affect infected cells that are past the eclipse phase and are thus generating virus.

Immune cells travel in a biased correlated random walk along chemical gradients[2]. To control for spatial migration, the submodel contains three diffusing chemicals in addition to free virions: pro-inflammatory cytokines secreted by macrophages, $CD8^+$ T cells, and post-eclipse phase infected cells recruit immune cells into tissue from blood or lymph nodes, which could ultimately be modeled in a separate submodel. We assume all immune cells migrate in the tissue toward infected cells along a chemokine gradient, which is assumed to be secreted by infected cells for simplicity. Infected cells and macrophages also secrete IFN-I, which will reduce viral burst size from neighboring infected cells in future versions of the model.

Model changes

The v2 model was expanded to include the following submodel components (**Fig. 3.1**):

- **T:** tissue (which contains epithelial and other cells, and diffusible factors)
- **RT:** ACE2 receptor trafficking (including virus endocytosis)
- **V:** viral endocytosis, replication, and exocytosis responses
- **VR:** cell response to viral replication, including cell death and IFN synthesis
- **E:** epithelial cell (includes RT, V and VR).
- **D:** dead cell
- **MPhi:** macrophage
- **N:** neutrophil
- **CD8:** $CD8^+$ T cell

Biological hypotheses

The v3 model introduced new assumptions regarding how the infected and dead cells are cleared and how immune cells act in the model (indicated by X.C.Y, where X denotes prototype, C denoted modeling component, and Y denotes a biological hypothesis, for easy reference):

3.T.1 Virus diffuses in the microenvironment with low diffusion coefficient
3.T.2 Virus adhesion to a cell stops its diffusion (acts as an uptake term)
3.T.3 Pro-inflammatory cytokine diffuses in the microenvironment
3.T.4 Pro-inflammatory cytokine is taken up by recruited immune cells
3.T.5 Pro-inflammatory cytokine is eliminated or cleared
3.T.6 Chemokine diffuses in the microenvironment
3.T.7 Chemokine is taken up by immune cells during chemotaxis
3.T.8 Chemokine is eliminated or cleared
3.T.9 Debris diffuses in the microenvironment
3.T.10 Debris is taken up by macrophages and neutrophils during chemotaxis

3.T.11 Debris is eliminated or cleared

3.RT.1 Virus adheres to unbound external ACE2 receptor to become external (virus)-bound ACE2 receptor
3.RT.2 Bound external ACE2 receptor is internalized (endocytosed) to become internal bound ACE2 receptor
3.RT.3 Internalized bound ACE2 receptor releases its virion and becomes unbound internalized receptor. The released virus is available for use by the viral lifecycle model **V**
3.RT.4 Internalized unbound ACE2 receptor is returned to the cell surface to become external unbound receptor
3.RT.5 Each receptor can bind to at most one virus particle.

3.V.1 Internalized virus (previously released in 2.RT.3) is uncoated
3.V.2 Uncoated virus (viral contents) lead to release of functioning RNA
3.V.3 RNA creates viral protein at a constant rate unless it degrades
3.V.4 Viral protein is transformed to an assembled virus state
3.V.5 Assembled virus is released by the cell (exocytosis)

3.VR.1 After infection, cells secrete chemokine
3.VR.2 As a proxy for viral disruption of the cell, the probability of cell death increases with the total number of assembled virions
3.VR.3 Apoptosed cells lyse and release some or all of their contents

3.E.1 Live epithelial cells undergo apoptosis after sufficient cumulative contact time with adhered $CD8^+$ T cells.
3.E.2 Live epithelial cells follow all rules of RT
3.E.3 Live epithelial cells follow all rules of V
3.E.4 Live epithelial cells follow all rules of VR
3.E.5 Dead epithelial cells follow all rules of D.

3.D.1 Dead cells produce debris

3.Mphi.1 Resident (unactivated) and newly recruited macrophages move along debris gradients.
3.MPhi.2 Macrophages phagocytose dead cells
3.Mphi.3 Macrophages break down phagocytosed materials
3.Mphi.4 After phagocytosing dead cells, macrophages activate and secrete pro-inflammatory cytokines
3.Mphi.5 Activated macrophages can decrease migration speed
3.Mphi.6 Activated macrophages have a higher apoptosis rate
3.Mphi.7 Activated macrophages migrate along chemokine and debris gradients
3.Mphi.8 Macrophages are recruited into tissue by pro-inflammatory cytokines.
3.MPhi.9 Macrophages die naturally and become dead cells.

3.N.1 Neutrophils are recruited into the tissue by pro-inflammatory cytokines
3.N.2 Neutrophils die naturally and become dead cells
3.N.3 Neutrophils migrate locally in the tissue along chemokine and debris gradients
3.N.4 Neutrophils phagocytose dead cells and activate
3.N.5 Neutrophils break down phagocytosed materials
3.N.6 Activated neutrophils reduce migration speed
3.N.7 Neutrophils uptake virus

3.CD8.1 $CD8^+$ T cells are recruited into the tissue by pro-inflammatory cytokines
3.CD8.2 $CD8^+$ T cells apoptose naturally and become dead cells

3.CD8.3 CD8⁺ T cells move locally in the tissue along chemokine gradients
3.CD8.4 CD8⁺ T cells adhere to infected cells. Cumulated contact time with adhered CD8⁺ T cells can induce apoptosis (See 3.E.1)

Unit tests

To confirm the dynamics of the immune model qualitatively reproduce the *in-situ* dynamics, we monitored the population numbers of immune cells (macrophages, neutrophils, CD8⁺ T cells) over time and compared with our biological expectations.

Translation to mathematics, rules, and model components

There were no changes to the ACE2 receptor trafficking model ***RT*** or the intracellular viral replication dynamics model ***V.***

Initialization

An initial population of $MPhi_0$ macrophages is seeded randomly throughout the tissue.

Estimates for immune parameters

The diffusion coefficient for the chemokine, pro-inflammatory cytokine, and debris, $D_{\text{chemokine}}, D_{\text{cytokine}}$, and D_{debris}, were set at $555.56\ \mu m^2/min$ which was estimated by Matzavinos *et al* as the diffusion coefficient for monoclonal antibodies[12]. This is equivalent to $8\ \times 10^{-3}\ cm^2/day$, which is similar to $1.25\ \times 10^{-3}\ cm^2/day$ estimated by Liao *et al.*[13,14]. Decay and secretion rates of the pro-inflammatory cytokine, chemokine, and debris were assumed to be equivalent. Decay rates for the signaling substrates, $\lambda_{chemokine}, \lambda_{cytokine}$ and λ_{debris}, were all set to $1.02 \times 10^{-2}/min$, which was estimated as the decay rate of IL-6 by Buchwalder *et al.*[15].The secretion rate for each signaling substrate, $S_{chemokine}, S_{cytokine}$ and S_{debris}, was $0.8254/\rho^*\ 1/min$, obtained through fitting the secretion rate of infected cells to the production of IL-6 by infected basal epithelial cells measured by Ye *et al.*[16] over 25 hour[16]. The uptake rate of pro-inflammatory cytokine, $U_{cytokine}$, was estimated to be $0.0018\ (pg/ml)^{-1}day^{-1}$ from measurements of the binding rate of IL-6 [17]. The chemokine uptake rate, $U_{chemokine}$, was estimated to be $0.0510\ (pgml)^{-1}day^{-1}$ [18]. The uptake rate of debris, U_{debris}, was assumed to be equivalent to that of $U_{chemokine}$, because it acts as a chemoattractant.

Macrophages, neutrophils and CD8⁺ T cells all have different sizes. Macrophages have an average diameter of $21\ \mu m$ [19], giving a total volume of $4849 \mu m^3$. Neutrophils have an average diameter of $14\ \mu m$ [20], giving a total volume of $1437\ \mu m^3$. When activated, CD8⁺ T cells have a diameter of 0.7 $9.7\ \mu m$[21], giving a total volume of $478 \mu m^3$. For all immune cells, the volume of nucleus was assumed to be 10% of the cells total volume[22].

The active migration rate of macrophages and CD8⁺ T cells along the chemokine gradient was $s_{mot,a} = 4 \mu m/min$ based on *in vitro* and *in vivo* measurements of leukocyte chemotaxis rates[23]. We assume these cells have a migration bias of 0.5 (unitless). Neutrophils move faster with stronger bias along the chemokine gradient at $s_{mot} = 19 \mu m/min$, with a bias of 0.91[23]. Once macrophages and neutrophils encounter material to phagocytose, their motility reduces to $s_{mot,p} = 0.4 \mu m/min$ and if a CD8⁺ T cell connects to an infected cell they are no longer motile, i.e., $s_{mot,p} = 0 \mu m/min$. Cells persist on their given trajectory for 5 minutes before a new trajectory is chosen. All immune cells undergo apoptosis at different rates, with neutrophils undergoing apoptosis on average after 18.72 hours[24] (i.e. $a_{I,N} =\ 8.87 \times 10^{-4}$ min^{-1}), macrophages on average every 3.3 days[25] (i.e. $a_{I,M\Phi} = 2.083 \times 10^{-4}$ min^{-1}) and CD8⁺ T on average every 2.5 days (i.e. $a_{I,T} = 2.778 \times 10^{-4}$ min^{-1})[26].

Macrophages are well known for their capability in phagocytosing dead cell debris[27]. As such, we set the probability of a macrophage phagocytosing a dead cell in its neighborhood to $p_{phag,M\Phi} = 1$. To account for the fact that neutrophils split their time between phagocytosing dead cells and taking up virus[28], we set the probability

that neutrophils phagocytose dead cells as $p_{phag,N} = 0.7$.

For the recruitment of immune cells, parameters were chosen to achieve immunologically reasonable arrival times for the immune cell subsets. Neutrophil and macrophage infiltration into tissue is faster than T cell infiltration, with neutrophils and macrophages arriving within 1 to 2 days after infection[29] and $CD8^+$ T cells arriving closer to 4-5 days after infection[30]. The minimum and saturated signal concentrations, ρ_{min} and ρ_{sat}, for macrophages and neutrophils were, therefore, assumed to be equivalent and fixed as $\rho_{min} = 0.1\ \mathrm{substrate}/\mu m^3$ and $\rho_{sat} = 0.3\ \mathrm{substrate}/\mu m^3$. Whereas, $CD8^+$ T cells had higher signal concentrations for their minimum and saturated recruitment signals, i.e. $\rho_{min} = 0.4\ \mathrm{substrate}/\mu m^3$ and $\rho_{sat} = 0.7\ \mathrm{substrate}/\mu m^3$. The recruitment rate for the different immune types was assumed to be equivalent, i.e. $r_{\mathrm{recruit}} = 4 \times 10^{-9}\ \mathrm{cells/min}/\mu m^3$ and the immune recruitment rate time-step was $\Delta t_{immune} = 10\ \mathrm{min}$.

Direct observations of $CD8^+$ T cell-infected cell interactions and quantification of infected cell fate revealed that death required a median of 3.5 distinct $CD8^+$ T cell contacts. Killed infected cells have a cumulative median contact time of 50 min and individual contacts between $CD8^+$ T cells and infected cells lasts on average 8.5 min[31]. We therefore set $T_{\mathrm{CD8\_contact\_death}} = 50\ \mathrm{min}$.

Other implementation notes

This simplified immune model does not yet include many key immune agents, including dendritic cells, natural killer (NK) cells, B cells, antibodies, the complement system, and most cytokines. No anti-inflammatory cytokines are modeled, nor can this model return to homeostasis following potential infection clearance. Dynamics of cytokine binding and unbinding to receptors are also omitted. The model does not yet incorporate known SARS-CoV-2 immune evasion techniques, such as a delayed IFN-I response and lymphopenia (decreased $CD8^+$ T cells) from early in infection. In addition, the antigen-presentation from macrophages and subsequent activation process of $CD4^+$ and $CD8^+$ T cells has been omitted. Many of these important mechanisms are planned for inclusion in future versions. See further discussion in v3 modeling results below.

Software release

The core model associated with the v3 prototype is Version 0.2.1. The nanoHUB app associated with the v3 prototype is Version 3.2. GitHub releases and Zenodo snapshots are given in the Appendix.

The cloud-hosted interactive model can be run at https://nanohub.org/tools/pc4COVID-19.

Model behavior: what does the current version teach us?

Except as noted below, all simulation results use the v3 model default parameters, which are supplied in the XML configuration parameter file of the version 0.3.2 core model repository.

In all plots, dark blue cells have 0 assembled virus, pale blue cells have 1-9 assembled virions, grey cells have 10-99 assembled virions, light yellow cells have 100-999 assembled virions, and bright yellow cells contain 1000 or more assembled virions. Black cells are apoptotic, and white spaces show regions devoid of cells (extensive tissue damage). Unactivated macrophages are green, activated macrophages are magenta, $CD8^+$ T cells are red, and neutrophils are cyan. Apoptotic immune cells are light orange.

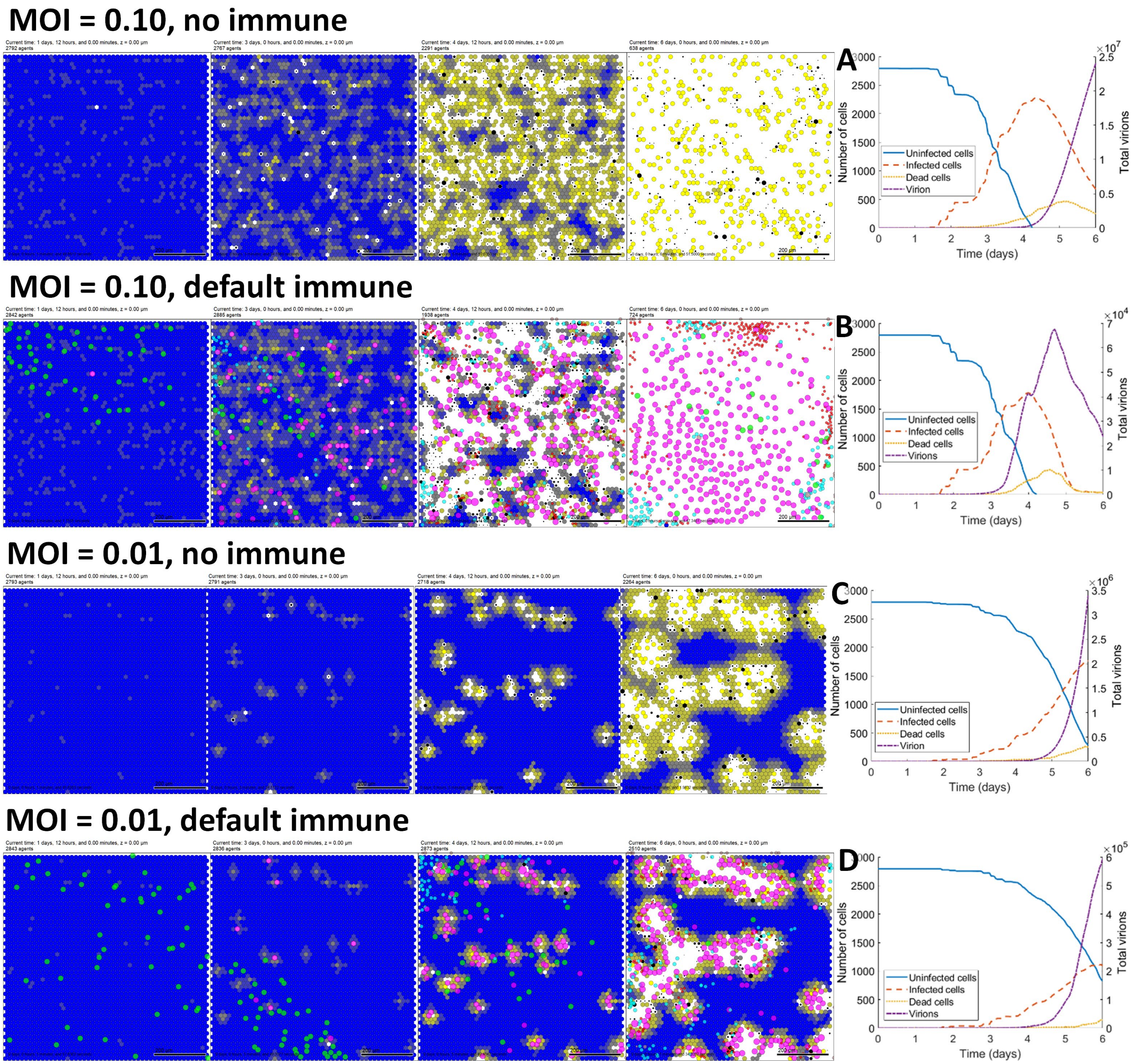

Fig. 3.2: Version 3 model at 46, 72, 108, and 144 hours (default immune settings). In all plots, epithelial cells are colored from blue (no assembled virions) to bright yellow (1000 or more virions). Black cells are apoptotic, and white regions show damaged tissues where apoptotic cells have degraded to expose (unmodeled) basement membrane. Green cells are macrophages, magenta cells are activated macrophages, cyan cells are neutrophils, and red cells are $CD8^+$ T cells. Bar: 200 μm.

Rows 1-2: Simulated dynamics starting with an MOI (multiplicity of infection) of 0.10 without an immune response (**Row 1**) and with an immune response (**Row 2**). **Plots A-B** show uninfected (blue), infected (orange), and dead (yellow) cell counts and total extracellular virion (purple) without an immune response (**A**) and with an immune response (**B**). The immune response clears infected and dead cells more quickly and limits the maximum extracellular viral load, but the underlying tissue is completely destroyed.

Rows 3-4: Simulated dynamics starting with an MOI (multiplicity of infection) of 0.01 without an immune response (**Row 3**) and with an immune response (**Row 4**). **Plots C-D** show uninfected, infected, and dead cell counts and total extracellular virion without an immune response (**C**) and with an immune response (**D**) (same coloring as A-B). The immune response slows the spread of the infection and increases uninfected cell survival.

Impact of adding the immune response (default parameters)

Figs. 3.2-3.5 demonstrate the results of simulating SARS-Cov-2 infection under different parameter regimes. **Fig. 3.2** simulates the dynamics without and with an immune response for a MOI of 0.10 (top results) and 0.01 (bottom results), using the default immune parameters. When the MOI is 0.10, most of tissue is destroyed, either

by the virus (**Fig. 3.2 top row**) or the immune system (**Fig. 3.2 second row**). Reducing the MOI allows some of the tissue to survive the infection in the absence (**Fig. 3.2, third row**) or presence (**Fig. 3.2, fourth row**) of the immune response. All subsequent model results will show a MOI of 0.01 to highlight differences in dynamics to changes in immune parameters. As discussed below, this may not be necessary once the interferon response in infected cells is added to the model.

At high MOI, macrophages are rapidly activated (**Fig. 3.2, first panel in row 2**), and the release of inflammatory cytokines results in the infiltration of CD8$^+$ T cells by day 3 (**Fig. 3.2, second panel in row 2**). The CD8$^+$ T cells kill all infected cells and the tissue is destroyed. At low MOI (0.01), the number of tissue cells surviving is greater (**Fig. 3.2, fourth row, panel 4**). However, macrophage activation is delayed, which further delays the infiltration of CD8$^+$ T cells. At the end of the simulation, on day 6, there is a large number of infected cells (**Fig. 3.2 D**), and the viral titers are still rising. This suggests that, under these default conditions, the lower MOI simply delays the

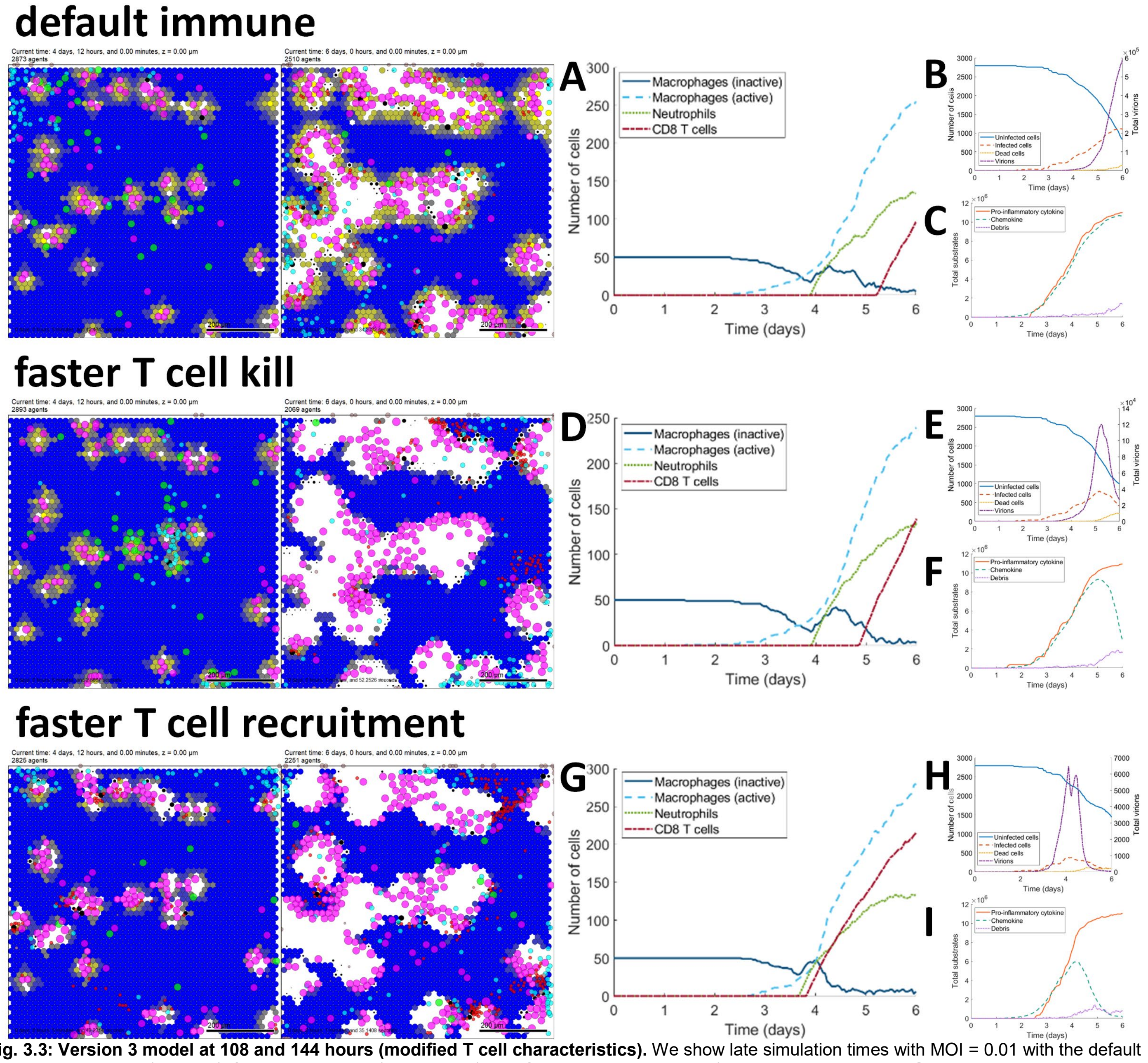

Fig. 3.3: Version 3 model at 108 and 144 hours (modified T cell characteristics). We show late simulation times with MOI = 0.01 with the default immune parameters (**top row**), faster T cell killing (**middle row**), and faster T cell recruitment (**bottom row**). **A, D, and G** plot immune cell dynamics, **B, E, and H** plot infected cell and virion dynamics, and **C, F, and I** plot changes in signaling factors for each simulated scenario. Faster T cell killing and faster T cell recruitment both slow infection dynamics and (nearly) control the infection by 6 days. Cells are colored as in Fig. 3.2.

infection; the tissue is completely destroyed if the simulation is continued beyond 6 days, as seen with the higher MOI of 0.10 (result not shown).

Changing T cell parameters

In the initial model v3 simulation results introduced in **Fig. 3.2**, it appeared that the dynamics of CD8$^+$ T cell recruitment and activation relative to viral replication might be important. Thus, we varied some of the immune cell parameters to determine whether the survival of the tissue could be improved. **Fig. 3.3 (second row)** shows the results when the rate of CD8$^+$ T cell killing was doubled by reducing the threshold contact time for cell death from 50 min to 25 min. Even macrophage, neutrophil, and CD8$^+$ T cell recruitment were slightly reduced compared to the default parameters (compare **Fig. 3.3 D** to **Fig. 3.3 A**), the increased ability of CD8$^+$ T cells to kill infected cells results in fewer infected cells and the viral titers are falling (**Fig. 3.3 B and E**).

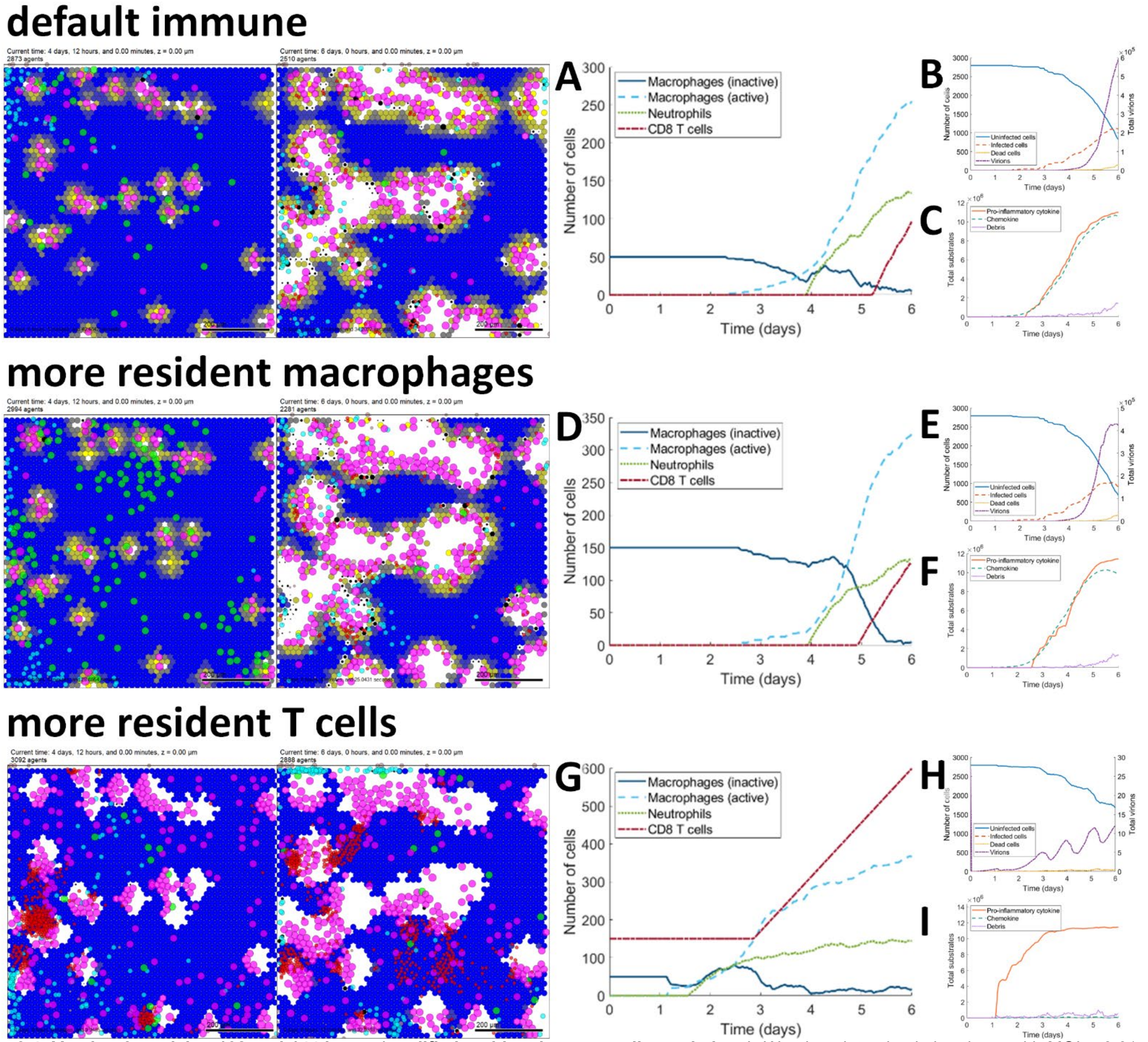

Fig. 3.4: Version 3 model at 108 and 144 hours (modified resident immune cell populations). We show late simulation times with MOI = 0.01 with the default immune parameters (**top row**), increased resident macrophages (**middle row**), and addition of resident T cells (**bottom row**). **A, D, and G** plot immune cell dynamics, **B, E, and H** plot infected cell and virion dynamics, and **C, F, and I** plot changes in signaling factors for each simulated scenario. Increasing the resident macrophage population did not appreciably inhibit the infection, while introducing resident T cells reduced viral titers and better controlled the infection. Cells are colored as in Fig. 3.2.

We also increased the recruitment rate of CD8+ T cells to the tissue in response to inflammatory cytokines by reducing $\rho_{\min}$ from 0.4 to 0.1, and by reducing ρ_{sat} from 0.7 to 0.4; see **Fig. 3.3 (third row)**. This resulted in greater sparing of tissue, and the virus was completely cleared under these conditions (**Fig. 3.3 H**).

Changing resident immune cell populations

We also investigated whether the outcome would be influenced by the number of macrophages and T cells that might be resident within the tissue at the time of infection. We first increased the initial number of macrophages from 50 to 150 (**Fig. 3.4, second row**). Interestingly, this did not result in an improved outcome over that seen with the default parameters (compare **Fig. 3.4, top row**). CD8+ T cell recruitment was enhanced (**Fig. 3.4 D**) but the viral load was continuing to increase at day 6 (**Fig. 3.4 B**), suggesting that increased number of macrophages did not result in more virus control. This could be because in the present model macrophages essentially removed dead cells and do not have a role in killing infected cells. Moreover, because the model's macrophages cannot activate until dead infected cells are present, increasing the number of macrophages cannot trigger a faster immune response. This suggests that after reaching a minimal number of macrophages, adding more resident macrophages has a minimal impact on improving immune response.

In contrast, the presence of resident T cells (**Fig. 3.4, bottom row**) did result in an improved outcome, with more tissue spared and essentially no viral replication (**Fig. 3.4 H**). The final viral count on day 6 was under 15 as T cells were able to kill every infected cell before it could release a significant amount of assembled virions (**Fig. 3.4 H**). This drastically slowed the spread of the infection through the tissue. We also note that the faster T cell killing resulted in earlier accumulation of apoptotic cells, leading to faster activation of macrophages and hence accelerated immune cell recruitment (**Fig. 3.4 G**).

The addition of an interferon response (which could prevent nearby cells from endocytosing these few virions)

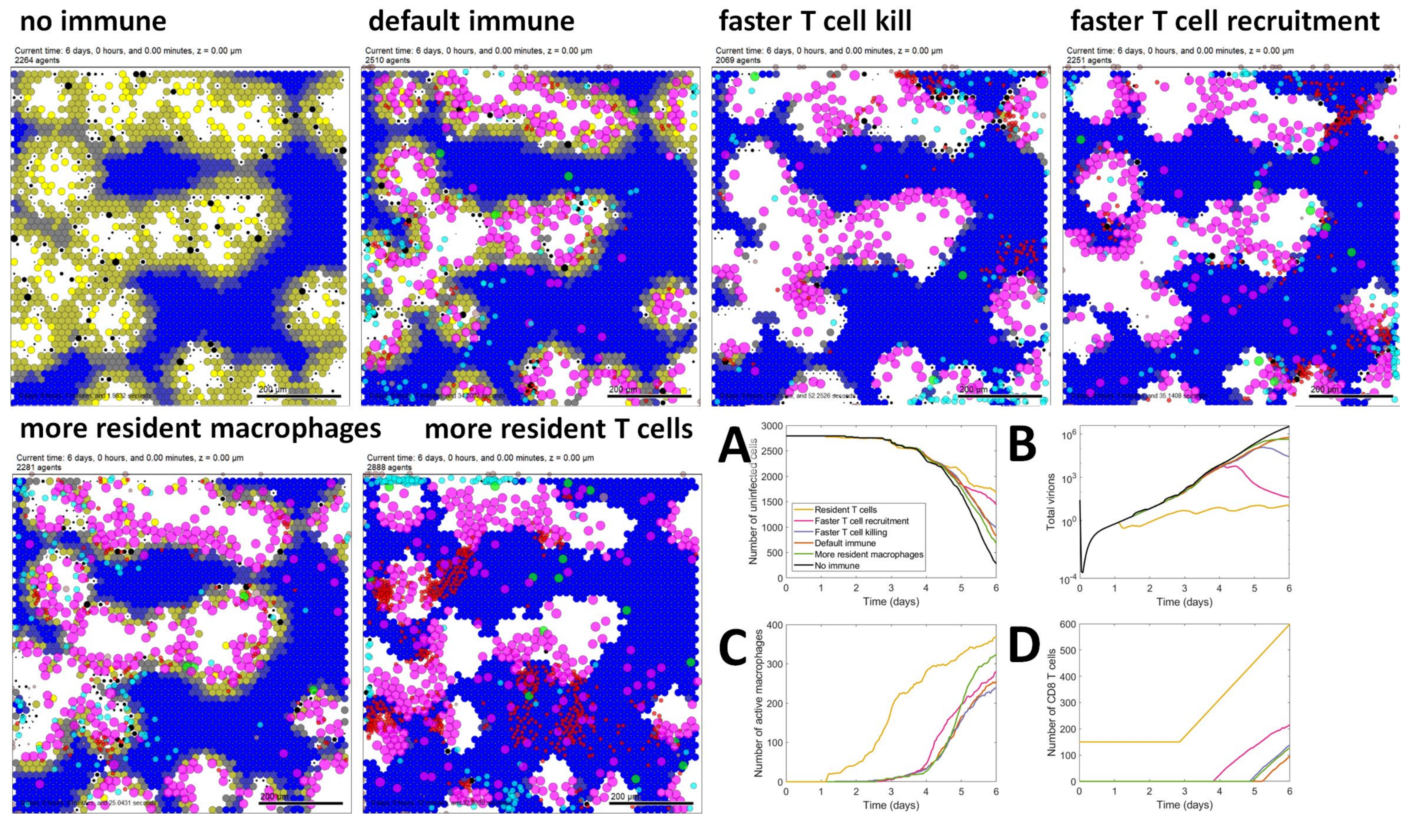

Fig. 3.5: Comparison of version 3 model runs. We show late simulation times with MOI = 0.01 with the default immune parameters (**top row**), increased resident macrophages (**middle row**), and addition of resident T cells (**bottom row**). **A, D, and G** plot immune cell dynamics, **B, E, and H** plot infected cell and virion dynamics, and **C, F, and I** plot changes in signaling factors for each simulated scenario. Increasing the resident macrophage population did not appreciably inhibit the infection, while introducing resident T cells reduced viral titers and better controlled the infection. Cells are colored as in Fig. 3.2.

or antibodies (which could directly neutralize the released virions) could potentially completely control the infection in future versions of the model.

Further comparison of v3 model results

When we compare these simulations side by side (**Fig. 3.5**) we can see that the two scenarios with the best outcomes are those in which the recruitment rate of T cells is increased and when resident T cells are included. These resulted in the largest amount of surviving uninfected tissue (**Fig. 3.5 A**) as well as viral clearance (**Fig. 3.5 B**). It is interesting to note that the increase in recruitment resulted in T cells arriving in the tissue only one day earlier than the default scenario (**Fig. 3.5 D**), but this had a dramatic effect. Under these conditions, the virus expanded but was cleared within 6 days of infection. This model provides a mechanism to explore how $CD8^+$ T cell recruitment could be enhanced in COVID-19 patients. While the presence of resident T cells prevented the viral infection from propagating there was still a significant amount of tissue loss in the current model.

Discussion of v3 model results

The inclusion of the basic tissue immune system to the model has provided some interesting new avenues for research. It is important to note some of the caveats with the existing model. In order to be able to reliably see the beneficial effect of the immune response we had to reduce the viral MOI tenfold. This was because at the higher MOI, either the virus killed the tissue in the absence of the immune response, whereas when the immune response was present it was the immune response that destroyed the tissue (although in this model, the immune cells only destroyed infected cells and did not damage uninfected cells). It is important to note that the present model does not include the cellular interferon response, which would have the effect of slowing viral replication and making more cells resistant to viral infection. When this aspect is included, it is possible that the MOI will not need to be reduced to the same degree.

The results of the simulations so far suggest that controlling the recruitment of $CD8^+$ T cells is a critical parameter leading to viral clearance and reduced tissue destruction. Recent single cell RNA sequencing studies of cells from bronchoalveolar lavage fluid from COVID-19 patients have demonstrated that individuals with moderate disease have increased numbers of $CD8^+$ T cells infiltrating the lungs compared to those with severe disease[32]. This suggests that aspects of the model that can influence this parameter should be explored further. This could include the addition of a more detailed lymph node model in which the kinetics of activation and proliferation of specific $CD8^+$ T cells can be explored.

The presence of resident $CD8^+$ T cells prior to infection effectively prevented viral replication, although at the cost of some tissue damage. It is known that resident memory $CD8^+$ T cells in the lung can provide protection from subsequent infection[33]. It is possible that certain individuals infected with SARS-CoV-2 have been exposed to related coronaviruses and recent studies have observed that up to 40% of unexposed individuals have detectable T cell responses to SARS-CoV-2 proteins[34,35]. Moreover, presence of $CD8^+$ T cells is plausible in uninfected tissues that are adjacent to infected tissues: an initial infection may resemble the default scenario of extensive damage, but the recruited $CD8^+$ T cells could provide protection to nearby tissues from such damage. In the present simulations, it was assumed that all of the resident $CD8^+$ T cells could recognize SARS-CoV-2, but, in reality, only a small fraction of these T cells would be specific for the virus. This can be explored in future simulations.

The fact that increasing the macrophage number did not affect the outcome could reflect the fact that in the present model, these cells have no role in removing infected cells and only remove cells once they have died. Thus, they do not influence the infection beyond secreting inflammatory cytokines that are necessary for the recruitment of $CD8^+$ T cells and neutrophils to the tissue. In subsequent model versions it may be possible to add an interaction between macrophages that have taken up virus and/or dead infected cells with $CD8^+$ T cells. This interaction could further activate macrophages and allow them to kill infected cells before they die.

Selected feedback from domain experts within the coalition and the community

During the extensive v3 model development cycle, the modeling coalition met weekly to discuss and refine the model assumptions and record feedback for future work. Several members presented results at virtual seminars

and virtual conferences; feedback from audience questions and interactions are also reflected here.

Several members noted that because the ACE2 and viral replication dynamics models use ODEs, it is possible that a cell in a low extracellular viral concentration could endocytose less than one full virion. These cells (with < 1 endocytosed virion) can still synthesize viral proteins and assemble virions in the current ODE model, leading to artificially fast viral propagation in the tissue. Future model versions must compensate for this by only allowing integer numbers of virions to uncoat and replicate. Moreover, for a coated virus such as SARS-CoV-2, we must ensure that only live cells release assembled virions; this is the default model setting in v3 (lysed cells release 0% of their assembled virions by default settings).

It was also widely noted the antiviral effects of interferons should be incorporated to more accurately model the rate of spread of an infection. This is of critical importance in light of recent news reports that interferon beta treatment is emerging as a treatment option[36,37]. Within infected cells, further feedbacks (e.g., on endocytosis) may be needed to prevent further re-infection of those cells. We may also need to model heterogeneity in cells' susceptibility to infection and cytokine production. Cytokine production by infected cells may or may not vary with the amount of virus in those cells.

While the initial immune model in v3 was able to address open questions on the effect of T cells, further work is needed, including explicit cross-talk and feedback between pro- and anti-inflammatory cytokines. Future models should include more neutrophil behaviors, including their own secretion of pro-inflammatory cytokines.

Future immune models should also include activation of antigen-presenting cells (APCs) and T helper cells. Macrophages that have taken up dead infected cells should present antigens to $CD4^+$ and $CD8^+$ T cells. Interactions of macrophages with $CD4^+$ T cells should render them capable of killing infected cells, particularly those with antibodies bound to their surface.

While lymphopenia is a topic of significant clinical interest[38,39], there is currently no mechanism for it in the model. This could be addressed by linking T cell death to the level of inflammatory cytokines, since the degree of lymphopenia has been correlated with levels of IL-6, IL-10 and TNF-α[38]. Also, the current model only captures a "cytokine storm" in the sense that as more macrophages are recruited, they also secrete pro-inflammatory cytokines, leading to an accelerated accumulation of cytokines as macrophages accumulate. Future models will need to address this more mechanistically.

In terms of the model development process, we found that there may need to be more flexibility in the length of each development cycle. The transition from Phase 1 to Phase 2 of the project requires substantial training of new developers and creation of software infrastructure. The two-to-three week development cycle noted above is more appropriate to late Phase 2 when all this infrastructure is in place and model changes are more minor from one version to the next.

Core team discussions and priorities for v4

In the next development cycle, we plan to introduce type I IFN secretion and its antiviral effects in nearby uninfected cells, particularly reduced receptor endocytosis and viral replication. This will allow us to investigate recent reports on interferon beta

We also plan to introduce a refined, expert-driven model of viral replication to avoid the model artifacts discussed above and to ensure that cells can only replicate virus if they are infected by at least one *whole* virion, that they can only replicate viral proteins if they have at least one full set of viral RNA coated, and they can only assemble virions if they have at least one set of replicated viral proteins. Adding discrete / integer checks on these behaviors may introduce delays analogous to delay differential equations that improve model realism.

We plan to continue refining the immune response submodel as addressed above, with a focus on improving pro- and anti-inflammatory responses and adding missing immune cell types. We plan to link this with a new lymph node model that will more mechanistically regulate T cell expansion, "education" and recruitment.

Discussion

Within three weeks of the World Health Organization's declaration of a global pandemic of COVID-19[40], community-based prototyping built upon an existing PhysiCell 3D cell-modeling framework to rapidly develop Version 1 of an intracellular and tissue-level model of SARS-CoV-2[2]. A growing coalition of domain experts from across STEM fields are working together to ensure accuracy and utility of this agent-based model of intracellular, extracellular, and multicellular SARS-CoV-2 infection dynamics. Version 1 development underscored the necessity of clearly explaining model components, defining scope, and communicating progress as it occurs for invaluable real-time feedback from collaborators and the broader community. This rapid prototyping already helped in growing the coalition and recruiting complementary expertise; for instance, a team modeling lymph node dynamics and immune infiltration joined during the Version 1 cycle after seeing initial progress.

The version 1 prototype also showed the scientific benefit of rapid prototyping: even a basic coupling between extracellular virion transport, intracellular replication dynamics, and viral response (apoptosis) showed the direct relationship between the extracellular virion transport rate and the spread of infection in a tissue. More importantly, it showed that for viruses that rapidly create and exocytose new virions, release of additional assembled virions at the time of cell death does not significantly speed the spread of infection. Moreover, decreasing the cell tolerance to viral load does not drastically change the rate at which the infection spreads, but it does accelerate the rate of tissue damage and loss, which could potentially trigger edema and ARDS earlier. This suggests that working to slow apoptosis may help preserve tissue integrity and delay adverse severe respiratory responses. That such a simple model could already point to actionable hypotheses for experimental and clinical investigations points to the value of rapid model iteration and investigation, rather than waiting for a "perfect" model that incorporates all processes with mechanistic molecular-scale detail.

Version 2 showed promise of increasing mechanistic details to evaluate potential inhibitors. For example, it was found that that reducing the expression of ACE2 receptors could paradoxically lead to faster spread of the infection across the tissue, although the individual infected cells would replicate virus more slowly. On the other hand, taking advantage of high receptor expression but interfering with viral release from internalized receptors may help slow infectious dynamics. Generally, adding sufficient actionable cell mechanisms to the model framework allows us to ask pharmacologically-driven questions on potential pharmacologic interventions, and how these findings are affected by heterogeneity, stochasticity, and the multiscale interactions in the simulated tissue.

Version 3 allowed our first investigations of immune system responses. We found that T cell behaviors are critical to controlling the spread of an infection through the tissue. In particular, rapid recruitment as well as the presence of "educated" $CD8^+$ T cells prior to infection (e.g., after responding to infection in a nearby tissue) had a significant protective effect, even in the current model that does not explicitly model antibodies. This is consistent with emerging studies that link T cell responses to patients with the best recovery[32,34,35].

As work on future versions progresses, teams will work in parallel on submodels to add, parameterize, and test new model components. It will be important to balance the need for new functionality with the requirement for constrained scope, while also balancing the importance of model validation with timely dissemination of results. Thus, this preprint will be updated with every development cycle to invite feedback and community contributions. Between cycles, the most up-to-date information about this model can be found at http://COVID-19.physicell.org.

References

1 Heiland, R., Mishler, D., Zhang, T., Bower, E. & Macklin, P. xml2jupyter: Mapping parameters between XML and Jupyter widgets. *J Open Source Softw* **4**, doi:10.21105/joss.01408 (2019).

2 Ghaffarizadeh, A., Heiland, R., Friedman, S. H., Mumenthaler, S. M. & Macklin, P. PhysiCell: An open source physics-based cell simulator for 3-D multicellular systems. *PLoS Comput Biol* **14**, e1005991, doi:10.1371/journal.pcbi.1005991 (2018).

3 Macklin, P., Edgerton, M. E., Thompson, A. M. & Cristini, V. Patient-calibrated agent-based modelling of ductal carcinoma in situ (DCIS): from microscopic measurements to macroscopic predictions of clinical progression. *J Theor Biol* **301**, 122-140, doi:10.1016/j.jtbi.2012.02.002 (2012).
4 Keck, F. *et al.* Mitochondrial-Directed Antioxidant Reduces Microglial-Induced Inflammation in Murine In Vitro Model of TC-83 Infection. *Viruses* **10**, doi:10.3390/v10110606 (2018).
5 Keck, F. *et al.* Altered mitochondrial dynamics as a consequence of Venezuelan Equine encephalitis virus infection. *Virulence* **8**, 1849-1866, doi:10.1080/21505594.2016.1276690 (2017).
6 Lowe, D. *Angiotensin and the Coronavirus*, <https://blogs.sciencemag.org/pipeline/archives/2020/03/17/angiotensin-and-the-coronavirus> (2020).
7 Beauchemin, C., Forrest, S. & Koster, F. T. 23-36 (Springer Berlin Heidelberg).
8 Sniekers, Y. H. & van Donkelaar, C. C. Determining Diffusion Coefficients in Inhomogeneous Tissues Using Fluorescence Recovery after Photobleaching. *Biophysical Journal* **89**, 1302-1307, doi:10.1529/biophysj.104.053652 (2005).
9 Wang, H. *et al.* SARS coronavirus entry into host cells through a novel clathrin- and caveolae-independent endocytic pathway. *Cell Research* **18**, 290-301, doi:10.1038/cr.2008.15 (2008).
10 Macklin, P. When Seeing Isn't Believing: How Math Can Guide Our Interpretation of Measurements and Experiments. *Cell Systems* **5**, 92-94, doi:10.1016/j.cels.2017.08.005 (2017).
11 Letort, G. *et al.* PhysiBoSS: a multi-scale agent-based modelling framework integrating physical dimension and cell signalling. *Bioinformatics* **35**, 1188-1196, doi:10.1093/bioinformatics/bty766 (2019).
12 Matzavinos, A. Mathematical modelling of the spatio-temporal response of cytotoxic T-lymphocytes to a solid tumour. *Mathematical Medicine and Biology* **21**, 1-34, doi:10.1093/imammb/21.1.1 (2004).
13 Liao, K.-L., Bai, X.-F. & Friedman, A. The role of CD200–CD200R in tumor immune evasion. *Journal of Theoretical Biology* **328**, 65-76, doi:10.1016/j.jtbi.2013.03.017 (2013).
14 Secomb, T. W., Liao, K.-L., Bai, X.-F. & Friedman, A. Mathematical Modeling of Interleukin-27 Induction of Anti-Tumor T Cells Response. *PLoS ONE* **9**, doi:10.1371/journal.pone.0091844 (2014).
15 Buchwalder, P.-A., Buclin, T., Trinchard, I., Munafo, A. & Biollaz, J. Pharmacokinetics and Pharmacodynamics of IFN-β1a in Healthy Volunteers. *Journal of Interferon & Cytokine Research* **20**, 857-866, doi:10.1089/10799900050163226 (2000).
16 Ye, S., Lowther, S., Stambas, J. & Sandri-Goldin, R. M. Inhibition of Reactive Oxygen Species Production Ameliorates Inflammation Induced by Influenza A Viruses via Upregulation of SOCS1 and SOCS3. *Journal of Virology* **89**, 2672-2683, doi:10.1128/jvi.03529-14 (2015).
17 Tenhumberg, S. *et al.* Structure-guided Optimization of the Interleukin-6 Trans-signaling Antagonist sgp130. *Journal of Biological Chemistry* **283**, 27200-27207, doi:10.1074/jbc.M803694200 (2008).
18 Arnaud, P. Les différents interférons : Pharmacologie, mécanismes d'action, tolérance et effets secondaires. *La Revue de Médecine Interne* **23**, 449S-458S, doi:10.1016/s0248-8663(02)00659-8 (2002).
19 Krombach, F. *et al.* Cell size of alveolar macrophages: an interspecies comparison. *Environmental Health Perspectives* **105**, 1261-1263, doi:10.1289/ehp.97105s51261 (1997).
20 *Blood and Bone Marrow Pathology*. (2011).
21 Du, M., Kalia, N., Frumento, G., Chen, F. & Zhang, Z. Biomechanical properties of human T cells in the process of activation based on diametric compression by micromanipulation. *Medical Engineering & Physics* **40**, 20-27, doi:10.1016/j.medengphy.2016.11.011 (2017).
22 Heyden, S. & Ortiz, M. Investigation of the influence of viscoelasticity on oncotripsy. *Computer Methods in Applied Mechanics and Engineering* **314**, 314-322, doi:10.1016/j.cma.2016.08.026 (2017).
23 Trepat, X., Chen, Z. & Jacobson, K. in *Comprehensive Physiology* (2012).
24 Craig, M., Humphries, A. R. & Mackey, M. C. An upper bound for the half-removal time of neutrophils from circulation. *Blood* **128**, 1989-1991, doi:10.1182/blood-2016-07-730325 (2016).
25 Eftimie, R. & Eftimie, G. Tumour-associated macrophages and oncolytic virotherapies: a mathematical investigation into a complex dynamics. *Letters in Biomathematics* **5**, S6-S35, doi:10.30707/LiB5.2Eftimiea (2018).
26 Kim, P. S., Lee, P. P. & Levy, D. Modeling regulation mechanisms in the immune system. *Journal of Theoretical Biology* **246**, 33-69, doi:10.1016/j.jtbi.2006.12.012 (2007).

27 Klöditz, K. & Fadeel, B. Three cell deaths and a funeral: macrophage clearance of cells undergoing distinct modes of cell death. *Cell Death Discovery* **5**, doi:10.1038/s41420-019-0146-x (2019).
28 Selders, G. S., Fetz, A. E., Radic, M. Z. & Bowlin, G. L. An overview of the role of neutrophils in innate immunity, inflammation and host-biomaterial integration. *Regenerative Biomaterials* **4**, 55-68, doi:10.1093/rb/rbw041 (2017).
29 Brandes, M., Klauschen, F., Kuchen, S. & Germain, Ronald N. A Systems Analysis Identifies a Feedforward Inflammatory Circuit Leading to Lethal Influenza Infection. *Cell* **154**, 197-212, doi:10.1016/j.cell.2013.06.013 (2013).
30 Janeway Jr, C. A., Travers, P. T., Walport, P. & Shlomchik, M. J. *Immunobiology*. 5th edn, (Garland Science, 2001).
31 Halle, S. *et al.* In Vivo Killing Capacity of Cytotoxic T Cells Is Limited and Involves Dynamic Interactions and T Cell Cooperativity. *Immunity* **44**, 233-245, doi:10.1016/j.immuni.2016.01.010 (2016).
32 Liao, M. *et al.* Single-cell landscape of bronchoalveolar immune cells in patients with COVID-19. *Nature Medicine* **26**, 842-844, doi:10.1038/s41591-020-0901-9 (2020).
33 McMaster, S. R., Wilson, J. J., Wang, H. & Kohlmeier, J. E. Airway-Resident Memory CD8 T Cells Provide Antigen-Specific Protection against Respiratory Virus Challenge through Rapid IFN-γ Production. *The Journal of Immunology* **195**, 203-209, doi:10.4049/jimmunol.1402975 (2015).
34 Grifoni, A. *et al.* Targets of T Cell Responses to SARS-CoV-2 Coronavirus in Humans with COVID-19 Disease and Unexposed Individuals. *Cell* **181**, 1489-1501.e1415, doi:10.1016/j.cell.2020.05.015 (2020).
35 Sette, A. & Crotty, S. Pre-existing immunity to SARS-CoV-2: the knowns and unknowns. *Nature Reviews Immunology* **20**, 457-458, doi:10.1038/s41577-020-0389-z (2020).
36 Wadman, M. Can boosting interferons, the body's frontline virus fighters, beat COVID-19? *Science*, doi:10.1126/science.abd7137 (2020).
37 Rowlatt, J. *Coronavirus: Protein treatment trial 'a breakthrough'*, <https://www.bbc.com/news/health-53467022> (2020).
38 Diao, B. *et al.* Reduction and Functional Exhaustion of T Cells in Patients With Coronavirus Disease 2019 (COVID-19). *Frontiers in Immunology* **11**, doi:10.3389/fimmu.2020.00827 (2020).
39 Mathew, D. *et al.*, doi:10.1101/2020.05.20.106401 (2020).
40 Organization, W. H. *WHO Director-General's opening remarks at the media briefing on COVID-19 - 11 March 2020*, <https://www.who.int/dg/speeches/detail/who-director-general-s-opening-remarks-at-the-media-briefing-on-covid-19---11-march-2020> (2020).
